# The combination of synthesis and ultra-high-resolution NMR spectroscopy reveals the correct structure of caylobolide A

**DOI:** 10.1038/s44160-025-00762-2

**Published:** 2025-03-13

**Authors:** Malcolm R. P. George, Max Deering, Daniele Fiorito, Keren Solomon, Kevin Tidgewell, Adam Noble, Craig P. Butts, Varinder K. Aggarwal

**Affiliations:** 1 School of Chemistry, University of Bristol, Bristol, UK; 2 Department of Pharmaceutical Sciences, University of Kentucky, Lexington, KY USA; 3 Present Address: Dipartimento di Chimica, Materiali e Ingegneria Chimica ‘Giulio Natta’, Politecnico di Milano, Milan, Italy

**Keywords:** Natural product synthesis, Structure elucidation

## Abstract

Polyketide-derived natural products bearing repeat 1,5-polyols are commonly encountered but their structures are notoriously difficult to determine using spectroscopic techniques. The presence of distal 1,5-diol moieties frequently leads to spectral overlap and chemical shift degeneracy, giving rise to ambiguity in their assignment. Caylobolide A is a representative member of this class of natural products, bearing a 36-membered lactone, with six 1,5-diol units and two 1,3-diol units. Its partial structure had been proposed, but only 4 of the 12 stereogenic centres had been assigned. Here we report a blueprint for the structure determination of this class of natural products, comprising the use of ultra-high-resolution NMR spectroscopy, Mosher’s ester analysis and an efficient mixed isomer synthesis to unveil the correct structure of caylobolide A. With this approach, the partial stereochemistry proposed and the position of the triol unit within the carbon chain has been reassigned, culminating in the total synthesis of caylobolide A in 17 steps.

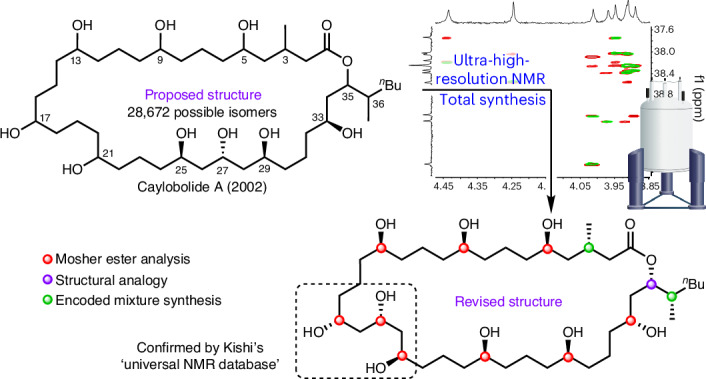

## Abstract (web summary)

The structures of 1,5-polyol-containing polyketide-derived natural products are notoriously difficult to determine using spectroscopic techniques due to spectral overlap and chemical shift degeneracy. Now, a blueprint for the structure determination of this class of natural products is reported, combining ultra-high-resolution NMR spectroscopy and synthesis to unveil the correct structure of caylobolide A.

## Main

Marine cyanobacteria are a valuable source of a wide variety of secondary metabolites with a diverse range of biological applications that include antifungal, antibacterial, antimalarial, antiviral, anti-inflammatory and cytotoxic activity^1^. These metabolites enable the survival of marine cyanobacteria in extreme environments with high salinity, extreme temperatures, high ultraviolet and heavy metals, and hence their structures have become highly specialized to these niche surroundings^2^. The structures are often unusual, of great interest in natural product research and can serve as a rich source of inspiration for the development of new therapeutics. Marine macrolides represent a subsection of marine cyanobacterial secondary metabolites, in which a macrocyclic lactone is the key defining structural feature, typically decorated with alcohol and alkyl groups^3^.

**Fig. 1:**
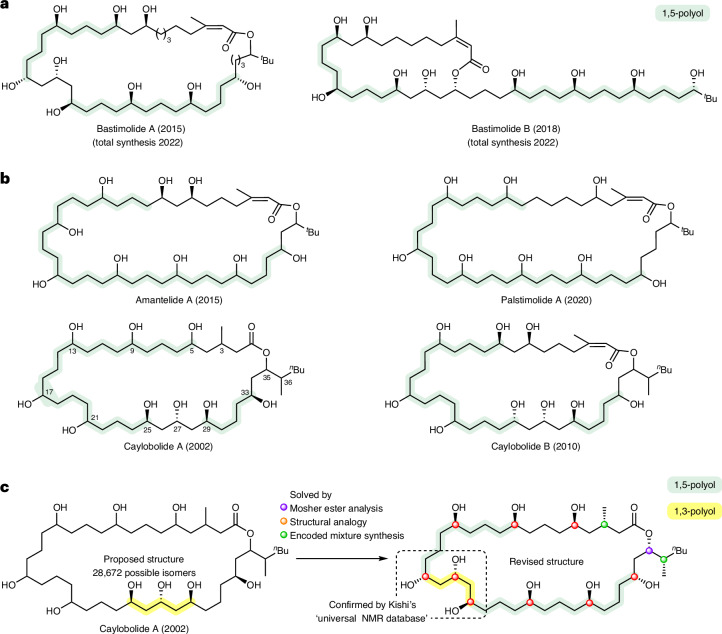
Structurally related marine macrolides possessing a 1,5-polyol backbone. **a**, Solved structures: bastimolide A^9^ and B.^12^ **b**, Unknown structures: amantelide A,^8^ palstimolide A,^13^ caylobolide A^6^ and caylobolide B.^7,11^ **c**, This work, solving the absolute configuration of caylobolide A. The 1,5-polyol regions of each natural product are highlighted in green. The number of possible isomers reflects the 12 stereogenic centres (2^12^) and the seven possible positions for the 1,3,5-triol (between carbons 5/9, 9/13, 13/17, 17/21, 21/25, 25/29, 29/33).

To fully explore the biological potential of these natural products, it is essential that their structure is correctly identified and that synthetic routes are developed. 1,5-Diols are a frequently encountered structural motif found in marine macrolides, but they are also challenging structural motifs to analyse^4,5^. For example, the distal relationship between functionalities gives rise to near-degenerate chemical shifts (and hence spectral overlap) in key regions of NMR spectra, leading to a high degree of structural ambiguity, especially when repeat units are present. A representative family of such natural products is shown in Fig. 1a^6–13^; only limited stereochemical assignments have been possible for most of them. The exception is bastimolide A whose structure was solved by X-ray crystallography after derivatization to the corresponding nona-*p*-nitrobenzoate^9^, and subsequently bastimolide B was assigned by analogy^12^ and confirmed by total synthesis^14^. In the absence of single-crystal X-ray analysis, the skeleton is generally identified by NMR spectroscopy, with stereochemical assignment frequently achieved using Mosher’s ester analysis^15,16^. In the case of caylobolide A, a partial stereochemical assignment was made based on this technique but severe convolution of NMR spectroscopic signals prevented this from offering a complete solution in the original report^6^. Despite not knowing the stereochemical structure of caylobolide A, the total synthesis of two out of the 28,672 possible isomers was completed^17^, but these did not match the natural product or reveal any further information on its structure.

Since the isolation and original acquisition of NMR spectroscopic data for caylobolide A in 2002, NMR spectroscopic and computational techniques and hardware have substantially improved^18,19^. For example, modern ultra-high-digital-resolution two-dimensional (2D) NMR spectroscopic techniques, often based on pure-shift pulse sequences which collapse proton multiplets into singlets, essentially eliminate signal overlap and increase signal intensity^20–22^. Nevertheless, even with such advancements, full stereochemical structure elucidation remains challenging, as highlighted by palstimolide A (Fig. 1a), whose 3D structure could not be determined in 2020^13^.

Here we describe the use of ultra-high-resolution NMR spectroscopy, Mosher’s ester analysis and synthesis to unveil the correct structure of caylobolide A (Fig. 1b). Our analysis has not only revised the position of the 1,3,5-triol motif but also assigned stereochemistry to all stereocentres, culminating in the first total synthesis of the correct structure of caylobolide A in 17 steps (longest linear sequence (LLS)), utilizing iterative stereocontrolled homologation of boronic esters to construct the 1,5-polyol backbone. This method provides a blueprint to tackle such seemingly intractable problems, which should enable the structure determination of some of the most complex molecules.

## Results and discussion

### Isolation

Caylobolide A was reisolated from a collection of marine cyanobacterium and was identified as the major compound in the extract by comparison of ^1^H and ^13^C NMR spectroscopic chemical shifts (DMSO-*d*_6_) and high-resolution mass spectrometry data with literature data^6^, yielding 40 mg (0.27% yield) of pure material (see Supplementary Information for full details of reisolation)

### NMR spectroscopic analysis

**Fig. 2:**
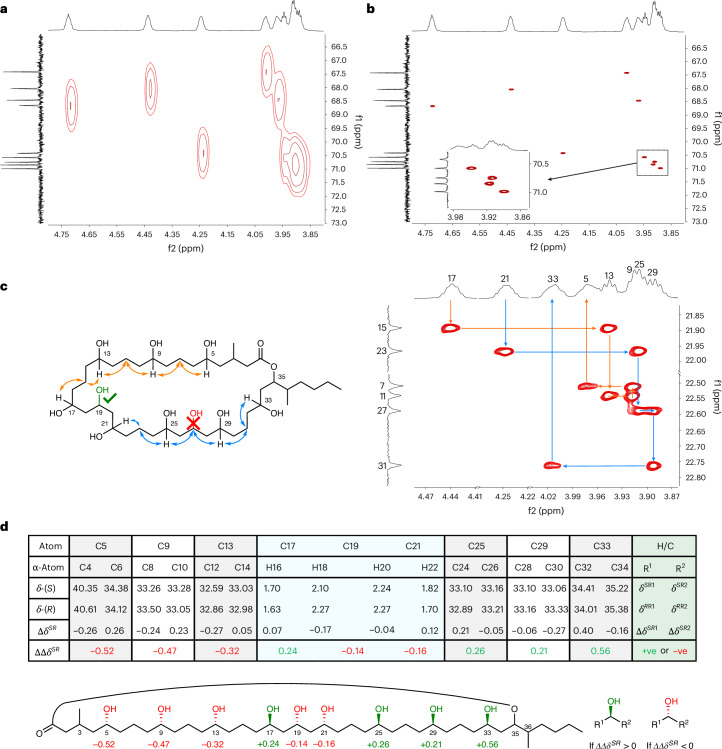
HSQC and HSQC-TOCSY spectra of caylobolide A at 700 MHz in pyridine-*d*_5_. **a**, HSQC spectra with 128 f1 increments over 31,645.6 Hz, giving a spectral resolution of ∼247 Hz. **b**, Pure-shift HSQC spectra with 1,024 f1 increments linear predicted to 3,072 and zero-filled to 4,096, over 6,443.3 Hz, giving a spectral resolution of ∼1.5 Hz. **c**, ^3^*J*_CH_ correlations observed by HSQC-TOCSY were used to ‘walk’ around the backbone of caylobolide A, leading to a regiochemical reassignment. The originally proposed structure suggested that the triol was at C25–C29 and has been repositioned to be at C17–C21. **d**, Tabulated chemical shifts (ppm) for the (*S*) and (*R*) Mosher esters of caylobolide A with the difference between Δ*δ*^*S*/*R*^ values giving ΔΔ*δ*^*SR*^, the sign of which directly correlates to the absolute stereochemistry of the stereogenic carbinol of interest; illustrated in the caylobolide A carbinol, with ΔΔ*δ*^*SR*^ values indicated at all six stereogenic secondary 1,5-alcohols. Atom numbers are highlighted in bold. Green ΔΔ*δ*^*SR*^ values depict stereochemistry as ‘up’ and red ΔΔ*δ*^*SR*^ values depict stereochemistry as ‘down’ in the orientation shown. It is worth noting that the ^13^C ΔΔ*δ*^*SR*^ values of the 1,3,5-triol did not align to the relative stereochemistry suggested by the Kishi analysis, ascribed to perturbation by the proximal stereocentres, and therefore analysis was performed using ^1^H chemical shift analysis with consideration of the pairwise additive effect of poly-Mosher esters (Supplementary Information, section 2.4.4)^23,24^.

Key to the NMR spectroscopic evaluation of caylobolide A, and analogous complex polyol structures, is the use of high spectroscopic resolution of the overlapping and convoluted NMR signals. To achieve this, we acquired data on caylobolide A using 2D NMR spectroscopic methods that combined: (1) ultra-high ^13^C digital resolution, that is, acquiring many data points per ppm; and (2) ^1^H ‘pure-shift’ methodology, which collapses broad ^1^H multiplets into sharp singlets. We also ran the spectra at relatively high magnetic fields to disperse the peaks, although it should be noted that this effect has a relatively small impact on the ability to resolve overlapped signals compared to increasing digital resolution and eliminating multiplicity. For the study reported here, heteronuclear single quantum coherence (HSQC) and heteronuclear single quantum coherence-total correlation spectroscopy (HSQC-TOCSY) spectra were acquired at 700 MHz, with ^13^C digital resolution of approximately 1.5 Hz by using spectral aliasing (12.5–49.1 ppm ^13^C spectrum range) and a very large number (4,096) of data points in that ^13^C dimension, combined with ^1^H pure-shifting of the HSQC spectra through bilinear rotation decoupling during the signal acquisition^21^. Under these conditions, all *CH*OH signals in the ^1^H range of 3.86–4.75 ppm became differentiable, as illustrated in the 4,096-data-point pure-shift HSQC (Fig. 2b), which does not occur in a comparable standard resolution (128 f1 data points) non-pure-shift HSQC (Fig. 2a) as used in the original report. A practical point of note is that the nine critical C*H*OH ^1^H NMR spectroscopic signals of caylobolide A overlapped with residual water present in DMSO-*d*_6_, but resolved in pyridine-*d*_5_.

Determination of the connectivity in caylobolide A was achieved by interpretation of a similarly ultra-high-resolution HSQC-TOCSY spectra with a 30 ms mixing time (Fig. 2c), but without the ^1^H pure shift because bilinear rotation decoupling is not compatible with hyphenated TOCSY NMR spectroscopy pulse sequences. Starting from lactone H-35, identified by HMBC correlation from the carbonyl group, HSQC-TOCSY signals for the stereogenic C*H*O protons showed clear ^3^*J*_CH_ correlations to β-*C*H_2_ carbons, first to H-33 and then sequentially to ‘walking’ along the 1,3-relationships around the backbone of caylobolide A (Fig. 2c). Notably, H-29 shows a ^3^*J*_CH_ correlation to C27 which has a chemical shift *δ* 22.6 ppm that is not compatible with the assignment of a carbinol centre, which was expected for C27 if the originally reported structure of caylobolide A was correct. Consequently, we concluded that C25–C29 is a 1,5-diol not a 1,3,5-triol as reported in the original structure. Further sequential assignment identified C21–C17 as the position of the 1,3,5-triol, which is then followed by a further triad of 1,5-diols from C17 to C5. Interestingly, this revised polyol pattern matches that of bastimolide A and B, suggesting a degree of commonality in their biosynthetic origin. The assignment of the NMR data for the macrocycle, in particular the identification of the α-*C*H_2_ groups, was then completed by comparison with a second HSQC-TOCSY spectrum that used a longer TOCSY mixing period of 60 ms, providing longer-range ^4^*J*_CH_ correlations. This comparison differentiates ^2^*J*_CH_ (present in both 30 ms and 60 ms mixing time spectra) and ^4^*J*_CH_ (present in only 60 ms mixing time spectra) correlations from the C*H*OH centres, enabling the sequential assignment of all α-*CH*_2_ signals working around the macrocycle starting from H-35 (Supplementary Fig. 2).

Determination of the absolute stereochemistry of the carbinol centres of caylobolide A was now possible through a Mosher’s ester analysis. Separate syntheses of the *R-* and *S-*nona-Mosher’s esters was followed by full assignment of the α-*C*H_2_^13^C chemical shifts in each reaction mixture using the same HSQC-TOCSY methodology described for the natural product above (pyridine-*d*_5_, 700 MHz; see Supplementary Information for full details). The sign of the difference in ^13^C chemical shifts for each pair of α-carbons, $\Delta\Delta\delta_{n}^{SR}\;(=\Delta\delta_{n-1}^{SR}-\Delta\delta_{n+1}^{SR})$, for each stereogenic carbinol centre allows explicit identification of the absolute stereochemistry (Fig. 2d). The relative stereochemistry of the 1,3,5-triol was determined by chemical shift comparison to Kishi’s ‘universal NMR database’^23,24^. Analysis of ^1^H $\Delta\delta_{n}^{SR}$ values confirmed the absolute stereochemistry (Supplementary Information, section 2.4.4) and matches that of bastimolide A, again implicating a common biosynthetic origin. In summary, this approach has enabled us to unambiguously assign all *δ*_C_ and *δ*_H_ (excluding OH groups) chemical shifts, revise the 2D structure and determine the absolute configuration of all nine carbinol stereocentres in caylobolide A, reducing the potential number of isomers of caylobolide from 28,672 to just eight, with only three unknown centres at C3, C35 and C36. To elucidate these centres, a synthetic approach was designed.

### Mixture-method total synthesis strategy

**Fig. 3:**
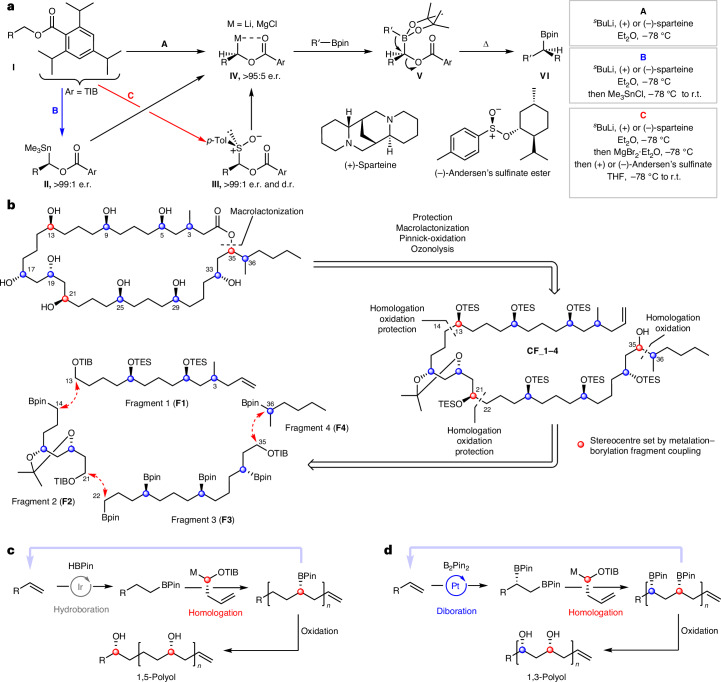
Metalation–borylation strategies utilized for the installation of stereocentres of caylobolide A. **a**, Different methods used for the generation of metalated carbenoids for application in metalation–borylation. Blue, generation of a tin-based carbenoid precursor; red, generation of an α-sulfinylbenzoate carbenoid precursor. The Δ above the top right arrow represents heat. **b**, Retrosynthetic analysis of caylobolide A. Following disconnection of the macrolactone, the complex polyol intermediate **CF_1-4** was further disconnected into four fragments, all unified by metalation–borylation strategies. **c**, Strategy to install 1,5-polyols through iterative hydroboration–homologation of an alkene followed by stereospecific oxidation. **d**, Strategy to install 1,3-polyols through iterative diboration–homologation of an alkene followed by stereospecific oxidation. *p*-Tol, *para*-tolyl; pin, pinacolato; TES, triethylsilyl; THF, tetrahydrofuran.

Our synthetic blueprint to elucidate the remaining stereogenic centres is based on using our stereocontrolled metalation–borylation methodology to both construct and then assemble the different fragments^25,26^. The method involves generation of an enantioenriched metalated benzoate ester **IV** through sparteine-controlled asymmetric deprotonation, tin–lithium exchange or sulfoxide–magnesium exchange of precursors **I**–**III** followed by reaction with a boronic ester to give a homologated boronic ester **VI** with high stereocontrol through 1,2-metalate rearrangement of boronate complex **V** (Fig. 3a)^27–29^. Figure 3b shows the retrosynthetic analysis of caylobolide A, with disconnection of the macrolactone to give polyol **CF_1-4** (CF = combined fragments) where the carboxylic acid is masked as a terminal alkene. Further disconnection of complex polyol **CF_1-4** led to a four-fragment convergent synthetic strategy, in which fragment unification can be achieved through our stereocontrolled metalation–borylation methodology^25^. Such a fragment coupling, followed by boron oxidation, would not only assemble the polyol chain, but would also generate the secondary alcohols at C13, C21 and C35 with the desired stereochemistry. The 1,5-polyol moieties of fragments 1 (**F1**) and 3 (**F3**) could be constructed by an iterative homologation/hydroboration sequence with enantiopure carbenoid building blocks derived from α-sulfinylbenzoates (Fig. 3c)^14,28^. The unknown stereochemistry at methyl-bearing C3 could be introduced in fragment 1 from citronellol, for which both enantiomers are readily available from the chiral pool. Replacing hydroboration with diboration would lead to the formation of the 1,3-diol moiety (Fig. 3d) in fragment 2 (**F2**)^30^. Finally, both enantiomers of fragment 4 (**F4**) could be constructed through our boronic ester homologation of *n*-butylboronic acid pinacol ester. We envisaged using our mixture encoded synthetic approach to solve the stereochemistry at C35 and C36, just as we had done in our structural reassignment of the polyketide baulamycin^31^. Using a known 2:1 mixture of isomers at C36 and using (+)-sparteine in the final coupling of fragment **F4** to control the C35 stereogenic centre would give a mixture of C36 diastereoisomers. Repeating this process but using (−)-sparteine would give another mixture of C36 diastereoisomers with the opposite stereochemistry at C35. Comparison of the two mixtures with the natural product would allow rapid identification of the correct isomer, with NMR spectroscopy integration providing the signature of the C36 stereochemistry.

### Fragment synthesis

**Fig. 4:**
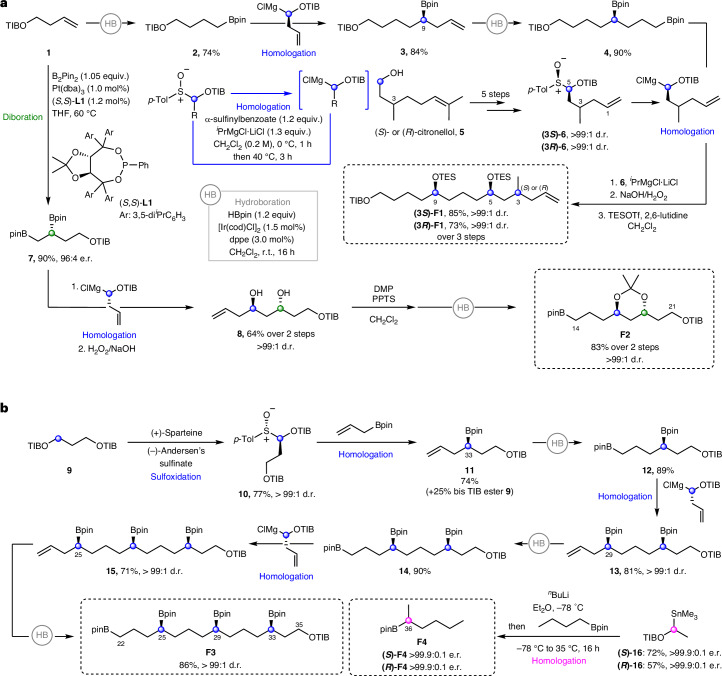
Synthesis of fragments F1, F2, F3 and F4. **a**, **F1** synthesis. Iterative hydroboration–homologation sequence, followed by boronic ester oxidation and TES protection. **F2** synthesis. Diboration of **1**, followed by homologation–oxidation, acetonide protection and hydroboration. **b**, **F3** synthesis. Iterative hydroboration–homologation sequence with enantioenriched sulfoxide building blocks to achieve enantiopure fragment **3** in seven steps from 9, installing three stereogenic centres. **F4** synthesis. Formation of an enantioenriched stannane followed by a homologation of *n*-butylboronic acid pinacol ester to afford fragment **4**. cod, 1,5-cyclooctadiene; dppe, ethylenebis(diphenylphosphine); Tf, trifluoromethanesulfonyl; DMP, 2,2-dimethoxypropane; dba, dibenzylideneacetone; PPTS, pyridinium *p*-toluene sulfonate.

Fragment 1 (**F1**) was synthesized from homoallylic TIB (2,4,6-triisopropylbenzoyl) ester **1** using a series of iterative hydroboration and homologations to initially give intermediate bis-boronic ester **4** (Fig. 4a). Separately, sulfoxides **(3*S*)-6** and **(3*R*)-6** were prepared in five steps starting from (*R*)- and (*S*)-citronellol **5**, respectively, and subsequently coupled with bis-boronic ester **4**. Following boronic ester oxidation and TES protection, (3*R*)- and (3*S*)-diastereomers of **F1** were obtained. Fragment 2 (**F2**) was synthesized in four steps from homoallylic TIB ester **1** using a sequence of diboration, homologation, oxidation, protection and hydroboration (Fig. 4a)^14^.

For fragment 3 (**F3**), 1,3-bis-TIB ester **9** was deprotonated with (+)-sparteine and trapping with (–)-Andersen’s sulfinate gave sulfoxide **10** (Fig. 4b)^27^. Stereocontrolled homologation–hydroboration sequences then installed the 1,5-polyboronic esters in an iterative fashion to yield tetraboronic ester **F3** as a single diastereoisomer and enantiomer in 21% overall yield across eight steps. Both enantiomers of fragment 4 (**F4**) were readily synthesized by lithiation–borylation using enantiopure stannanes **(*S*)-16** and **(*R*)-16** and *n*-butylboronic acid pinacol ester (Fig. 4b).

As we have demonstrated previously^28,30^, coupling of essentially enantiopure building blocks (stannanes/sulfoxides) with boronic esters is stereospecific and occurs with >99:1 e.r./d.r., and the same levels of stereocontrol can be expected in the current examples.

### Fragment coupling (F1, F2 and F3)

**Fig. 5:**
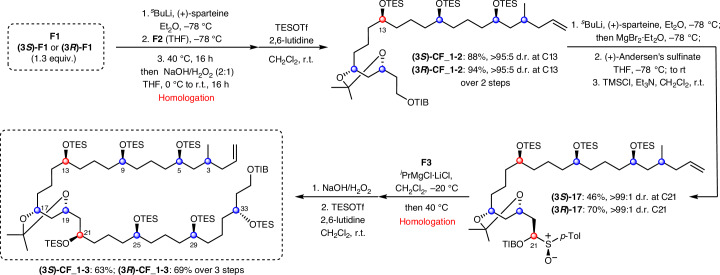
Coupling fragments F1, F2 and F3. Lithiation–borylation with (+)-sparteine to unify **F1** and **F2**. Subsequent coupling to **F3** was achieved by sulfoxide homologation. TMS, trimethylsilyl.

The C3-(*R*) and C3-(*S*) isomers of **F1** were coupled, separately, to **F2** via lithiation–borylation, and subsequent oxidation of the resulting boronic ester and TES protection gave the coupled products **(3*S*)-CF_1-2** and **(3*R*)-CF_1-2** in >95:5 d.r. (Fig. 5). Attempts to directly couple TIB ester **CF_1-2** with **F3** through the same lithiation–borylation process unfortunately suffered from poor yields (<40%). Instead, **CF_1-2** was deprotonated at C21 with (+)-sparteine, and trapped with (+)-Andersen’s sulfinate to give sulfoxide intermediate **17**, which was subsequently treated with ^*i*^PrMgCl·LiCl in the presence of **F3**^27^. This sequence gave the fragment-coupled product in a much improved 70% yield. The high selectivity for reaction of the primary boronic ester over secondary boronic esters is especially noteworthy because it enables the secondary boronic esters of **F3** to be carried through, acting as masked alcohols, without the need for additional oxidation and protection steps. The unified fragment was then oxidized and protected to give **(3*R*)-CF_1-3** in 69% yield over three steps.

### Mixture-method total synthesis

**Fig. 6:**
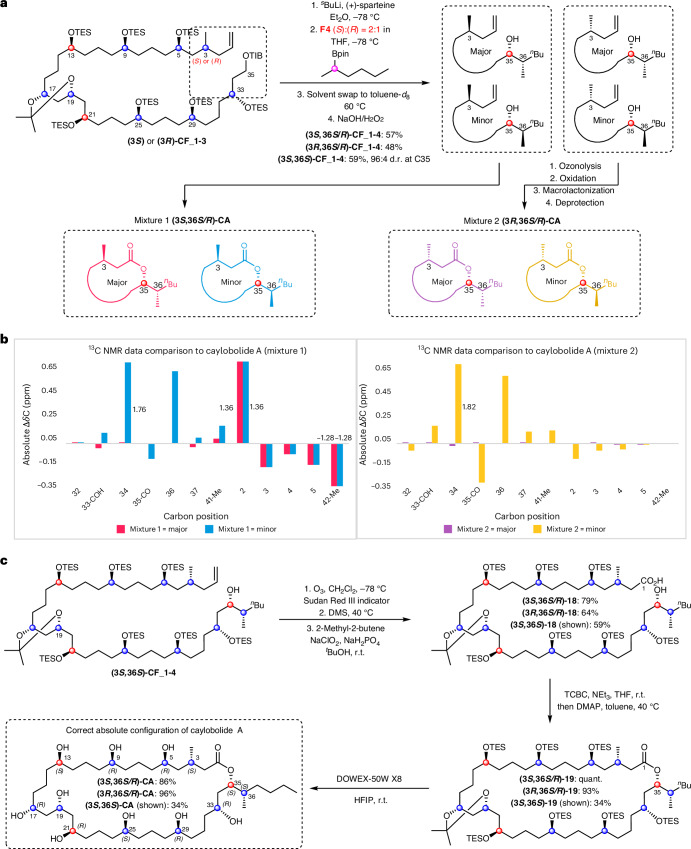
Key synthetic steps of the mixture study of caylobolide A. **a**, The forward synthetic procedure to introduce the mixtures by lithiation–borylation of either C3-(*S*) or C3-(*R*) **CF_1-3** with a 2:1 *S*:*R* mixture of enantiomers of **F4** followed by immediate peroxidic oxidation to the corresponding secondary alcohols **CF_1-4**. **b**, Comparison of diastereomeric mixture ^13^C NMR chemical shifts to caylobolide A. Chemical shifts were identified through analysis of ultra-high-resolution pure-shift HSQC spectra. Only relevant chemical shift differences are shown, and signals which were further away from stereochemical changes became degenerate. **c**, Synthesis of caylobolide A diastereomeric mixtures: (1) ozonolysis, (2) ozonide reduction, (3) Pinnick oxidation, Yamaguchi esterification and final global deprotection. HFIP, hexafluoroisopropanol; DMS, dimethylsulfide; DMAP, 4-(dimethylamino)pyridine; TCBC, 2,4,6-trichlorobenzoyl chloride.

With the C3-(*R*) and C3-(*S*) diastereomers of **CF_1-3** in hand, final lithiation–borylation with fragment **F4** can install the remaining two stereocentres at C35 and C36: the enantiomer of sparteine dictates the stereochemistry at C35, and the enantiomer of fragment **F4** dictates C36. Because it was found that caylobolide A is closely structurally related to bastimolide A, we hypothesized that the configuration of the lactone carbon (C35) would probably be the same, and so synthetic efforts towards the C35-(*S*) diastereomers were first targeted by using (+)-sparteine for lithiation of **CF_1-3**. As described above, we used our encoded-mixture method to determine the stereochemistry of C36. All four diastereomers at C3 and C36 were synthesized by separately reacting each diastereomer of **C2** with a mixture of C36 enantiomers of fragment **F4** in a 2:1 *S*:*R* ratio (Fig. 6a). Subsequent ozonolysis, Pinnick oxidation, macrolactonization and global deprotection of the two reaction mixtures yielded **Mixture 1** with C3-(*R*) and **Mixture 2** with C3-(*S*), each with a 2:1 *S*:*R* diastereomeric mixture at C36.

### Diastereomeric mixture analysis and total synthesis

^13^C chemical shifts of both diastereomeric mixtures were compared to the shifts of the naturally sourced caylobolide A sample (Fig. 6b). NMR spectroscopic data unambiguously revealed that neither diastereoisomer in **Mixture 1** matched the natural product, but the major diastereomer in **Mixture 2** did: the (3*S*)-, (35*S*)- and (36*S*)-diastereomers matched the natural product. On this basis, the total synthesis of the diastereopure natural product was undertaken by coupling **(3*S*)-CF_1-3** with **(36*S*)-F4** through lithiation–borylation with (+)-sparteine, and oxidation to yield the desired secondary alcohol **(3*S*,36*S*)-CF_1-4** (Fig. 6c). Macrolactonization and global deprotection then gave caylobolide A, the data of which fully matched that of the natural product.

## Conclusion

The structure, stereochemistry and total synthesis of caylobolide A, an archetypal member of a class of complex polyhydroxylated macrocyclic lactone natural products, has been achieved through the synergy of ultra-high-resolution NMR spectroscopic techniques, Mosher’s ester analysis and stereocontrolled synthesis. An unprecedented nona-Mosher’s ester analysis was performed on the macrolactone, leveraging ultra-high-resolution 2D NMR spectroscopic methods to reduce the number of potential isomers from 28,672 to eight. By analogy to bastimolide A, the stereochemistry of the lactone C–O centre was tentatively assigned, reducing this number to four. The remaining unknown stereocentre was determined through the highly convergent fragment-based total synthesis with late-stage, stereodivergent, lithiation–borylation fragment couplings to encode a mixture of diastereomers of known composition that could be compared to the natural product with ^13^C NMR spectroscopy. The total synthesis of caylobolide A was then achieved, using boronic ester homologation, in just 17 steps (longest linear sequence) from citronellol, providing a highly efficient route to this class of natural products with complete stereocontrol. This blueprint for structure determination and synthesis should enable the structure of other complex natural products, particularly those bearing repeating distal stereocentres, to be solved.

## Methods

Iterative homologation–hydroboration sequences were achieved using the following procedures.

### Homologation of boronic esters with α-sulfinylbenzoates as carbenoid precursors

^*i*^PrMgCl·LiCl (1.14 M in THF, 1.20 equiv.) was added dropwise to a mixture of boronic ester (1.00 equiv.) and sulfoxide (1.10 equiv.) in CH_2_Cl_2_ (0.20 M with respect to boronic ester) at 0 °C and the resulting solution was stirred for 1 h at the same temperature (pale yellow solution). After warming to room temperature, the reaction mixture was heated at 40 °C for 3 h (turbid white solution). The reaction mixture was then cooled to room temperature and quenched with saturated aqueous NH_4_Cl. The aqueous phase was extracted with diethyl ether (3×). The combined organics were dried over anhydrous Na_2_SO_4_ and filtered over a short pad of silica deactivated with diethyl ether:triethylamine 1% in order to remove the residual TIB acid.

The C–B stereochemistry of the product is controlled by the stereochemistry in the α position to sulfur of the sulfoxide starting material.

### Hydroboration of terminal alkenes

Following a literature procedure, pinacolborane^32^ (1.20 equiv. with respect to alkene) and alkene (1.00 equiv.) were added successively to a solution of [Ir(cod)Cl]_2_ (0.015 equiv.) and 1,2-bis(diphenylphosphino)ethane dppe (0.030 equiv.) in CH_2_Cl_2_ (0.30 M) at room temperature. The reaction mixture was then stirred for 16 h at room temperature. The reaction was quenched with methanol and extracted with diethyl ether (3×). The organics were dried over MgSO_4_ and evaporated under reduced pressure. The crude residue was purified by column chromatography to afford pure product.

All methods, including α-sulfinylbenzoate preparation, are fully described in the Supplementary Information with associated data supporting this research.

## Supplementary information

Supplementary Information Supplementary Figs. 1–27, Discussion and Tables 1–6.

## Data Availability

The data supporting this research are available within the Article and its Supplementary Information. NMR FID files and their associated processing parameters are available via figshare at 10.6084/m9.figshare.28173632 (ref. ^33^).
