# Supplementary material for: The combination of synthesis and ultra-high-resolution NMR spectroscopy reveals the correct structure of caylobolide A
Source: Nat Synth. 2025 Mar 13;4(7):859–68. doi: 10.1038/s44160-025-00762-2 (PMC12254034; doi:10.1038/s44160-025-00762-2)
Supplement: Supplementary file 1 — Supplementary Figs. 1–27, Discussion and Tables 1–6. [file 44160_2025_762_MOESM1_ESM.pdf]

nature synthesis

Supplementary information https://doi.org/10.1038/s44160-025-00762-2

The combination of synthesis and ultra-high-resolution NMR spectroscopy reveals the correct structure of caylobolide A

In the format provided by the authors and unedited

Table of Contents

List of Supplementary Figures and Tables 2

List of Characterised Products 4

1 GENERAL INFORMATION 7

2 EXPERIMENTAL DATA 9

2.1 Isolation of Caylobolide A 9

2.1.1 Fractionation details 9

2.2 Caylobolide A NMR analysis (DMSO-*d*6) 11

2.2.1 ^{1}H NMR (101 MHz, DMSO-*d*6) of 0024i5F 11

2.2.2 HSQC (DMSO-*d*6) of 0024i5F 11

2.2.3 COSY (DMSO-*d*6) of 0024i5F 12

2.2.4 HMBC (DMSO-*d*6) of 0024i5F 12

2.2.5 HSQC-TOCSY of 0024i5F 12

2.3 Skeletal reassignment by NMR (pyridine-*d*5) 14

2.3.1 Ultra-high-resolution HSQC-TOCSY 14

2.3.2 Ultra-high-resolution pureshift HSQC 17

2.3.3 Other 2D spectra 19

2.3.4 1D spectra of caylobolide A 21

2.4 Stereochemical reassignment by NMR 23

2.4.1 Kishi's 1,3-diol analysis (universal NMR database) 23

2.4.2 Mosher ester analysis 23

2.4.3 Mosher ester NMR spectra 24

2.4.4 Simplified Mosher ester analysis 27

3 GENERAL PROCEDURES 29

General procedure 1 (GP1) – α-sulfinylbenzoate preparation 29

General procedure 2 (GP2) – Sulfoxide homologation 30

General procedure 3 (GP3) – Hydroboration 31

General procedure 4 (GP4) – Boronic ester oxidation 31

General procedure 5 (GP5) – TES protection of alcohols 32

4 PREPARATION AND CHARACTERISATION DATA 32

4.1 Fragment 1 34

4.2 Fragment 2 47

4.3 Fragment 3 52

4.4 Fragment 4 61
4.5 Caylobolide A (3*R*, 36*S*/*R*) fragment couplings 66
4.6 Caylobolide A (3*R*, 36*S*/*R*) endgame 76
4.7 Caylobolide A (3*S*, 36*S*/*R*) fragment couplings 82
4.8 Caylobolide A (3*S*, 36*S*/*R*) endgame 91
4.9 Stereocontrolled caylobolide A (3*S*, 36*S*) endgame 95
4.9.1 NMR comparison table 101
5 REFERENCES 102
6 SPECTRAL APPENDIX 103
^{1}H NMR comparison of synthetic vs naturally occurring caylobolide A 158
^{13}C NMR comparison of synthetic vs naturally occurring caylobolide A 161

List of Supplementary Figures and Tables

Figure S1: Fractionation details for the purification of caylobolide A (fraction 0024i5F) 10

Table S1: 1H-NMR Comparison of 0024i5F and caylobolide A in DMSO-*d*6. 14

Figure S2: Overlaid HSQC-TOCSY spectra of caylobolide A in pyridine-*d*5 with TOCSY mixing times of 30 ms (green) and 60 ms (red) used to identify and differentiate between α-carbon atoms. 15

Figure S3: Ultra-high-resolution HSQC-TOCSY of caylobolide A in pyridine-*d*5 (Phase-sensitive ge-2D HSQC-TOCSY with DIPSI-2 using PEP and adiabatic inversion and refocusing pulses: hsqcdietgpsisp.2) with a 30 ms mixing time. Spectrometer frequency (949.57, 238.77), acquired size (1024, 2700), spectral size (4096, 8192), spectral width (9469.7, 8116.9), 24 scans. Spectrum centred at the aliphatic region. 15

Figure S3: Ultra-high-resolution HSQC-TOCSY of caylobolide A in pyridine-*d*5 (Phase-sensitive ge-2D HSQC-TOCSY with DIPSI-2 using PEP and adiabatic inversion and refocusing pulses: hsqcdietgpsisp.2) with a 60 ms mixing time. Spectrometer frequency (700.19, 176.07) acquired size (755, 1024), spectral size (2048, 8192), spectral width (6302.5, 6443.3), 4 scans. Spectrum centred at the aliphatic region. 16

Figure S4: Ultra-high-resolution pureshift HSQC of caylobolide A in pyridine-*d*5 (Phase-sensitive ge-2D multiplicity-edited HSQC using echo-antiecho and inversion and matched sweep adiabatic pulses with broadband homodecoupling: hsqcedetgpsp.3). Spectrometer frequency (949.57, 238.77), acquired size (1024, 512), spectral size (4096, 2048), spectral width (9469.7, 8116.9), 32 scans. Spectrum centred at the aliphatic region where *CH*O correlations are folded back into the lower ppm region (correlations between 3-6 ppm in ^{1}H). 17

Table S2: 1H-NMR of 0024i5F (caylobolide A) in pyridine-*d5*. ^{13}C chemical shifts referenced to pyridine (123.87 ppm) and ^{1}H NMR referenced to pyridine (7.22 ppm). H_2O signal appears at 5.15 ppm. 22

Figure S12: Analysis of caylobolide A 1,3,5-triol (C17-C21) by Kishi's universal NMR database. ^{13}C Chemical shifts of >70 ppm or <70 ppm indicates a syn or anti 1,3-diol relationship respectively. 23

Figure S13: Ultra-high-resolution HSQC of caylobolide A (S)-nona Mosher ester (pyridine-d5) zoomed in to the region containing CHO correlations with atom numbers assigned to each correlation. 24

Figure S14: Ultra-high-resolution HSQC-TOCSY (30 ms mixing time) of caylobolide A (S)-nona Mosher ester (pyridine-d5) zoomed into the region displaying $^2J_{CH}$ correlations used for the determination of α-CH_2 chemical shifts. Atom numbers assigned to ^{13}C chemical shifts for each correlation. 25

Figure S15: Ultra-high-resolution HSQC of caylobolide A (R)-nona Mosher ester (pyridine-d5) zoomed in to the region containing CHO correlations with atom numbers assigned to each correlation. 26

Figure S16: Ultra-high-resolution HSQC-TOCSY (30 ms mixing time) of caylobolide A (R)-nona Mosher ester (pyridine-d5) zoomed into the region displaying $^2J_{CH}$ correlations used for the determination of α-CH_2 chemical shifts. Atom numbers assigned to ^{13}C chemical shifts for each correlation. 27

Table S3: Mosher ester analysis ^{13}C values for caylobolide A 1,5-polyol region. 27

Figure S17: Predicted pairwise additive effects in the Mosher ester analysis of the anti-syn 1,3,5-triol (C17-C21) portion of caylobolide A. **–** $\Delta\delta^{SR} < 0$; **+** $\Delta\delta^{SR} > 0$; **|** (equivocal) $\Delta\delta^{SR} \approx 0$. 28

Table S4: Mosher ester analysis ^1H values for caylobolide A 1,3-polyol region. 28

Figure S18: **a)** Predicted $\Delta\Delta\delta^{SR}$ pairwise additive effects in the Mosher ester analysis of the anti-syn 1,3,5-triol (C17-C21) portion of caylobolide A. $\Delta\Delta\delta^{SR} = \Delta\delta^{SR}_{(n-1)} - \Delta\delta^{SR}_{(n+1)}$. – $\Delta\delta^{SR} < 0$; + $\Delta\delta^{SR} > 0$; | (equivocal) $\Delta\delta^{SR} \approx 0$. **b)** The absolute stereochemistry of all hydroxylated stereocentres of caylobolide A, reoriented for simplicity of analysis. 29

Table S5: Data used for the comparison of diastereomeric mixtures 1 and 2 to naturally occurring caylobolide A for the determination of stereochemistry at C3 and C36. The major component of Mixture 2 (3S, 36S)-CA matches caylobolide A, with a mean absolute error of 0.0075 ppm for the atoms investigated, and a maximum difference of 0.02 ppm. 94

Table S6: ^{13}C NMR comparison of caylobolide A to synthetic caylobolide A. Spectra recorded in pyridine-d5, referenced to 123.87 ppm 101

Figure S20: Full ^1H NMR spectral comparison of synthetic and naturally occurring caylobolide A in pyridine-d5 (700 MHz). 158

Figure S21: ^1H NMR spectral comparison of the CHOH region of synthetic and naturally occurring caylobolide A in pyridine-d5 (700 MHz). 159

Figure S22: ^1H NMR spectral comparison of CH_2-2 and CH-3 of synthetic and naturally occurring caylobolide A in pyridine-d5 (700 MHz). 159

Figure S23: ^1H NMR spectral comparison of the alkyl region (0.7-2.2 ppm) of synthetic and naturally occurring caylobolide A in pyridine-d5 (700 MHz). 160

Figure S24: Full ^{13}C NMR spectral comparison of synthetic and naturally occurring caylobolide A in pyridine-d5 (151 Hz synthetic, 176 MHz natural). 161

Figure S25: ^{13}C NMR spectral comparison of the CHO region (67-76 ppm) of synthetic and naturally occurring caylobolide A in pyridine-d5 (151 Hz synthetic, 176 MHz natural). 162

Figure S26: ^{13}C NMR spectral comparison of the α-CH_2 methylene region (36-46 ppm) of synthetic and naturally occurring caylobolide A in pyridine-d5 (151 Hz synthetic, 176 MHz natural). 162

Figure S27: ^{13}C NMR spectral comparison of the β-CH_2 methylene, methyl and C-3 region (12-34 ppm) of synthetic and naturally occurring caylobolide A in pyridine-d5 (151 Hz synthetic, 176 MHz natural). 163

List of Characterised Products

Caylobolide A nona-Mosher ester derivatisation 23

But-3-en-1-yl 2,4,6-triisopropylbenzoate (1) 32

(1*R*)-1-(*p*-Tolylsulfinyl)but-3-en-1-yl 2,4,6-triisopropylbenzoate (S1-*anti*) & (1*S*)-1-(*p*-tolylsulfinyl)but-3-en-1-yl 2,4,6-triisopropylbenzoate (S1-*syn*) 33

(1*S*)-1-(*p*-Tolylsulfinyl)but-3-en-1-yl 2,4,6-triisopropylbenzoate (S1-*syn*) 34

(*S*)-3,7-Dimethyloct-6-en-1-yl 2,4,6-triisopropylbenzoate ((3*S*)-S2) 34

(*S*)-6-Hydroxy-3-methylhexyl 2,4,6-triisopropylbenzoate ((3*S*)-S3) 35

(*S*)-6-Iodo-3-methylhexyl 2,4,6-triisopropylbenzoate ((3*S*)-S4) 36

(*S*)-3-Methylhex-5-en-1-yl 2,4,6-triisopropylbenzoate ((3*S*)-S5) 37

(1R,3S)-3-Methyl-1-(p-tolylsulfinyl)hex-5-en-1-yl 2,4,6-triisopropylbenzoate ((3*S*)-6) 38

(*R*)-3,7-Dimethyloct-6-en-1-yl 2,4,6-triisopropylbenzoate ((3*R*)-S2) 39

(*R*)-6-Hydroxy-3-methylhexyl 2,4,6-triisopropylbenzoate ((3*R*)-S3) 39

(*R*)-6-Iodo-3-methylhexyl 2,4,6-triisopropylbenzoate ((3*R*)-S4) 40

(*R*)-3-Methylhex-5-en-1-yl 2,4,6-triisopropylbenzoate ((3*R*)-S5) 40

(1R,3R)-3-methyl-1-(p-tolylsulfinyl)hex-5-en-1-yl 2,4,6-triisopropylbenzoate ((3*R*)-6) 41

4-(4,4,5,5-Tetramethyl-1,3,2-dioxaborolan-2-yl)butyl 2,4,6-triisopropylbenzoate (2) 42

(*R*)-5-(4,4,5,5-Tetramethyl-1,3,2-dioxaborolan-2-yl)oct-7-en-1-yl 2,4,6-triisopropylbenzoate (3) 42

(*S*)-5,8-bis(4,4,5,5-Tetramethyl-1,3,2-dioxaborolan-2-yl)octyl 2,4,6-triisopropylbenzoate (4) 43

(5R,9R,11S)-11-methyl-5,9-bis((triethylsilyl)oxy)tetradec-13-en-1-yl 2,4,6-triisopropylbenzoate ((3*S*)-F1) 44

(5R,9R,11R)-11-methyl-5,9-bis((triethylsilyl)oxy)tetradec-13-en-1-yl 2,4,6-triisopropylbenzoate (3*R*-F1) 46

(*S*)-3,4-Bis(4,4,5,5-tetramethyl-1,3,2-dioxaborolan-2-yl)butyl 2,4,6-triisopropylbenzoate (7) 47

(3*R*,5*R*)-3,5-Dihydroxyoct-7-en-1-yl 2,4,6-triisopropylbenzoate (8) 49

2-((4*R*,6*R*)-6-Allyl-2,2-dimethyl-1,3-dioxan-4-yl)ethyl 2,4,6-triisopropylbenzoate (S9) 50

2-((4*R*,6*R*)-2,2-Dimethyl-6-(3-(4,4,5,5-tetramethyl-1,3,2-dioxaborolan-2-yl)propyl)-1,3-dioxan-4-yl)ethyl 2,4,6-triisopropylbenzoate (F2) 51

Propane-1,3-diyl bis(2,4,6-triisopropylbenzoate) (9) 52

(1R)-1-(p-tolylsulfinyl)propane-1,3-diyl bis(2,4,6-triisopropylbenzoate) (10-*anti*) 53

(*R*)-3-(4,4,5,5-Tetramethyl-1,3,2-dioxaborolan-2-yl)hex-5-en-1-yl 2,4,6-triisopropylbenzoate (11) 55

(*R*)-3,6-Bis(4,4,5,5-tetramethyl-1,3,2-dioxaborolan-2-yl)hexyl 2,4,6-triisopropylbenzoate (12) 56

(3*R*,7*S*)-3,7-Bis(4,4,5,5-tetramethyl-1,3,2-dioxaborolan-2-yl)dec-9-en-1-yl 2,4,6-triisopropylbenzoate (13) 57

(3*R*,7*R*)-3,7,10-Tris(4,4,5,5-tetramethyl-1,3,2-dioxaborolan-2-yl)decyl 2,4,6-triisopropylbenzoate (14) 58

(3*R*,7*R*,11*S*)-3,7,11-Tris(4,4,5,5-tetramethyl-1,3,2-dioxaborolan-2-yl)tetradec-13-en-1-yl 2,4,6-triisopropylbenzoate (15) 59

(3*R*,7*R*,11*R*)-3,7,11,14-Tetrakis(4,4,5,5-tetramethyl-1,3,2-dioxaborolan-2-yl)tetradecyl 2,4,6-triisopropylbenzoate (F3) 60

Ethyl 2,4,6-triisopropylbenzoate (S10) 61

(*S*)-1-(Trimethylstannyl)ethyl 2,4,6-triisopropylbenzoate ((*S*)-16) 62

(*R*)-2-(Hexan-2-yl)-4,4,5,5-tetramethyl-1,3,2-dioxaborolane ((*R*)-F4) 63

(*R*)-1-(Trimethylstannyl)ethyl 2,4,6-triisopropylbenzoate ((*R*)-16) 64

(*S*)-2-(Hexan-2-yl)-4,4,5,5-tetramethyl-1,3,2-dioxaborolane ((*S*)-F4) 65

2-((6S)-6-((4R,8R,12R,14S)-4-hydroxy-14-methyl-8,12-bis((triethylsilyl)oxy)heptadec-16-en-1-yl)-2,2-dimethyl-1,3-dioxan-4-yl)ethyl 2,4,6-triisopropylbenzoate ((3*S*)-S12) 66

2-((6S)-2,2-dimethyl-6-((4R,8R,12R,14S)-14-methyl-4,8,12-tris((triethylsilyl)oxy)heptadec-16-en-1-yl)-1,3-dioxan-4-yl)ethyl 2,4,6-triisopropylbenzoate ((3S)-CF_1-2) 68

(1R)-2-((6S)-2,2-dimethyl-6-((4R,8R,12R,14S)-14-methyl-4,8,12-tris((triethylsilyl)oxy)heptadec-16-en-1-yl)-1,3-dioxan-4-yl)-1-(p-tolylsulfinyl)ethyl 2,4,6-triisopropylbenzoate ((3*S*)-17) 69

(3R,7R,11S,15R)-16-((4R,6S)-2,2-dimethyl-6-((4R,8R,12R,14S)-14-methyl-4,8,12-tris((triethylsilyl)oxy)heptadec-16-en-1-yl)-1,3-dioxan-4-yl)-3,7,11,15-tetrakis(4,4,5,5-tetramethyl-1,3,2-dioxaborolan-2-yl)hexadecyl 2,4,6-triisopropylbenzoate ((3*S*)-S13) 71

(3R,7R,11S,15R)-16-((4S,6S)-2,2-dimethyl-6-((4R,8R,12R,14S)-14-methyl-4,8,12-tris((triethylsilyl)oxy)heptadec-16-en-1-yl)-1,3-dioxan-4-yl)-3,7,11,15-tetrakis((triethylsilyl)oxy)hexadecyl 2,4,6-triisopropylbenzoate ((3*S*)-CF_1-3) .. 73

(6S,8R,12R,16S,20R)-21-((4S,6S)-2,2-dimethyl-6-((4R,8R,12R,14S)-14-methyl-4,8,12-tris((triethylsilyl)oxy)heptadec-16-en-1-yl)-1,3-dioxan-4-yl)-5-methyl-8,12,16,20-tetrakis((triethylsilyl)oxy)henicosan-6-ol ((3*S*, 36*S*/*R*)-CF_1-4) 75

(3R,5R,9R,13R)-16-((4S,6S)-6-((2R,6S,10R,14R,16S)-16-hydroxy-17-methyl-2,6,10,14-tetrakis((triethylsilyl)oxy)henicosyl)-2,2-dimethyl-1,3-dioxan-4-yl)-3-methyl-5,9,13-tris((triethylsilyl)oxy)hexadecanoic acid ((3*R*, 36*S*/*R*)-18) 76

(1S,5R,9R,13R,15R,19S,21R,25R,29S,33R,35S)-19-(hexan-2-yl)-15,37,37-trimethyl-5,9,13,21,25,29,33-heptakis((triethylsilyl)oxy)-18,36,38-trioxabicyclo[33.3.1]nonatriacontan-17-one ((3*R*, 36*S*/*R*)-19)................................ 78

(4R,6R,10R,14S,18R,22R,26S,30R,34R,36S)-36-(hexan-2-yl)-6,10,14,18,20,22,26,30,34-nonahydroxy-4-methyloxacyclohexatriacontan-2-one ((3*R*, 36*S*/*R*)-CA).. 79

2-((6S)-6-((4R,8R,12R,14R)-4-hydroxy-14-methyl-8,12-bis((triethylsilyl)oxy)heptadec-16-en-1-yl)-2,2-dimethyl-1,3-dioxan-4-yl)ethyl 2,4,6-triisopropylbenzoate ((3*R*)-S12) 82

2-((6S)-2,2-dimethyl-6-((4R,8R,12R,14R)-14-methyl-4,8,12-tris((triethylsilyl)oxy)heptadec-16-en-1-yl)-1,3-dioxan-4-yl)ethyl 2,4,6-triisopropylbenzoate ((3*R*)-CF_1-2) 83

(1R)-2-((6S)-2,2-dimethyl-6-((4R,8R,12R,14R)-14-methyl-4,8,12-tris((triethylsilyl)oxy)heptadec-16-en-1-yl)-1,3-dioxan-4-yl)-1-(p-tolylsulfinyl)ethyl 2,4,6-triisopropylbenzoate ((3*R*)-17).. 85

(3R,7R,11S,15R)-16-((4R,6S)-2,2-dimethyl-6-((4R,8R,12R,14R)-14-methyl-4,8,12-tris((triethylsilyl)oxy)heptadec-16-en-1-yl)-1,3-dioxan-4-yl)-3,7,11,15-tetrakis(4,4,5,5-tetramethyl-1,3,2-dioxaborolan-2-yl)hexadecyl 2,4,6-triisopropylbenzoate ((3*R*)-S13) 86

(3R,7R,11S,15R)-16-((4S,6S)-2,2-dimethyl-6-((4R,8R,12R,14R)-14-methyl-4,8,12-tris((triethylsilyl)oxy)heptadec-16-en-1-yl)-1,3-dioxan-4-yl)-3,7,11,15-tetrakis((triethylsilyl)oxy)hexadecyl 2,4,6-triisopropylbenzoate ((3*R*)-CF_1-3) .. 88

(6S,8R,12R,16S,20R)-21-((4S,6S)-2,2-dimethyl-6-((4R,8R,12R,14R)-14-methyl-4,8,12-tris((triethylsilyl)oxy)heptadec-16-en-1-yl)-1,3-dioxan-4-yl)-5-methyl-8,12,16,20-tetrakis((triethylsilyl)oxy)henicosan-6-ol ((3*R*, 36*S*/*R*)-CF_1-4) 90

(3S,5R,9R,13R)-16-((4S,6S)-6-((2R,6S,10R,14R,16S)-16-hydroxy-17-methyl-2,6,10,14-tetrakis((triethylsilyl)oxy)henicosyl)-2,2-dimethyl-1,3-dioxan-4-yl)-3-methyl-5,9,13-tris((triethylsilyl)oxy)hexadecanoic acid ((3*S*, 36*S*/*R*)-18)............. 91

(1S,5R,9R,13R,15S,19S,21R,25R,29S,33R,35S)-19-(hexan-2-yl)-15,37,37-trimethyl-5,9,13,21,25,29,33-heptakis((triethylsilyl)oxy)-18,36,38-trioxabicyclo[33.3.1]nonatriacontan-17-one ((3*S*, 36*S*/*R*)-19)................................ 92

(4S,6R,10R,14S,18R,22R,26S,30R,34R,36S)-36-(hexan-2-yl)-6,10,14,18,20,22,26,30,34-nonahydroxy-4-methyloxacyclohexatriacontan-2-one (3*S*, 36*S*/*R*)-CA 93

(5S,6S,8R,12R,16S,20R)-21-((4S,6S)-2,2-dimethyl-6-((4R,8R,12R,14R)-14-methyl-4,8,12-tris((triethylsilyl)oxy)heptadec-16-en-1-yl)-1,3-dioxan-4-yl)-5-methyl-8,12,16,20-tetrakis((triethylsilyl)oxy)henicosan-6-ol ((3*R*, 36*S*)-CF_1-4) 95
(3S,5R,9R,13R)-16-((4S,6S)-6-((2R,6S,10R,14R,16S,17S)-16-hydroxy-17-methyl-2,6,10,14-tetrakis((triethylsilyl)oxy)henicosyl)-2,2-dimethyl-1,3-dioxan-4-yl)-3-methyl-5,9,13-tris((triethylsilyl)oxy)hexadecanoic acid ((3*S*, 36*S*)-18) 96
(1S,5R,9R,13R,15S,19S,21R,25R,29S,33R,35S)-19-((S)-hexan-2-yl)-15,37,37-trimethyl-5,9,13,21,25,29,33-heptakis((triethylsilyl)oxy)-18,36,38-trioxabicyclo[33.3.1]nonatriacontan-17-one (3*S*, 36*S*)-19 98
(4S,6R,10R,14S,18R,22R,26S,30R,34R,36S)-36-((S)-hexan-2-yl)-6,10,14,18,20,22,26,30,34-nonahydroxy-4-methyloxacyclohexatriacontan-2-one ((3*S*, 36*S*)-CA) 99

1 GENERAL INFORMATION

Solvents, Reagents, Glassware and Reaction Setup: unless otherwise stated, all reactions were conducted under an inert atmosphere of nitrogen in flame dried glassware using standard Schlenk techniques. Air- and moisture-sensitive liquids and solutions were transferred via syringe into the reaction vessels through a rubber septum. Unless otherwise specified, all reagents were purchased at highest commercial quality and used as received. Non-anhydrous solvents were purchased (unless specified) at the highest commercial quality and used as received. CH_2Cl_2, Et_2O and THF were dried on an Anhydrous Engineering alumina column drying system. Temperatures described below −10 °C were achieved using Thermo Scientific EK-90 or Huber TC100E cryostats or appropriate solvent/dry ice baths.

Chromatography: flash column chromatography was carried out using Sigma-Aldrich silica gel (60 Å, 230-400 mesh, 40-63 μm) or a Biotage Isolera One automated flash purification system, as indicated. Reactions were followed by thin-layer chromatography (TLC) where practical, using aluminium-backed Merck Kieselgel 60 F254 fluorescent treated silica gel plates, which were visualised under UV light or by staining with aqueous basic $KMnO_4$, acidic *p*-anisaldehyde solution in ethanol, or phosphomolybdic acid solution in ethanol.

NMR High-field NMR spectra were collected on either a 9.4 T Jeol ECZ spectrometer fitted with a 5 mm 40ROHFXS probe (400 MHz), 9.4 T Jeol ECS spectrometer fitted with a 5 mm 40RO5AT probe (400 MHz), 9.4 T Bruker Nano spectrometer fitted with a 5 mm BBFO probe (400 MHz), 11.75 T Bruker Avance III HD spectrometer fitted with a 5 mm DCH 500S1 cryoprobe (500 MHz), 14.1 T Bruker Neo spectrometer fitted with a 5 mm TXO

600S3 cryoprobe (600 MHz), 16.44 T Bruker AVANCE III HD spectrometer fitted with a 1.7-mm TCI triple resonance micro cryoprobe (700 MHz) or Oxford's 22.3 T Oxford Instruments spectrometer fitted with a 5 mm TCI cryoprobe (950 MHz). All NMR experiments were collected at 1 atm and 298 K according to the respective spectrometer console. Chemical shifts (δ) are quoted in parts per million (ppm) and referenced to the appropriate NMR solvent peak(s) and are assigned in accordance with numbered diagrams; with resonances described as s (singlets), d (doublets), t (triplets), q (quartets), p (pentets), combinations thereof (i.e. td indicates a triplet of doublets) or m (multiplets) and br. s (broad singlet). Numbering of C-atoms for NMR assignment follows the numbering given to Caylobolide A in the original paper.

HRMS (high resolution mass spectra) were recorded on a Bruker Daltonics MicrOTOF II by Electrospray Ionisation (ESI); a Thermo Scientific QExactive by Electron Ionisation (EI); a Thermo Scientific Orbitrap Elite by ESI or Atmospheric Pressure Chemical Ionisation (APCI); or a Bruker UltrafleXtreme by Matrix-assisted Laser Desorption/Ionisation (MALDI). Only molecular ions ($[M+H]^+$, $[M+NH_4]^+$ or $[M+Na]^+$) are reported.

HPLC: enantiomeric ratio was determined by HPLC analysis on chiral stationary phase performed using Daicel Chiralpak IA/IB columns (4.6 mm×250 mm×5 μm)/OD column (4.6 mm×250 mm×10 μm)/AD-H (4.6 mm×250 mm×5 μm) column on an Agilent system and monitored using a diode array detector (DAD).

IR spectra were recorded on neat compounds using a Perkin Elmer (Spectrum One) FT-IR spectrometer (ATR sampling accessory). Selected absorbances (ν_{max}, expressed in cm^{-1}) are reported.

Melting points (m.p.) were recorded in degrees Celsius (°C) using a Stuart SMP30 melting point apparatus.

Optical rotations ($[\alpha]_D^{25}$) were measured on a Bellingham & Stanley Ltd. ADP 220 polarimeter.

ECD were recorded on a JASCO J-815 CD spectrometer.

Naming of compounds: Compound names are generated by ChemDraw Professional 20.0 software (PerkinElmer), following the IUPAC nomenclature.

2 EXPERIMENTAL DATA

2.1 Isolation of Caylobolide A

2.1.1 Fractionation details

A 150 mL sample of cyanobacterium was collected in June 2015 at a depth of 5-10 feet off the coast of Cas Abao, Curacao, and given the collection code CUCA-060415-7. It was described as a red filamentous mat in the sand. The cyanobacterial sample was put in a mix of 1:1 sea water and ethanol immediately upon collection, and later stored in −20°C upon return from Curacao to Pittsburgh.

In September 2016, the 150 mL sample of the cyanobacterium was extracted using CH_2Cl_2:MeOH 2:1 for 30 minute soaks five times, and an additional soak was left overnight at room temperature. The aqueous phase was separated from the organic phase, which was dried and evaporated in vacuo to afford 662 mg of an oily crude extract, given the internal code DUQ0024. The extract was then subjected to flash liquid chromatography, using the gradient described in table 2A.

At the same time, fraction 0024i, eluting with 100% MeOH, was subjected to normal phase column chromatography, using a gradient of 0-100% MeOH in CH_2Cl_2, yielding seven fractions (0024i1-7). Fraction 0024i5, with the same peaks in the aromatic region of the ^{1}H-NMR as seen in 0024H6E4, was subjected to another normal phase column, using a gradient of 0-100% MeOH in EtOAc, with a slower gradient between 20-60% MeOH where 0024i5 eluted in the previous column, yielding seven fractions (0024i5A-G). The ^{1}H-NMR of fraction 0024i5F showed an ^{1}H-NMR similar to that seen for 0024H6E4, without some of the minor peaks including those seen in the aromatic region. Fraction 0024i5F was a known compound caylobolide A

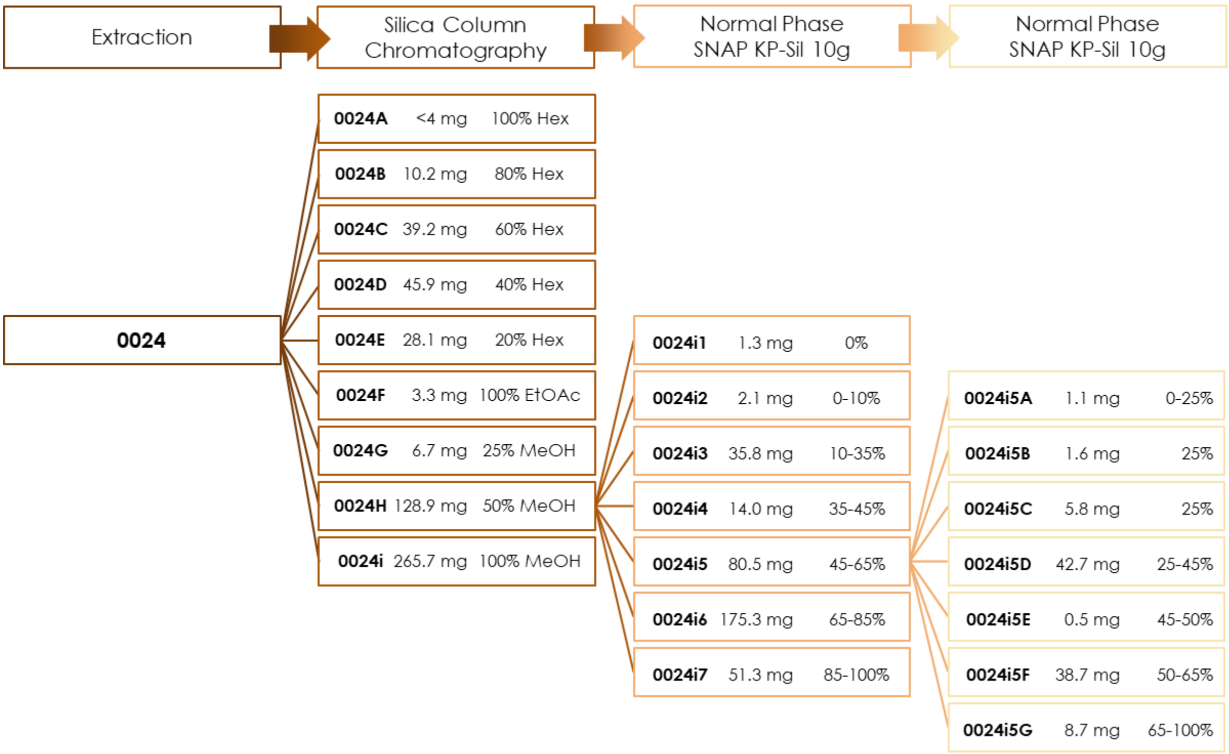

Figure S1: Fractionation details for the purification of caylobolide A (fraction 0024i5F)

0024i5F ESI+.

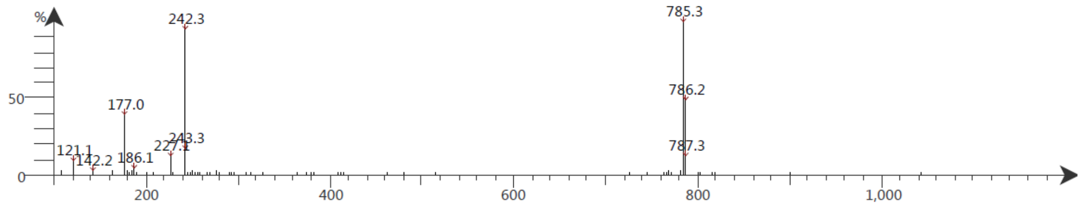

0024i5F ESI-

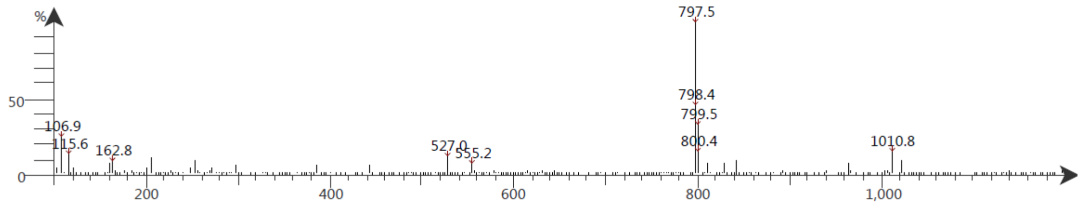

2.2 Caylobolide A NMR analysis (DMSO-*d*6)

2.2.1 ^{1}H NMR (101 MHz, DMSO-*d*6) of 0024i5F

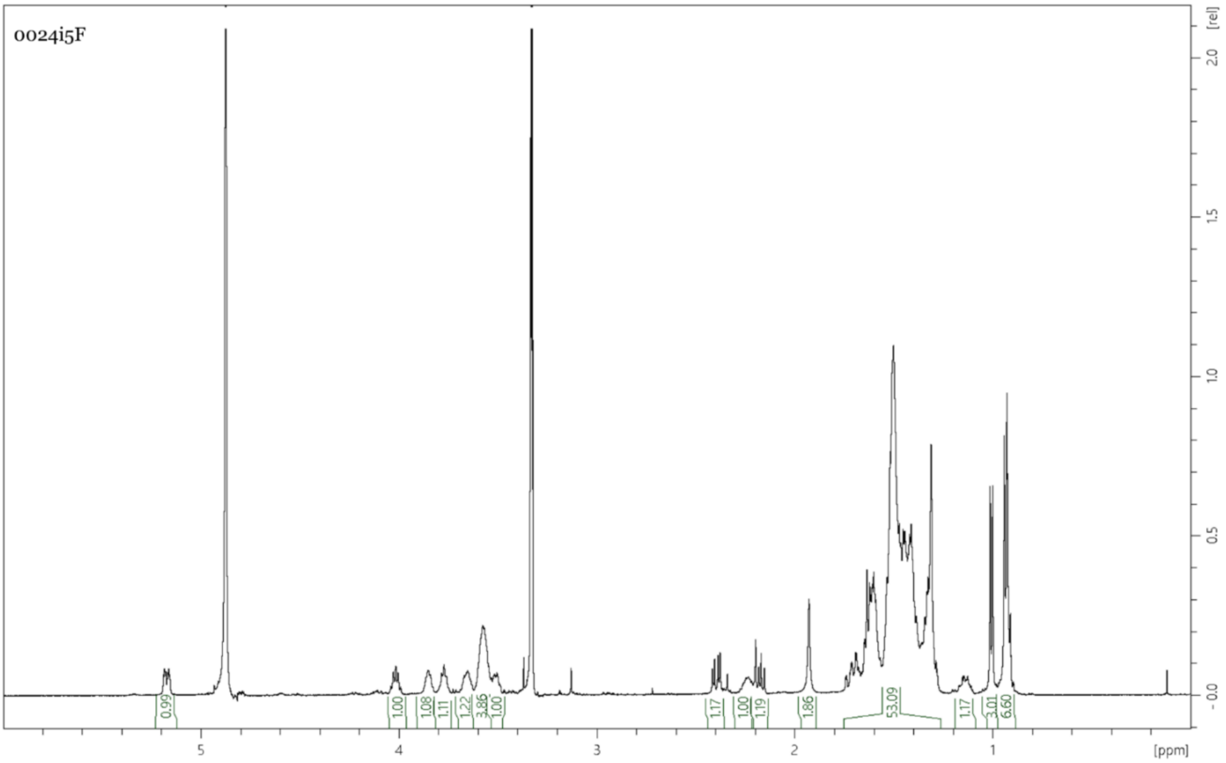

2.2.2 HSQC (DMSO-*d*6) of 0024i5F

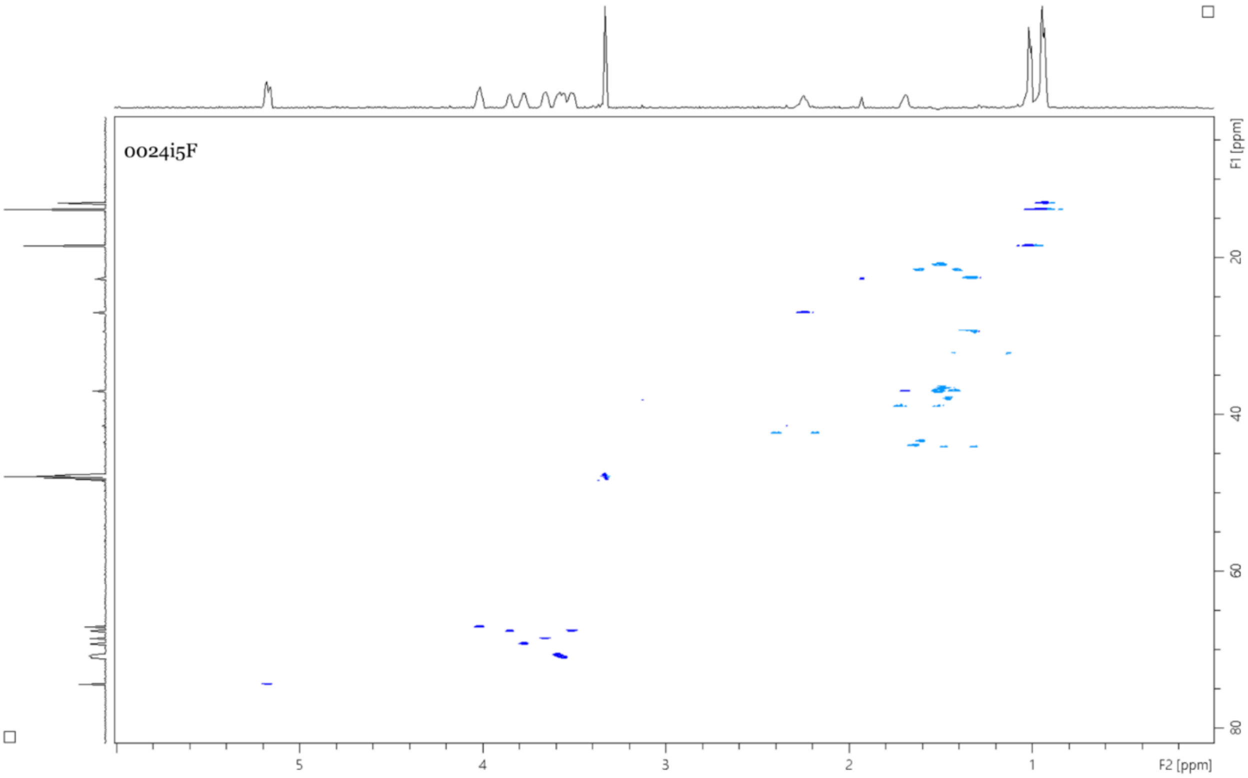

2.2.3 COSY (DMSO-*d6*) of 0024i5F

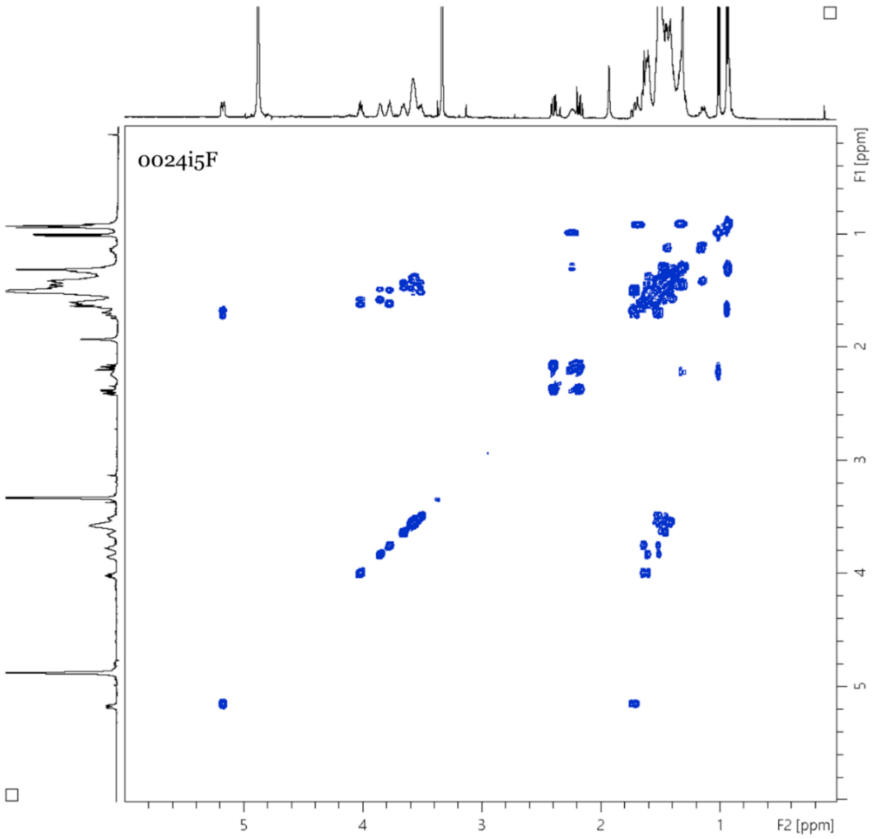

2.2.4 HMBC (DMSO-*d6*) of 0024i5F

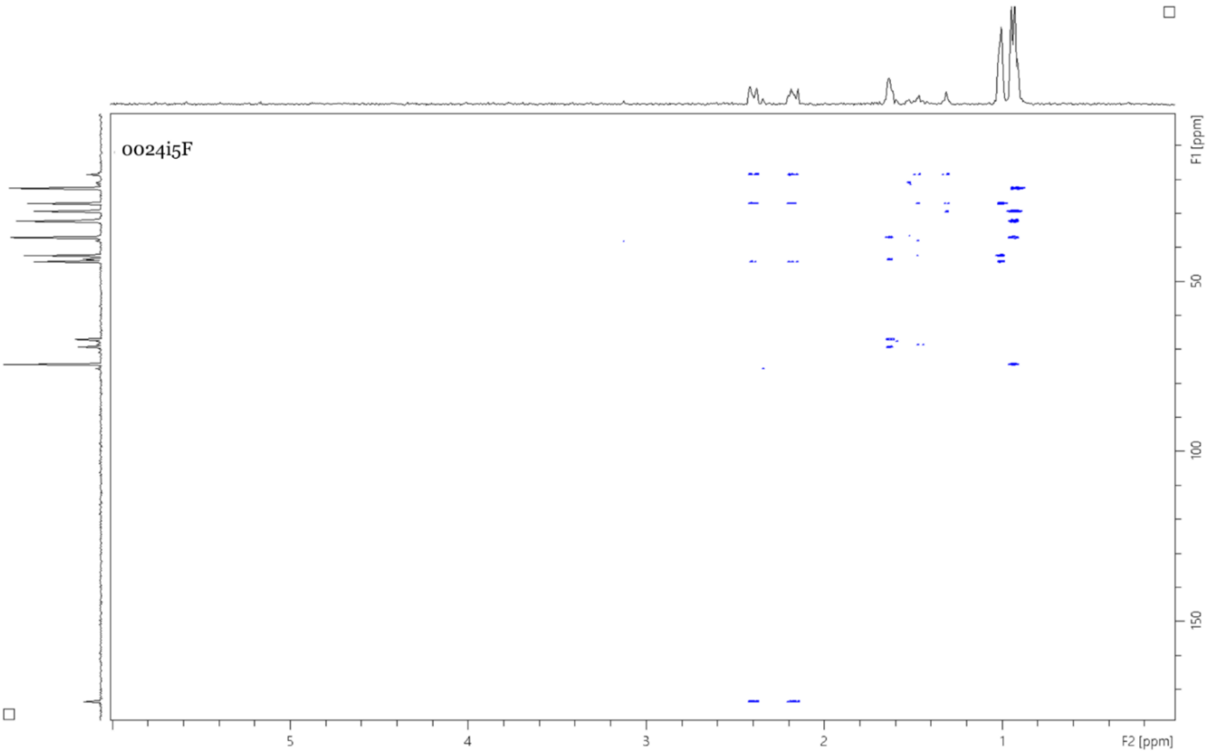

2.2.5 HSQC-TOCSY of 0024i5F

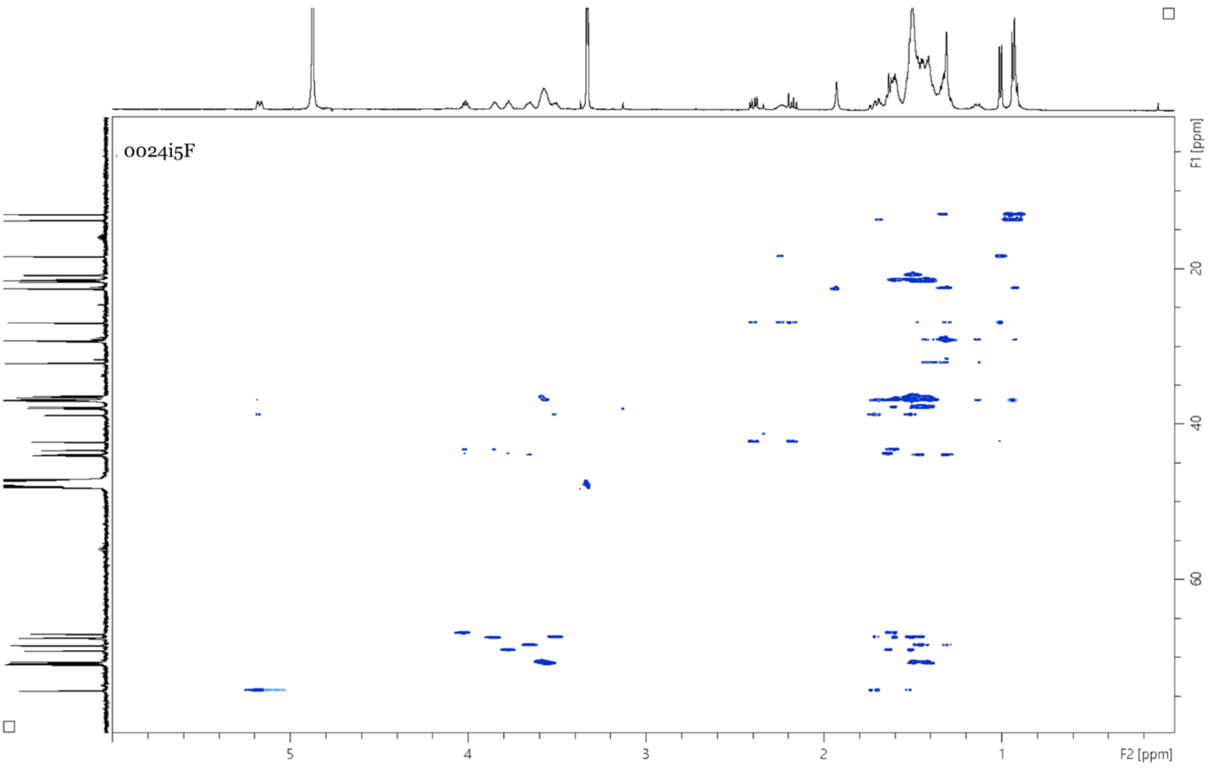

δH 0024i5F	#H	Mult	*J* [Hz]	δH Cay 2002	#H	Mult	*J* [Hz]
5.02	1	dt	9.9, 2.9	5.02	1	ddd	9.5, 6.0, 3.0
4.51	1	d	4.5	*			
4.39	1	d	4.6	*			
4.29	3	m		*			
4.21	4	m		*			
3.79	1	m		3.85	1	dq	12.4, 6.0, 5.0
3.62	1	m		3.63	1	dq	11.6, 4.5
3.55	1	m		3.55	1	dq	11.6, 5.0
3.45	1	m		3.45	1		
2.28	1	m		2.28	1	dd	13.0, 8.5
2.08	1	m		2.08	1	m	8.5, 4.5
2.08	1	m		2.05	1	dd	13.0, 4.5
1.60	1	m		1.60	1	m	
1.16-1.57	53	m		1.58	1		
				1.56	1		
				1.55	1		
				1.54	1		
				1.51	1		
				1.50	1		
				1.45	7		
				1.36	1		
				1.35	1		
				1.32	1		

				1.26	6		
				1.23	3		
1.13	1	m		1.16	1		
1.03	1	m					
0.85	3	m		0.88	3	t	6.7
0.85	3	m		0.85	3	t	6.0
0.85	3	m		0.83	3	t	7.0
3.34	H_2O						
2.51	DMSO						

Table S1: 1H-NMR Comparison of 0024i5F and caylobolide A in DMSO-*d*6.

2.3 Skeletal reassignment by NMR (pyridine-*d*5)

2.3.1 Ultra-high-resolution HSQC-TOCSY

For the full structural assignment of caylobolide A, 2 mg of genuine natural product was dissolved in 40 µL of pyridine-*d5* and added to a 1.7 mm NMR tube. The sample was subjected to an array of experiments to uniquely assign all ^{13}C and ^{1}H chemical shifts. A key experiment for the full structural assignment of such highly convoluted spectra as that in caylobolide A is the HSQC-TOCSY experiment. This was previously applied by our group to the full skeletal reassignment of synthetic bastimolide A. The HSQC-TOCSY (Heteronuclear Single Quantum Coherence-TOtal Correlation SpectroscopY) experiment is a 2D hybrid inverse gated experiment consisting of a HSQC pulse train, followed by a TOCSY mixing period. This provides a 2D spectrum which shows $^{n}J_{CH}$ correlations for a given spin system, where n is highly dependent upon the duration of the TOCSY mixing period (D9 in the pulse program parameters). For the full chemical shift assignment of caylobolide A, two HSQC-TOCSY spectra were required at mixing times of 30 ms and 60 ms. The spectra recorded with a 30 ms mixing time showed $^{1\text{-}3}J_{CH}$ correlations, which were used to assign the chemical shifts of the stereogenic carbon atoms, and their respective protons. The spectra recorded with a 60 ms mixing time showed $^{1\text{-}4}J_{CH}$ correlations, which were used to assign the chemical shifts of carbon atoms adjacent to stereocenters.

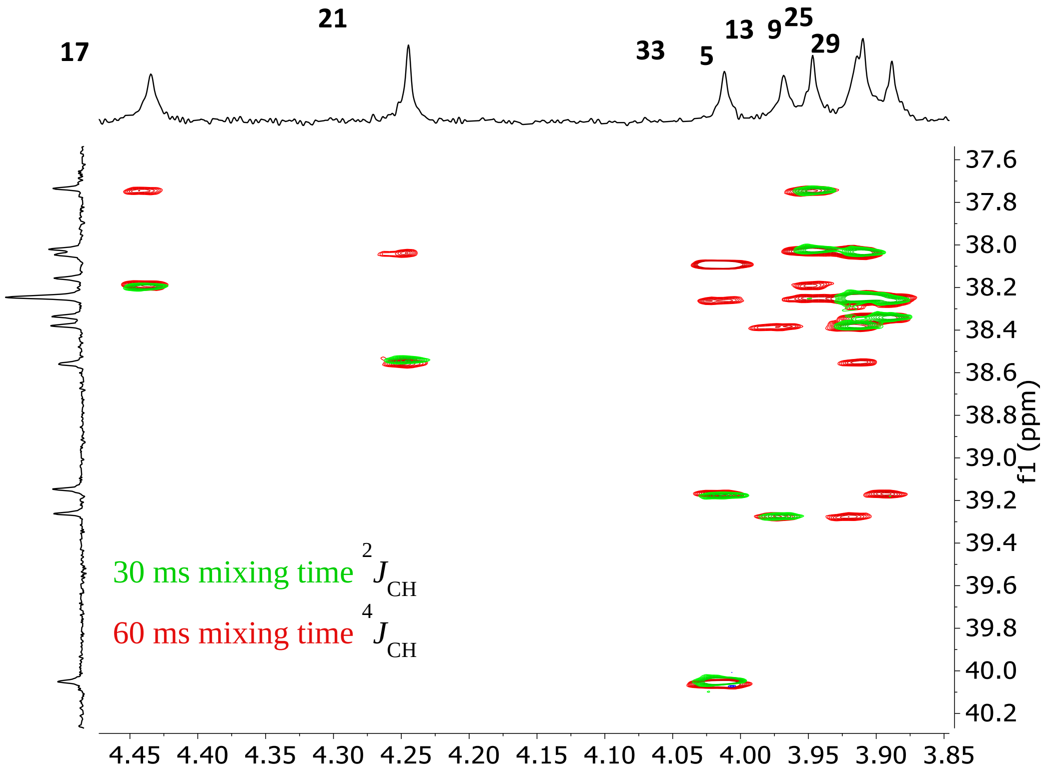

Figure S2: Overlaid HSQC-TOCSY spectra of caylobolide A in pyridine-*d*5 with TOCSY mixing times of 30 ms (green) and 60 ms (red) used to identify and differentiate between α-carbon atoms.

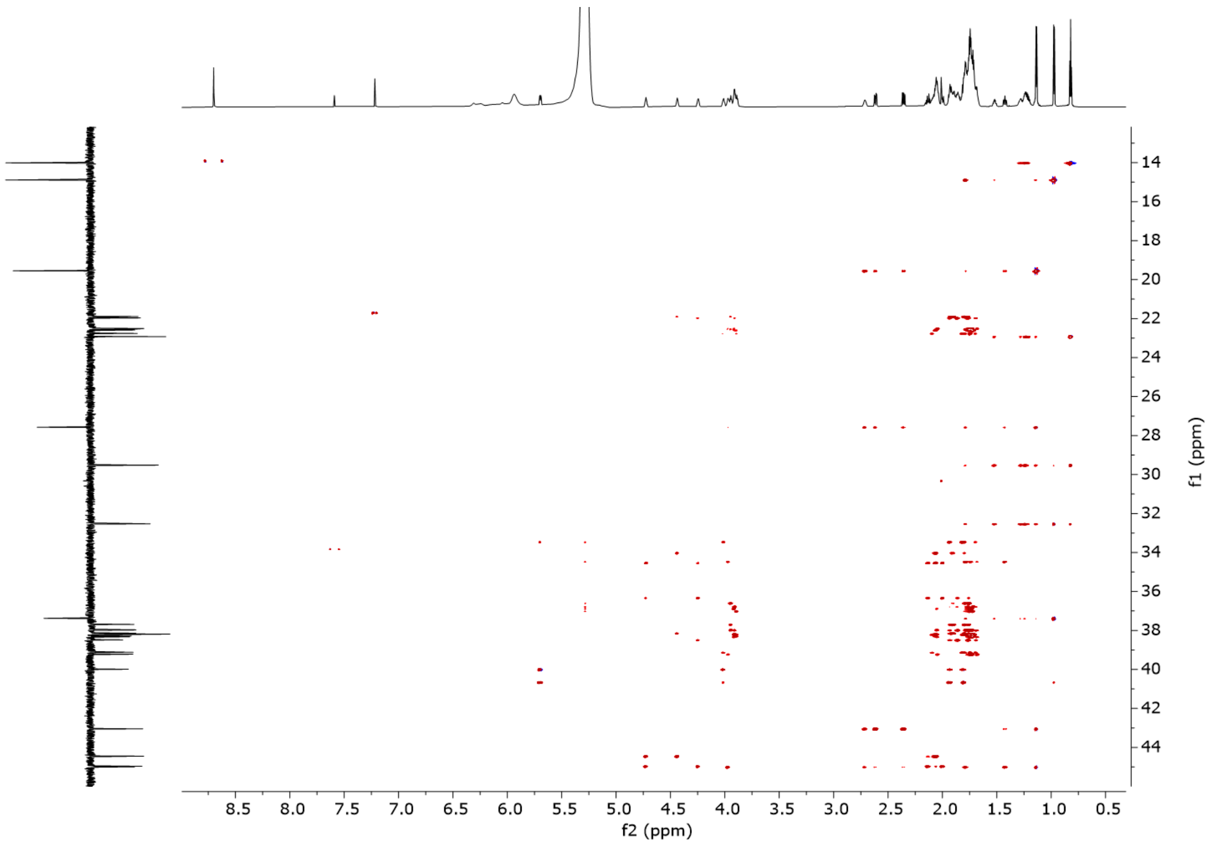

Figure S3: Ultra-high-resolution HSQC-TOCSY of caylobolide A in pyridine-*d*5 (Phase-sensitive ge-2D HSQC-TOCSY with DIPSI-2 using PEP and adiabatic inversion and refocusing pulses: hsqcdietgpsisp.2) with a 30 ms mixing time. Spectrometer frequency (949.57, 238.77), acquired size (1024, 2700),

spectral size (4096, 8192), spectral width (9469.7, 8116.9), 24 scans. Spectrum centred at the aliphatic region.

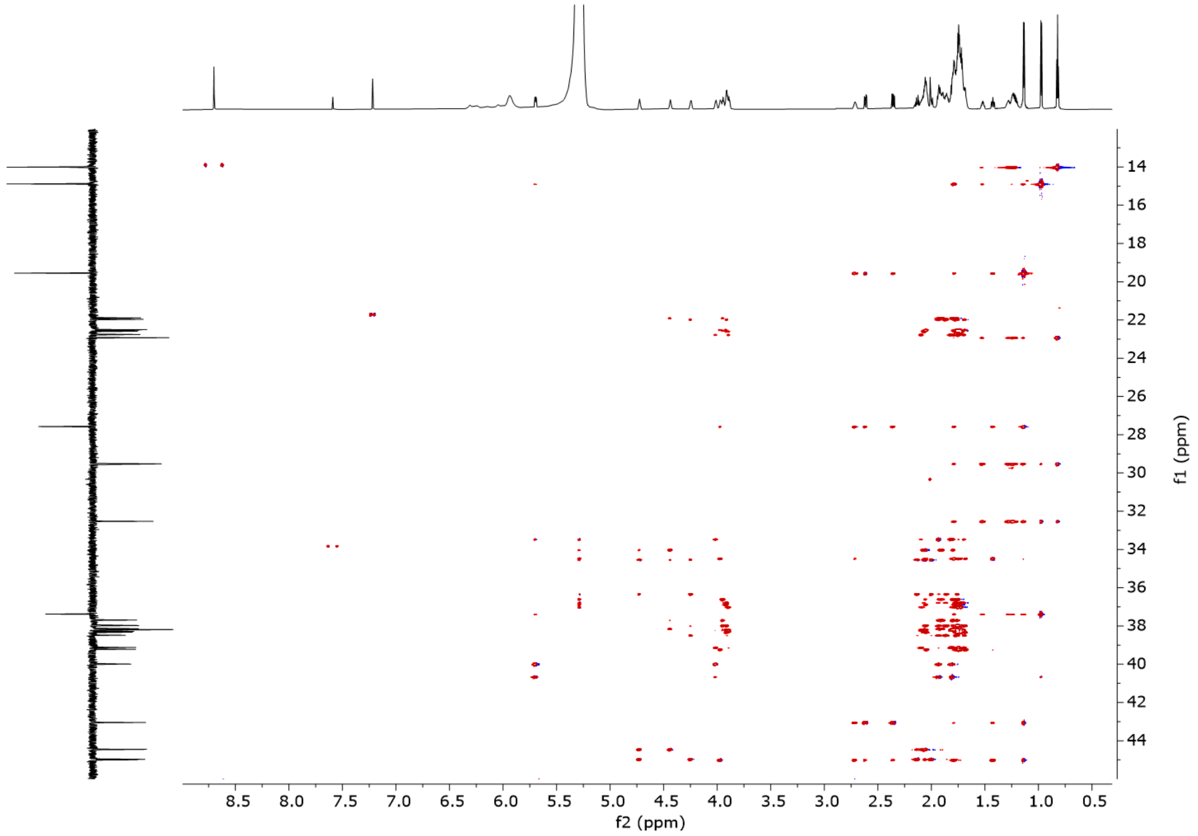

Figure S3: Ultra-high-resolution HSQC-TOCSY of caylobolide A in pyridine-*d*5 (Phase-sensitive ge-2D HSQC-TOCSY with DIPSI-2 using PEP and adiabatic inversion and refocusing pulses: hsqcdietgpsisp.2) with a 60 ms mixing time. Spectrometer frequency (700.19, 176.07) acquired size (755, 1024), spectral size (2048, 8192), spectral width (6302.5, 6443.3), 4 scans. Spectrum centred at the aliphatic region.

2.3.2 Ultra-high-resolution pureshift HSQC

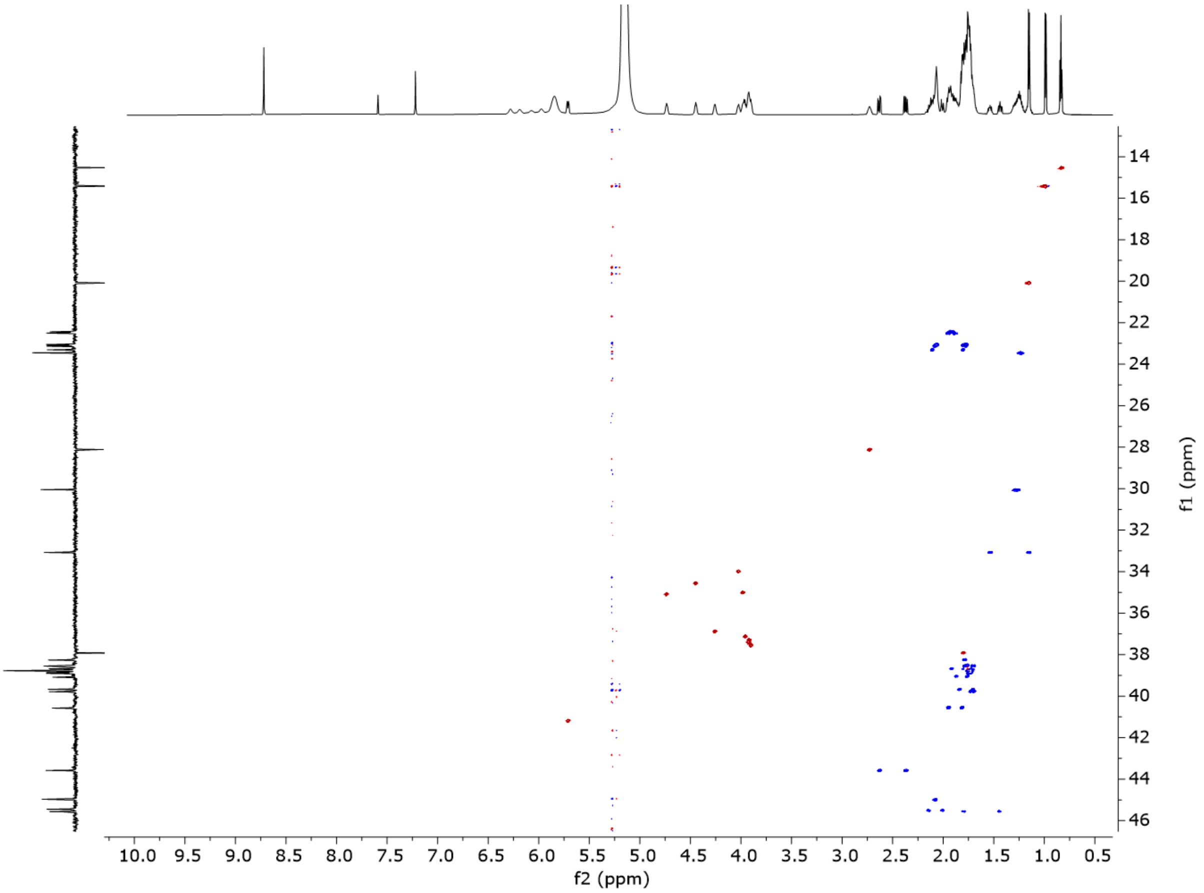

Figure S4: Ultra-high-resolution pureshift HSQC of caylobolide A in pyridine-*d*5 (Phase-sensitive ge-2D multiplicity-edited HSQC using echo-antiecho and inversion and matched sweep adiabatic pulses with broadband homodecoupling: hsqcedetgpsp.3). Spectrometer frequency (949.57, 238.77), acquired size (1024, 512), spectral size (4096, 2048), spectral width (9469.7, 8116.9), 32 scans. Spectrum centred at the aliphatic region where *CH*O correlations are folded back into the lower ppm region (correlations between 3-6 ppm in ^{1}H).

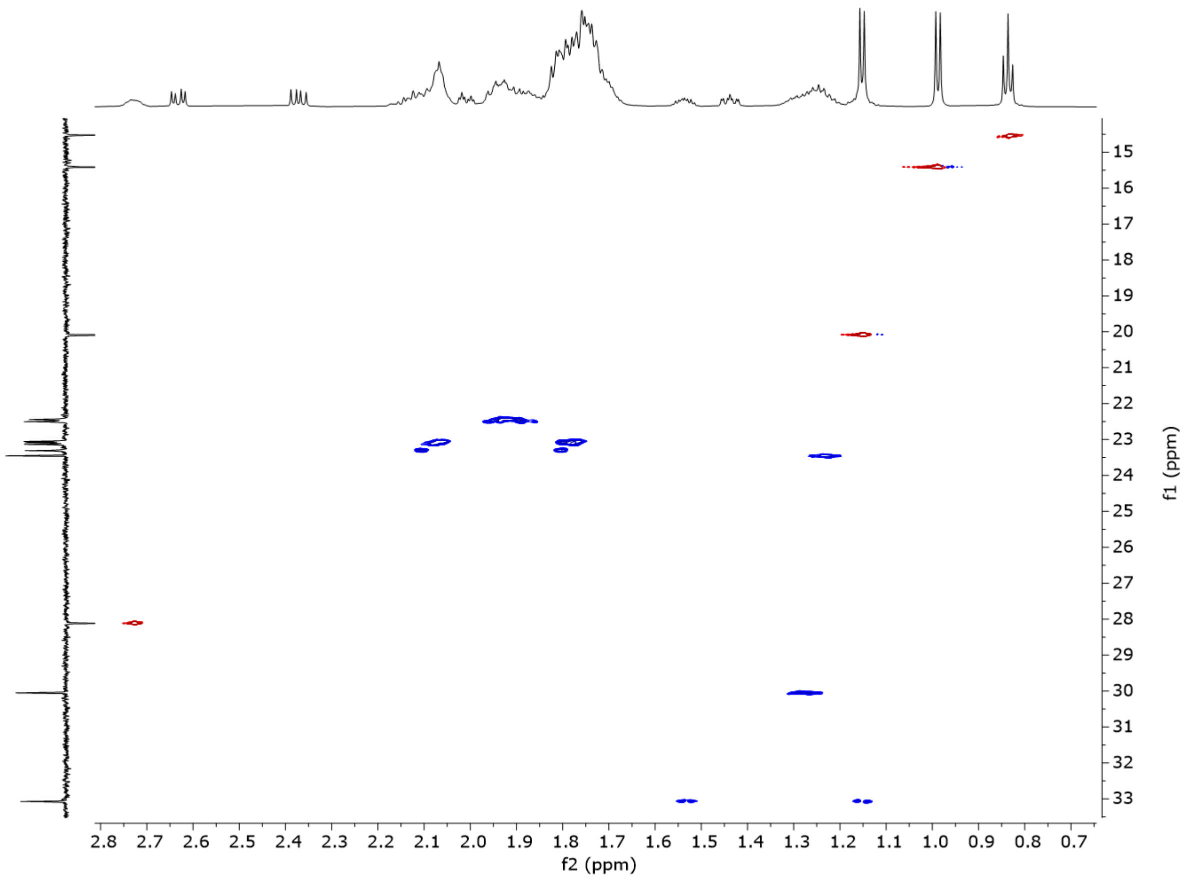

Figure S5: Zoomed in region (^{13}C: 15-33 ppm, ^{1}H: 0.7-2.8 ppm) of ultra-high-resolution pure shift HSQC of caylobolide A in pyridine-*d*5.

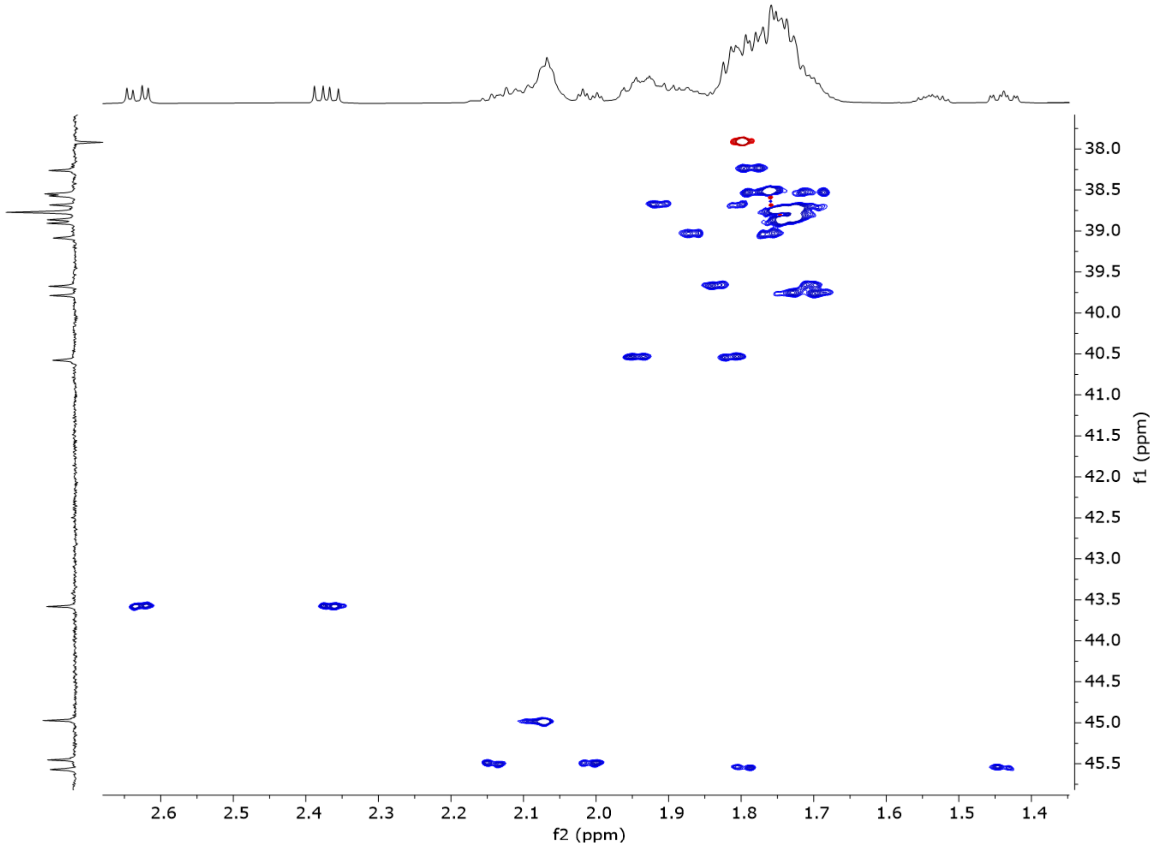

Figure S6: Zoomed in region (^{13}C: 37-46 ppm, ^{1}H: 1.4-2.7 ppm) of ultra-high-resolution pure shift HSQC of caylobolide A in pyridine-*d*5.

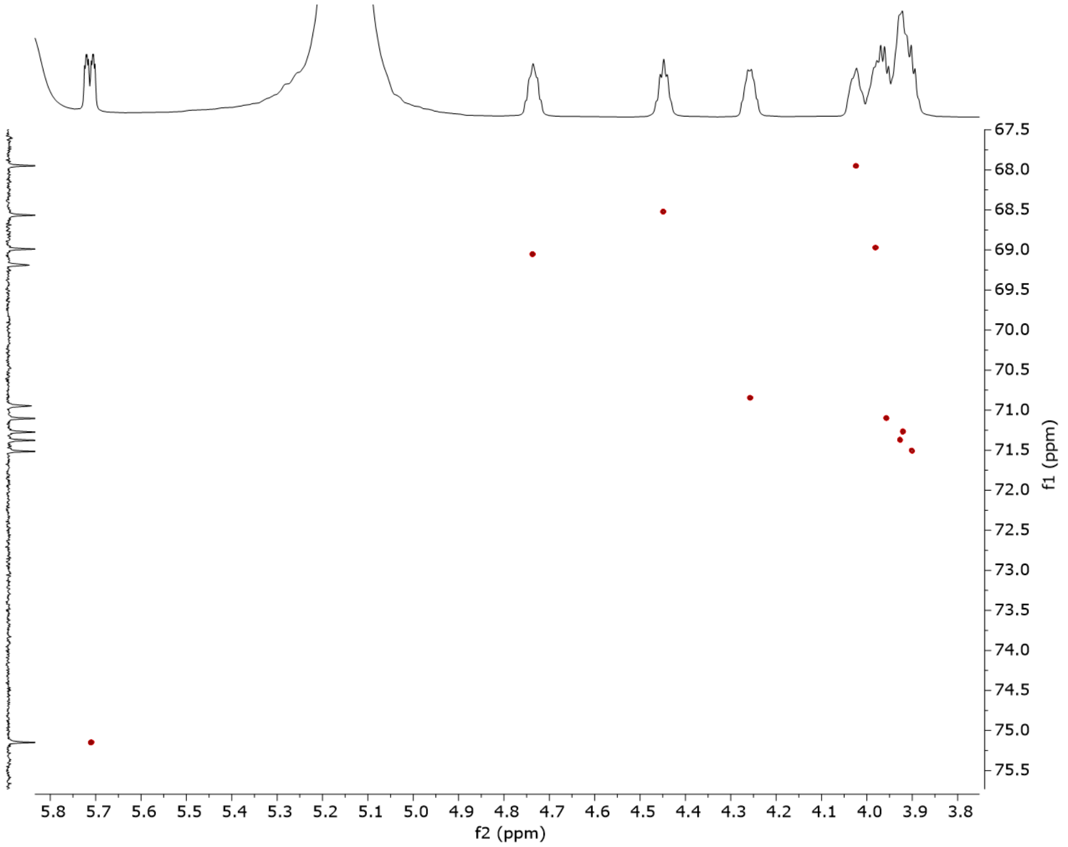

Figure S7: Zoomed in region (^{13}C: 67-76 ppm, ^{1}H: 3.8-5.8 ppm) of ultra-high-resolution pureshift HSQC of caylobolide A in pyridine-*d*5. Spectra recentred to align with *C*H*O* correlations.

2.3.3 Other 2D spectra

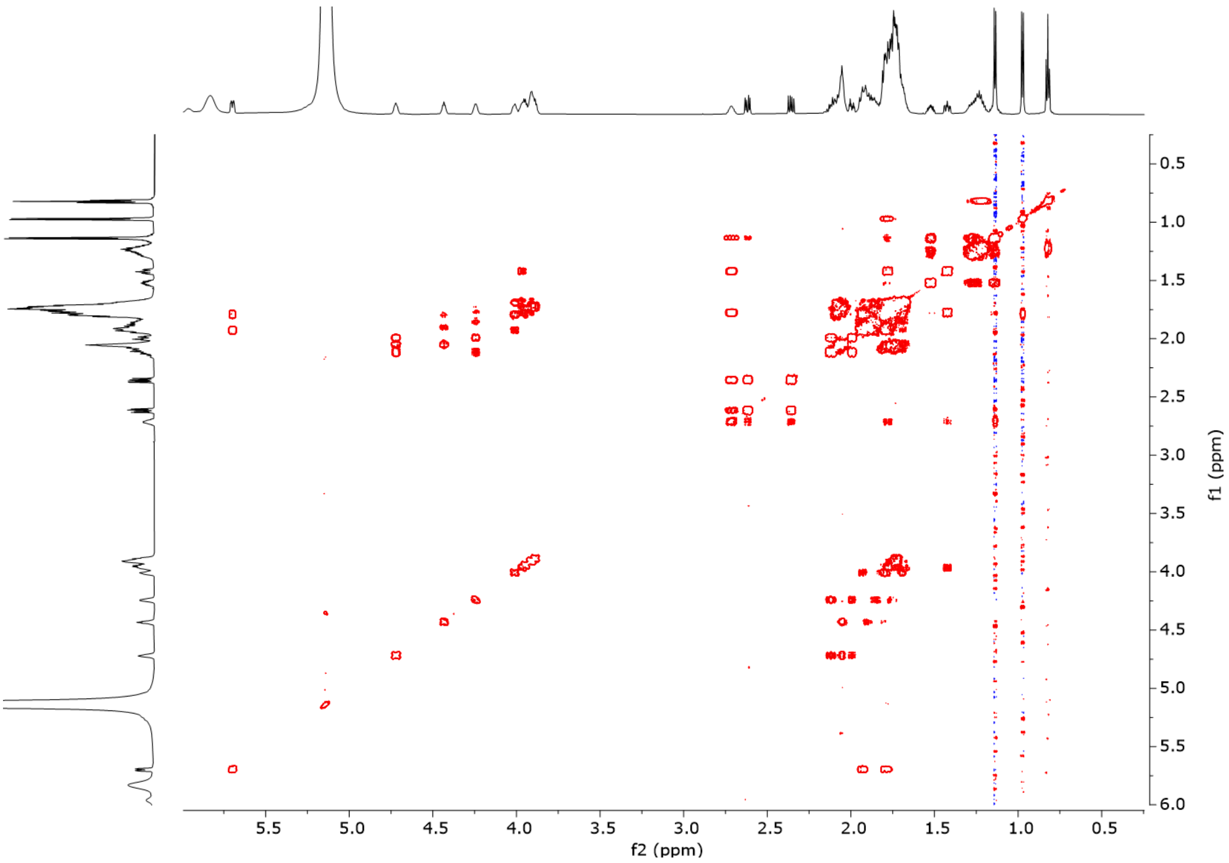

Figure S8: COSY NMR spectra of caylobolide A in pyridine-*d*5 (700 MHz, 4 scans).

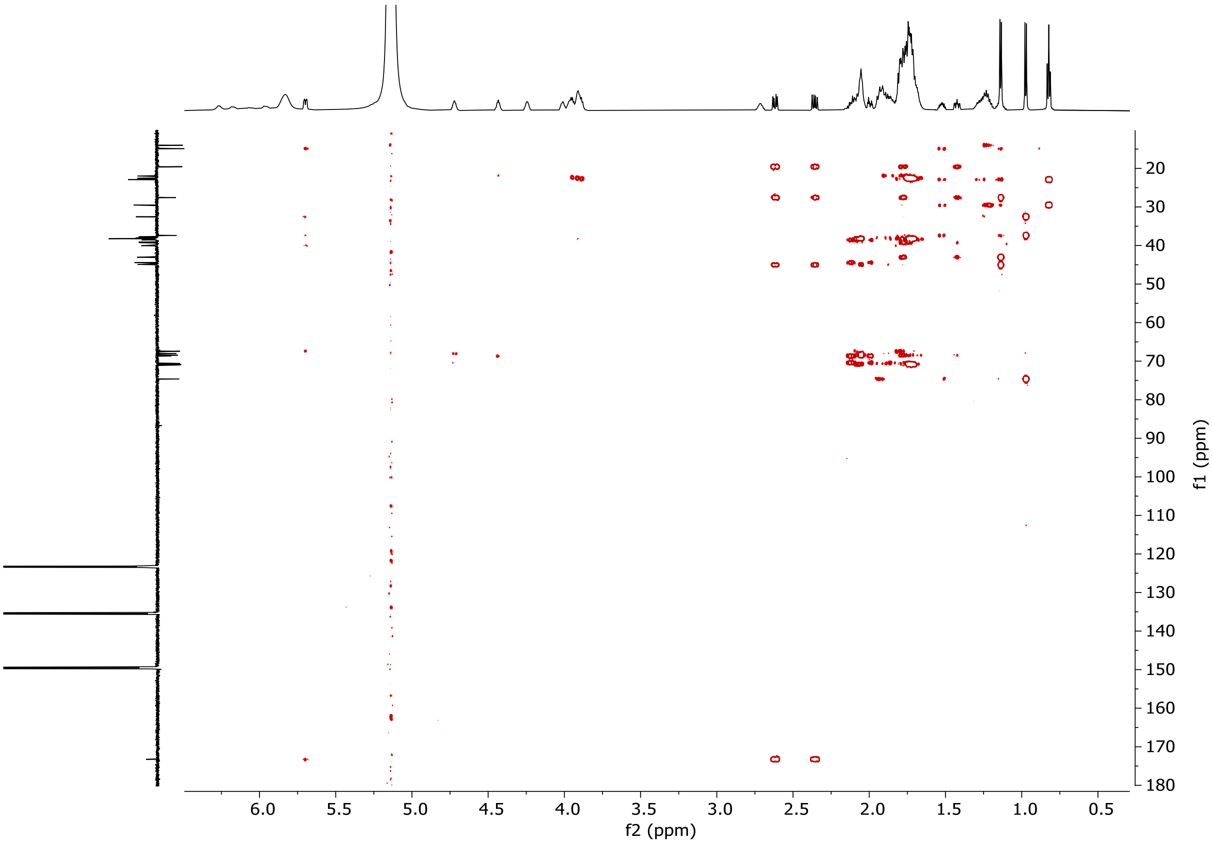

Figure S9: HMBC NMR spectra of caylobolide A in pyridine-*d*5 (700 MHz, 4 scans).

2.3.4 1D spectra of caylobolide A

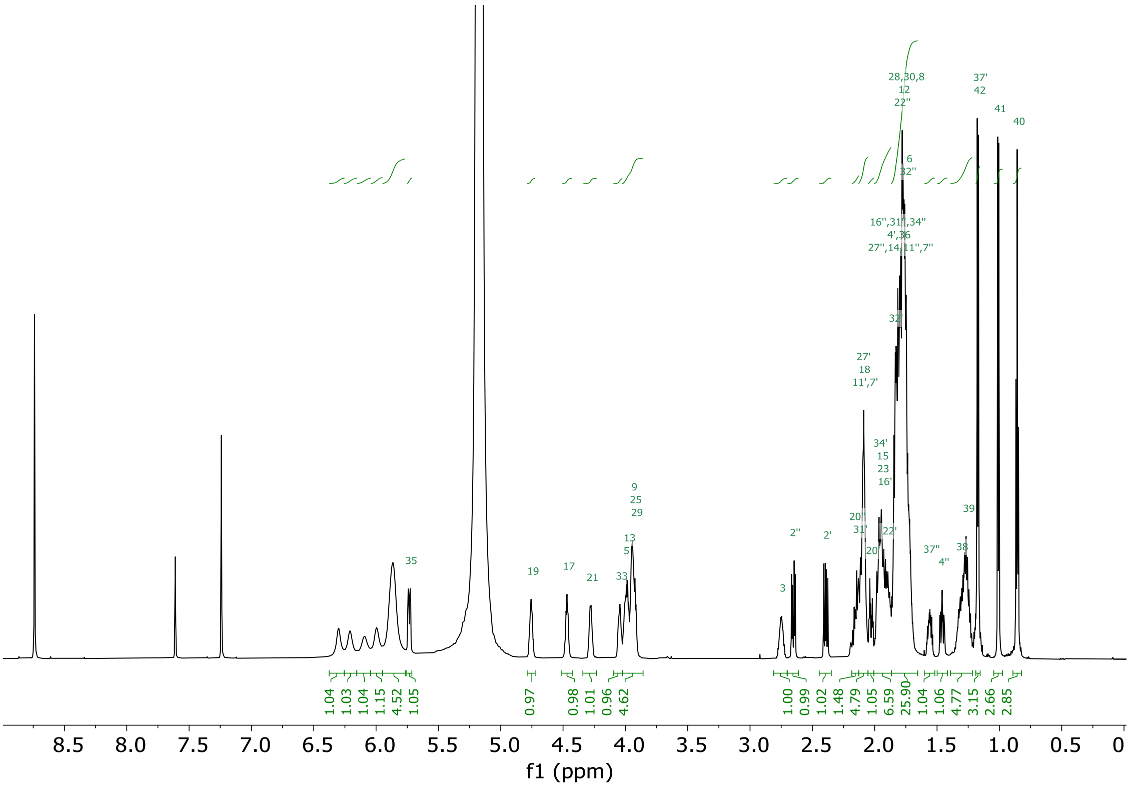

Figure S10: ^{1}H NMR spectra of caylobolide A in pyridine-*d*5 (700 MHz, 16 scans).

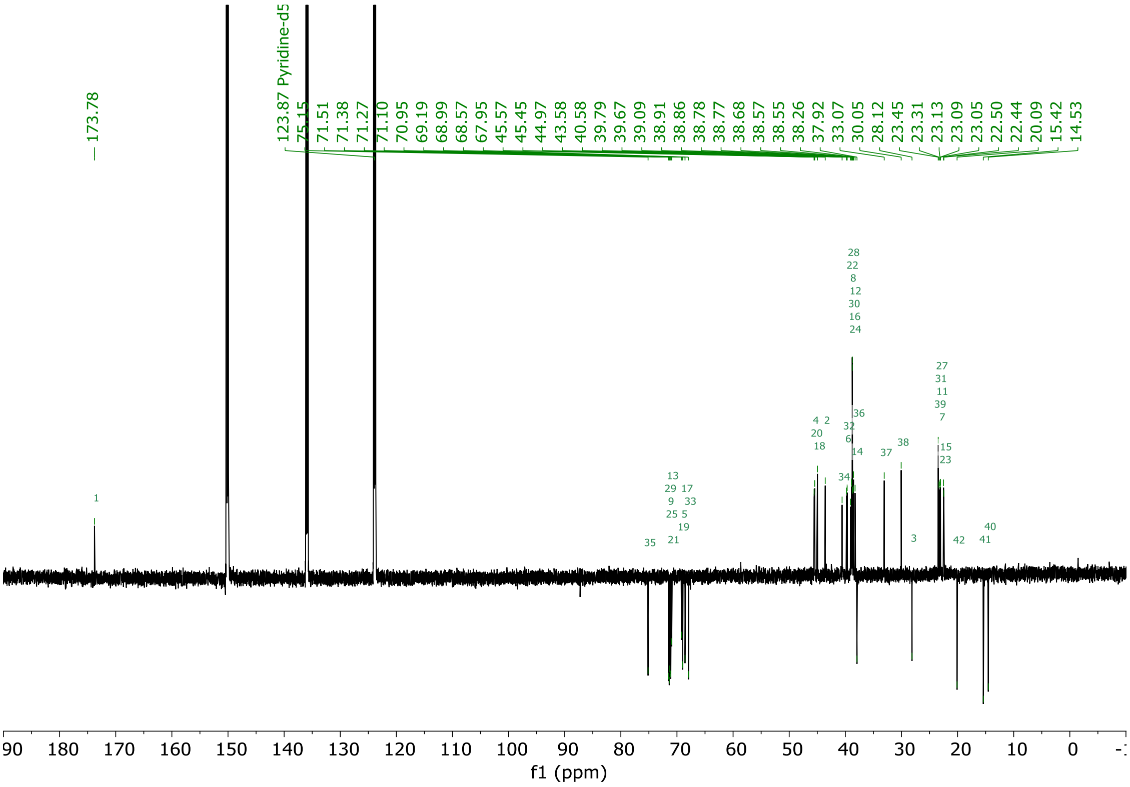

Figure S11: ^{13}C DEPTQ - DEPT with quaternary carbons using gradients NMR spectra of caylobolide A in pyridine-*d*5 (176 MHz).

Table S2: 1H-NMR of 0024i5F (caylobolide A) in pyridine-*d5*. ^{13}C chemical shifts referenced to pyridine (123.87 ppm) and ^{1}H NMR referenced to pyridine (7.22 ppm). H_2O signal appears at 5.15 ppm.

Atom	13C δ (ppm)	#H	1H δ (ppm)	Mult	J (Hz)
**1**	173.78				
**2**	43.58	1	2.36	dd	14.7, 8.4
		1	2.62	dd	14.7, 5.8
**3**	28.12	1	2.72		
**4**	45.57	1	1.79		
		1	1.42	ddd	13.1, 9.3, 3.1
**5**	68.99	1	3.97		
**6**	39.79	2	1.70		
**7**	23.05	1	2.06		
		1	1.78		
**8**	38.91	2	1.73		
**9**	71.38	1	3.91		
**10**	38.77	2	1.72		
**11**	23.09	1	2.06		
		1	1.78		
**12**	38.55	2	1.75		
**13**	71.1	1	3.95		
**14**	38.26	2	1.78		
**15**	22.44	2	1.92		
**16**	38.68	1	1.90		
		1	1.80		
**17**	68.57	1	4.43	dddd	5.9, 5.9, 5.7, 5.7
**18**	44.97	2	2.06		
**19**	69.19	1	4.72	dddd	5.3, 5.3, 5.3, 5.3
**20**	45.45	1	1.99	ddd	13.8, 4.2, 4.2
		1	2.12	ddd	14.8, 8.8, 8.8
**21**	70.95	1	4.24	dddd	4.7, 4.7, 4.5, 4.5
**22**	39.09	1	1.86		
		1	1.76		
**23**	22.5	2	1.91		
**24**	38.57	1	1.69		
		1	1.77		
**25**	71.27	1	3.9		
**26**	38.77	2	1.72		
**27**	23.13	1	2.07		
		1	1.78		
**28**	38.86	2	1.73		
**29**	71.51	1	3.89		
**30**	38.78	2	1.73		
**31**	23.31	1	2.10		
		1	1.80		
**32**	39.67	1	1.81		
		1	1.71		
**33**	67.95	1	4.01		
**34**	40.58	1	1.94		
		1	1.80		
**35**	75.15	1	5.70	ddd	10.2, 3.7, 2.3
**36**	37.92	2	1.79		
**37**	33.07	1	1.13		
		1	1.53	dddd	13.2, 10.5, 5.2, 5.2
**38**	30.05	2	1.28		
**39**	23.45	2	1.23		
**40**	14.53	3	0.82	t	7.0
**41**	15.42	3	0.97	d	6.8
**42**	20.09	3	1.14	d	6.6

2.4 Stereochemical reassignment by NMR

2.4.1 Kishi's 1,3-diol analysis (universal NMR database)

Analysis of the 1,3,5-triol of caylobolide A using Kishi's universal NMR database indicates an *anti*-*syn* relationship for carbons C17-C19-C21 respectively.[1-4] Observations made by Kishi were recorded in MeOD and DMSO-*d*6 and so were appropriately scaled through analysis of the chemical shifts of a known cyclic 1,3,5-triol recorded in pyridine-*d*5 belonging to bastimolide A.[5] ^{13}C data reported for bastimolide A made no reference to being referenced to a solvent signal, and so it is suspected that the 1,3,5-triol region of bastimolide A and caylobolide A are identical, but are not correctly referenced due to there being similar chemical shift differences ($\Delta\delta_{BA\text{-}CA}$ = 0.2 ppm).

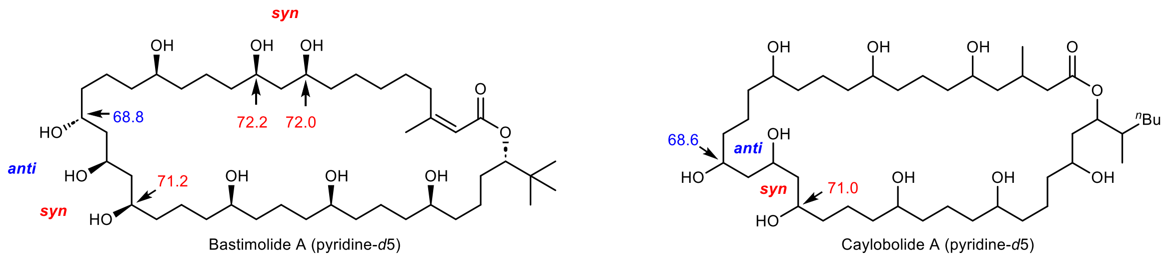

Figure S12: Analysis of caylobolide A 1,3,5-triol (C17-C21) by Kishi's universal NMR database. ^{13}C Chemical shifts of >70 ppm or <70 ppm indicates a *syn* or *anti* 1,3-diol relationship respectively.

2.4.2 Mosher ester analysis

Caylobolide A nona-Mosher ester derivatisation

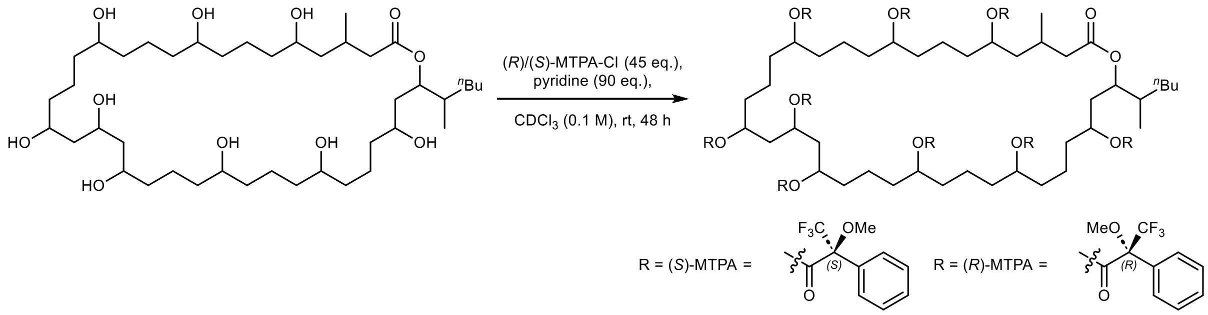

To a suspension of Caylobolide A (0.7 mg, 0.92 µmol, 1.0 equiv.) in $CDCl_3$ (92 µL, 0.01 M), pyridine (6.5 mg, 6.6 µL, 83 µmol, 90 equiv.) and (*R*)- *or* (*S*)-MTPA chloride (10 mg, 7.7 µL, 41 µmol, 45 equiv.) were added and the resulting mixture stirred for 16 h at room temperature. Saturated sodium hydrogen carbonate was added to quench the reaction mixture, which was then blown down under a stream of nitrogen. The resulting residue was resuspended in CH_2Cl_2, filtered over a short pad of silica, and evaporated under a stream of nitrogen to give a colourless film. The crude material was then dissolved in pyridine-*d5*

(99.96% purity) and analysed by NMR spectroscopy to extract relevant chemical shifts for Moshers ester analysis.

2.4.3 Mosher ester NMR spectra

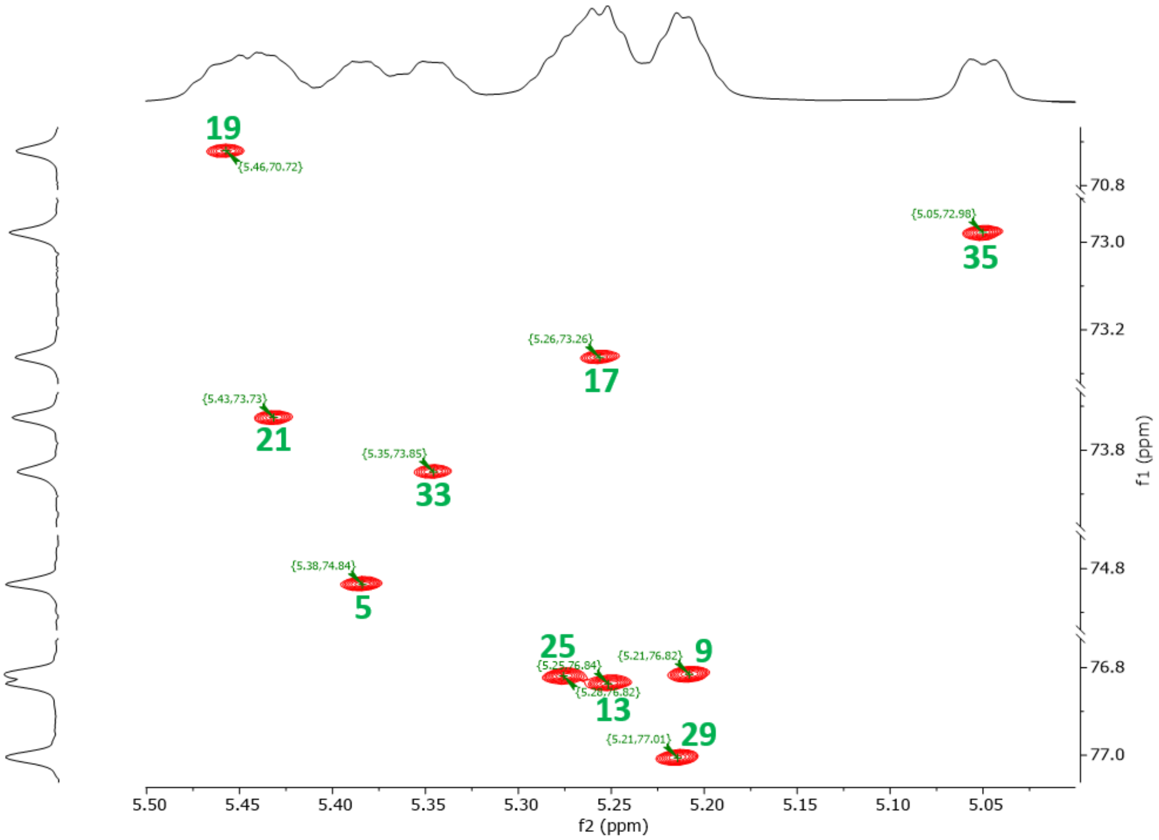

Figure S13: Ultra-high-resolution HSQC of caylobolide A (*S*)-nona Mosher ester (pyridine-*d*5) zoomed in to the region containing *CH*O correlations with atom numbers assigned to each correlation.

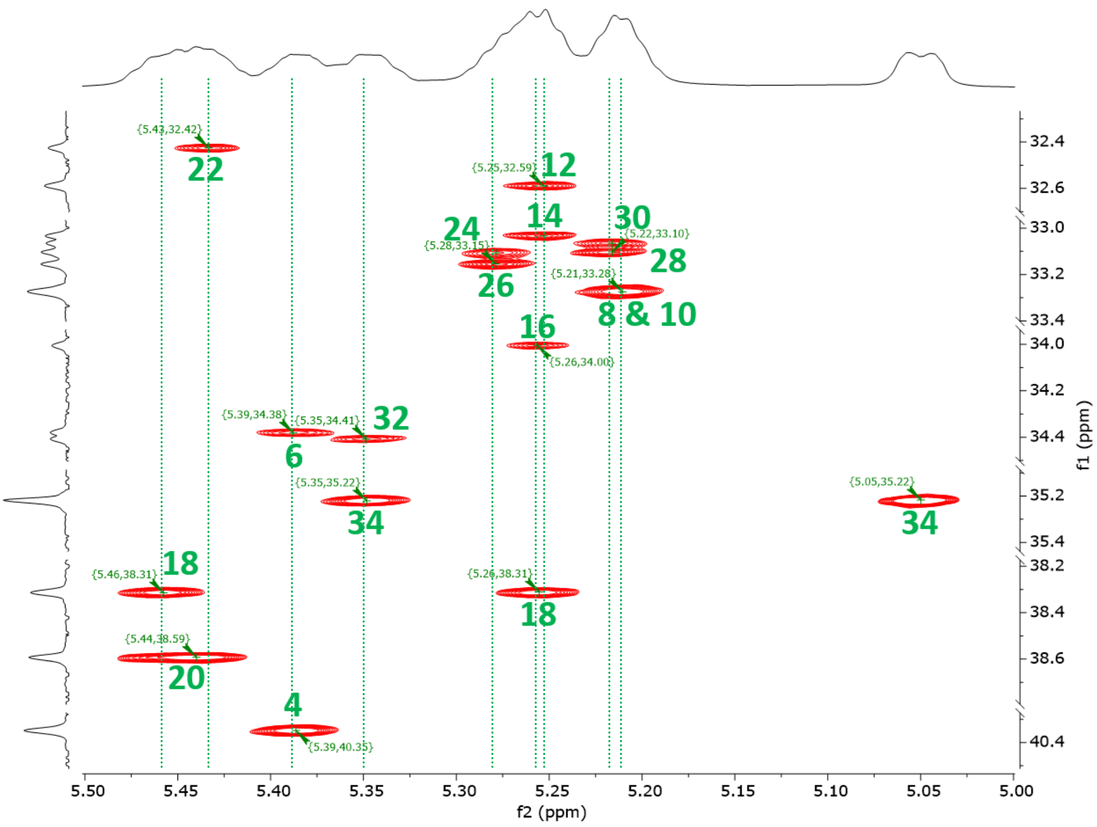

Figure S14: Ultra-high-resolution HSQC-TOCSY (30 ms mixing time) of caylobolide A (*S*)-nona Mosher ester (pyridine-*d*5) zoomed into the region displaying $^{2}J_{CH}$ correlations used for the determination of α-CH_2 chemical shifts. Atom numbers assigned to ^{13}C chemical shifts for each correlation.

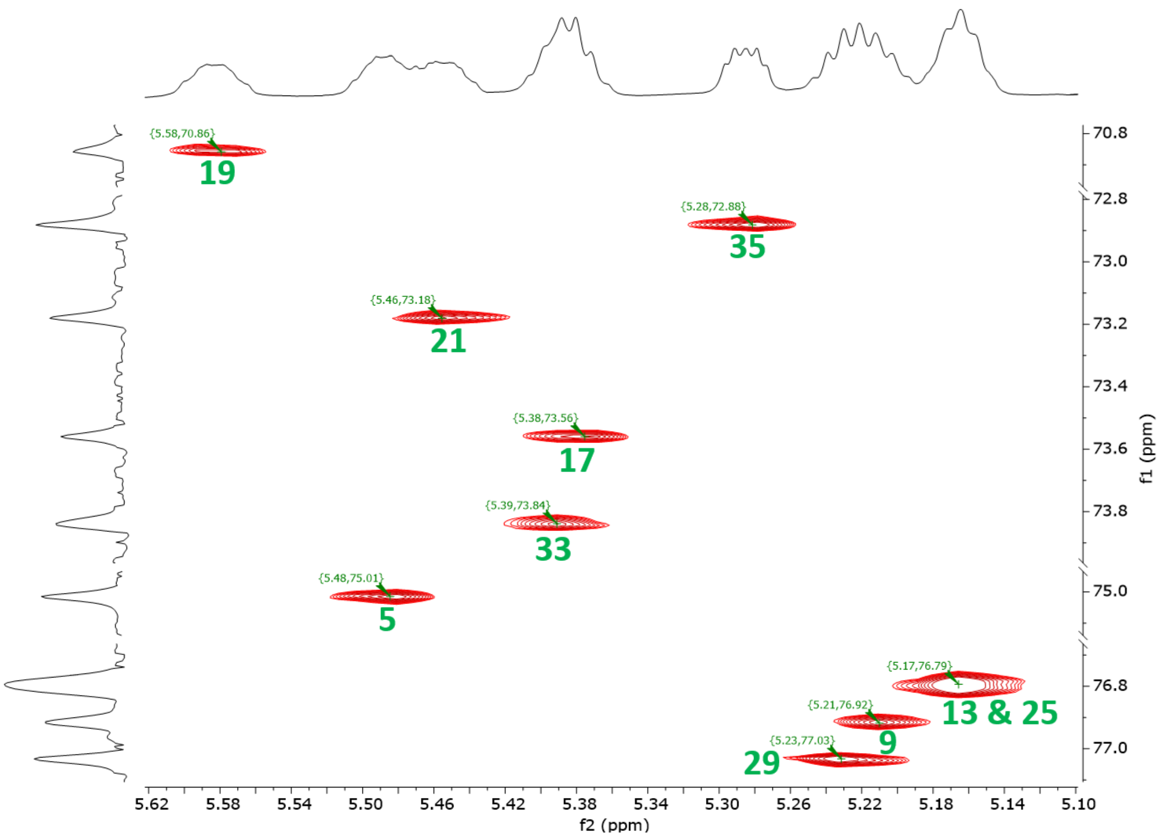

Figure S15: Ultra-high-resolution HSQC of caylobolide A (*R*)-nona Mosher ester (pyridine-*d*5) zoomed in to the region containing *CH*O correlations with atom numbers assigned to each correlation.

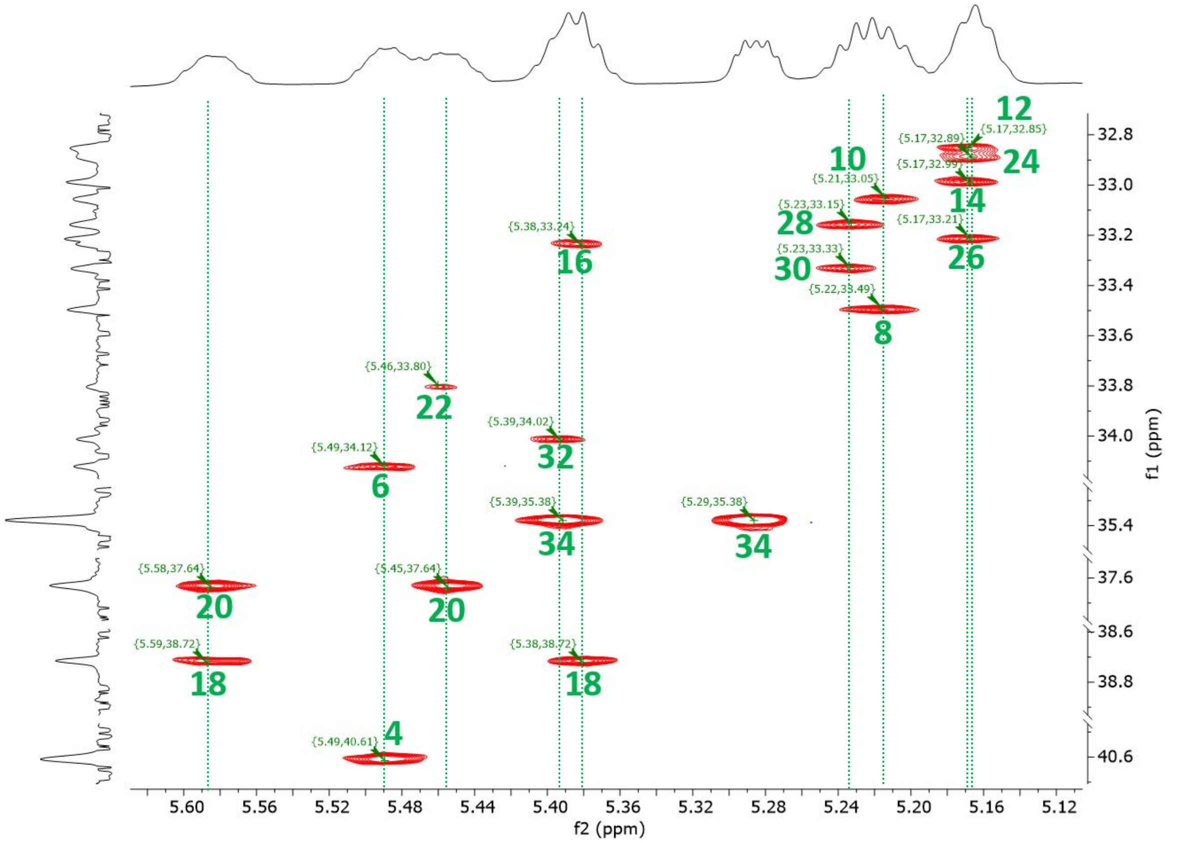

Figure S16: Ultra-high-resolution HSQC-TOCSY (30 ms mixing time) of caylobolide A (*R*)-nona Mosher ester (pyridine-*d*5) zoomed into the region displaying $^{2}J_{CH}$ correlations used for the determination of α-CH_2 chemical shifts. Atom numbers assigned to ^{13}C chemical shifts for each correlation.

2.4.4 Simplified Mosher ester analysis

Carbon chemical shifts were extracted in an analogous way to that of the parent caylobolide A using ultra-high-resolution HSQC-TOCSY spectra. $\Delta\delta^{SR}$ calculated by $\delta_S - \delta_R$ to give differential chemical shifts of α-carbon atoms between the (*S*) and (*R*) Mosher esters. $\Delta\Delta\delta^{SR}$ calculated by $\Delta\delta^{SR(n-1)}$ - $\Delta\delta^{SR(n+1)}$ where n is the atom number of the stereocentre of interest (Table S3). If the value of $\Delta\Delta\delta^{SR}$ calculated is negative then the stereochemistry is "up" when oriented with the lower numbered atom on the right, and the hydroxylated centre is pointing up as shown below.

Table S3: Mosher ester analysis ^{13}C values for caylobolide A 1,5-polyol region.

**Atom**	**C5**		**C9**		**C13**		**C25**		**C29**		**C33**		**C**	
**α-Atom**	**C4**	**C6**	**C8**	**C10**	**C12**	**C14**	**C24**	**C26**	**C28**	**C30**	**C32**	**C34**	**R**^{1}	**R**^{2}
**δ-(*S*)**	40.35	34.38	33.26	33.28	32.59	33.03	33.10	33.16	33.10	33.06	34.41	35.22	δSR^1	δSR^2
**δ-(*R*)**	40.61	34.12	33.50	33.05	32.86	32.98	32.89	33.21	33.16	33.33	34.01	35.38	δRR^1	δRR^2
$\Delta\delta^{SR}$	-0.26	0.26	-0.24	0.23	-0.27	0.05	0.21	-0.05	-0.06	-0.27	0.40	-0.16	$\Delta\delta^{SR1}$	$\Delta\delta^{SR2}$
$\Delta\Delta\delta^{SR}$	**-0.52**		**-0.47**		**-0.32**		**0.26**		**0.21**		**0.56**		**+ve or –ve**	

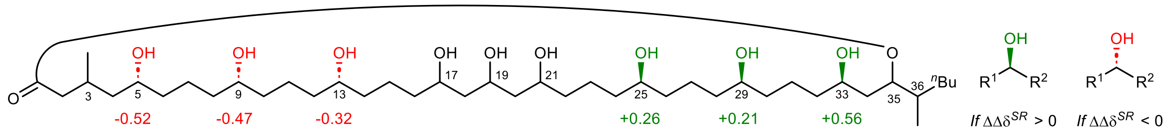

This ^{13}C-based Mosher's ester analysis was found not to work for the 1,3,5-triol system from C17-C19-C21, suggesting an *anti-anti* relative stereochemistry which contradicted the *anti-syn* determination using Kishi's universal NMR database (*see section 2.4.1*). Hence we reverted to the more usual ^{1}H NMR chemical shifts, with careful consideration of the pairwise additive effect of the proximal Mosher esters.[6-8] A qualitative prediction of the signs of $\Delta\delta^{SR}$ for the protons at each carbon for the two possible enantiomeric situations were predicted (Figure S17**).**

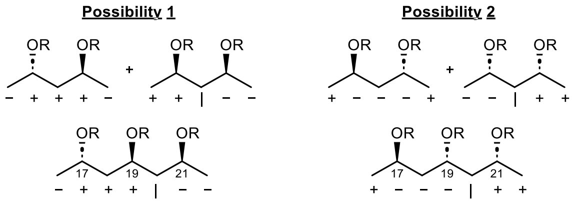

Figure S17: Predicted pairwise additive effects in the Mosher ester analysis of the anti-syn 1,3,5-triol (C17-C21) portion of caylobolide A. **–** $\Delta\delta^{SR} < 0$; **+** $\Delta\delta^{SR} > 0$; **|** (equivocal) $\Delta\delta^{SR} \approx 0$.

Upon comparison of our predicted signs to the measured $\Delta\delta^{SR}$ values ("Average $\Delta\delta^{SR}$", Table S4), it was clear that the "Possibility 2" configuration in Figure S17 closely matched the pattern of signs.

Table S4: Mosher ester analysis ^{1}H values for caylobolide A 1,3-polyol region.

**Atom**	**H16**		**H17**	**H18**		**H19**	**H20**		**H21**	**H22**	
**δ-(*S*)**	1.65	1.75	5.26	2.02	2.18	5.45	2.08	2.39	5.43	1.78	1.85
**δ-(*R*)**	1.6	1.66	5.38	2.09	2.45	5.59	2.17	2.37	5.45	1.65	1.74
$\Delta\delta^{SR}$	0.05	0.09	−0.12	−0.07	−0.27	−0.14	−0.09	0.02	−0.02	0.13	0.11
**Average** $\Delta\delta^{SR}$	0.07		−0.12	−0.17		-0.14	−0.035		−0.02	0.12	
+/−/|	+		−	−		−	|		|	+	
$\Delta\Delta\delta^{SR}$			+0.24			-0.135			-0.155		

To further test this assignment of stereochemistry, we calculated $\Delta\Delta\delta^{SR}$ values for the 1,3,5-triol region of caylobolide – namely the difference in $\Delta\delta^{SR}$ between the methylene groups adjacent to the stereocentre (so the difference H16 and H18 to assess $\Delta\Delta\delta^{SR}$ for C17, and H18 and H20 to assess $\Delta\Delta\delta^{SR}$ for C19, etc). The expected signs of the $\Delta\Delta\delta^{SR}$ values were predicted for the eight possible configurations of a 1,3,5-triol (Figure S18a) and matched well to the experimental values (Table S4) to give the final structure as shown in Figure S18b which

describes the absolute stereochemistry of all nine hydroxylated stereocentres of caylobolide A.

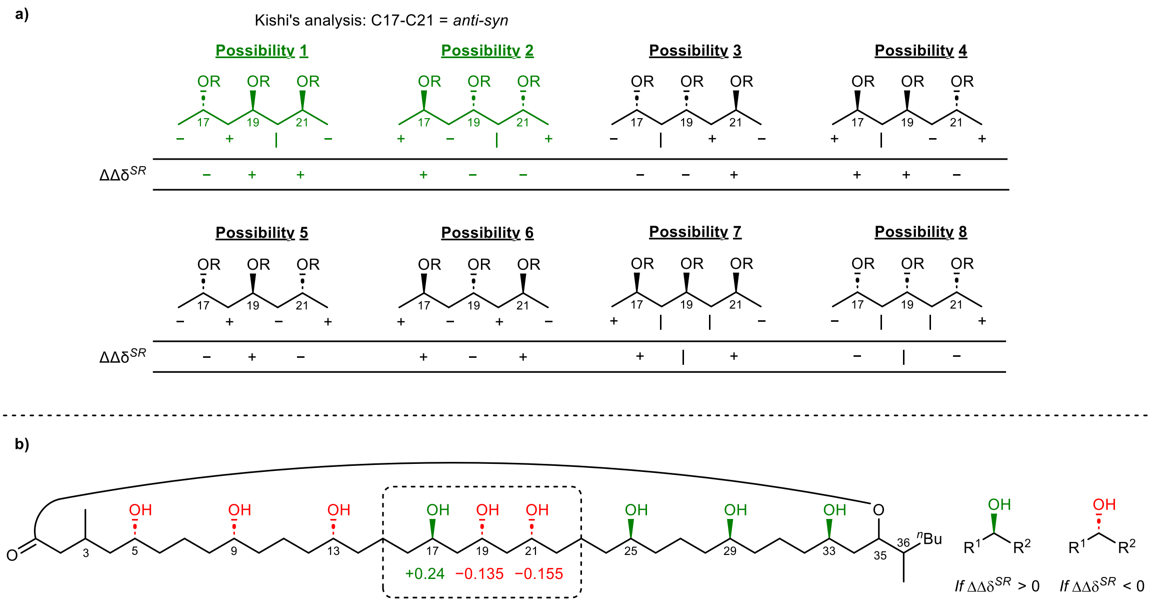

Figure S18: a) Predicted ΔΔδ^{SR} pairwise additive effects in the Mosher ester analysis of the anti-syn 1,3,5-triol (C17-C21) portion of caylobolide A. ΔΔδ^{SR} = Δδ$^{SR}_{(n-1)}$ - Δδ$^{SR}_{(n+1)}$. − Δδ^{SR} < 0; + Δδ^{SR} >0; | (equivocal) Δδ^{SR} ≈ 0. **b)** The absolute stereochemistry of all hydroxylated stereocentres of caylobolide A, reoriented for simplicity of analysis.

3 GENERAL PROCEDURES

General procedure 1 (GP1) – α-sulfinylbenzoate preparation

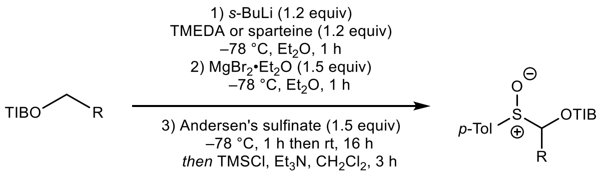

According to the modified literature procedure[9], TMEDA/sparteine (1.20 equiv.) was added to a flame dried two-neck round bottomed flask under nitrogen, followed by the TIB ester (1.00 equiv.). The mixture was degassed by stirring under vacuum for 30 min, then dry diethyl ether (0.30 M *wrt* TIB ester) was added and the reaction mixture was cooled to −78 °C. To the reaction mixture, *s*-BuLi (1.30 M hexane, 1.20 equiv.) was added via syringe pump (1.00 mL/min). The reaction mixture was stirred 1 h at −78 °C. In a separate flame dried two-neck round bottomed flask under nitrogen with a condenser, to a stirred suspension of Mg turnings (4.00 equiv.) in Et_2O (2.00 M), dibromoethane (1.50 equiv.) was added dropwise (*CAUTION: exothermic reaction*). After reflux and gas evolution ceased, the

$MgBr_2$•Et_2O solution was stirred for 30 min at room temperature (*formed 2 layers, top colourless, bottom grey*). The solution was then transferred to the main reaction vessel via syringe pump (1.00 mL/min). The reaction mixture was stirred at −78 °C for 2 h. To this reaction mixture, Andersen's sulfinate (1.50 equiv.) dissolved in THF (1.00 M) was added dropwise with a syringe pump (0.50 mL/min) and the reaction mixture was stirred 1 h at −78 °C, then 16 h at room temperature. The reaction mixture was quenched with 2 M HCl (60 mL), the layers separated, and the organics washed with 2 M HCl (4x). The combined aqueous phase was washed with Et_2O (3x), then the combined organics were washed with saturated $NaHCO_3$ and brine, dried over anhydrous Na_2SO_4, filtered and concentrated under reduced pressure.

To facilitate the chromatographic separation of sulfoxide from menthol, silylation of menthol was performed as follows. The crude mixture was stirred 2 h under vacuum, then dissolved in CH_2Cl_2 (20 mL). Triethylamine (1.50 equiv.) was added followed by the dropwise addition of trimethylsilyl chloride TMSCl *or* triethylsilyl chloride TESCl (1.30 equiv.) and the mixture was stirred 3 h at room temperature. The reaction mixture was then diluted with Et_2O and water, the layers separated, and the organics were dried one dried over anhydrous Na_2SO_4, filtered and concentrated under reduced pressure. The crude residue was purified by automated flash column chromatography to afford pure product(s).

Stereochemistry at sulfur determined by (+) or (−)-Andersens sulfinate. Stereochemistry α- to sulfur determined by (+) or (−)-sparteine.

General procedure 2 (GP2) – Sulfoxide homologation

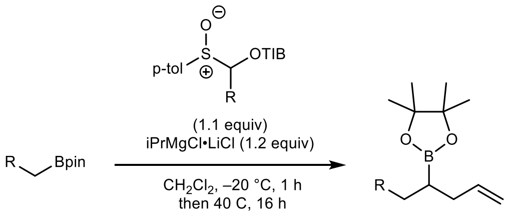

*i*PrMgCl•LiCl (1.14 M in THF, 1.20 equiv.) was added dropwise to a mixture of boronic ester (1.00 equiv.) and sulfoxide (1.10 equiv.) in CH_2Cl_2 (0.20 M with respect to boronic ester) at 0 °C and the resulting solution was stirred 1 h at the same temperature (*pale yellow solution*). After warming to room temperature, the reaction mixture was heated at 40 °C for 3 h (*turbid white solution*). The reaction mixture was then cooled to room temperature and was quenched with saturated aqueous NH_4Cl. The aqueous phase was extracted with Et_2O (3x). The

combined organics were dried over anhydrous Na_2SO_4 and filtered over a short pad of silica deactivated with Et_2O: Et_3N 1% in order to remove the residual TIB acid.

C-B stereochemistry of the product is determined by the stereochemistry in the α position to sulphur of the sulfoxide starting material.

General procedure 3 (GP3) – Hydroboration

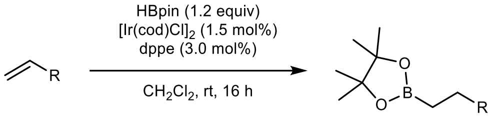

According to a literature procedure[10] Pinacolborane (1.20 equiv. *wrt* alkene) and alkene (1.00 equiv.) were added successively to a solution of $[Ir(cod)Cl]_2$ (0.015 equiv.) and 1,2-bis(diphenylphosphino)ethane dppe (0.030 equiv.) in CH_2Cl_2 (0.30 M) at room temperature. The reaction mixture was then stirred 16 h at room temperature. The reaction was quenched with methanol and extracted with diethyl ether (3x). The organics were dried over $MgSO_4$ and evaporated under reduced pressure. The crude residue was purified by column chromatography to afford pure product.

General procedure 4 (GP4) – Boronic ester oxidation

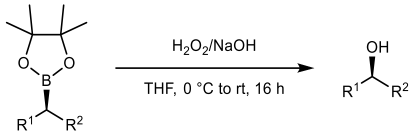

To a solution of boronic ester in THF (0.20 M with respect to boronic ester), a preformed solution of 3 M NaOH and H_2O_2 30% v/v (2:1, total volume equal to that of THF) was added dropwise at 0 °C. The reaction mixture was vigorously stirred at room temperature for 16 h. Sat. aq. $Na_2S_2O_3$ was added dropwise at 0 °C under stirring and the layers were separated. The aqueous phase was extracted with Et_2O (3x). The combined organics were dried over anhydrous Na_2SO_4, filtered and concentrated under reduced pressure.

GP4.1 HPLC samples: The crude residue was analysed by chiral HPLC, further details given in the characterisation section.

GP4.2 Synthetic intermediates: The crude mixture was purified by automated flash column chromatography to afford pure product.

General procedure 5 (GP5) – TES protection of alcohols

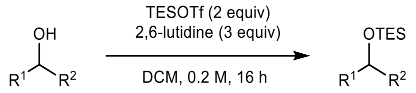

To a suspension of alcohol (1.00 equiv.) in CH_2Cl_2 (0.20 M), 2,6-lutidine (3 equiv. *wrt*. alcohol) was added at room temperature. To the reaction mixture, triethylsilyl trifluoromethanesulfonate TESOTf (2.00 equiv. *wrt*. alcohol) was added dropwise at 0 °C. The resulting solution was stirred 16 h at room temperature. Sat. aq. $NaHCO_3$ was added, and the layers were separated. The aqueous phase was extracted with CH_2Cl_2 (3x). The combined organics were dried over anhydrous Na_2SO_4, filtered and concentrated under reduced pressure. The crude residue was purified by column chromatography to afford pure product.

4 PREPARATION AND CHARACTERISATION DATA

But-3-en-1-yl 2,4,6-triisopropylbenzoate (1)

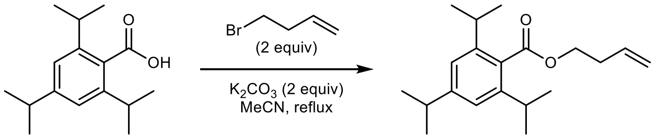

Potassium carbonate (22.3 g, 161 mmol) was added to a stirred solution of 2,4,6-triisopropylbenzoic acid (20.0 g, 80.5 mmol) in MeCN (244 mL, 0.33 M). The mixture was stirred vigorously for 10 min then 4-bromobut-1-ene (21.7 g, 16.3 mL, 161 mmol) was added. The resulting mixture was refluxed for 16 h. The reaction mixture was cooled to room temperature and the undissolved K_2CO_3 filtered off and washed with EtOAc under reduced pressure. The filtrate was evaporated under reduced pressure and the resulting residue was dissolved in EtOAc (200 mL), washed with water (2 x 100 mL) and brine (100 mL) before drying over $MgSO_4$. The crude residue was purified by flash column chromatography to give the title compound as a colourless oil (23.1 g, 95%).

All recorded spectroscopic data matched that previously reported[9]

^{1}H NMR (400 MHz, $CDCl_3$) δ 7.01 (s, 2H, *H*-Ar), 5.84 (ddt, *J* = 17.0, 10.3, 6.7 Hz, 1H, C*H*-alkene), 5.16 (ddt, *J* = 17.2, 1.6, 1.6 Hz, 1H, C*H-trans* alkene), 5.10 (ddt, *J* = 10.2, 1.4, 1.4 Hz, 1H, C*H-cis* alkene), 4.38 (t, *J* = 6.7 Hz, 2H, O-CH_2), 2.88 (m, 3H, C*H*-*i*Pr), 2.50 (m, 2H, CH_2), 1.25 (m, 18H, CH_3-*i*Pr). See spectrum.

^{13}C NMR (101 MHz, $CDCl_3$) δ 171.02, 150.24, 144.93, 134.16, 130.65, 120.99, 117.44, 77.48, 77.16, 76.84, 64.19, 34.57, 33.20, 31.60, 24.29, 24.09. See spectrum.

IR (ν_{max}/cm^{-1}, neat): 2961, 2932, 2870, 1725 (C=O), 1607, 1462, 1379, 1321, 1251, 1142, 1077.

(1*R*)-1-(*p*-Tolylsulfinyl)but-3-en-1-yl 2,4,6-triisopropylbenzoate (S1-*anti*) & (1*S*)-1-(*p*-tolylsulfinyl)but-3-en-1-yl 2,4,6-triisopropylbenzoate (S1-*syn*)

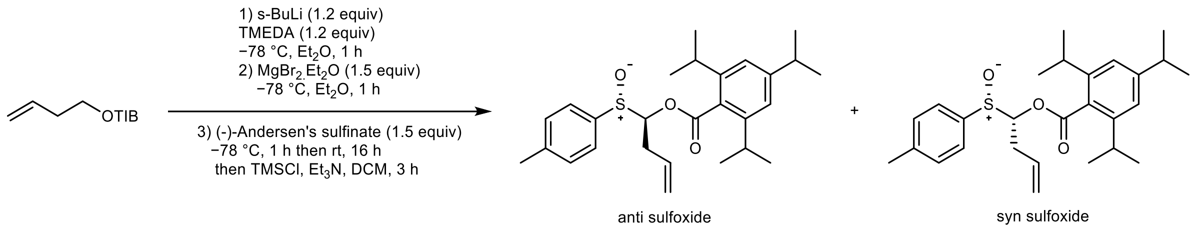

Prepared following **GP1** using alkene **1** (10.0 g, 33.1 mmol), TMEDA (4.61 g, 5.95 mL, 39.7 mmol), *s*-BuLi (1.3 M in hexanes) (30.5 mL, 39.7 mmol), (–)-Andersen's sulfinate (14.6 g, 49.6 mmol), magnesium turnings (3.21 g, 132 mmol), dibromoethane (9.32 g, 4.29 mL, 49.6 mmol), Et_3N (5.02 g, 6.91 mL, 49.6 mmol), trimethylsilyl chloride (4.67 g, 5.45 mL, 43.0 mmol). The crude residue was purified by automated flash column chromatography in two batches (Biotage HC-100 g, hexane: EtOAc 0-20%) to afford the title compounds:

All recorded spectroscopic data matched that previously reported[9]

Less polar ***syn*** (3 g, 21%, >20:1 *d.r.*, ≥99:1 *e.r.*) as a crystalline white solid.

^{1}H NMR (400 MHz, CDCl3) See spectrum.

^{13}C NMR (101 MHz, CDCl3) See spectrum.

$[\alpha]_D^{25}$: 147 (c = 0.6, $CHCl_3$).

M.P.: 86-88 °C

More polar ***anti*** (6.75 g, 46%, >20:1 *d.r.*, ≥99:1 *e.r.*) as a pale orange oil.

^{1}H NMR (400 MHz, CDCl3) See spectrum.

^{13}C NMR (101 MHz, CDCl3) See spectrum.

$[\alpha]_D^{25}$: −8 (c = 1, $CHCl_3$).

(1*S*)-1-(*p*-Tolylsulfinyl)but-3-en-1-yl 2,4,6-triisopropylbenzoate (S1-*syn*)

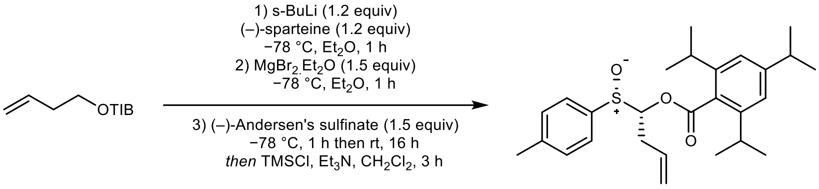

Prepared following **GP1** using alkene **6** (10.0 g, 33.1 mmol), (–)-sparteine (9.30 g, 9.12 mL, 39.7 mmol), *s*-BuLi (1.30 M in hexanes) (30.5 mL, 39.7 mmol), (–)-Andersen's sulfinate (14.6 g, 49.6 mmol), magnesium turnings (3.21 g, 132 mmol), dibromoethane (9.32 g, 4.29 mL, 49.6 mmol), Et_3N (5.02 g, 6.91 mL, 49.6 mmol), trimethylsilyl chloride (4.67 g, 5.45 mL, 43.0 mmol). The crude residue was purified by automated flash column chromatography in two batches (Biotage HC-100 g, hexane: EtOAc 0-20%) to afford a yellow solid. The solid was triturated with pentane at −78 °C and filtered under reduced pressure to give the title compound as a white amorphous solid (5.50 g, 37%, >20:1 *d.r.*, ≥99:1 *e.r.*).

All recorded spectroscopic data matched that previously reported[9]

Both ^{1}H *and* ^{13}C *spectroscopic data was identical to that of syn sulfoxide* ***8-syn*** *obtained in the previous procedure using TMEDA.*

4.1 Fragment 1

(*S*)-3,7-Dimethyloct-6-en-1-yl 2,4,6-triisopropylbenzoate ((3*S*)-S2)

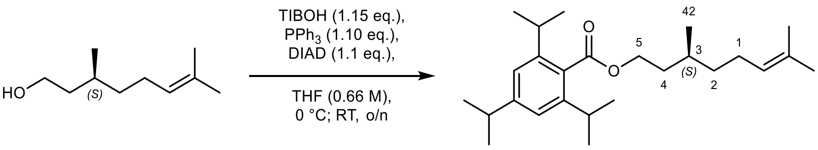

To a stirred suspension of PPh_3 (9.23 g, 35.2 mmol, 1.10 equiv.) and DIAD (7.11 g, 35.2 mmol, 1.10 equiv.) in THF (64.0 mL, 0.50 M) at 0 °C (ice bath), (*S*)-citronellol (1 equiv.) and 2,4,6-triisopropylbenzoic acid (9.14 g, 36.8 mmol) were added. The reaction mixture was stirred at rt for 16 h. The volatiles were removed *in vacuo* and the crude residue was partitioned between diethyl ether and sat. aq. $NaHCO_3$. The organic layer was then washed with water and brine and dried over $MgSO_4$. The crude mixture was purified by normal phase flash column chromatography (hexane: EtOAc 9:1) to afford the title compound (12.1 g, 98% yield) as a colourless oil.

^{1}H NMR (400 MHz, CDCl3) δ 7.01 (s, 2H, *H*-Ar), 5.13 – 5.05 (m, 1H, C*H*-alkene), 4.42 – 4.29 (m, 2H, *H*-5), 2.96 – 2.78 (m, 3H, C*H*-*i*Pr), 2.10 – 1.89 (m, 2H, *H*-1), 1.84 – 1.73 (m, 1H, *H*-4'), 1.67 (d, *J* = 1.3 Hz, 3H, CH_3-*trans* alkene), 1.66 – 1.60 (m, 1H, *H*-3), 1.59 (s, 3H, CH_3-*cis* alkene), 1.58 – 1.47 (m, 1H, *H*-4''), 1.45 – 1.33 (m, 1H, *H*-2'), 1.25 (d, *J* = 6.8 Hz, 18H, CH_3-*i*Pr), 1.23 – 1.15 (m, 1H, *H*-2''), 0.95 (d, *J* = 6.5 Hz, 3H, *H*-42). See spectrum.

^{13}C NMR (101 MHz, CDCl3) δ 171.19 (*C*O-benzoate), 150.17 (*C*-*para*), 144.86 (*C*-*ortho*), 131.50 (*C*-alkene), 130.88 (*C*-*ipso*), 124.68 (*C*H-alkene), 120.97 (*C*H-*meta*), 63.49 (CH_2-5), 37.16 (CH_2-2), 35.74 (CH_2-4), 34.58 (*C*H-*i*Pr *para*), 31.64 (*C*H-*i*Pr *ortho*), 29.61 (*C*H-3), 25.84 (CH_3-*trans* alkene), 25.57 (CH_2-1), 24.29 (CH_3-*i*Pr *ortho*), 24.10 (CH_3-*i*Pr *para*), 19.35 (CH_3-42), 17.75 (CH_3-*cis* alkene). See spectrum.

HRMS (m/z): (ESI) calculated for $C_{26}H_{42}O_2$ [M+H]+ 387.3258, found 387.3268.

TLC: $\boldsymbol{R_f}$ = 0.46 (95:5 hexane/EtOAc, stained with ceric ammonium molybdate (CAM))

IR (ν_{max}/cm^{-1}, neat): 2962, 2928, 2871, 1727 (C=O), 1607, 1461, 1252, 1076.

$[\alpha]_D^{25}$: −4 (*c* = 1, $CHCl_3$).

(*S*)-6-Hydroxy-3-methylhexyl 2,4,6-triisopropylbenzoate ((3*S*)-S3)

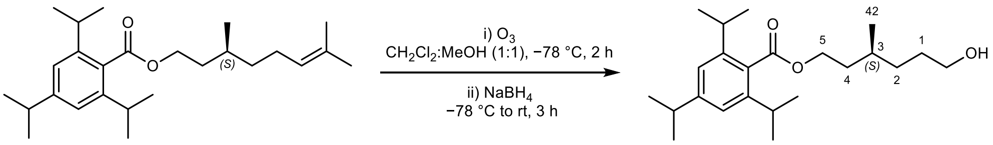

Ozone from a generator was bubbled through a solution of alkene **S2** (12.37 g, 32.0 mmol, 1 equiv.) in CH_2Cl_2:MeOH (1:1, 160 mL, 0.20 M) and a small amount of Sudan III red stain (< 5 mg) at −78 °C for approximately 2 h until the solution turned blue. Oxygen was then bubbled through the solution at −78 °C until the blue colour disappeared. Sodium borohydride (6.05 g, 160 mmol, 5.00 equiv.) was added at −78 °C, and the reaction mixture was allowed to warm to room temperature and stirred for 3 h. The reaction mixture was concentration under reduced pressure and the resulting residue partitioned between sat. aq. $NaHCO_3$ and ether. The aqueous layer was extracted with Et_2O (3x). The combined organic layers were washed with brine, and were dried over $MgSO_4$. The crude mixture was purified by normal phase flash column chromatography (pentane: Et_2O 4:1) to afford the title compound (8.52 g, 76%) as a colourless oil.

^{1}H NMR (400 MHz, $CDCl_3$) δ 7.00 (s, 2H, *H*-Ar), 4.43 – 4.27 (m, 2H, *H*-5), 3.70 – 3.55 (m, 2H, *H*-1), 2.94 – 2.78 (m, 3H, C*H*-*i*Pr), 2.20 (br. s, 1H, O*H*), 1.84 – 1.72 (m, 1H, *H*-4'), 1.70 – 1.59 (m, 2H, *H*-3 & *H*1'), 1.59 – 1.48 (m, 2H, *H*1'' & *H*4''), 1.48 – 1.37 (m, 1H, *H*2'), 1.24 (d, *J* = 6.9 Hz, 19H, CH_3-*i*Pr & *H*2''), 1.00 – 0.93 (m, 3H, *H*-42). See spectrum.

^{13}C NMR (101 MHz, $CDCl_3$) δ 171.18 (*C*O-benzoate), 150.20 (*C*-*para*), 144.84 (*C*-*ortho*), 130.77 (*C*-*ipso*), 120.97 (*C*H-*meta*), 63.35 (*C*H_2-5), 63.30 (*C*H_2OH), 35.75 (*C*H_2-4), 34.55 (*C*H-*i*Pr *para*), 32.99 (*C*H_2-2), 31.64 (*C*H-*i*Pr *ortho*), 30.26 (*C*H_2-1), 29.78 (*C*H-3), 24.27 (*C*H_3-*i*Pr *ortho*), 24.08 (*C*H_3-*i*Pr *para*), 19.37 (*C*H_3-42). See spectrum.

HRMS (m/z): (ESI) calculated for $C_{26}H_{42}O_2$ [M+H]+ 385.2713, found 385.2727.

TLC: R_f = 0.07 (85:15 hexane/EtOAc, stained with ceric ammonium molybdate (CAM))

IR (ν_{max}/cm^{-1}, neat): 3424 (OH), 2960, 2930, 2870, 1724 (C=O), 1606, 1461, 1251, 1076.

$[\alpha]_D^{25}$: 4 (*c* = 0.5, $CHCl_3$).

(*S*)-6-Iodo-3-methylhexyl 2,4,6-triisopropylbenzoate ((3*S*)-S4)

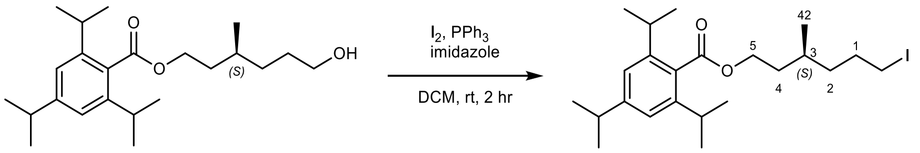

Iodine (7.74 g, 30.5 mmol, 1.30 equiv.) was added portionwise to triphenylphosphine (7.99 g, 30.5 mmol, 1.30 equiv.), imidazole (2.39 g, 35.2 mmol, 1.50 equiv.) and the alcohol (8.50 g, 23.4 mmol, 1.00 equiv.) in CH_2Cl_2 (117 mL, 0.20 M), and the mixture was stirred at rt for 2 h. Sat. aq. sodium thiosulfate and Et_2O were added, and the organic later was washed with water and brine. The organic layer was dried over $MgSO_4$. The crude mixture was purified by normal phase flash column chromatography (pentane: Et_2O 9:1) to afford the title compound (8.73 g, 79%) as a colourless oil.

^{1}H NMR (400 MHz, $CDCl_3$) δ 7.01 (s, 2H, *H*-Ar), 4.42 – 4.28 (m, 2H, *H*-5), 3.22 – 3.11 (m, 2H, CH_2I), 2.87 (m, 3H, C*H*-*i*Pr), 1.95 – 1.71 (m, 3H, *H*-1 & *H*-4'), 1.71 – 1.61 (m, 1H, *H*3), 1.61 – 1.38 (m, 2H, *H*4'' & *H*2'), 1.35 – 1.27 (m, 1H, *H*2''), 1.25 (d, *J* = 6.9 Hz, 18H, CH_3-*i*Pr), 0.96 (d, *J* = 6.5 Hz, 3H, *H*42). See spectrum.

^{13}C NMR (101 MHz, $CDCl_3$) δ 171.12 (*C*O-benzoate), 150.23 (*C*-*para*), 144.84 (*C*-*ortho*), 130.74 (*C*-*ipso*), 120.99 (*C*H-*meta*), 63.18 (*C*H_2-5), 37.84 (*C*H_2-2), 35.65 (*C*H_2-4), 34.56

(*C*H-*i*Pr *para*), 31.66 (*C*H-*i*Pr *ortho*), 31.23 (*C*H_2-1), 29.28 (*C*H-3), 24.30 (*C*H_3-*i*Pr *ortho*), 24.09 27 (*C*H_3-*i*Pr *para*), 19.38 (*C*H_3-42), 7.09 (*C*H_2I). See spectrum.

HRMS (m/z): (ESI) calculated for $C_{23}H_{37}IO_2$ [M+H]+ 473.1911, found 473.1903.

TLC: $\boldsymbol{R_f}$ = 0.34 (95:5 hexane/EtOAc, stained with ceric ammonium molybdate (CAM))

IR (ν_{max}/cm^{-1}, neat): 2959, 2927, 2869, 1722 (C=O), 1606, 1460, 1250, 1074, 504 (C-I).

$[\boldsymbol{\alpha}]_{\mathbf{D}}^{\mathbf{25}}$: -4 (*c* = 1, $CHCl_3$).

(*S*)-3-Methylhex-5-en-1-yl 2,4,6-triisopropylbenzoate ((3*S*)-S5)

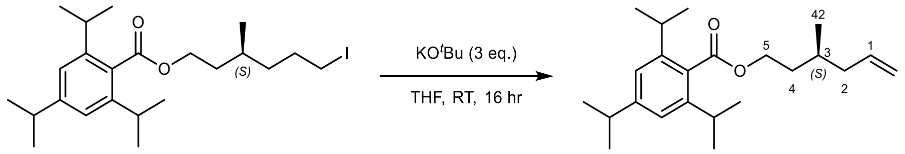

Iodide **3*S*-S4** (8.70 g, 18.4 mmol, 1.00 equiv.) was dissolved in THF (92 mL, 0.20 M), potassium tert-butoxide (3.00 equiv.) was added, and the reaction mixture was stirred at rt for 16 h. The reaction mixture was partitioned between water and Et_2O. The organic layer was washed with brine and dried over $MgSO_4$. The crude mixture was purified by normal phase flash column chromatography (pentane: Et_2O 9:1) to afford the title compound (5.30 g, 84%) as an orange oil.

^{1}H NMR (400 MHz, $CDCl_3$) δ 7.01 (s, 2H, *H*-Ar), 5.85 – 5.69 (m, 1H, *H*-1), 5.06 – 5.01 (m, 1H, C*H-cis* alkene), 5.00 (m, 1H C*H-trans* alkene), 4.43 – 4.28 (m, 2H, *H*-5), 2.95 – 2.79 (m, 3H, C*H*-*i*Pr), 2.17 – 2.07 (m, 1H, *H*-2'), 2.01 – 1.91 (m, 1H, *H*-2''), 1.86 – 1.76 (m, 1H, *H*-4'), 1.76 – 1.67 (m, 1H, *H*-3), 1.60 – 1.48 (m, 1H, *H*-4''), 1.25 (d, *J* = 6.9 Hz, 18H, CH_3-*i*Pr), 0.95 (d, *J* = 6.6 Hz, 3H, *H*-42). See spectrum.

^{13}C NMR (101 MHz, $CDCl_3$) δ 171.15 (*C*O-benzoate), 150.19 (*C-para*), 144.86 (*C-ortho*), 136.89 (*C*H-1, 130.82 (*C-ipso*), 120.98 (*C*H-*meta*), 116.34 (*C*H_2- alkene), 63.31 (*C*H_2-5), 41.35 (*C*H_2-2), 35.22 (*C*H_2-4), 34.57 (*C*H-*i*Pr *para*), 31.64 (*C*H-*i*Pr *ortho*), 29.79 (*C*H-3), 24.29 (*C*H_3-*i*Pr *ortho*), 24.10 ((*C*H_3-*i*Pr *para*), 19.28 (*C*H_3-42). See spectrum.

HRMS (m/z): (ESI) calculated for $C_{23}H_{36}O_2$ [M+H]+ 345.2788, found 345.2801.

TLC: $\boldsymbol{R_f}$ = 0.37 (95:5 hexane/EtOAc, stained with ceric ammonium molybdate (CAM))

IR (ν_{max}/cm^{-1}, neat): 2961, 2928, 2871, 1726 (C=O), 1461, 1251, 1033.

$[\alpha]_D^{25}$: 0 (c = 1, $CHCl_3$).

(1R,3S)-3-Methyl-1-(p-tolylsulfinyl)hex-5-en-1-yl 2,4,6-triisopropylbenzoate ((3*S*)-6)

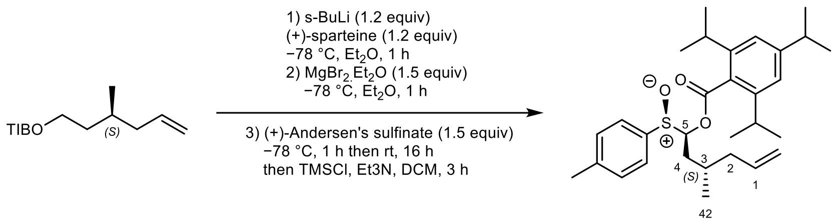

Prepared following General Procedure 1 using alkene **(3*S*)-S5** (2.50 g, 7.30 mmol), (+)-sparteine (2.04 g, 2.00 mL, 8.70 mmol), *s*-BuLi (1.30 M in hexanes) (6.70 mL, 8.70 mmol), (+)-Andersen's sulfinate (3.20 g, 10.9 mmol), magnesium turnings (0.71 g, 29.0 mmol), dibromoethane (2.04 g, 0.94 mL, 10.9 mmol), Et_3N (1.10 g, 1.52 mL, 10.9 mmol), trimethylsilyl chloride (1.02 g, 1.20 mL, 9.40 mmol). The crude residue was purified by automated flash column chromatography (Biotage HC-50 g, *n*-hexane: EtOAc 0-20%) to afford the title compound (1.85 g, 53%, >99:1 *dr*) as a white solid.

^{1}H NMR (400 MHz, $CDCl_3$) δ 7.64 (d, *J* = 8.2 Hz, 2H, *H*-Ar), 7.37 (d, *J* = 7.8 Hz, 2H, *H*-Ar), 7.05 (s, 2H, *H*-Ar), 5.77 (dd, *J* = 10.5, 2.6 Hz, 1H, *H*-5), 5.49 (dddd, *J* = 16.9, 10.2, 7.6, 6.6 Hz, 1H, *H*-1), 4.84 (ddt, *J* = 10.1, 2.1, 1.0 Hz, 1H, C*H*-*cis* alkene), 4.77 (dq, *J* = 17.0, 1.5 Hz, 1H, C*H*-*trans* alkene), 2.94 (dp, *J* = 23.4, 6.9 Hz, 3H, C*H*-*i*Pr), 2.44 (s, 3H, CH_3-tolyl), 2.01 – 1.93 (m, 1H, *H*-4'), 1.89 (ddd, *J* = 14.4, 10.5, 3.9 Hz, 1H, *H*-2'), 1.68 (m, 1H, *H*-4''), 1.64 – 1.55 (m, 1H, *H*-3), 1.50 (ddd, *J* = 14.6, 9.4, 2.7 Hz, 1H, *H*-2''), 1.31 (d, *J* = 6.8 Hz, 6H, CH_3-*i*Pr), 1.27 (d, *J* = 7.1 Hz, 12H, CH_3-*i*Pr), 0.86 (d, *J* = 6.6 Hz, 3H, *H*-42). See spectrum.

^{13}C NMR (101 MHz, $CDCl_3$) δ 170.68 (*C*O-benzoate), 151.02 (*C*H-TIB-*para*), 145.38 (*C*H-TIB-*ortho*), 141.58 (*C*-tolyl-*ipso*), 137.62 (*C*-tolyl-*para*), 135.43 (*C*H-1), 130.11 (*C*H-tolyl-*meta*), 128.99 (*C*H-TIB-*ipso*), 124.33 (*C*H-tolyl-*ortho*), 121.17 (TIB-*C*H-*meta*), 116.85 (*C*H_2-alkene), 91.40 (*C*H-5), 39.59 (*C*H_2-4), 34.60 (*C*H-*i*Pr *para*), 31.83 (*C*H-*i*Pr *ortho*), 29.02 (*C*H_2-2), 28.60 (*C*H-3), 24.59 (*C*H_3-*i*Pr *ortho*), 24.30 (*C*H_3-*i*Pr *ortho*), 24.06 (*C*H_3-*i*Pr *para*), 24.05 (*C*H_3-*i*Pr *para*), 21.57 (*C*H_3-tolyl), 20.28 (*C*H_3-42). See spectrum.

HRMS (m/z): (ESI) calculated for $C_{30}H_{42}O_3S$ [M+H]+ 505.2747, found 505.2743.

TLC: *R*$_f$ (major) = 0.46, ***R*$_f$** minor = 0.42 (85:15 hexane/EtOAc, stained with *p*-anisaldehyde)

IR (νmax/cm−1, neat): 3076, 2961, 2927, 2871, 1732 (C=O), 1606, 1461, 1364, 1234, 1046 (S=O).

$[\alpha]_D^{25}$: −110 (c = 1, $CHCl_3$).

(*R*)-3,7-Dimethyloct-6-en-1-yl 2,4,6-triisopropylbenzoate ((3*R*)-S2)

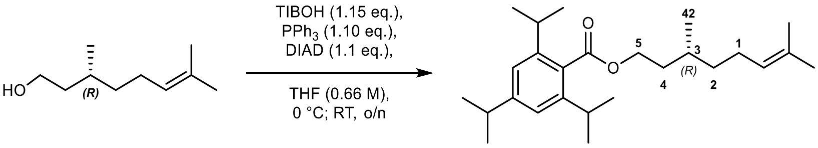

Prepared following the same procedure as described for (3*S*) enantiomer **(3*S*)-S2** using PPh_3 (9.23 g, 35.2 mmol), DIAD (7.11 g, 35.2 mmol), (S)-Citronellol (5.00 g, 32.0 mmol) and THF (64 mL). The crude mixture was purified by normal phase flash column chromatography (hexane: EtOAc 9:1) to afford the title compound (12.1 g, 98% yield) as a colourless oil.

Both ^1H and ^{13}C spectroscopic data was identical to that of it's enantiomer **(3*S*)-S2**.

HRMS (m/z): (ESI) calculated for $C_{26}H_{42}O_2$ [M+H]+ 387.3258, found 387.3258.

IR (νmax/cm−1, neat): 2960, 2926, 2870, 1724 (C=O), 1607, 1461, 1250, 1074.

$[\alpha]_D^{25}$: 3 (c = 0.8, $CHCl_3$).

(*R*)-6-Hydroxy-3-methylhexyl 2,4,6-triisopropylbenzoate ((3*R*)-S3)

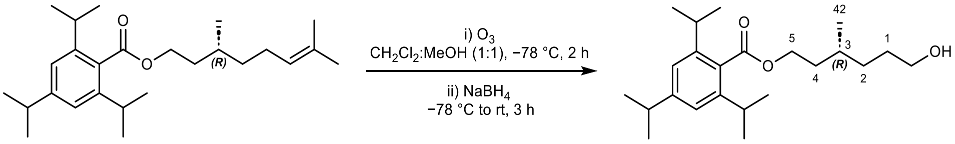

Prepared following the same procedure as described for (3*S*) enantiomer **(3*S*)-S3** using alkene **(*R*)-63** (12.1 g, 31.3 mmol), CH_2Cl_2:MeOH (1:1, 160 mL), sodium borohydride (5.92 g, 156 mmol). The crude mixture was purified by normal phase flash column chromatography (pentane: Et_2O 4:1) to afford the title compound (6.90 g, 61%) as a colourless oil.

Both ^1H and ^{13}C spectroscopic data was identical to that of it's enantiomer **(3*S*)-S3**.

HRMS (m/z): (ESI) calculated for $C_{26}H_{42}O_2$ [M+Na]+ 385.2713, found 385.2722.

IR (νmax/cm−1, neat): 3425 (OH), 2960, 2930, 2870, 1723 (C=O), 1606, 1461, 1250, 1075.

$[\alpha]_D^{25}$: -4 (c = 1, $CHCl_3$).

(*R*)-6-Iodo-3-methylhexyl 2,4,6-triisopropylbenzoate ((3*R*)-S4)

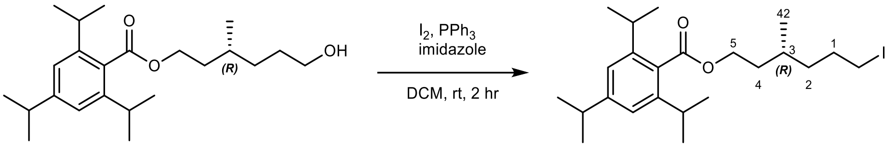

Prepared following the same procedure as described for (3*S*) enantiomer **(3*S*)-S4** using iodine (6.19g, 24.4 mmol), PPh_3 (6.40 g, 24.4 mmol), imidazole (1.92 g, 28.1 mmol) and alcohol **(*R*)-64** (6.80 g, 18.8 mmol) in CH_2Cl_2 (94 mL). The crude mixture was purified by normal phase flash column chromatography (pentane: Et_2O 9:1) to afford the title compound (6.20 g, 70%) as a colourless oil.

Both ^1H and ^{13}C spectroscopic data was identical to that of it's enantiomer **(3*S*)-S4**.

HRMS (m/z): (ESI) calculated for $C_{23}H_{37}IO_2$ [M+H]+ 473.1911, found 473.1909.

IR (ν_{max}/cm^{-1}, neat): 2961, 2929, 2870, 1725 (C=O), 1607, 1461, 1251, 1076, 504 (C-I).

$[\alpha]_D^{25}$: 0 (c = 1, $CHCl_3$).

(*R*)-3-Methylhex-5-en-1-yl 2,4,6-triisopropylbenzoate ((3*R*)-S5)

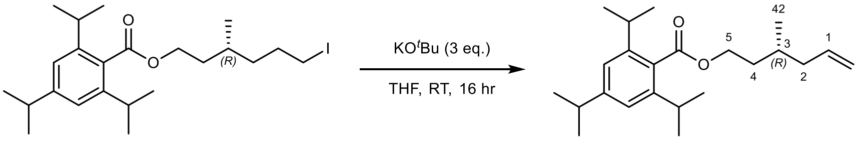

Prepared following the same procedure as described for (3*S*) enantiomer **(3*S*)-S5** using iodide **(*R*)-65** (6.10 g, 12.9 mmol), potassium tert-butoxide (4.35 g, 38.7 mmol), in THF (65 mL). The crude mixture was purified by normal phase flash column chromatography (pentane: Et_2O 9:1) to afford the title compound (4.16 g, 94%) as an orange oil.

Both ^1H and ^{13}C spectroscopic data was identical to that of it's enantiomer **(3*S*)-S5**.

HRMS (m/z): (EI) calculated for $C_{23}H_{36}O_2$ [M]+ 344.2710, found 344.2705.

IR (ν_{max}/cm^{-1}, neat): 2963, 2926, 2870, 1725 (C=O), 1607, 1462, 1380, 1319, 1251, 1143, 1076.

$[\alpha]_D^{25}$: 0 (c = 1, $CHCl_3$).

(1R,3R)-3-methyl-1-(p-tolylsulfinyl)hex-5-en-1-yl 2,4,6-triisopropylbenzoate ((3*R*)-6)

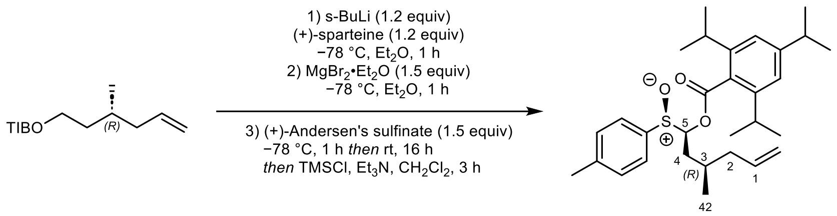

Prepared following the same procedure as described for (3*S*) diastereomer **(3*S*)-6** using alkene **(3*S*)-S5** (3.00 g, 8.70 mmol), (+)-sparteine (2.45 g, 2.40 mL, 10.4 mmol), *s*-BuLi (1.30 M in hexanes) (8.00 mL, 10.4 mmol), (+)-Andersen's sulfinate (3.85 g, 13.1 mmol), magnesium turnings (0.85 g, 34.8 mmol), dibromoethane (2.45 g, 1.13 mL, 13.1 mmol), Et_3N (1.32 g, 1.82 mL, 11.3 mmol), trimethylsilyl chloride (1.23 g, 1.82 mL, 11.3 mmol). The crude residue was purified by automated flash column chromatography (Biotage HC-50 g, *n*-hexane: EtOAc 0-20%) to afford the title compound (2.04 g, 48%, >99:1 *dr*) as a white solid.

^{1}H NMR (500 MHz, $CDCl_3$) δ 7.56 (d, *J* = 8.2 Hz, 2H, *H*-Ar), 7.32 (d, *J* = 8.1 Hz, 2H, *H*-Ar), 7.03 (s, 2H, *H*-Ar), 6.14 (dd, *J* = 9.2, 3.3 Hz, 1H, *H*-5), 5.64 (ddt, *J* = 17.2, 10.3, 7.1 Hz, 1H, *H*-1), 5.01 – 4.92 (m, 2H, CH_2- alkene), 2.96 – 2.79 (m, 3H, C*H*-*i*Pr), 2.42 (s, 3H, CH_3-tolyl), 2.21 – 2.12 (m, 1H, *H*-2'), 1.86 (ddd, *J* = 13.7, 7.4, 7.4 Hz, 1H, *H*-2''), 1.78 – 1.67 (m, 2H, *H*-4', *H*-3), 1.44 – 1.36 (m, 1H, *H*-4''), 1.30 – 1.17 (m, 18H, CH_3-*i*Pr), 0.92 (d, *J* = 6.3 Hz, 3H, *H*-42). See spectrum.

^{13}C NMR (126 MHz, $CDCl_3$) δ 169.36 (*C*O-benzoate), 150.80 (*C*H-TIB-*para*), 145.35 (*C*H-TIB-*ortho*), 142.18 (*C*-tolyl-*ipso*), 136.70 (*C*-tolyl-*para*), 135.62 (*C*H-1), 129.98 (*C*H-tolyl-*meta*), 129.09 (*C*H-TIB-*ipso*), 125.78 (*C*H-tolyl-*ortho*), 121.13 (TIB-*C*H-*meta*), 117.04 (*C*H_2-alkene), 87.02 (*C*H-5), 39.85 (*C*H_2-4), 34.51 (*C*H-*i*Pr *para*), 34.28 (*C*H_2-2), 31.65 (*C*H-*i*Pr *ortho*), 29.22 (*C*H-3), 24.69 (*C*H_3-*i*Pr *ortho*), 24.23 (*C*H_3-*i*Pr *ortho*), 24.02 (*C*H_3-*i*Pr *para*), 24.01 (*C*H_3-*i*Pr *para*), 21.59 (*C*H_3-tolyl), 20.06 (*C*H_3-42). See spectrum.

HRMS (m/z): (ESI) calculated for $C_{30}H_{42}O_3S$ [M+Na]+ 505.2747, found 505.2763.

TLC: ***R*f** (major) = 0.44, ***R*f** minor = 0.40 (85:15 hexane/EtOAc, stained with *p*-anisaldehyde)

IR (ν_{max}/cm^{-1}, neat): 2962, 2926, 2870, 1737 (C=O), 1604, 1459, 1383, 1230, 1040 (S=O).

$[\alpha]_D^{25}$: -26 (*c* = 1, $CHCl_3$).

4-(4,4,5,5-Tetramethyl-1,3,2-dioxaborolan-2-yl)butyl 2,4,6-triisopropylbenzoate (2)

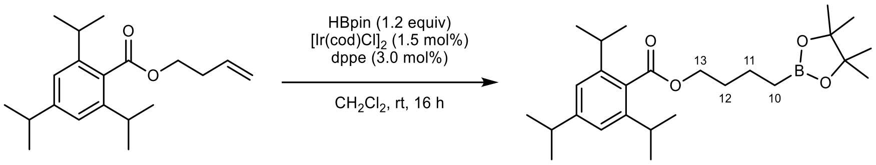

Prepared following General Procedure 3 using pinacolborane (3.05 g, 3.45 mL, 23.8 mmol), alkene **1** (6.00 g, 19.8 mmol), [Ir(cod)Cl]$_2$ (200 mg, 0.298 mmol) and 1,2-bis(diphenylphosphino)ethane dppe (237 mg, 0.595 mmol). The crude mixture was purified by normal phase flash column chromatography (4% Et_2O in pentane) to afford the title compound (6.30 g, 74%) as a colourless oil.

^{1}H NMR (400 MHz, $CDCl_3$) δ 7.00 (s, 2H, *H*-Ar), 4.29 (t, *J* = 6.7 Hz, 2H, *H*-13), 2.87 (m, 3H, C*H*-*i*Pr), 1.79 – 1.69 (m, 2H, *H*-12), 1.58 – 1.48 (m, 2H, *H*-11), 1.24 (d, *J* = 6.9 Hz, 30H, CH_3-*i*Pr & CH_3-Bpin), 0.82 (t, *J* = 7.8 Hz, 2H, *H*-10). See spectrum.

^{13}C NMR (101 MHz, $CDCl_3$) δ 171.01 (*C*O-benzoate), 149.97 (*C*H-TIB-*para*), 144.74 (*C*H-TIB-*ortho*), 130.78 (*C*H-TIB-*ipso*), 120.81 (*C*H-TIB-*meta*), 82.99 (*C*-Bpin), 64.88 (*C*H_2-13), 34.43 (*C*H-*i*Pr *para*), 31.47 (*C*H-*i*Pr *ortho*), 31.05 (*C*H_2-12), 24.81 (*C*H_3-Bpin *or* *C*H_3-*i*Pr), 24.15 (*C*H_3-Bpin *or* *C*H_3-*i*Pr), 23.96 (*C*H_3-Bpin *or* *C*H_3-*i*Pr), 20.53 (*C*H_2-11). See spectrum.

carbon next to boron (*C*H_2-10) *not observed due to quadrupolar relaxation*

HRMS (m/z): (ESI) calculated for $C_{26}H_{43}BO_4$ [M+H]+ 453.3147, found 453.3167.

TLC: ***R*$_f$** = 0.5 (85:15 hexane/EtOAc, stained with *p*-anisaldehyde).

IR (ν_{max}/cm^{-1}, neat): 2961, 2932, 2870, 1724 (C=O), 1607, 1462, 1379, 1321, 1250, 1139, 1076, 968, 876.

(*R*)-5-(4,4,5,5-Tetramethyl-1,3,2-dioxaborolan-2-yl)oct-7-en-1-yl 2,4,6-triisopropylbenzoate (3)

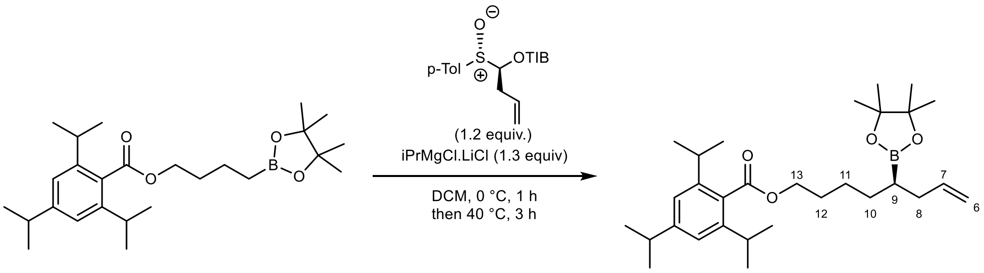

Prepared following General Procedure 2 using boronic ester **2** (1.00 g, 2.32 mmol), anti sulfoxide **S1-*anti*** (1.23 g, 2.79 mmol), *i*PrMgCl·LiCl (1.2 M in THF, 2.52 mL, 3.02 mmol). The crude mixture was purified by normal phase flash column chromatography (Biotage HC-50 g, *n*-hexane: EtOAc 0-30%) to afford the title compound (0.949 g, 84%) as a colourless oil.

^{1}H NMR (400 MHz, $CDCl_3$) δ 6.99 (s, 2H, *H*-Ar), 5.79 (ddt, *J* = 17.0, 10.1, 6.9 Hz, 1H, *H*-7), 5.04 – 4.96 (m, 1H, *H*-6 *cis*), 4.93 (ddt, *J* = 10.1, 2.2, 1.1 Hz, 1H, *H*-6 *trans*), 4.28 (t, *J* = 6.8 Hz, 2H, *H*-13), 2.86 (dp, *J* = 13.6, 6.8 Hz, 3H, C*H*-*i*Pr), 2.22 – 2.06 (m, 2H, *H*-8), 1.79 – 1.64 (m, 2H, *H*-12), 1.50 – 1.34 (m, 4H, *H*-10 & *H*-11), 1.28 – 1.18 (m, 30H, CH_3-*i*Pr & CH_3--Bpin), 1.07 (dd, *J* = 10.6, 6.5 Hz, 1H, *H*-9). See spectrum.

^{13}C NMR (101 MHz, $CDCl_3$) δ 171.16 (*C*O-benzoate), 150.13 (*C*H-TIB-*para*), 144.86 (*C*H-TIB-*ortho*), 138.61 (*C*H-7), 130.88 (*C*H-TIB-*ipso*), 120.94 (*C*H-TIB-*meta*), 115.06 (*C*H_2-6), 83.17 (*C*-Bpin), 65.17 (*C*H_2-13), 35.53 (*C*H_2-8), 34.58 (*C*H-*i*Pr *para*), 31.62 (*C*H-*i*Pr *ortho*), 30.49 (*C*H_2-10), 29.01 (*C*H_2-12), 25.68 (*C*H_2-11), 24.94 (*C*H_3-Bpin *or* CH_3-*i*Pr), 24.31 (*C*H_3-Bpin *or* CH_3-*i*Pr), 24.11 (*C*H_3-Bpin *or* CH_3-*i*Pr). See spectrum.

carbon next to boron (*C*H-9) not *observed due to quadrupolar relaxation*

HRMS (m/z): (MALDI) calculated for $C_{30}H_{49}BO_4$ [M+Na]+ 507.3621, found 507.3630.

TLC: ***R*f** = 0.43 (85:15 hexane/EtOAc, stained with ceric ammonium molybdate (CAM))

IR (ν_{max}/cm^{-1}, neat): 2962, 2929, 2869, 1726 (C=O), 1607, 1462, 1383, 1320, 1251, 1142, 1075.

$[\alpha]_D^{25}$: −9 (*c* = 0.9, $CHCl_3$).

(*S*)-5,8-bis(4,4,5,5-Tetramethyl-1,3,2-dioxaborolan-2-yl)octyl 2,4,6-triisopropylbenzoate (4)

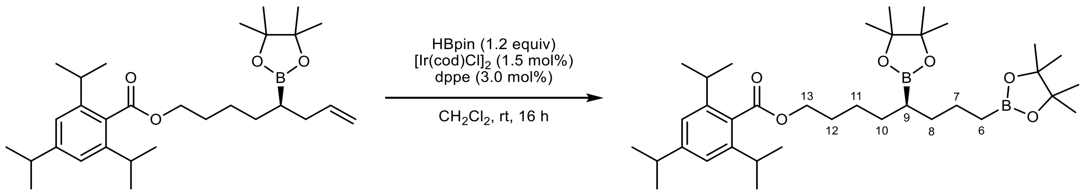

Prepared following General Procedure 3 using pinacolborane (0.539 g, 0.611 mL, 4.21 mmol), alkene **3** (1.70 g, 3.51 mmol), $[Ir(cod)Cl]_2$ (35.0 mg, 52.6 µmol) and 1,2-bis(diphenylphosphino)ethane dppe (41.9 mg, 0.105 mmol). The crude mixture was purified

by normal phase flash column chromatography (Biotage HC-50 g, *n*-hexane: EtOAc 0-20%) to afford the title compound (1.93 g, 90%) as a colourless oil.

^{1}H NMR (400 MHz, CDCl$_3$) δ 6.99 (s, 2H, *H*-Ar), 4.31 – 4.22 (m, 2H, *H*-13), 2.85 (dh, *J* = 13.6, 6.8 Hz, 3H, C*H*-*i*Pr), 1.78 – 1.64 (m, 2H, *H*-12), 1.49 – 1.30 (m, 8H, *H*-7, *H*-8, *H*-10, *H*-11), 1.27 – 1.17 (m, 42H, C*H*$_3$-*i*Pr & C*H*$_3$-Bpin), 0.95 (m, 1H, *H*-9), 0.75 (t, *J* = 7.1 Hz, 2H, *H*-6). See spectrum.

^{13}C NMR (101 MHz, CDCl$_3$) δ 171.03 (*C*O-benzoate), 149.95 (*C*H-TIB-*para*), 144.72 (*C*H-TIB-*ortho*), 130.78 (*C*H-TIB-*ipso*), 120.78 (*C*H-TIB-*meta*), 82.81 (*C*-Bpin), 82.79 (*C*-Bpin), 65.11 (*C*H$_2$-13), 34.44 (*C*H-*i*Pr *para*), 34.00 (*C*H$_2$-8), 31.46 (*C*H-*i*Pr *ortho*), 30.75 (*C*H$_2$-10), 28.95 (*C*H$_2$-12), 25.68 (*C*H$_2$-11), 24.82 (*C*H$_3$-Bpin *or* *C*H$_3$-*i*Pr), 24.79 (*C*H$_3$-Bpin *or* *C*H$_3$-*i*Pr), 24.76 (*C*H$_3$-Bpin *or* *C*H$_3$-*i*Pr), 24.16 (*C*H$_3$-Bpin *or* *C*H$_3$-*i*Pr), 23.97 (*C*H$_3$-Bpin *or* *C*H$_3$-*i*Pr), 23.61 (*C*H$_2$-7). See spectrum.

carbon next to boron (*C*H-9 & *C*H$_2$-6) not *observed due to quadrupolar relaxation*

HRMS (m/z): (MALDI) calculated for $C_{36}H_{62}B_2O_6$ [M+Na]+ 635.4637, found 635.4344.

TLC: *R*$_f$ = 0.27 (90:10 hexane/EtOAc, stained with *p*-anisaldehyde).

IR (ν_{max}/cm^{-1}, neat): 296, 2929, 2869, 1725 (C=O), 1607, 1461, 1371, 1318, 1251, 1144, 1076, 968, 876.

$[\alpha]_D^{25}$: 6 (*c* = 1, $CHCl_3$).

(5R,9R,11S)-11-methyl-5,9-bis((triethylsilyl)oxy)tetradec-13-en-1-yl 2,4,6-triisopropylbenzoate ((3*S*)-F1)

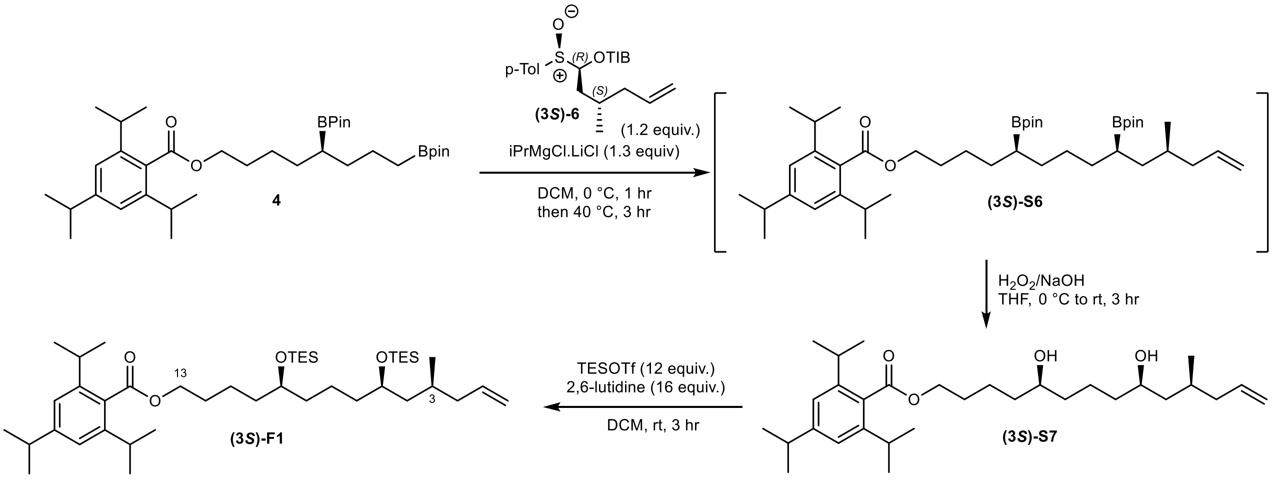

Prepared following General Procedure **2** using boronic ester **4** (1.00 g, 1.63 mmol), sulfoxide **(3*S*)-6** (945 mg, 1.96 mmol), *i*PrMgCl·LiCl (1.20 M in THF, 1.77 mL, 2.12 mmol). The reaction mixture was filtered through silica treated and eluted with 1% triethylamine in diethyl ether. The filtrate was evaporated *in vacuo* and the crude boronic ester **(3*S*)-S6** oxidised according to General Procedure **4**. The crude mixture was purified by normal phase column chromatography (Biotage 25 g pentane: Et_2O, 0-50%) to afford a mixture of diol **(3*S*)-S7** and pinacol. The mixture was then TES protected according to General Procedure **5**, using triethylsilyl triflate (5.18 g, 4.43 mL, 19.6 mmol), 2,6-lutidine (2.80 g, 3.03 mL, 26.1 mmol). The crude mixture was purified by normal phase flash column chromatography (Biotage 50 g pentane: Et_2O, 0-10%) to afford the title compound (1.00 g, 85% over 3 steps) as a colourless oil.

^{1}H NMR (400 MHz, $CDCl_3$) δ 7.00 (s, 2H, *H*-Ar), 5.84 – 5.69 (m, 1H, *H*-1), 5.04 – 4.95 (m, 2H, alkene), 4.30 (t, *J* = 6.8 Hz, 2H, *H*-13), 3.79 – 3.69 (m, 1H, *H*-5), 3.65 (d, *J* = 5.5 Hz, 1H, 1.67 (m, 2H, *H*-12), 1.66 – 1.57 (m, 1H, *H*-3), 1.53 – 1.27 (m, 12H, *H*-4, *H*-6, *H*-7, *H*-8, *H*-10, *H*-11), 1.27 – 1.20 (m, 18H, CH_3-*i*Pr), 0.95 (m, 18H, OTES-CH_3), 0.88 (d, *J* = 6.7 Hz, 3H, *H*-42), 0.65 – 0.52 (m, 12H, OTES-CH_2). See spectrum.

^{13}C NMR (101 MHz, $CDCl_3$) δ 171.13 (*C*O-benzoate), 150.19 (*C*-*para*), 144.85 (*C*-*ortho*), 137.34 (*C*H-1), 130.84 (*C*-*ipso*), 120.97 (*C*-*meta*), 115.98 (*C*H_2- alkene), 72.29 (*C*H-9), 70.54 (*C*H-5), 65.18 (*C*H_2-13), 44.50 (*C*H_2-4), 41.60 (*C*H_2-2), 37.77 (*C*H_2-6 *or* 8 *or* 10), 37.69 (*C*H_2-6 *or* 8 *or* 10), 37.02 (*C*H_2-6 *or* 8 *or* 10), 34.58 (*C*H-*i*Pr *para*), 31.64 (*C*H-*i*Pr *ortho*), 29.50 (*C*H-3), 29.11 (*C*H_2-12), 24.29 (*C*H_3-*i*Pr *ortho*), 24.10 (*C*H_3-*i*Pr *para*), 22.12 (*C*H_2-11), 21.17 (*C*H_2-7), 20.08 (*C*H_3-42), 7.12 (OTES-*C*H_3), 7.09 (OTES-*C*H_3), 5.36 (OTES-*C*H_2), 5.28 (OTES-*C*H_2). See spectrum.

HRMS (m/z): (ESI) calculated for $C_{43}H_{80}O_4Si_2$ [M+H]+ 717.5673, found 717.5745.

TLC: R_f = 0.55 (90:10 hexane/EtOAc, stained with *p*-anisaldehyde).

IR (ν_{max}/cm^{-1}, neat): 2956, 2936, 2911, 2875, 1727 (C=O), 1607, 1460, 1381, 1250, 1076, 1008 (Si-O), 725 (Si-C).

$[\alpha]_D^{25}$: -6 (*c* = 1, $CHCl_3$).

(5R,9R,11R)-11-methyl-5,9-bis((triethylsilyl)oxy)tetradec-13-en-1-yl 2,4,6-triisopropylbenzoate (3*R*-F1)

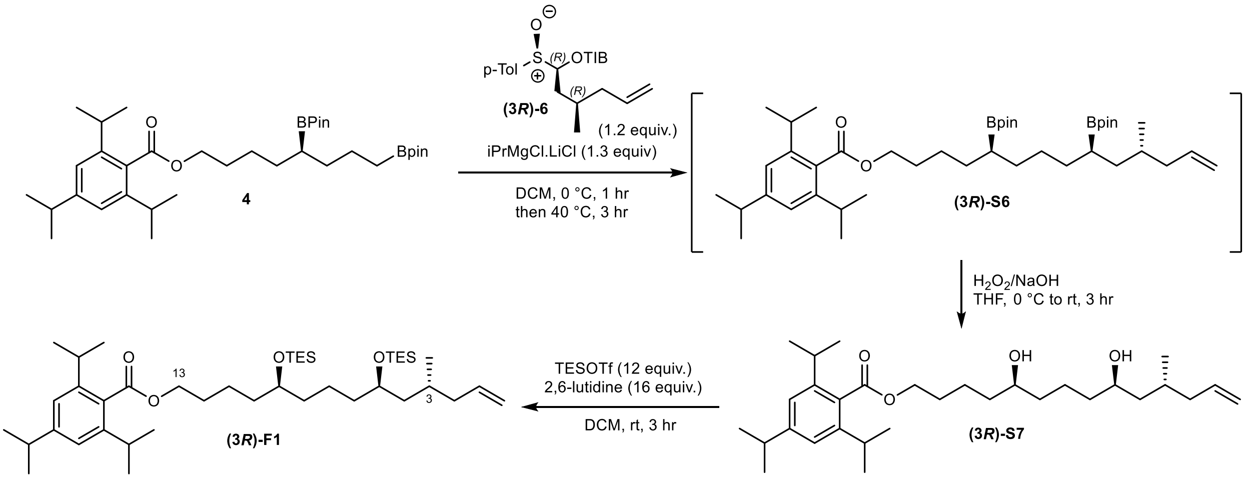

Prepared following General Procedure **2** using boronic ester **4** (800 mg, 1.31 mmol), sulfoxide **(3*R*)-6** (757 mg, 1.57 mmol), *i*PrMgCl·LiCl (1.20 M in THF, 1.41 mL, 1.70 mmol). The reaction mixture was filtered through silica treated and eluted with 1% triethylamine in diethyl ether. The filtrate was evaporated *in vacuo* and the crude boronic ester **(3*R*)-S6** oxidised according to General Procedure **4**. The crude mixture was purified by normal phase column chromatography (Biotage 25 g pentane: Et_2O, 0-50%) to afford a mixture of diol **(3*R*)-S7** and pinacol. The mixture was then TES protected according to General Procedure **5**, using triethylsilyl triflate (4.14 g, 3.54 mL, 15.7 mmol), 2,6-lutidine (2.24 g, 2.42 mL, 20.9 mmol). The crude mixture was purified by normal phase flash column chromatography (Biotage 50 g pentane: Et_2O, 0-10%) to afford the title compound (685 mg, 73% over 3 steps) as a colourless oil.

^{1}H NMR (500 MHz, $CDCl_3$) δ 7.00 (s, 2H, *H*-Ar), 5.77 (ddt, *J* = 17.3, 10.4, 7.0 Hz, 1H, *H*-1), 5.03 – 4.94 (m, 2H, alkene), 4.29 (t, *J* = 6.8 Hz, 2H, *H*-13), 3.77 – 3.68 (m, 1H, *H*-5), 3.63 (dddd, *J* = 5.4 Hz, 1H, , *H*-9), 2.93 – 2.80 (m, 3H, C*H*-*i*Pr), 2.09 – 2.00 (m, 1H, *H*-2’), 1.94 – 1.86 (m, 1H, *H*-2’’), 1.79 – 1.64 (m, 3H, *H*-12, *H*-3), 1.51 – 1.27 (m, 13H, *H*-4’, *H*-6, *H*-7, *H*-8, *H*-10, *H*-11), 1.24 (d, *J* = 6.9 Hz, 18H, CH_3-*i*Pr), 1.17 (dddd, *J* = 13.3, 8.6, 4.2, 4.2 Hz, 2H, *H*-4’’), 0.98 – 0.91 (m, 18H, OTES-CH_3), 0.87 (d, *J* = 6.6 Hz, 4H, *H*-42), 0.63 – 0.55 (m, 12H, OTES-CH_2). See spectrum.

^{13}C NMR (126 MHz, $CDCl_3$) δ 171.15 (*C*O-benzoate), 150.20 (*C-para*), 144.85 (*C-ortho*), 137.57 (*C*H-1), 130.82 (*C-ipso*), 120.97 (*C-meta*), 115.83 (CH_2- alkene), 72.28 (*C*H-9), 70.43 (*C*H-5), 65.19 (CH_2-13), 44.30 (CH_2-4), 42.15 (CH_2-2), 38.50 (CH_2-6 *or* 8 *or* 10), 37.66

(CH_2-6 *or* 8 *or* 10), 37.01 (CH_2-6 *or* 8 *or* 10), 34.58 (*C*H-*i*Pr *para*), 31.64 (*C*H-*i*Pr *ortho*), 29.21 (*C*H-3), 29.10 (CH_2-12), 24.29 (CH_3-*i*Pr *ortho*), 24.10 (CH_3-*i*Pr *para*), 22.14 (CH_2-11), 21.20 (CH_2-7), 19.72 (CH_3-42), 7.12 (OTES-CH_3), 7.09 (OTES-CH_3), 5.37 (OTES-CH_2), 5.26 (OTES-CH_2). See spectrum.

HRMS (m/z): (ESI) calculated for $C_{43}H_{80}O_4Si_2$ [M+H]+ 717.5673, found 717.5659.

TLC: R_f = 0.57 (90:10 hexane/EtOAc, stained with *p*-anisaldehyde).

IR (ν_{max}/cm^{-1}, neat): 2956, 2933, 2911, 2875, 1727 (C=O), 1607, 1460, 1380, 1250, 1075, 1007 (Si-O), 723 (Si-C).

$[\alpha]_D^{25}$: -4 (c = 1, $CHCl_3$).

4.2 Fragment 2

(*S*)-3,4-Bis(4,4,5,5-tetramethyl-1,3,2-dioxaborolan-2-yl)butyl 2,4,6-triisopropylbenzoate (7)

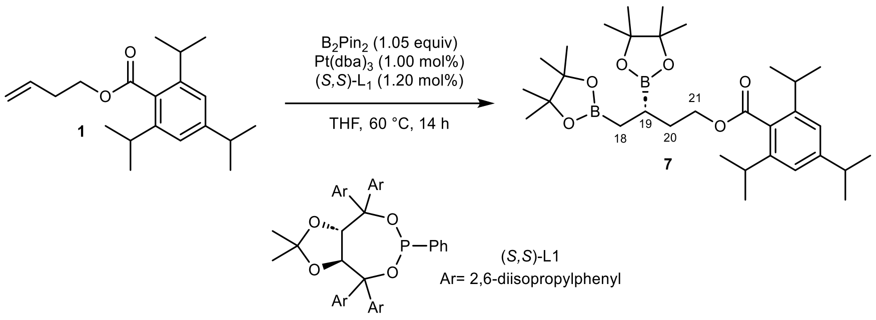

According to the modified literature procedure[11], homoallyl-TIB ester **1** was added to a flame dried Schlenk tube equipped with a magnetic stir bar was added $Pt(dba)_3$ (151 mg, 165 µM, 1.00 mol%), (*S,S*)-**L1** (180 mg, 198 µM, 1.20 mol%) and B_2Pin_2 (4.41 g, 17.4 mmol, 1.05 equiv.) under a nitrogen atmosphere. After addition of solids, the Schlenk was evacuated and refilled with nitrogen three times. Then, THF (1.00 M with respect to the alkene) was added via syringe. The solution was heated to 80 °C in an oil bath for 30 minutes. The reaction mixture was then cooled to room temperature and alkene (5.00 g, 16.5 mmol, 1.00 equiv.) was added neat. The reaction mixture was stirred 14 h at 60 °C. The reaction mixture was then cooled to room temperature and the solvent was evaporated under reduced pressure. The crude mixture was purified by automated flash column chromatography (Biotage HC-50 g, pentane: Et_2O 0-10%) to afford the title compound (8.24 g, 90%) as a colourless oil.

The enantiomeric excess was determined upon oxidation of the bis(boronic ester) (see **GP9.1**).

All recorded spectroscopic data matched that previously reported[12]

TLC: *R*f = 0.40 (85:15 pentane/Et2O, stained with *p*-anisaldehyde

^{1}H NMR (400 MHz, $CDCl_3$) δ 6.98 (s, 2H, *H*-Ar), 4.33 (t, *J* = 6.8, 2H, *H*-21), 2.87 (hept, *J* = 6.9 Hz, 3H, C*H*-*i*Pr), 1.90 (ddt, *J* = 13.6, 8.4, 6.8 Hz, 1H, *H*-20'), 1.73 (dq, *J* = 13.8, 7.0 Hz, 1H, *H*-20''), 1.24 (d, *J* = 6.9 Hz, 18H, CH_3-*i*Pr), 1.21 (m, 24H, CH_3-Bpin), 0.94 – 0.84 (m, 3H, *H*-18 & *H*-19). See spectrum.

^{13}C NMR (101 MHz, $CDCl_3$) δ 171.13 (*C*O-benzoate), 149.98 (*C*H-TIB-*para*), 144.88 (*C*H-TIB-*ortho*), 131.10 (*C*H-TIB-*ipso*), 120.88 (*C*H-TIB-*meta*), 83.14 (*C*-Bpin), 83.07 (*C*-Bpin), 64.64 (CH_2-21), 34.58 (*C*H-*i*Pr *para*), 32.19 (CH_2-20), 31.55 (*C*H-*i*Pr *ortho*), 25.00 (CH_3-Bpin), 24.95 (CH_3-Bpin), 24.87 (CH_3-Bpin), 24.32 (CH_3-*i*Pr), 24.30 (CH_3-*i*Pr), 24.11 (CH_3-*i*Pr). See spectrum.

carbon next to boron (CH_2-18 & *C*H-19) not *observed due to quadrupolar relaxation*

HRMS (m/z): (MALDI) calculated for $C_{32}H_{54}B_2O_6$ [M+Na]+ 579.4010, found 579.4016.

IR (ν_{max}/cm^{-1}, neat): 2963, 2930, 2871, 1724 (C=O), 1463, 1371, 1317, 1251, 1140, 1077.

$[\alpha]_D^{25}$: 6 (*c* = 1, $CHCl_3$).

The racemic bis-boronic ester was prepared by diboration using a method described in the literature for similar compounds.

*The enantioenriched bis-boronic ester and racemic bis-boronic ester were both oxidised according to **GP4** for chiral HPLC analysis.*

HPLC on chiral stationary phase Daicel Chiralpak-IB column (25 cm), 97:3 *n*-hexane:IPA, flow rate: 0.3 mL/min., rt, 210 nm, Retention times: 55.59 min. (S, major) and 58.95 min. (R, minor).

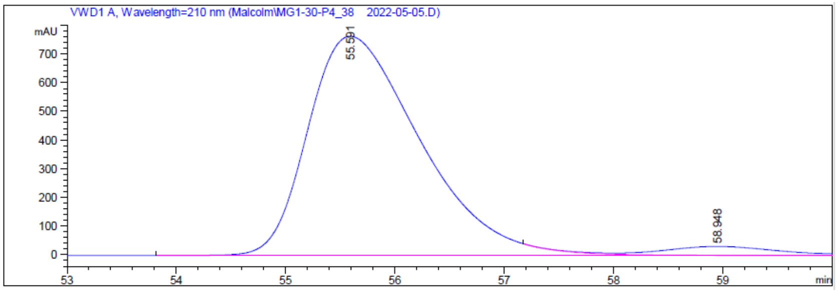

Peak #	RetTime [min]	Type	Width [min]	Area [mAU*s]	Height [mAU]	Area %
1	55.591	BV R	1.0970	5.45397e4	764.75079	95.7918
2	58.948	VB E	1.1421	2395.96533	31.24835	4.2082

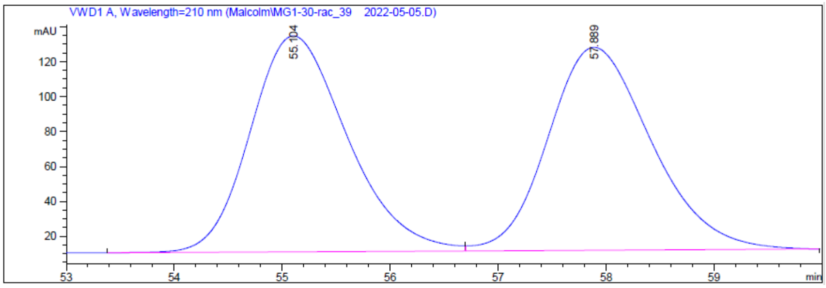

Peak #	RetTime [min]	Type	Width [min]	Area [mAU*s]	Height [mAU]	Area %
1	55.104	BV	0.9656	7732.78467	123.63711	49.9796
2	57.889	VBA	1.0246	7739.08398	116.18924	50.0204

(3*R*,5*R*)-3,5-Dihydroxyoct-7-en-1-yl 2,4,6-triisopropylbenzoate (8)

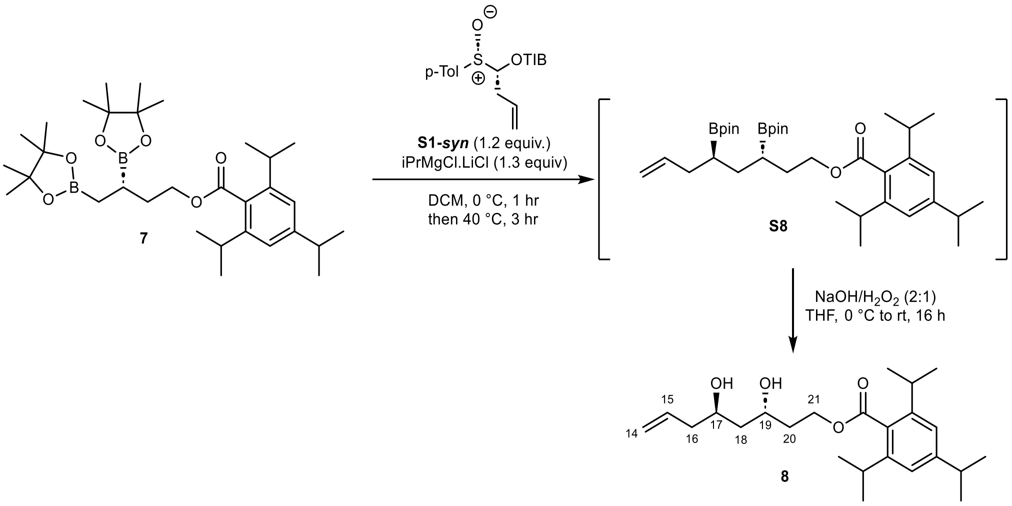

Prepared following General Procedure 2 using boronic ester **7** (2.00 g, 3.59 mmol), sulfoxide **S1-*syn*** (1.90 g, 4.31 mmol), *i*PrMgCl·LiCl (1.2 M in THF, 3.89 mL, 4.67 mmol) in CH_2Cl_2 (18 mL). The organics were concentrated under reduced pressure and the crude mixture was engaged in the next step without further purification.

Crude 1,3-bis-boronic ester **S8** was oxidised according to General Procedure 4 using THF (18.00 mL, 0.20 M with respect to bis-boronic ester), a preformed solution of 3 M NaOH (12.00 mL) and H_2O_2 30% v/v (6.00 mL). The crude mixture was purified by automated flash column chromatography (Biotage HC-100g, pentane: EtOAc 0-40%) to afford the title compound (904 mg, 64% yield over 2 steps, >20:1 dr) as a colourless oil.

All recorded spectroscopic data matched that previously reported[12]

^{1}H NMR (400 MHz, $CDCl_3$) δ 7.00 (s, 2H, *H*-Ar), 5.87 – 5.73 (m, 1H, *H*-15), 5.19 – 5.10 (m, 2H, *H*-14), 4.56 (ddd, *J* = 11.1, 7.4, 6.3 Hz, 1H, *H*-21), 4.40 (dt, *J* = 11.4, 5.8 Hz, 1H, *H*-21), 4.19 – 4.07 (m, 1H, *H*-19), 4.06 – 3.96 (m, 1H, *H*-17), 2.96 – 2.77 (m, 3H, *H*-iPr), 2.35 – 2.20 (m, 3H, *H*-16), 1.97 – 1.84 (m, 2H, *H*-20), 1.75 – 1.62 (m, 2H, *H*-18), 1.30 – 1.20 (m, 18H, CH_3-iPr). See spectrum.

^{13}C NMR (101 MHz, $CDCl_3$) δ 171.34 (*C*O-benzoate), 150.39 (*C-para*), 144.92 (*C-ortho*), 134.59 (*C*H-15), 130.46 (*C-ipso*), 121.02 (*C*H-Ar), 118.65 (CH_2-14), 68.26 (*C*H-17), 66.30 (CH_2-19), 62.32 (CH_2-21), 42.24 (CH_2-18), 42.13 (CH_2-16), 36.55 (CH_2-20), 34.57 (*C*-*i*Pr *para*), 31.70 (*C*H-*i*Pr *ortho*), 24.31 (CH_3-*i*Pr), 24.28 (CH_3-*i*Pr), 24.08 (CH_3-*i*Pr). See spectrum.

IR (ν_{max}/cm^{-1}, neat): 3454 (OH), 2970, 2928, 2869, 1738 (C=O), 1649 (C=C), 1463, 1365, 1085, 1042, 1023, 812.

2-((4*R*,6*R*)-6-Allyl-2,2-dimethyl-1,3-dioxan-4-yl)ethyl 2,4,6-triisopropylbenzoate (S9)

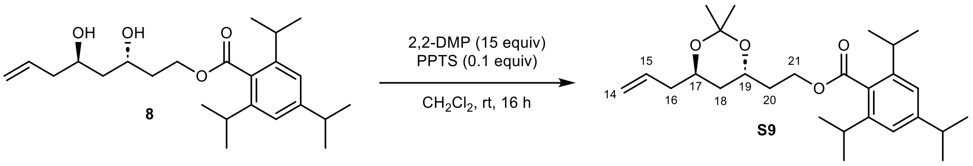

According to a literature procedure, to a solution of diol **8** (850 mg, 2.18 mmol, 1.00 equiv.) in CH_2Cl_2 (22 mL, 0.10 M), 2,2-dimethoxypropane (3.40 g, 3.26 mmol, 15.0 equiv.) and pyridinium p-toluene sulfonate (54.7 mg, 218 µM, 0.10 equiv.) were added. The reaction mixture was stirred 16 h at ambient temperature. Sat. aq. $NaHCO_3$ was added and the layers were separated. The aqueous phase was extracted with CH_2Cl_2 (3x). The combined organics were dried over anhydrous Na_2SO_4, filtered and concentrated under reduced pressure. The

crude mixture was purified by normal phase flash column chromatography (Biotage HC-50 g, *n*-hexane: EtOAc 0-10%) to afford the title compound (860 mg, 92%) as a colourless oil.

Note: due to the acetonide lability under acidic conditions, it is recommended to use $CDCl_3$ filtered over basic alumina or CD_2Cl_2 as solvent for NMR analysis.

All recorded spectroscopic data matched that previously reported[12]

^{1}H NMR (400 MHz, $CDCl_3$) δ 7.01 (s, 2H, *H*-Ar), 5.79 (ddt, *J* = 17.1, 10.3, 6.8 Hz, 1H, *H*-15), 5.15 – 5.00 (m, 2H, *H*-14), 4.45 – 4.32 (m, 2H, *H*-21), 3.96 (m, 1H, *H*-19), 3.88 (m, 1H, *H*-17), 2.95 – 2.78 (m, 3H, *H*-iPr), 2.30 (ddd, *J* = 13.2, 6.7, 6.7 Hz, 1H, *H*-16'), 2.24 – 2.14 (, 1H, *H*-16''), 1.96 – 1.81 (m, 2H, *H*-20), 1.63 (dd, *J* = 7.7 Hz, 2H, *H*-18), 1.36 (s, 3H, CH_3-acetonide), 1.35 (s, 3H, CH_3-acetonide), 1.24 (d, *J* = 6.8 Hz, 18H, CH_3-iPr). See spectrum.

^{13}C NMR (101 MHz, $CDCl_3$) δ 171.06 (*C*O-benzoate), 150.29 (*C*-*para*), 144.88 (*C*-*ortho*), 134.46 (*C*H-15), 130.66 (*C*-*ipso*), 121.00 (*C*H-Ar), 117.12 (CH_2-14), 100.57 (*C*O *anti*-acetonide), 66.25 (*C*H-17), 63.62 (CH_2-19), 61.83 (CH_2-21), 40.26 (CH_2-16), 38.03 (CH_2-18), 35.02 (CH_2-20), 34.57 (*C*-*i*Pr *para*), 31.67 (*C*H-*i*Pr *ortho*), 24.91 (*C*H3-*anti*-acetonide), 24.88 (*C*H3-*anti*-acetonide), 24.36 (CH_3-*i*Pr), 24.25 (CH_3-*i*Pr), 24.09 (CH_3-*i*Pr). See spectrum.

2-((4*R*,6*R*)-2,2-Dimethyl-6-(3-(4,4,5,5-tetramethyl-1,3,2-dioxaborolan-2-yl)propyl)-1,3-dioxan-4-yl)ethyl 2,4,6-triisopropylbenzoate (F2)

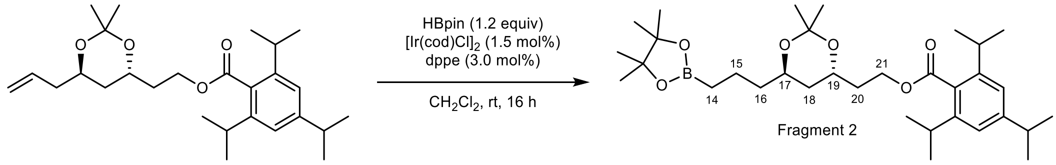

Prepared following General Procedure **3** using pinacolborane (370 mg, 0.42 mL, 2.89 mmol), alkene **S9** (830 mg, 1.93 mmol), $[Ir(cod)Cl]_2$ (19.4 mg, 18.9 µM) and 1,2-bis(diphenylphosphino)ethane dppe (23.0 mg, 57.8 µM) in CH_2Cl_2 (6.4 mL). The crude mixture was purified by normal phase flash column chromatography (Biotage HC-50 g, pentane: Et_2O 0-10%) to afford the title compound (0.88 g, 82%) as a colourless oil.

All recorded spectroscopic data matched that previously reported[12]

^{1}H NMR (400 MHz, $CDCl_3$) δ 7.00 (s, 2H, *H*-Ar), 4.48 – 4.28 (m, 2H, *H*-21), 4.03 – 3.87 (m, 1H, *H*-19), 3.87 – 3.71 (m, 1H, *H*-17), 2.86 (hept, *J* = 6.9 Hz, 3H, *H*-iPr), 1.99 – 1.78 (m, 2H, *H*-20), 1.68 – 1.36 (m, 6H, *H*-15, *H*-16, *H*-18),1.34 (s, 3H, CH_3-acetonide), 1.32 (s, 3H,

CH_3-acetonide), 1.28 – 1.20 (m, 30H, CH_3-*i*Pr & CH_3-Bpin), 0.78 (t, J = 7.4 Hz, 2H, *H*-14). See spectrum.

^{13}C NMR (101 MHz, $CDCl_3$) δ 171.08 (*C*O-benzoate), 150.27 (*C-para*), 144.89 (*C-ortho*), 130.71 (*C-ipso*), 121.01 (*C*H-Ar), 100.45 (*C*O *anti*-acetonide), 83.08 (*C*-Bpin), 66.54 (*C*H-17), 63.64 (*C*H_2-19), 61.92 (*C*H_2-21), 38.78 (*C*H_2-16), 38.76 (*C*H_2-18), 35.07 (*C*H_2-20), 34.59 (*C*-*i*Pr *para*), 31.67 (*C*H-*i*Pr *ortho*), 24.99 (*C*H_3-Bpin), 24.97 (*C*H_3-Bpin), 24.94 (*C*H3-*anti*-acetonide), 24.86 (*C*H3-*anti*-acetonide), 24.36 (*C*H_3-*i*Pr), 24.26 (*C*H_3-*i*Pr), 24.10 (*C*H_3-*i*Pr), 19.86 (*C*H_2-15). See spectrum.

carbon next to boron (*C*H_2-14) not *observed due to quadrupolar relaxation*

HRMS (m/z): (ESI) calculated for $C_{33}H_{55}BO_6$ [M+Na]+ 581.3984, found 581.4000.

IR (ν_{max}/cm^{-1}, neat): 2961, 2934, 2871, 1726 (C=O), 1607, 1379, 1250, 1144, 1076.

4.3 Fragment 3

Propane-1,3-diyl bis(2,4,6-triisopropylbenzoate) (9)

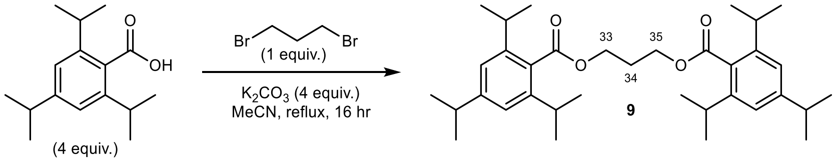

Potassium carbonate (21.9 g, 159 mmol, 4.00 equiv.) was added to a stirred solution of 2,4,6-triisopropylbenzoic acid (39.4 g, 159 mmol, 4.00 equiv.) in MeCN (120 mL, 0.33 M). The mixture was stirred vigorously for 10 min then 1,3-dibromopropane (8.00 g, 4.02 mL, 39.6 mmol, 1.00 equiv.) was added. The resulting mixture was refluxed for 16 h. The reaction mixture was cooled to room temperature and the undissolved K_2CO_3 filtered off and washed with EtOAc under reduced pressure. The filtrate was evaporated under reduced pressure, and the resulting residue was dissolved in EtOAc (200 mL), washed with water (2 x 100 mL) and brine (100 mL) before drying over $MgSO_4$. The crude residue was purified by flash column chromatography (4% Et_2O in pentane) to give the title compound (19.8 g, 93%) as a white amorphous solid.

^{1}H NMR (400 MHz, $CDCl_3$) δ 7.03 (s, 4H, *H*-Ar), 4.44 (t, *J* = 6.4 Hz, 4H, *H*-33 & *H*-35), 2.88 (m, 6H, C*H*-*i*Pr), 2.19 (p, *J* = 6.4 Hz, 2H, *H*-34), 1.27 (dd, *J* = 6.9, 1.0 Hz, 36H, CH_3-*i*Pr). See spectrum.

^{13}C NMR (101 MHz, $CDCl_3$) δ 170.85 (*C*O-benzoate), 150.41 (*C*-*para*), 144.94 (*C*-*ortho*), 130.38 (*C*-*ipso*), 121.04 (*C*H-*meta)*, 61.58 (*C*H_2-33 & *C*H_2-35), 34.58 (*C*H-*i*Pr *para*), 31.75 (*C*H-*i*Pr *ortho*), 28.34 (*C*H_2-34), 24.30 (*C*H_3-*i*Pr *ortho*), 24.08 (*C*H_3-*i*Pr *para*). See spectrum.

HRMS (m/z): (ESI) calculated for $C_{35}H_{52}O_4$ [M+H]+ 559.3758, found 559.3759.

TLC: ***R*f** = 0.48 (85:15 hexane/EtOAc, stained with ceric ammonium molybdate (CAM))

IR (ν_{max}/cm^{-1}, neat): 2961, 2930, 2908, 2870, 1727 (C=O), 1606, 1461, 1248, 1068.

(1R)-1-(p-tolylsulfinyl)propane-1,3-diyl bis(2,4,6-triisopropylbenzoate) (10-*anti*)

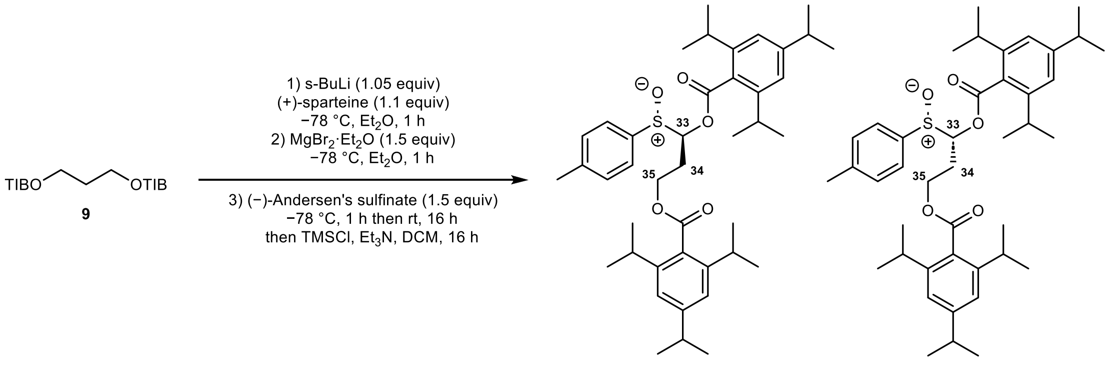

Prepared following General Procedure 1 using TIB ester **9** (10.0 g, 18.6 mmol), (+)-sparteine (4.80 g, 20.5 mmol, 4.71 mL), *s*-BuLi (1.30 M in hexanes) (15.0 mL, 19.6 mmol), (−)-Andersen's sulfinate (8.23 g, 27.9 mmol), magnesium turnings (1.81 g, 74.5 mmol), dibromoethane (5.25 g, 27.9 mmol, 2.42 mL), Et_3N (2.82 g, 27.9 mmol, 3.89 mL), trimethylsilyl chloride (2.63 g, 24.2 mmol, 3.07 mL). The crude residue was purified by automated flash column chromatography (Biotage HC-200 g, *n*-hexane: EtOAc 0-10%) to give a mixture of diastereomers (11.2 g, 89%) as a white amorphous solid. The *syn* and *anti* diastereomers were inseparable by normal phase column chromatography so a portion (2.10 g) was purified portion-wise (*ca*. 80 mg injections) by reverse phase preparative HPLC to give:

Major *anti* diastereomer (**10-*anti***) (1.80 g, 14%, 86% recovery) as a white amorphous solid.

^{1}H NMR (400 MHz, $CDCl_3$) δ 7.46 (d, *J* = 8.2 Hz, 2H, *H*-Ar), 7.22 (d, *J* = 7.8 Hz, 2H, *H*-Ar), 6.95 (d, *J* = 14.1 Hz, 4H, *H*-Ar), 6.05 (dd, *J* = 9.1, 3.6 Hz, 1H, *H*-33), 4.42 – 4.21 (m, 2H, *C*H_2-35), 2.90 – 2.62 (m, 6H, C*H*-*i*Pr), 2.33 (s, 3H, C*H*$_3$-tolyl), 2.31 – 2.21 (m, 1H, *H*-34'), 1.67 (ddt, *J* = 14.6, 9.1, 5.7 Hz, 1H, *C*H_2-34''), 1.25 – 1.07 (m, 36H, C*H*$_3$-*i*Pr). See spectrum.

^{13}C NMR (101 MHz, $CDCl_3$) δ 170.64 (*C*O-benzoate), 169.04 (*C*O-benzoate), 151.06 (*C*-TIB-*para*), 150.53 (*C*-TIB-*para*), 145.38 (*C*-TIB-*ortho*), 145.00 (*C*-TIB-*ortho*), 142.44 (*C*-tolyl-*ipso*), 135.95 (*C*-tolyl-*para*), 130.08 (*C*H-tolyl-*meta*), 130.02 (*C*-TIB-*ipso*), 128.76 (*C*-TIB-*ipso*), 125.64 (*C*H-tolyl-*ortho*), 121.24 (*C*H-TIB-*meta*), 121.04 (*C*H-TIB-*meta*), 84.99 (*C*H-33), 60.19 (*C*H_2-35), 34.59 (*C*H-*i*Pr *para*), 34.57 (*C*H-*i*Pr *para*), 31.95 (*C*H-*i*Pr *ortho*), 31.78 (*C*H-*i*Pr *ortho*), 27.26 (*C*H_2-34), 24.75 (*C*H_3-*i*Pr), 24.30 (*C*H_3-*i*Pr), 24.26 (*C*H_3-*i*Pr), 24.21 (*C*H_3-*i*Pr), 24.09 (*C*H_3-*i*Pr), 24.04 (*C*H_3-*i*Pr), 21.60 (*C*H_3-tolyl). See spectrum.

HRMS (m/z): (ESI) calculated for $C_{42}H_{58}O_5S$ [M+Na]+ 697.3897, found 697.3876.

TLC: R_f = 0.44 (90:10 pentane/Et_2O, stained with *p*-anisaldehyde).

IR (ν_{max}/cm^{-1}, neat): 2961, 2929, 2870, 1731 (C=O), 1606, 1461, 1384, 1248, 1039 (S=O).

$[\alpha]_D^{25}$: −22 (*c* = 1, $CHCl_3$).

Minor *syn* diastereomer (**10-*syn***) (170 mg, 1.4%, 8% recovery) as a white amorphous solid in a 78:22 *dr* (*syn/anti*). Analysed without further purification.

^{1}H NMR (400 MHz, $CDCl_3$) δ 7.64 (d, *J* = 7.9 Hz, 2H, *H*-Ar), 7.33 (d, *J* = 8.2 Hz, 2H, *H*-Ar), 7.07 (s, 2H, *H*-Ar), 6.98 (s, 2H, *H*-Ar), 5.82 (dd, *J* = 10.3, 3.1 Hz, 1H, *H*-33), 4.41 – 4.27 (m, 2H, *C*H_2-35), 3.03 – 2.79 (m, 4H, C*H*-*i*Pr), 2.66 (hept, *J* = 6.8 Hz, 2H, C*H*-*i*Pr), 2.45 – 2.32 (m, 4H, C*H*$_3$-tolyl & *H*-34'), 2.15 – 2.07 (m, 1H, *H*-34''), 1.34 – 1.22 (m, 24H, C*H*$_3$-*i*Pr), 1.18 (d, *J* = 6.8 Hz, 6H, C*H*$_3$-*i*Pr), 1.13 (d, *J* = 6.8 Hz, 6H, C*H*$_3$-*i*Pr). See spectrum.

^{13}C NMR (101 MHz, $CDCl_3$) δ 170.44 (*C*O-benzoate), 170.29 (*C*O-benzoate), 151.15 (*C*-TIB-*para*), 150.39 (*C*-TIB-*para*), 145.49 (*C*-TIB-*ortho*), 144.95 (*C*-TIB-*ortho*), 141.89 (*C*-tolyl-*ipso*), 137.03 (*C*-tolyl-*para*), 130.28 (*C*H-tolyl-*meta*), 130.00 (*C*-TIB-*ipso*), 128.63 (*C*-TIB-*ipso*), 124.27 (*C*H-tolyl-*ortho*), 121.19 (*C*H-TIB-*meta*), 120.94 (*C*H-TIB-*meta*), 89.60 (*C*H-33), 60.16 (*C*H_2-35), 34.59 (*C*H-*i*Pr *para*), 34.52 (*C*H-*i*Pr *para*), 31.86 (*C*H-*i*Pr *ortho*), 31.69 (*C*H-*i*Pr *ortho*), 24.55 (*C*H_3-*i*Pr), 24.32 (*C*H_3-*i*Pr), 24.27 (*C*H_3-*i*Pr), 24.23 (*C*H_3-*i*Pr), 24.03 (*C*H_3-*i*Pr), 22.76 (*C*H_3-*i*Pr), 21.54 (*C*H_2-34). See spectrum.

HRMS (m/z): (ESI) calculated for $C_{42}H_{58}O_5S$ [M+Na]+ 697.3897, found 697.3884.

TLC: ***R*f** = 0.44 (90:10 pentane/Et_2O, stained with *p*-anisaldehyde).

IR (ν_{max}/cm^{-1}, neat): 2962, 2926, 2870, 1730 (C=O), 1606, 1460, 1363, 1233, 1033 (S=O).

(*R*)-3-(4,4,5,5-Tetramethyl-1,3,2-dioxaborolan-2-yl)hex-5-en-1-yl 2,4,6-triisopropylbenzoate (11)

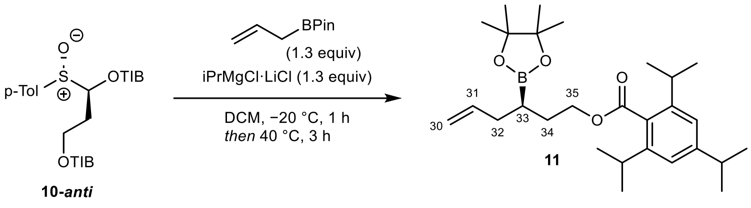

*i*PrMgCl·LiCl (1.30 M in THF) (2.44 mL, 1.30 equiv.) was added dropwise to a mixture of allylboronic acid pinacol ester (534 mg, 3.17 mmol, 1.00 equiv.) and sulfoxide **10-*anti*** (1.65 g, 2.44 mmol, 1.10 equiv.) in CH_2Cl_2 (12 mL, 0.20 M with respect to boronic ester) at –20 °C and the resulting solution was stirred 1 h at the same temperature (*pale yellow solution*). After warming to room temperature, the reaction mixture was heated at 40 °C for 3 h (*turbid white solution*). The reaction mixture was then cooled to room temperature, and was quenched with saturated aqueous NH_4Cl. The aqueous phase was extracted with Et_2O (3x). The combined organics were dried over anhydrous Na_2SO_4 and filtered over a short pad of silica deactivated with Et_2O: Et_3N 1% in order to remove the residual TIB acid. The crude mixture was purified by normal phase flash column chromatography (Biotage HC-50 g, *n*-hexane: EtOAc 0-30%) to afford a mixture of the title compound and bis-TIB ester **9** (1.15 g, 74% desired + 25% bis TIB ester). The mixture was taken through crude to the next step without further purification.

^{1}H NMR (400 MHz, $CDCl_3$) δ 6.99 (s, 2H, *H*-Ar), 5.79 (ddt, *J* = 17.0, 10.1, 6.9 Hz, 1H, *H*-31), 5.03 (ddd, *J* = 17.0, 2.5, 1.1 Hz, 1H, *H*-30 *cis*), 4.96 (ddt, *J* = 10.1, 2.1, 1.1 Hz, 1H, *H*-30 *trans*), 4.39 – 4.26 (m, 2H, *H*-35), 2.94 – 2.78 (m, *J* = 7.0 Hz, 3H, C*H*-*i*Pr), 2.30 – 2.12 (m, 2H, *H*-32), 1.91 – 1.72 (m, 2H, *H*-34), 1.27 – 1.23 (m, 18H, C*H*3-*i*Pr), 1.22 (s, 12H, C*H*3-Bpin). See spectrum.

^{13}C NMR (101 MHz, $CDCl_3$) δ 171.10 (*C*O-benzoate), 150.10 (*C*-TIB-*para*), 144.88 (*C*-TIB-*ortho*), 138.05 (*C*H-30), 130.95 (*C*-TIB-*ipso*), 120.93 (*C*H-TIB-*meta*), 115.57 (*C*H2-31), 83.36 (*C*-Bpin), 64.58 (*C*-35), 35.27 (*C*H2-32), 34.58 (*C*H-*i*Pr *para*), 31.59 (*C*H-*i*Pr *ortho*),

29.53 (CH_2-34), 24.98 (CH_3-Bpin), 24.91 (CH_3-Bpin), 24.30 (CH_3-*i*Pr), 24.11 (CH_3-*i*Pr). See spectrum.

carbon next to boron (*C*-33) *not observed due to quadrupolar relaxation*

HRMS (m/z): (ESI) calculated for $C_{28}H_{45}BO_4$ [M+Na]+ 479.3303, found 479.3298.

TLC: R_f = 0.32 (90:10 pentane/Et_2O, stained with *p*-anisaldehyde).

IR (ν_{max}/cm^{-1}, neat): 2962, 2929, 2870, 1725 (C=O), 1607, 1462, 1380, 1323, 1250, 1140, 1075.

$[\alpha]_D^{25}$: −6 (*c* = 1, $CHCl_3$).

(*R*)-3,6-Bis(4,4,5,5-tetramethyl-1,3,2-dioxaborolan-2-yl)hexyl 2,4,6-triisopropylbenzoate (12)

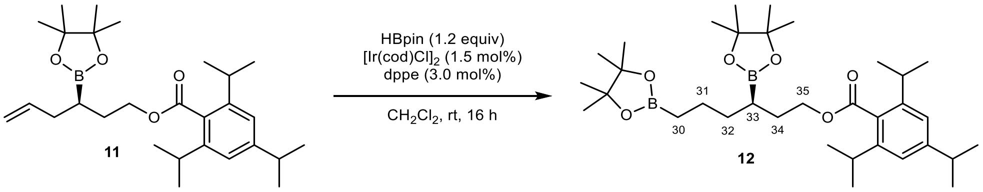

Prepared following General Procedure 3 using pinacolborane (345 mg, 0.391 mL, 2.69 mmol), alkene **11** (820 mg, 1.80 mmol), $[Ir(cod)Cl]_2$ (18.1 mg, 26.9 µmol) and 1,2-bis(diphenylphosphino)ethane dppe (21.5 mg, 53.9 µmol). The crude mixture was purified by normal phase flash column chromatography (Biotage HC-50 g, pentane: Et_2O 0-25%) to afford the title compound (1.16 g, 89%) as a colourless oil.

(*quantities calculated based on 72% by mass of the 1.14 g starting material being alkene* ***11***)

^{1}H NMR (400 MHz, $CDCl_3$) δ 6.98 (s, 2H, *H*-Ar), 4.30 (t, *J* = 7.0 Hz, 2H, *H*-35), 2.87 (h, *J* = 6.9 Hz, 3H, C*H*-*i*Pr), 1.89 – 1.72 (m, 2H, CH_2-34), 1.52 – 1.33 (m, 4H, CH_2-32 and CH_2-31), 1.23 (42H, CH_3-*i*Pr and CH_3-Bpin), 1.17 – 1.07 (m, 1H, C*H*-33), 0.76 (t, *J* = 7.2 Hz, 2H, CH_2-30). See spectrum.

^{13}C NMR (101 MHz, $CDCl_3$) δ 171.11 (*C*O-benzoate), 150.03 (*C*-TIB-*para*), 144.88 (*C*-TIB-*ortho*), 131.01 (*C*-TIB-*ipso*), 120.90 (*C*H-TIB-*meta*), 83.15 (*C*-Bpin), 82.97 (*C*-Bpin), 64.87 (*C*-35), 34.58 (*C*H-*i*Pr *para*), 33.85 (CH_2-32), 31.56 (*C*H-*i*Pr *ortho*), 29.93 (CH_2-34), 24.95 (CH_3-Bpin), 24.87 (CH_3-Bpin), 24.33 (CH_3-*i*Pr), 24.30 (CH_3-*i*Pr), 24.11 (CH_3-*i*Pr), 23.58 (CH_2-31). See spectrum.

carbon next to boron (*C*-30 & *C*-33) *not observed due to quadrupolar relaxation*

HRMS (m/z): (MALDI) calculated for $C_{34}H_{58}B_2O_6$ [M+Na]+ 607.4323, found 607.4319.

TLC: $\boldsymbol{R_f}$ = 0.33 (90:10 pentane/Et_2O, stained with *p*-anisaldehyde).

IR (ν_{max}/cm^{-1}, neat): 2962, 2929, 2870, 1724 (C=O), 1607, 1462, 1371, 1319, 1250, 1143, 1076, 967.

$[\alpha]_D^{25}$: −2 (*c* = 1, $CHCl_3$).

(3*R*,7*S*)-3,7-Bis(4,4,5,5-tetramethyl-1,3,2-dioxaborolan-2-yl)dec-9-en-1-yl 2,4,6-triisopropylbenzoate (13)

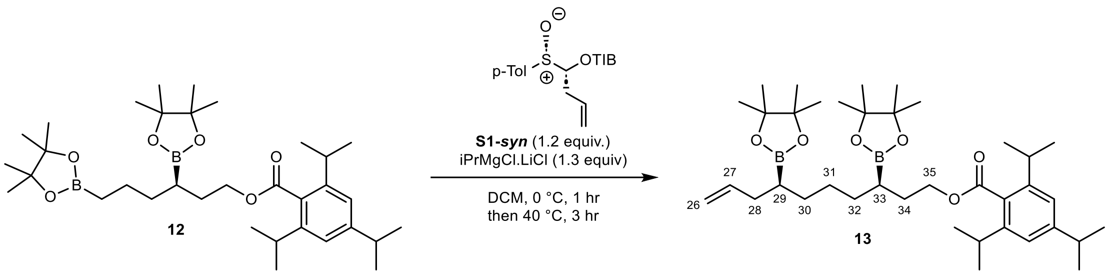

Prepared following General Procedure 2 using boronic ester **12** (1.06 g, 1.81 mmol), sulfoxide **S1-*syn*** (959 mg, 2.18 mmol), *i*PrMgCl·LiCl (1.20 M in THF, 1.96 mL, 2.36 mmol). The crude mixture was purified by normal phase flash column chromatography (Biotage HC-50 g, pentane: Et_2O 0-30%) to afford the title compound (938 mg, 81%) as a colourless oil.

^{1}H NMR (400 MHz, $CDCl_3$) δ 6.98 (s, 2H, *H*-Ar), 5.79 (ddt, *J* = 17.1, 10.2, 6.9 Hz, 1H, *H*-27), 4.99 (ddt, *J* = 17.1, 2.7, 1.5 Hz, 1H, *H*-26 *cis*), 4.91 (ddt, *J* = 10.1, 2.2, 1.1 Hz, 1H, *H*-26 *trans*), 4.30 (t, *J* = 7.0 Hz, 2H, *H*-35), 2.86 (hept, *J* = 7.0 Hz, 3H C*H*-*i*Pr), 2.13 (m, 2H, *H*-28), 1.87 – 1.71 (m, 2H, *H*-34), 1.51 – 1.27 (m, 6H, *H*-30, *H*-31, *H*-32), 1.24-1.21 (m, 42H, CH_3-*i*Pr & CH_3-Bpin), 1.16 – 0.99 (m, 2H, *H*-29 & *H*-33). See spectrum.

^{13}C NMR (101 MHz, $CDCl_3$) δ 171.10 (*C*O-benzoate), 150.05 (*C*-TIB-*para*), 144.88 (*C*-TIB-*ortho*), 138.84 (*C*H-26), 130.99 (*C*-TIB-*ipso*), 120.90 (*C*H-TIB-*meta*), 114.85 (CH_2-27), 83.16 (*C*-Bpin), 83.06 (*C*-Bpin), 64.84 (*C*-35), 35.51 (CH_2-28), 34.57 (*C*H-*i*Pr *para*), 31.56 (*C*H-*i*Pr *ortho*), 31.46 (CH_2-30 *or* CH_2-32), 31.20 (CH_2-30 *or* CH_2-32), 30.08 (CH_2-34), 28.58 (CH_2-31), 24.98 (CH_3-Bpin), 24.96 (CH_3-Bpin), 24.89 (CH_3-Bpin), 24.32 (CH_3-*i*Pr), 24.31 (CH_3-*i*Pr), 24.11 (CH_3-*i*Pr). See spectrum.

carbon next to boron (*C*-29 & *C*-33) *not observed due to quadrupolar relaxation*

HRMS (m/z): (MALDI) calculated for $C_{38}H_{64}B_2O_6$ [M+Na]+ 661.4794, found 661.4787.

TLC: $\boldsymbol{R}_\mathbf{f}$ = 0.62 (85:15 pentane/Et_2O, stained with *p*-anisaldehyde).

IR (ν_{max}/cm^{-1}, neat): 2963, 2926, 2869, 1723 (C=O), 1606, 1462, 1380, 1317, 1250, 1141, 1076, 737.

$[\alpha]_D^{25}$: −2 (*c* = 1, $CHCl_3$).

(3*R*,7*R*)-3,7,10-Tris(4,4,5,5-tetramethyl-1,3,2-dioxaborolan-2-yl)decyl 2,4,6-triisopropylbenzoate (14)

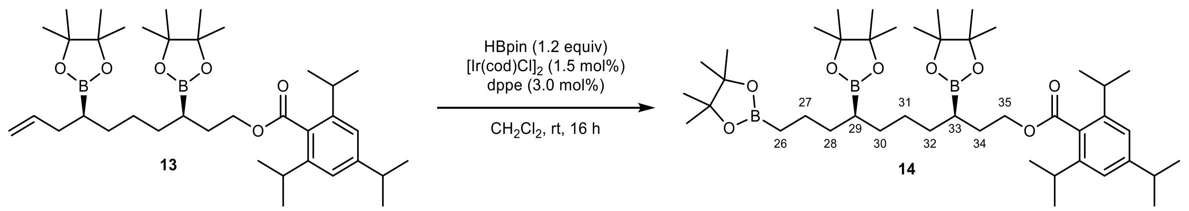

Prepared following General Procedure 3 using pinacolborane (463 mg, 524 µL, 3.61 mmol), alkene **13** (1.10 g, 2.41 mmol), $[Ir(cod)Cl]_2$ (24.2 mg, 36.1 µmol) and 1,2-bis(diphenylphosphino)ethane dppe (28.8 mg, 72.3 µmol). The crude mixture was purified by normal phase flash column chromatography (Biotage HC-50 g, pentane: Et_2O 0-25%) to afford the title compound (1.93 g, 90%) as a colourless oil.

^{1}H NMR (400 MHz, $CDCl_3$) δ 6.98 (s, 2H, *H*-Ar), 4.29 (t, *J* = 7.0 Hz, 2H, *H*-35), 2.85 (hept, *J* = 7.0 Hz, 3H, C*H*-*i*Pr), 1.87 – 1.70 (m, 2H, CH_2-34), 1.50 – 1.27 (m, 10H, CH_2-27, 28, 30, 31 & 32), 1.27 – 1.15 (m, 54H, CH_3-*i*Pr and CH_3-Bpin), 1.15 – 1.02 (m, 1H, C*H*-29 *or* 33), 0.93 (s, 1H, C*H*-29 *or* 33), 0.78 – 0.69 (m, 2H, C*H*-26). See spectrum.

^{13}C NMR (101 MHz, $CDCl_3$) δ 171.10 (*C*O-benzoate), 150.02 (*C*-TIB-*para*), 144.88 (*C*-TIB-*ortho*), 131.01 (*C*-TIB-*ipso*), 120.89 (*C*H-TIB-*meta*), 83.13 (*C*-Bpin), 82.90 (*C*-Bpin), 82.84 (*C*-Bpin), 64.88 (*C*-35), 34.57 (*C*H-*i*Pr *para*), 34.24 (*C*H_2-28), 31.65 (*C*H_2-30 *or* 32), 31.59 (*C*H_2-30 *or* 32), 31.55 (*C*H-*i*Pr *ortho*), 30.11 (*C*H_2-34), 28.83 (*C*H_2-31), 24.95 (*C*H_3-), 24.88 (*C*H_3-Bpin), 24.32 (*C*H_3-*i*Pr), 24.30 (*C*H_3-*i*Pr), 24.11 (*C*H_3-*i*Pr), 23.79 (*C*H_2-27). See spectrum.

carbon next to boron (*C*-26, *C*-29 & *C*-33) *not observed due to quadrupolar relaxation*

HRMS (m/z): (MALDI) calculated for $C_{44}H_{77}B_3O_8$ [M+Na]+ 789.5811, found 789.5821.

TLC: ***R*f** = 0.44 (95:15 pentane/Et_2O, stained with *p*-anisaldehyde).

IR (ν_{max}/cm^{-1}, neat): 2974, 2927, 2870, 1725 (C=O), 1607, 1462, 1379, 1317, 1252, 1144, 1076, 968.

$[\alpha]_D^{25}$: 0 (*c* = 1, $CHCl_3$).

(3*R*,7*R*,11*S*)-3,7,11-Tris(4,4,5,5-tetramethyl-1,3,2-dioxaborolan-2-yl)tetradec-13-en-1-yl 2,4,6-triisopropylbenzoate (15)

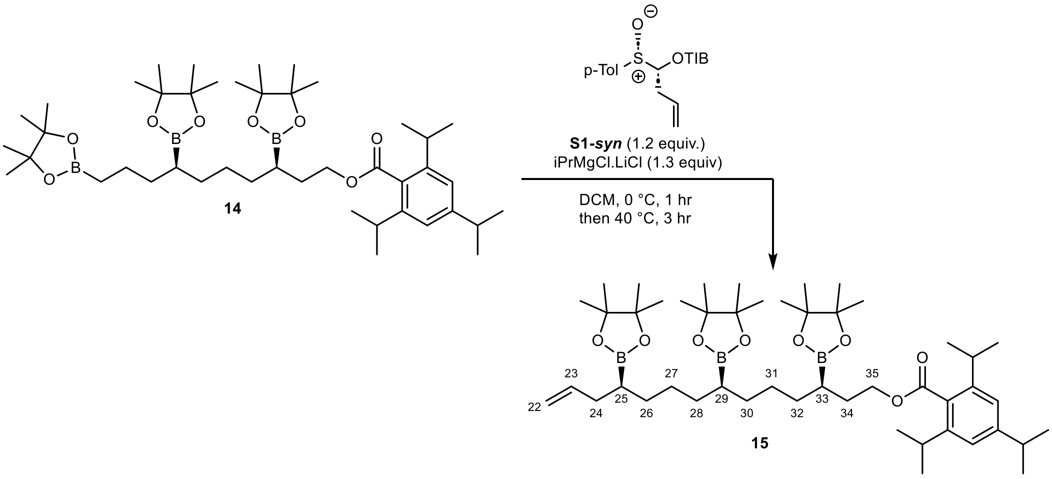

Prepared following General Procedure 2 using boronic ester **14** (1.00 g, 1.30 mmol), syn sulfoxide **S1-*syn*** (690 mg, 2.18 mmol), *i*PrMgCl·LiCl (1.20 M in THF, 1.41 mL, 1.70 mmol). The crude mixture was purified by normal phase flash column chromatography (Biotage HC-50 g, pentane: Et_2O 0-40%) to afford the title compound (759 mg, 71%) as a colourless oil.

^{1}H NMR (400 MHz, $CDCl_3$) δ 6.98 (s, 2H, *H*-Ar), 5.79 (ddt, *J* = 17.0, 10.2, 6.9 Hz, 1H, *H*-23), 5.03 – 4.95 (m, 1H *H*-22 *cis*), 4.91 (ddt, *J* = 10.1, 2.3, 1.1 Hz, 1H, *H*-22 *trans*), 4.34 – 4.24 (m, 2H, , *H*-35), 2.94 – 2.78 (m, *J* = 6.9 Hz, 3H, C*H*-*i*Pr), 2.12 (m, 1.4 Hz, 2H, *H*-24), 1.87 – 1.69 (m, 2H, *H*-34), 1.50 – 1.26 (m, 12H, *H*-26, *H*-27, *H*-28, *H*-30, *H*-31 & *H*-32), 1.26 – 1.14 (m, 54H, CH_3-*i*Pr & CH_3-Bpin), 1.13 – 0.87 (m, 3H, *H*-25 *H*-29 & *H*-33). See spectrum.

^{13}C NMR (101 MHz, $CDCl_3$) δ 171.09 (*C*O-benzoate), 150.03 (*C*-TIB-*para*), 144.88 (*C*-TIB-*ortho*), 138.94 (*C*H-22), 131.02 (*C*-TIB-*ipso*), 120.90 (*C*H-TIB-*meta*), 114.76 (*C*H_2-23), 83.13 (*C*-Bpin), 83.01 (*C*-Bpin), 82.85 (*C*-Bpin), 64.88 (*C*-35), 35.58 (*C*H_2-24), 34.58 (*C*H-*i*Pr *para*), 31.75 (*C*H_2-26 *or* *C*H_2-28 *or* *C*H_2-30 *or* *C*H_2-32), 31.66 (*C*H_2-26 *or* *C*H_2-28 *or* *C*H_2-30 *or* *C*H_2-32), 31.62 (*C*H_2-26 *or* *C*H_2-28 *or* *C*H_2-30 *or* *C*H_2-32), 31.56 (*C*H-*i*Pr *ortho*),

31.31 (CH_2-26 *or* CH_2-28 *or* CH_2-30 *or* CH_2-32), 30.13 (CH_2-34), 28.81 (CH_2-27 *or* CH_2-31), 28.80 (CH_2-27 *or* CH_2-31), 25.00 (CH_3-Bpin), 24.98 (CH_3-Bpin), 24.96 (CH_3-), 24.89 (CH_3-Bpin), 24.33 (CH_3-*i*Pr), 24.31 (CH_3-*i*Pr), 24.12 (CH_3-*i*Pr). See spectrum.

carbon next to boron (*C*-25, *C*-29 & *C*-33) *not observed due to quadrupolar relaxation*

HRMS (m/z): (MALDI) calculated for $C_{48}H_{83}B_3O_8$ [M+Na]+ 843.6282, found 843.6298.

TLC: R_f = 0.23 (90:10 hexane/EtOAc, stained with *p*-anisaldehyde).

IR (ν_{max}/cm^{-1}, neat): 2962, 2930, 2870, 1722 (C=O), 1606, 1462, 1369, 1312, 1251, 1138, 1076.

$[\alpha]_D^{25}$: −6 (*c* = 1, $CHCl_3$).

(3*R*,7*R*,11*R*)-3,7,11,14-Tetrakis(4,4,5,5-tetramethyl-1,3,2-dioxaborolan-2-yl)tetradecyl 2,4,6-triisopropylbenzoate (F3)

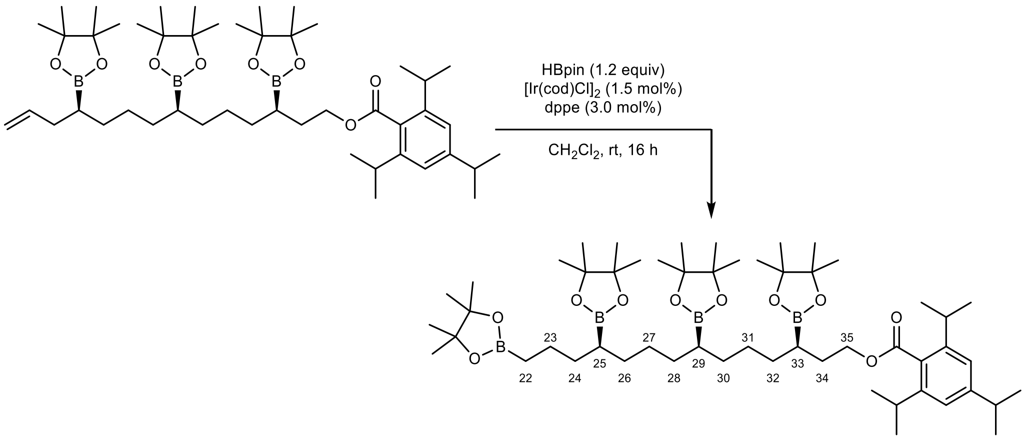

Prepared following General Procedure 3 using pinacolborane (463 mg, 524 µL, 3.61 mmol), alkene **15** (720 mg, 0.878 mmol), $[Ir(cod)Cl]_2$ (8.8 mg, 13.2 µmol) and 1,2-bis(diphenylphosphino)ethane dppe (10.5 mg, 26.3 µmol). The crude mixture was purified by normal phase flash column chromatography (Biotage HC-50 g, pentane: Et_2O 10-35%) to afford the title compound (715 mg, 86%) as a colourless oil.

^{1}H NMR (400 MHz, $CDCl_3$) δ 6.98 (s, 2H, *H*-Ar), 4.29 (t, *J* = 7.0 Hz, 2H, *H*-35), 2.95 – 2.76 (m, *J* = 6.9 Hz, 3H, C*H*-*i*Pr), 1.78 (m, 2H, CH_2-34), 1.52 – 1.26 (m, 15H, CH_2-23, 24, 26, 27, 28, 30, 31 & 32), 1.26 – 1.14 (m, 66H, CH_3-*i*Pr & CH_3-Bpin), 1.14 – 0.92 (m, 3H, C*H*-25, 29 & 33), 0.79 – 0.67 (m, 2H, C*H*-22).

See spectrum.

^{13}C NMR (101 MHz, $CDCl_3$) δ 171.09 (*C*O-benzoate), 150.02 (*C*-TIB-*para*), 144.89 (*C*-TIB-*ortho*), 131.03 (*C*-TIB-*ipso*), 120.89 (*C*H-TIB-*meta*), 83.12 (*C*-Bpin), 82.88 (*C*-Bpin), 82.82 (*C*-Bpin), 82.80 (*C*-Bpin), 64.89 (*C*-35), 34.58 (*C*H-*i*Pr *para*), 34.32 (*C*H_2-24), 31.81 (2*C*H_2-26, 28, 30 *or* 32), 31.75 (*C*H_2-26, 28, 30 *or* 32), 31.65 (*C*H_2-26, 28, 30 *or* 32), 31.55 (*C*H-*i*Pr *ortho*), 30.14 (*C*H_2-34), 29.09 (*C*H_2-27 *or* 31), 28.82 (*C*H_2-27 *or* 31) , 24.99 (*C*H_3-Bpin), 24.97 (*C*H_3-Bpin), 24.89 (*C*H_3-Bpin), 24.33 (*C*H_3-*i*Pr), 24.31 (*C*H_3-*i*Pr), 24.12 (*C*H_3-*i*Pr), 23.84 (*C*H_2-23). See spectrum.

carbon next to boron (*C*-22, *C*-25, *C*-29 & *C*-33) *not observed due to quadrupolar relaxation*

HRMS (m/z): (MALDI) calculated for $C_{54}H_{96}B_4O_{10}$ [M+Na]+ 971.7299, found 971.7306.

TLC: $\boldsymbol{R_f}$ = 0.31 (90:10 hexane/EtOAc, stained with *p*-anisaldehyde).

IR (ν_{max}/cm^{-1}, neat): 2975, 2925, 2867, 1725 (C=O), 1606, 1462, 1379, 1315, 1251, 1144, 1076, 968.

$[\alpha]_D^{25}$: −6 (*c* = 1, $CHCl_3$).

4.4 Fragment 4

Ethyl 2,4,6-triisopropylbenzoate (S10)

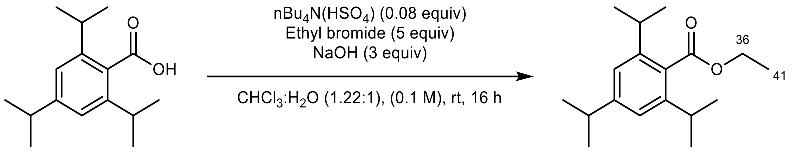

S10

According to a literature procedure[13] biphasic mixture of 2,4,6-triisopropylbenzoic acid (17.4 g, 70.0 mmol, 1.00 equiv.), tetrabutylammonium hydrogen sulphate (1.90 g, 5.60 mmol, 8.00 mol%), sodium hydroxide (8.40 g, 210 mmol, 3.00 equiv.),and ethyl bromide (38.1 g, 26.1 ml, 350 mmol, 5.00 equiv.) in chloroform (350 ml) and water (286 ml) was stirred vigorously for 16 h at room temperature. The layers were separated and the aqueous phase was extracted with CH_2Cl_2 (3 × 50 ml). The combined organic layers were washed with brine (150 ml), dried over anhydrous $MgSO_4$ and then concentrated under reduced pressure to give the title compound (17.9 g, 93%) as a colourless oil.

All recorded spectroscopic data matched that previously reported[13]

^{1}**H NMR** (400 MHz, $CDCl_3$) δ 7.01 (s, 2H, *H*-Ar), 4.38 (q, *J* = 7.1 Hz, 2H, *H*-36), 2.95 – 2.81 (m, 3H, C*H*-*i*Pr), 1.37 (t, *J* = 7.1 Hz, 3H, *H*-41), 1.25 (dd, *J* = 6.9, 3.1 Hz, 18H, CH_3-*i*Pr). See spectrum.

^{13}**C NMR** (101 MHz, $CDCl_3$) δ 170.97 (*C*O-benzoate), 150.21 (*C*-*para*), 144.87 (*C*-*ortho*), 130.78 (*C*-*ipso*), 120.97 (*C*H-*meta*), 60.90 (CH_2-36), 34.58 (*C*H-*i*Pr *para*), 31.57 (*C*H-*i*Pr *ortho*), 24.25 (CH_3-*i*Pr *ortho*), 24.10 (CH_3-*i*Pr *para*), 14.40 (*C*-41). See spectrum.

(*S*)-1-(Trimethylstannyl)ethyl 2,4,6-triisopropylbenzoate ((*S*)-16)

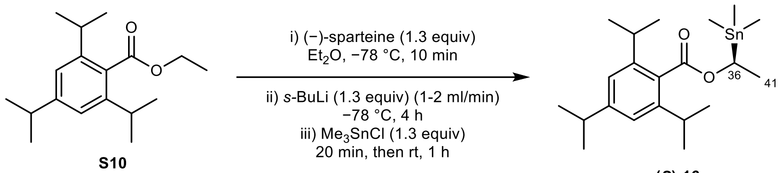

S10 **(*S*)-16**

According to a literature procedure[14] to a solution of ethyl 2,4,6-triisopropylbenzoate (10.0 g, 36.2 mmol, 1.00 equiv.) and (−)-sparteine (11.0 g, 10.8 mL, 47.0 mmol, 1.50 equiv.) in diethyl ether (181 mL, 0.30 M), *s*-BuLi (1.30 M solution in hexanes, 36.2 mL, 47.0 mmol, 1.30 equiv.) was added dropwise at −78 °C and the reaction mixture was stirred at same temperature for 3 h. Trimethyl tin chloride (1.00 M in hexane, 47.0 mL, 47.0 mmol, 1.30 equiv.) was added dropwise (1-2 mL/min) to the reaction mixture at −78 °C, the reaction mixture was stirred for 0.5 h at −78 °C and then it was warmed to room temperature and stirred for 1 h. The reaction was quenched by the addition of 2 M HCl aq. and extracted with EtOAc. The organic layer was extracted with 2M HCl aq. (3x). The organic layer was concentrated under reduced pressure and the crude residue was purified by recrystallisation from MeOH (3x) to afford the title compound (9.07 g, 57%, 99.9:0.1 *e.r.*) as a white crystalline solid.

All recorded spectroscopic data matched that previously reported[14]

^{1}**H NMR** (400 MHz, $CDCl_3$) δ 6.99 (s, 2H, *H*-Ar), 5.04 (q, *J* = 7.6 Hz, 1H, *H*-36), 2.87 (m, 3H, C*H*-*i*Pr), 1.59 (d, *J* = 7.6 Hz, 3H, *H*-41), 1.24 (d, *J* = 6.9 Hz, 18H, CH_3-*i*Pr), 0.18 (s, 9H, H_3-SnC). *Sn satellite peaks ignored for simplicity.* See spectrum.

^{13}**C NMR** (101 MHz, $CDCl_3$) δ 171.30 (*C*O-benzoate), 149.93 (*C*-*para*), 144.83 (*C*-*ortho*), 130.80 (*C*-*ipso*), 120.80 (*C*H-*meta*), 67.04 (*C*H-36), 34.41 (*C*H-*i*Pr *para*), 31.36 (*C*H-*i*Pr *ortho*), 24.35 (CH_3-*i*Pr *ortho*), 24.10 (CH_3-*i*Pr *ortho*), 23.96 (CH_3-*i*Pr *para*), 19.22 (*C*-41), -9.89 (*C*Sn). See spectrum.

IR (ν_{max}/cm^{-1}, neat): 2978, 2956, 2926, 2872, 2857, 1463, 1370, 1312, 1229, 1141, 859.

$[\boldsymbol{\alpha}]_{\mathbf{D}}^{\mathbf{25}}$: +35 ($c$ = 1, $CHCl_3$).

M.P: 65-66 °C (MeOH)

HPLC on chiral stationary phase Daicel Chiralpak-IB column (25 cm), 100% *n*-hexane, flow rate: 0.9 mL/min., rt, 230 nm, Retention times: 9.00 min. (S, major) and 11.58 min. (R, minor).

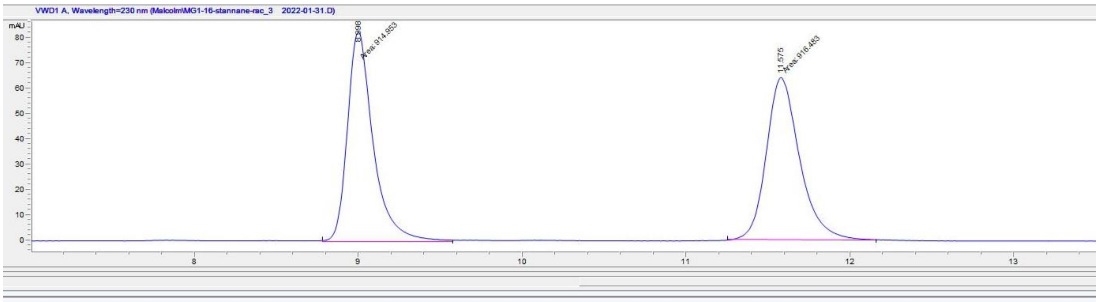

#	Time	Type	Area	Height	Width	Area%	Symmetry
1	8.998	MM	915	82.8	0.1842	49.958	0.685
2	11.575	MM	916.5	64.3	0.2376	50.042	0.768

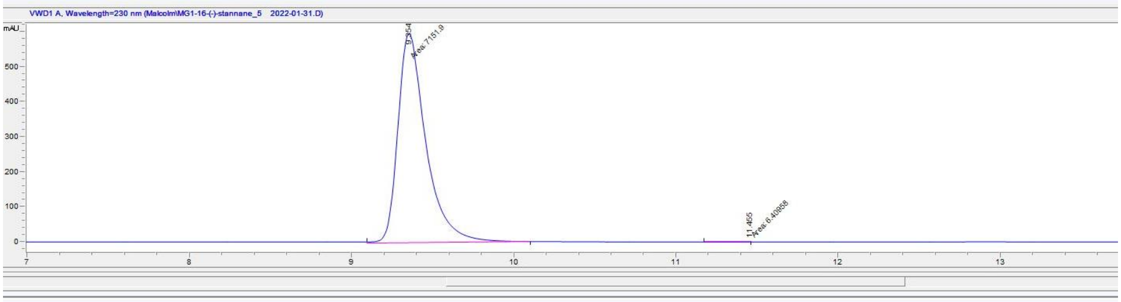

#	Time	Type	Area	Height	Width	Area%	Symmetry
1	9.354	MM	7151.9	594.2	0.2006	99.910	0.583
2	11.455	MM	6.4	7E-1	0.1529	0.090	2.88E-3

(*R*)-2-(Hexan-2-yl)-4,4,5,5-tetramethyl-1,3,2-dioxaborolane ((*R*)-F4)

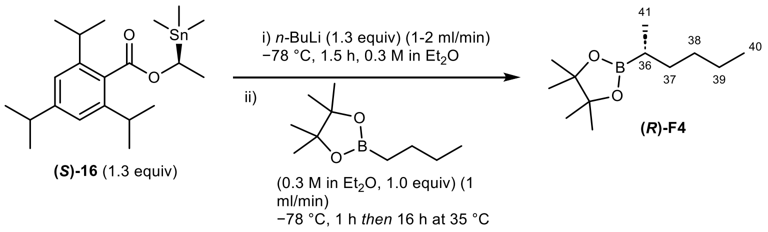

*n*BuLi (1.30 M in hexanes, 11.0 mL, 17.7 mmol, 1.30 equiv.) was added dropwise to a solution of stannane (7.75 g, 17.7 mmol, 1.30 equiv.) in Et_2O (0.30 M) at −78 °C and the reaction mixture was stirred 1.5 h at the same temperature. A solution of commercially available n-butylboronic acid pinacol ester (2.50 g, 2.87 mL, 13.6 mmol, 1.00 equiv.) in Et_2O (0.30 M) was added dropwise (1.00 mL/min) to the reaction mixture at −78 °C and the

resulting mixture was stirred 1 h at −78 °C, then it was warmed up to room temperature and heated 16 h at 35 °C. Diethyl ether and water were added to the mixture. The organic layer was washed with water (3x) and dried over anhydrous $MgSO_4$. A portion of the material was purified by normal phase preparative HPLC eluting with hexane:ethyl acetate (0-3%) to afford the title compound as a colourless oil.

^{1}H NMR (400 MHz, $CDCl_3$) δ 1.51 – 1.39 (m, 1H, *H*-37'), 1.32 – 1.24 (m, 5H, C*H*$_2$-38, C*H*$_2$-39 & *H*-37'), 1.23 (s, 12H, C*H*$_3$-Bpin), 1.04 – 0.92 (m, 4H, C*H*-36 & C*H*$_3$-41), 0.90 – 0.84 (m, 3H, C*H*$_3$-40). See spectrum.

^{13}C NMR (101 MHz, $CDCl_3$) δ 82.89 (*C*-Bpin), 33.07 (*C*H$_2$-37), 31.40 (*C*H$_3$-38), 24.90 (C*H*$_3$-Bpin), 24.87 (C*H*$_3$-Bpin), 23.06 (*C*H$_3$-39), 15.66 (*C*H$_3$-41), 14.27 (*C*H$_3$-40). See spectrum.

HRMS (m/z): (EI) calculated for $C_{12}H_{25}BO_2$ [M]+ 212.1942, found 212.1939.

$[\alpha]_D^{25}$: −2 (*c* = 1, $CHCl_3$).

(*R*)-1-(Trimethylstannyl)ethyl 2,4,6-triisopropylbenzoate ((*R*)-16)

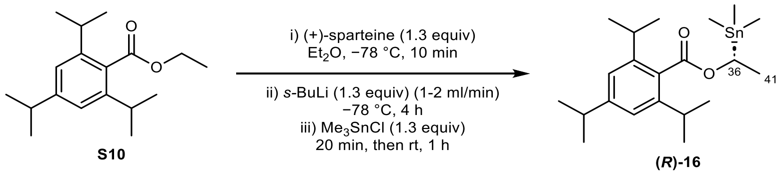

Prepared following the same procedure as described for (*S*) enantiomer **(*S*)-16** using ethyl 2,4,6-triisopropylbenzoate **S10** (5.00 g, 18.1 mmol), (+)-sparteine (5.51 g, 5.40 mL, 23.5 mmol), trimethyltin chloride (1 M in hexanes) (23.5 mL, 23.5 mmol), *s*-BuLi (1.30 M in hexanes) (18.1 mL, 23.5 mmol) in Et_2O (90 mL). The crude mixture was purified by recrystallisation from MeOH (3x) to afford the title compound (4.97 g, 63%, 99.9:0.1 *e.r.*) as a white crystalline solid.

Both ^{1}H and ^{13}C spectroscopic data was identical to that of it's enantiomer **(*S*)-16**

$[\alpha]_D^{25}$: −35 (*c* = 1, $CHCl_3$).

M.P: 65-66 °C (MeOH)

HPLC on chiral stationary phase Daicel Chiralpak-IB column (25 cm), 100% *n*-hexane, flow rate: 0.9 mL/min., rt, 230 nm, Retention times: 9.00 min. (S, major) and 11.58 min. (R, minor).

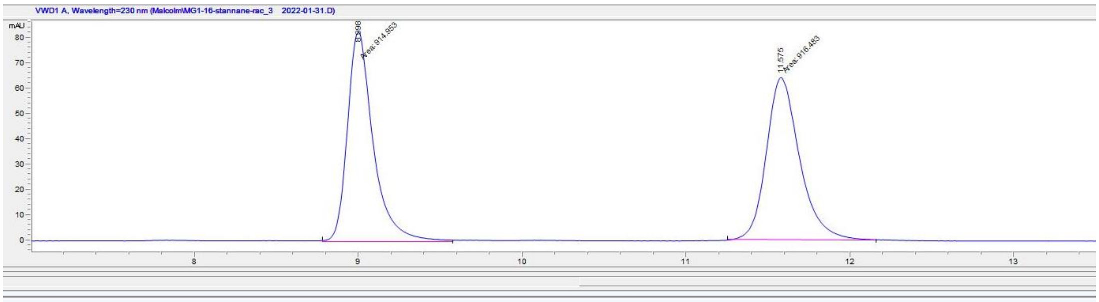

#	Time	Type	Area	Height	Width	Area%	Symmetry
1	8.998	MM	915	82.8	0.1842	49.958	0.685
2	11.575	MM	916.5	64.3	0.2376	50.042	0.768

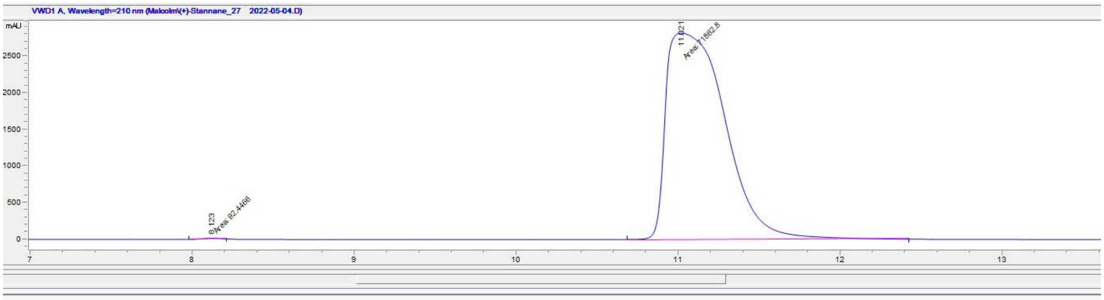

#	Time	Type	Area	Height	Width	Area%	Symmetry
1	8.123	MM	92.4	13.6	0.1134	0.128	1.466
2	11.021	MM	71862.8	2833	0.4228	99.872	0.348

(*S*)-2-(Hexan-2-yl)-4,4,5,5-tetramethyl-1,3,2-dioxaborolane ((*S*)-F4)

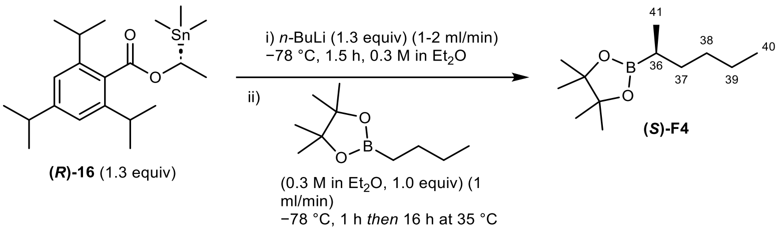

Prepared following the same procedure as described for (*R*) enantiomer **(*R*)-F4** using stannane **(*R*)-16** (4.65 g, 10.6 mmol), n-butylboronic acid pinacol ester (1.50 g, 1.72 mL, 8.15 mmol), *n*-BuLi (1.6 M in hexanes) (6.62 mL, 10.6 mmol).

Both ^{1}H and ^{13}C spectroscopic data was identical to that of it's enantiomer **(*R*)-F4**

HRMS (m/z): (EI) calculated for $C_{12}H_{25}BO_2$ [M]+ 212.1942, found 212.1939.

$[\alpha]_D^{25}$: 2 (c = 1, $CHCl_3$).

4.5 Caylobolide A (3*R*, 36*S*/*R*) fragment couplings

2-((6S)-6-((4R,8R,12R,14S)-4-hydroxy-14-methyl-8,12-bis((triethylsilyl)oxy)heptadec-16-en-1-yl)-2,2-dimethyl-1,3-dioxan-4-yl)ethyl 2,4,6-triisopropylbenzoate ((3*S*)-S12)

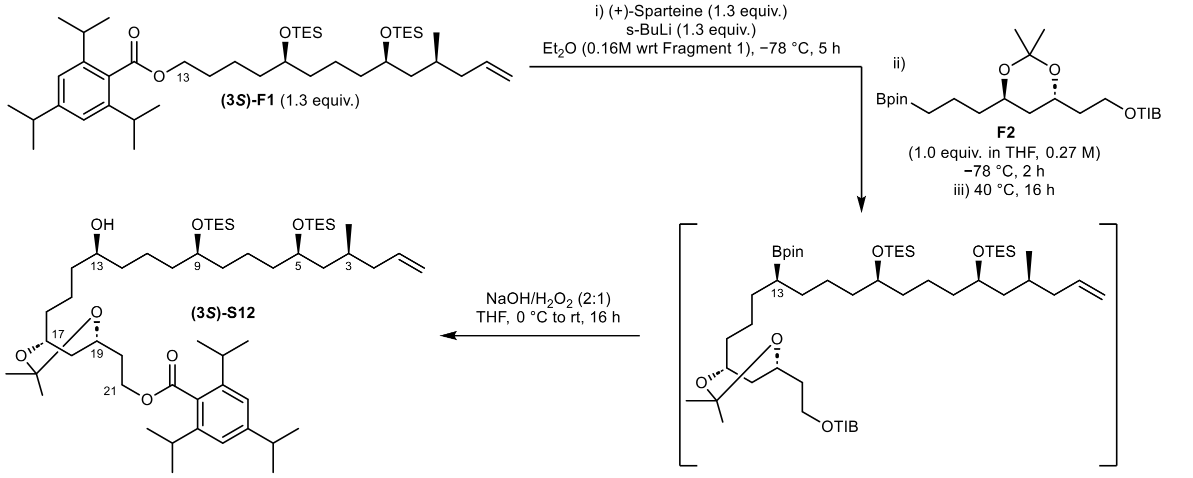

To a solution of **(3*S*)-F1** (417 mg, 0.582 mmol, 1.30 equiv.) and (+)-sparteine (123 mg, 121 µL, 0.58 mmol, 1.30 equiv.) in diethyl ether (3.6 mL, 0.16 M), *s*-BuLi (1.42 M in hexanes) (410 µL, 0.582 mmol, 1.30 equiv.) was added dropwise at −78 °C and the reaction mixture was stirred at same temperature for 5 h. A solution of **F2** in THF (0.27 M, 1.65 mL) (250 mg, 0.448 mmol) was added dropwise (1 mL/min) to the reaction mixture at −78 °C, the reaction mixture was stirred for 2 h at −78 °C after which time the reaction mixture was heated to 40 °C for 16 h. A 2:1 solution of aqueous sodium hydroxide (3 M) and hydrogen peroxide (30% aqueous solution) was added directly to the reaction mixture at 0 °C, and the reaction mixture then stirred at rt for 16 h. The reaction mixture was quenched by the addition of saturated sodium thiosulfate and was extracted with Et_2O (3 x 20 mL). The organic layers were combined, and washed with brine, then dried over anhydrous Na_2SO_4 and concentrated under reduced pressure. The crude residue was purified by automated flash column chromatography (Biotage HC-10 g, pentane: ethyl acetate 0-20%) to afford the title compound (376 mg, 92%) as a colourless oil.

^{1}H NMR (600 MHz, $CDCl_3$) δ 7.00 (s, 2H, *H*-Ar), 5.76 (ddt, *J* = 16.1, 11.3, 7.1 Hz, 1H, *H*-1), 5.04 – 4.96 (m, 2H, CH_2 alkene), 4.45 – 4.32 (m, 2H, *H*-21), 3.99 – 3.91 (m, 1H, *H*-19), 3.83 – 3.76 (m, 1H, *H*-17), 3.76 – 3.69 (m, 1H, *H*-5), 3.69 – 3.62 (m, 1H, *H*-9), 3.62 – 3.54 (m, 1H, *H*-13), 2.86 (m, 3H, C*H*-*i*Pr), 2.10 (ddd, *J* = 13.7, 6.9, 5.3 Hz, 1H, *H*-2'), 1.95 – 1.80 (m, 3H, *H*-2'' & *H*-21), 1.67 – 1.36 (m, 22H, CH_2-alkyls), 1.35 (s, 3H, CH_3-acetonide), 1.33

(s, 3H, CH_3-acetonide), 1.32 – 1.26 (m, 3H, CH_2-alkyls), 1.24 (dd, J = 7.0, 1.4 Hz, 18H, CH_3-*i*Pr), 0.96 (t, J = 7.9, 18H, CH_3-OTES), 0.88 (d, J = 6.7 Hz, 3H, CH_3-42), 0.59 (q, J = 8.0, 12H, CH_2-OTES). See spectrum.

^{13}C NMR (151 MHz, $CDCl_3$) δ 171.06 (*C*O-benzoate), 150.29 (*C-para*), 144.86 (*C-ortho*), 137.35 (*C*H-1), 130.65 (*C-ipso*), 121.00 (*C-meta*), 115.99 (*C*H_2- alkene), 100.54 (CO anti-acetonide), 72.40 (*C*H-9), 71.89 (*C*H-13), 70.54 (*C*H-5), 66.76 (*C*H-17), 63.64 (*C*H-19), 61.86 (*C*H-21), 44.49 (*C*H-4), 41.59 (*C*H-2), 38.85 (*C*H-18), 37.92 (*C*H_2-6 *or* CH_2-8, *or* CH_2-10 *or* CH_2-12 *or* CH_2-14 *or* CH_2-16), 37.74 (*C*H_2-6 *or* CH_2-8, *or* CH_2-10 *or* CH_2-12 *or* CH_2-14 *or* CH_2-16), 37.64 (*C*H_2-6 *or* CH_2-8, *or* CH_2-10 *or* CH_2-12 *or* CH_2-14 *or* CH_2-16), 37.39 (*C*H_2-6 *or* CH_2-8, *or* CH_2-10 *or* CH_2-12 *or* CH_2-14 *or* CH_2-16), 37.27 (*C*H_2-6 *or* CH_2-8, *or* CH_2-10 *or* CH_2-12 *or* CH_2-14 *or* CH_2-16), 35.94 (*C*H_2-6 *or* CH_2-8, *or* CH_2-10 *or* CH_2-12 *or* CH_2-14 *or* CH_2-16), 35.03 (*H*-20), 34.57 (*C*H-*i*Pr *para*), 31.66 (*C*H-*i*Pr *ortho*), 29.48 (*C*H-3), 24.85 (*C*H_3-*anti*-acetonide), 24.79 (*C*H_3-*anti*-acetonide), 24.36 (*C*H_3-*i*Pr), 24.24 (*C*H_3-*i*Pr), 24.09 (*C*H_3-*i*Pr), 21.83 (*C*H_2-7 *or* CH_2-11, *or* CH_2-15), 21.55 (*C*H_2-7 *or* CH_2-11, *or* CH_2-15), 21.18 (*C*H_2-7 *or* CH_2-11, *or* CH_2-15), 20.07 (*C*H_3-42), 7.13 (OTES-*C*H_3), 7.12 (OTES-*C*H_3), 5.34 (OTES-*C*H_2), 5.28 (OTES-*C*H_2). See spectrum.

HRMS (m/z): (ESI) calculated for $C_{54}H_{100}O_7Si_2$ [M+H]+ 917.7080, found 917.7064.

TLC: R_f Bpin = 0.19 (95:5 hexane/EtOAc, stained with *p*-anisaldehyde), R_f product (OH) = 0.17 (90:10 hexane/EtOAc, stained with *p*-anisaldehyde).

IR (ν_{max}/cm^{-1}, neat): 3456 (OH), 2956, 2937, 2875, 1728 (C=O), 1607, 1460, 1378, 1227, 1137, 1055, 1033, 741.5.

$[\alpha]_D^{25}$: −2 (c = 1.3, $CHCl_3$).

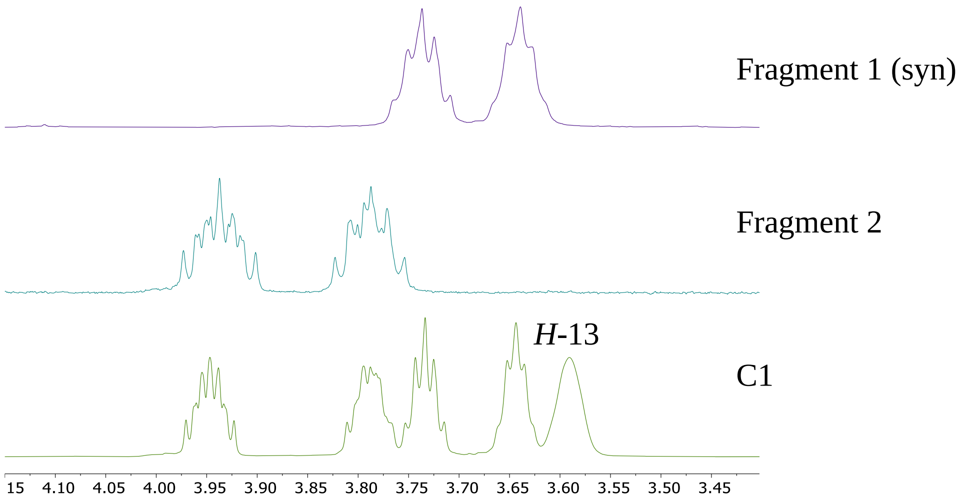

2-((6S)-2,2-dimethyl-6-((4R,8R,12R,14S)-14-methyl-4,8,12-tris((triethylsilyl)oxy)heptadec-16-en-1-yl)-1,3-dioxan-4-yl)ethyl 2,4,6-triisopropylbenzoate ((3S)-CF_1-2)

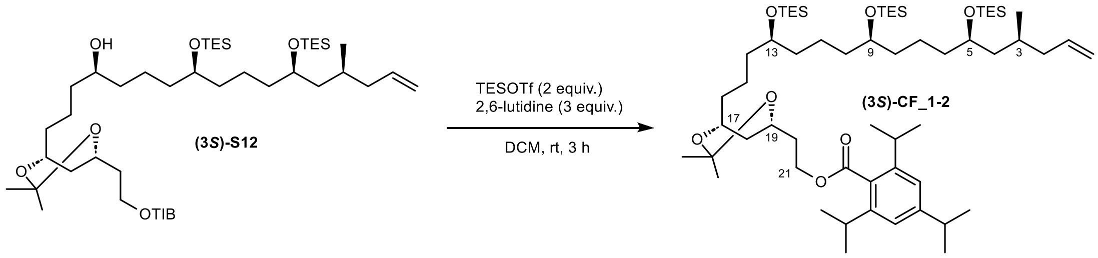

Prepared following General Procedure 5 using alcohol **(3S)-S12** (350 mg, 0.381 mmol), TESOTf (202 mg, 0.763 mmol, 173 µL), 2,6-lutidine (121 mg, 1.14 mmol, 131 µL). The crude mixture was purified by normal phase flash column chromatography (Biotage HC-10 g, pentane: EtOAc 0-10%) to afford the title compound (393 mg, quant.) as a colourless oil.

^{1}H NMR (600 MHz, CDCl$_3$) δ 7.00 (s, 2H, *H*-Ar), 5.83 – 5.70 (m, 1H, *H*-1), 5.08 – 4.95 (m, 2H, C*H*$_2$ alkene), 4.38 (m, 2H, *H*-21), 4.00 – 3.89 (m, 1H, *H*-19), 3.77 (m, 1H, *H*-17), 3.73 (m, 1H, *H*-5), 3.62 (m, 2H, *H*-9 & *H*-13), 2.86 (m, 3H, C*H*-*i*Pr), 2.10 (m, 1H, *H*-2’), 1.94 – 1.81 (m, 3H, *H*-2’’ & *H*-21), 1.66 – 1.56 (m, 3H, *H*-3 & *H*-18), 1.53 – 1.35 (m, 18H, C*H*$_2$-alkyl), 1.34 (s, 3H, C*H*$_3$-acetonide), 1.33 (s, 3H, C*H*$_3$-acetonide) 1.29 (m, 5H, C*H*$_2$-alkyls), 1.26 – 1.22 (m, 18H, C*H*$_3$-*i*Pr), 1.00 – 0.92 (m, 27H, C*H*$_3$-OTES), 0.91 – 0.86 (m, 3H, C*H*$_3$-42), 0.59 (m, 18H, C*H*$_2$-OTES). See spectrum.

^{13}C NMR (151 MHz, CDCl$_3$) δ 171.08 (*C*O-benzoate), 150.29 (*C*-*para*), 144.87 (*C*-*ortho*), 137.37 (*C*H-1), 130.67 (*C*-*ipso*), 121.00 (*C*-*meta*), 115.98 (*C*H$_2$- alkene), 100.50 (CO anti-acetonide), 72.49 (*C*H-9 *or* *C*H-13), 72.47 (*C*H-9 *or* *C*H-13), 70.55 (*C*H-5), 66.72 (*C*H-17),

63.64 (*C*H-19), 61.90 (*C*H-21), 44.51 (*C*H-4), 41.58 (*C*H-2), 38.89 (*C*H-18), 37.80 (CH_2-6 *or* CH_2-8, *or* CH_2-10 *or* CH_2-12 *or* CH_2-14 *or* CH_2-16), 37.72 (CH_2-6 *or* CH_2-8, *or* CH_2-10 *or* CH_2-12 *or* CH_2-14 *or* CH_2-16), 37.66 (CH_2-6 *or* CH_2-8, *or* CH_2-10 *or* CH_2-12 *or* CH_2-14 *or* CH_2-16), 37.22 (CH_2-6 *or* CH_2-8, *or* CH_2-10 *or* CH_2-12 *or* CH_2-14 *or* CH_2-16), 36.25 (CH_2-6 *or* CH_2-8, *or* CH_2-10 *or* CH_2-12 *or* CH_2-14 *or* CH_2-16), 35.06 (CH_2-20), 34.58 (*C*H-*i*Pr *para*), 31.67 (*C*H-*i*Pr *ortho*), 29.48 (*H*-3), 24.84 (CH_3-*anti*-acetonide), 24.72 (CH_3-*anti*-acetonide), 24.36 (CH_3-*i*Pr), 24.25 (CH_3-*i*Pr), 24.10 (CH_3-*i*Pr), 21.65 (CH_2-7 *or* CH_2-11, *or* CH_2-15), 21.42 (CH_2-7 *or* CH_2-11, *or* CH_2-15), 21.22 (CH_2-7 *or* CH_2-11, *or* CH_2-15), 20.08 (CH_3-42), 7.13 (OTES-CH_3), 7.11 (OTES-CH_3), 5.35 (OTES-CH_2), 5.30 (OTES-CH_2), 5.27 (OTES-CH_2). See spectrum.

HRMS (m/z): (MALDI) calculated for $C_{60}H_{114}O_7Si_3$ [M+Na]+ 1053.7765, found 1053.7751.

TLC: R_f = 0.51 (85:15 hexane/EtOAc, stained with ceric ammonium molybdate (CAM))

IR (ν_{max}/cm^{-1}, neat): 2955, 2936, 2912, 2876, 1728 (C=O), 1607, 1460, 1379, 1240, 1136, 1076, 1007, 726.

$[\alpha]_D^{25}$: +12 (c = 1.6, $CHCl_3$).

(1R)-2-((6S)-2,2-dimethyl-6-((4R,8R,12R,14S)-14-methyl-4,8,12-tris((triethylsilyl)oxy)heptadec-16-en-1-yl)-1,3-dioxan-4-yl)-1-(p-tolylsulfinyl)ethyl 2,4,6-triisopropylbenzoate ((3*S*)-17)

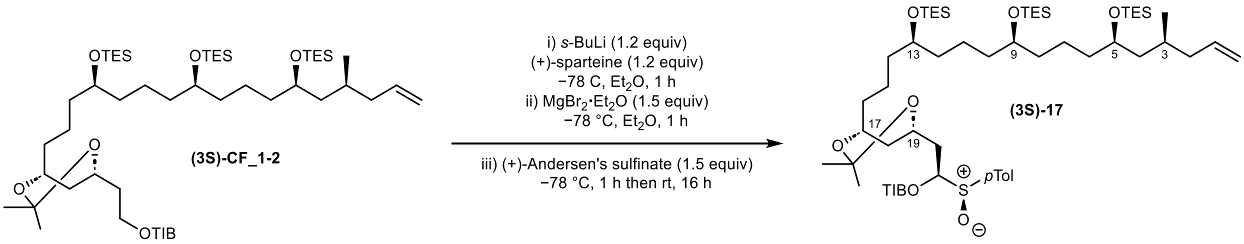

Prepared following General Procedure 1 using **(3S)-CF_1-2** (300 mg, 0.291 mmol), (+)-sparteine (82 mg, 80 µL, 0.35 mmol), *s*-BuLi (1.29 M in hexanes) (270 µL, 0.349 mmol), (+)-Andersen's sulfinate (128 mg, 0.436 mmol), magnesium turnings (57 mg, 2.30 mmol), dibromoethane (0.16 g, 76 µL, 0.87 mmol), Et_3N (44 mg, 60 µL, 0.44 mmol), trimethylsilyl chloride (41 mg, 48 µL, 0.38 mmol). The crude residue was purified by normal phase preparative HPLC (hexane: EtOAc 0-20%) to afford the title compound (158 mg, 46%) as a colourless oil.

^{1}H NMR (600 MHz, $CDCl_3$) δ 7.66 (d, *J* = 8.2 Hz, 2H, *H*-Ar), 7.38 – 7.33 (m, 2H, *H*-Ar), 7.05 (s, 2H, *H*-Ar), 5.80 (dd, *J* = 6.7, 5.0 Hz, 1H, *H*-21), 5.78 – 5.71 (m, 1H, *H*-1), 5.02 – 4.96 (m, 2H *H*-alkene), 3.77 – 3.70 (m, 1H, *H*-5), 3.62 (m, 3H, *H*-9 & *H*-13 & *H*-17), 3.50 (m, 1H, *H*-19), 2.94 (m, 3H, C*H*-*i*Pr), 2.42 (s, 3H, CH_3-tolyl), 2.16 – 2.07 (m, 2H, *H*-20' & *H*-2'), 1.98 (ddd, *J* = 14.8, 8.0, 6.7 Hz, 1H, *H*-20''), 1.89 – 1.82 (m, 1H, *H*-2''), 1.65 – 1.57 (m, 1H, *H*-3), 1.54 – 1.31 (m, 20H, -CH_2), 1.33 – 1.24 (m, 27H, CH_3-iPr & -CH_2), 1.22 (s, 3H, CH_3-acetonide), 1.00 (s, 3H, CH_3-acetonide), 0.98 – 0.92 (m, 27H, CH_3-OTES), 0.88 (d, *J* = 6.7 Hz, 3H, *H*-42), 0.63 – 0.54 (m, 18H, CH_2-OTES). See spectrum.

^{13}C NMR (151 MHz, $CDCl_3$) δ 170.22 (*C*O-benzoate), 151.04 (*C*H-TIB-*para*), 145.45 (*C*H-TIB-*ortho*), 141.68 (*C*-tolyl-*ipso*), 137.57 (*C*-tolyl-*para*), 137.30 (*C*H-1), 130.19 (*C*H-tolyl-*meta*), 128.82 (*C*H-TIB-*ipso*), 124.41 (*C*H-tolyl-*ortho*), 121.13 (TIB-*C*H-*meta*), 115.96 (CH_2-alkene), 100.42 (CO *anti*-acetonide), 90.10 (*C*H-21), 72.45 (*C*H-9 *or* *C*H-13), 72.40 (*C*H-9 *or* *C*H-13), 70.52 (*C*H-5), 66.42 (*C*H-17), 63.90 (*C*H-19), 44.47 (*C*H-4), 41.55 (*C*H-2), 38.58 (CH_2-6 *or* CH_2-8, *or* CH_2-10 *or* CH_2-12 *or* CH_2-14 *or* CH_2-16), 37.77 (CH_2-6 *or* CH_2-8, *or* CH_2-10 *or* CH_2-12 *or* CH_2-14 *or* CH_2-16), 37.76 (CH_2-6 *or* CH_2-8, *or* CH_2-10 *or* CH_2-12 *or* CH_2-14 *or* CH_2-16), 37.69 (CH_2-6 *or* CH_2-8, *or* CH_2-10 *or* CH_2-12 *or* CH_2-14 *or* CH_2-16), 37.61 (CH_2-6 *or* CH_2-8, *or* CH_2-10 *or* CH_2-12 *or* CH_2-14 *or* CH_2-16), 37.17 (CH_2-6 *or* CH_2-8, *or* CH_2-10 *or* CH_2-12 *or* CH_2-14 *or* CH_2-16), 36.15 (CH_2-6 *or* CH_2-8, *or* CH_2-10 *or* CH_2-12 *or* CH_2-14 *or* CH_2-16), 34.59 (*C*H-*i*Pr *para*), 31.65 (*C*H-*i*Pr *ortho*), 30.26 (*C*H-2), 29.46 (*C*-3), 24.49 (CH_3-*i*Pr), 24.46 (CH_3-*i*Pr), 24.38 (CH_3-*i*Pr), 24.32 (CH_3-*i*Pr), 24.03 (CH_3--*i*Pr), 21.62 (CH_2-7 *or* CH_2-11, *or* CH_2-15), 21.53 (CH_3-tolyl), 21.37 (CH_2-7 *or* CH_2-11, *or* CH_2-15), 21.17 (CH_2-7 *or* CH_2-11, *or* CH_2-15), 20.05 (CH_3-42), 7.10 (CH_3-OTES), 7.07 (CH_3-OTES), 5.32 (CH_2-OTES), 5.27 (CH_2-OTES), 5.23 (CH_2-OTES).

See spectrum.

HRMS (m/z): (nanospray) calculated for $C_{67}H_{120}O_8SSi_3$ [M+Na]+ 1191.7904, found 1191.7863.

TLC: $\boldsymbol{R_f}$ = 0.06 (9:1 hexane/EtOAc, stained with *p*-anisaldehyde)

IR (ν_{max}/cm^{-1}, neat): 2955, 2935, 2913, 2875, 1744 (C=O), 1607, 1459, 1379, 1229, 1039 (S=O), 1006 (Si-O), 724 (Si-C).

$[\alpha]_D^{25}$: −30 (*c* = 1, $CHCl_3$).

(3R,7R,11S,15R)-16-((4R,6S)-2,2-dimethyl-6-((4R,8R,12R,14S)-14-methyl-4,8,12-tris((triethylsilyl)oxy)heptadec-16-en-1-yl)-1,3-dioxan-4-yl)-3,7,11,15-tetrakis(4,4,5,5-tetramethyl-1,3,2-dioxaborolan-2-yl)hexadecyl 2,4,6-triisopropylbenzoate ((3*S*)-S13)

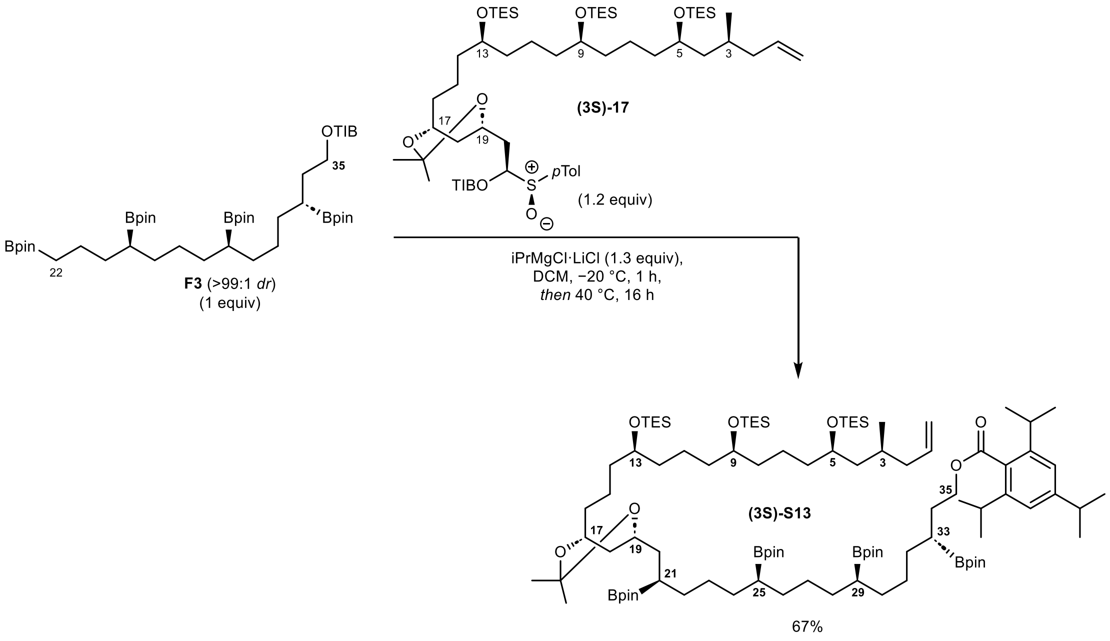

Prepared following General Procedure 2 using **F3** (100 mg, 0.105 mmol), **(3S)-17** (148 mg, 0.127 mmol), *i*PrMgCl·LiCl (1.30 M in THF, 137 µL, 0.137 mmol). The crude mixture was purified by normal phase flash column chromatography (Biotage HC-10 g, pentane: EtOAc 0-10%) to afford the title compound (122 mg, 67%) as a colourless oil.

^{1}H NMR (600 MHz, CDCl3) δ 6.97 (s, 2H, *H*-Ar), 5.80 – 5.70 (m, 1H, *H*-1), 5.03 – 4.93 (m, 2H, CH_2 alkene), 4.34 – 4.22 (m, 2H, *H*-35), 3.79 – 3.66 (m, 3H, *H*-5, *H*-17 & *H*-19), 3.66 – 3.56 (m, 2H, *H*-9 & *H*-13), 2.91 – 2.80 (m, 3H, C*H*-*i*Pr), 2.13 – 2.05 (m, 1H, *H*-2'), 1.88 – 1.71 (m, 3H, *H*-2'' & *H*-34), 1.66 – 1.03 (m, 117H, CH_2-alkyls, CH_3-acetonide, CH_3-*i*Pr & CH_3-Bpin), 1.01 – 0.91 (m, 27H, CH_3-OTES), 0.87 (d, *J* = 6.7 Hz, 3H, CH_3-42), 0.64 – 0.53 (m, 18H, CH_2-OTES). See spectrum.

^{13}C NMR (151 MHz, CDCl$_3$) δ 171.06 (*C*O-benzoate), 149.99 (*C-para*), 144.85 (*C-ortho*), 137.32 (*C*H-1), 130.99 (*C-ipso*), 120.86 (*C-meta*), 115.95 (*C*H$_2$- alkene), 100.29 (CO anti-acetonide), 83.09 (*C*-Bpin), 82.88 (*C*-Bpin), 82.79 (*C*-Bpin), 82.78 (*C*-Bpin), 72.52 (*C*H-9 *or* *C*H-13), 72.47 (*C*H-9 *or* *C*H-13), 70.53 (*C*H-5), 66.82 (*C*H-17), 66.35 (*C*H-19), 64.85 (*C*H-35), 44.48 (*C*H-4), 41.55 (*C*H-2), 39.42 (*C*H-18), 37.79 (*C*H$_2$-6, 8, 10, 12, 14, 16 *or* 20), 37.78 (*C*H$_2$-6, 8, 10, 12, 14, 16 *or* 20), 37.68 (*C*H$_2$-6, 8, 10, 12, 14, 16 *or* 20), 37.65 (*C*H$_2$-6,

8, 10, 12, 14, 16 *or* 20), 37.63 (*C*H_2-6, 8, 10, 12, 14, 16 *or* 20), 37.28 (*C*H_2-6, 8, 10, 12, 14, 16 *or* 20), 36.30 (*C*H_2-6, 8, 10, 12, 14, 16 *or* 20), 34.55 (*C*H-*i*Pr *para*), 32.04 (*C*H_2-22, 24, 26, 28, 30 *or* 32), 31.89 (*C*H_2-22, 24, 26, 28, 30 *or* 32), 31.83 (*C*H_2-22, 24, 26, 28, 30 *or* 32), 31.81 (*C*H_2-22, 24, 26, 28, 30 *or* 32), 31.80 (*C*H_2-22, 24, 26, 28, 30 *or* 32), 31.61 (*C*H_2-22, 24, 26, 28, 30 *or* 32), 31.53 (*C*H-*i*Pr *ortho*), 30.10 (*C*H_2-34), 29.45 (*H*-3), 29.03 (*C*H_2-23 *or* 27 *or* 31), 28.80 (*C*H_2-23 *or* 27 *or* 31), 28.73 (*C*H_2-23 *or* 27 *or* 31), 25.13 (*C*H_3), 24.95 (*C*H_3), 24.94 (*C*H_3), 24.90 (*C*H_3), 24.86 (*C*H_3), 24.84 (*C*H_3-*anti*-acetonide), 24.82 (*C*H_3-*anti*-acetonide), 24.30 (*C*H_3-*i*Pr), 24.28 (*C*H_3-*i*Pr), 24.09 (*C*H_3-*i*Pr), 21.77 (*C*H_2-7 *or* *C*H_2-11, *or* *C*H_2-15), 21.39 (*C*H_2-7 *or* *C*H_2-11, *or* *C*H_2-15), 21.19 (*C*H_2-7 *or* *C*H_2-11, *or* *C*H_2-15), 20.06 (*C*H_3-42), 7.10 (OTES-*C*H_3), 7.09 (OTES-*C*H_3), 5.32 (OTES-*C*H_2), 5.28 (OTES-*C*H_2), 5.24 (OTES-*C*H_2). See spectrum.

HRMS (m/z): (nanospray) calculated for $C_{98}H_{186}B_4O_{15}Si_3$ [M+Na]+ 1754.3369, found 1754.3435.

TLC: $\boldsymbol{R_f}$ = 0.48 (80:20 hexane/EtOAc, stained with *p*-anisaldehyde).

IR (ν_{max}/cm^{-1}, neat): 2956, 2928, 2875, 1726 (C=O), 1607, 1460, 1378, 1314, 1239, 1144, 1076, 1006, 725.

$[\boldsymbol{\alpha}]_{\mathbf{D}}^{\mathbf{25}}$: −2 (*c* = 1, $CHCl_3$).

(3R,7R,11S,15R)-16-((4S,6S)-2,2-dimethyl-6-((4R,8R,12R,14S)-14-methyl-4,8,12-tris((triethylsilyl)oxy)heptadec-16-en-1-yl)-1,3-dioxan-4-yl)-3,7,11,15-tetrakis((triethylsilyl)oxy)hexadecyl 2,4,6-triisopropylbenzoate ((3*S*)-CF_1-3)

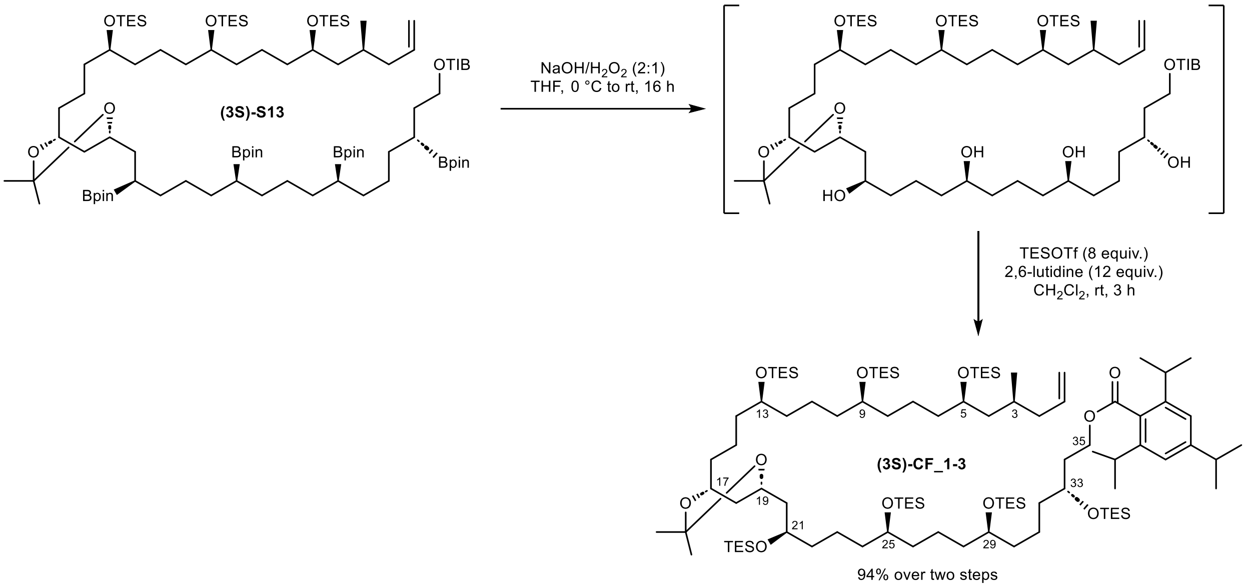

Prepared following General Procedure 4 using tetraboronic ester **(3S)-S13** (120 mg, 69 µmol), triethylsilyl triflate (220 mg, 188 µL, 0.831 mmol), 2,6-lutidine (118 mg, 127 µL, 1.11 mmol). The crude mixture was purified by normal phase flash column chromatography (Biotage HC-10 g, pentane: EtOAc 0-10%) to afford the title compound (114 mg, 94% over 2 steps) as a colourless oil.

^{1}H NMR (500 MHz, $CDCl_3$) δ 7.00 (s, 2H, *H*-Ar), 5.82 – 5.70 (m, 1H, *H*-1), 5.04 – 4.95 (m, 2H, C*H*2 alkene), 4.43 (ddd, *J* = 10.9, 7.4, 5.7 Hz, 1H, *H*-35'), 4.34 (dt, *J* = 10.9, 7.2 Hz, 1H, *H*-35''), 3.96 – 3.87 (m, 1H, *H*-21), 3.87 – 3.81 (m, 1H, *H*-33), 3.80 – 3.69 (m, 3H, *H*-5, *H*-17, *H*-19), 3.69 – 3.56 (m, 4H, *H*-9, *H*-13, *H*-25, *H*-29), 2.94 – 2.79 (m, 3H, C*H*-*i*Pr), 2.15 – 2.06 (m, 1H, *H*-2'), 1.93 – 1.79 (m, 3H, *H*-2'' & *H*-34), 1.73 (ddd, *J* = 13.5, 7.7, 5.6 Hz, 1H, *H*-20'), 1.69 – 1.33 (m, 39H, C*H*2), 1.32 (s, 3H, C*H*3-acetonide), 1.31 (s, 3H, C*H*3-acetonide), 1.28 (dd, *J* = 7.1, 3.3 Hz, 4H, C*H*2), 1.25 – 1.19 (m, 18H, C*H*3-*i*Pr), 0.96 (tdd, *J* = 7.7, 4.5, 2.8 Hz, 63H, C*H*3-OTES), 0.88 (d, *J* = 6.7 Hz, 3H, C*H*3-42), 0.67 – 0.52 (m, 42H, C*H*2-OTES). See spectrum.

^{13}C NMR (126 MHz, $CDCl_3$) δ 171.02 (*C*O-benzoate), 150.16 (*C*-*para*), 144.88 (*C*-*ortho*), 137.32 (*C*H-1), 130.76 (*C*-*ipso*), 120.93 (*C*-*meta*), 115.96 (*C*H2- alkene), 100.17 (CO anti-acetonide), 72.53 (*C*H-9, *C*H-13, *C*H-25 *or* *C*H-29), 72.51 (*C*H-9, *C*H-13, *C*H-25 *or* *C*H-29), 72.48 (*C*H-9, *C*H-13, *C*H-25 *or* *C*H-29), 72.45 (*C*H-9, *C*H-13, *C*H-25 *or* *C*H-29), 70.54 (*C*H-5, *C*H-17 *or* *C*H-19), 69.55 (*C*H-33), 69.18 (*C*H-5, *C*H-17 *or* *C*H-19), 66.69 (*C*H-5, *C*H-17

or *C*H-19), 63.76 (*C*H-21), 62.30 (*C*H-35), 44.49 (*C*H-4), 43.80 (*C*H-20), 41.57 (*C*H-2), 39.35 (*C*H-18), 38.09 (α-*C*H_2), 37.80 (α-*C*H_2), 37.78 (α-*C*H_2), 37.76 (α-*C*H_2), 37.69 (α-*C*H_2), 37.67 (α-*C*H_2), 37.65 (α-*C*H_2), 37.62 (α-*C*H_2), 37.60 (α-*C*H_2), 37.27 (α-*C*H_2), 36.31 (α-*C*H_2), 36.11 (*C*H-34), 34.55 (*C*H-*i*Pr *para*), 31.64 (*C*H-*i*Pr *ortho*), 29.85, 29.47 (*H*-3), 24.85 (*C*H_3-*anti*-acetonide), 24.80 (*C*H_3-*anti*-acetonide), 24.35 (*C*H_3-*i*Pr), 24.22 (*C*H_3-*i*Pr), 24.08 (*C*H_3-*i*Pr), 21.73 (β-*C*H_2), 21.47 (β-*C*H_2), 21.41 (β-*C*H_2), 21.30 (β-*C*H_2), 21.20 (β-*C*H_2), 21.12 (β-*C*H_2), 20.06 (*C*H_3-42), 7.11 (OTES-*C*H_3), 7.09 (OTES-*C*H_3), 7.08 (OTES-*C*H_3), 7.03 (OTES-*C*H_3), 5.33 (OTES-*C*H_2), 5.29 (OTES-*C*H_2), 5.27 (OTES-*C*H_2), 5.26 (OTES-*C*H_2), 5.20 (OTES-*C*H_2). *See spectrum*.

HRMS (m/z): (nanospray) calculated for $C_{98}H_{198}O_{11}Si_7$ [M+Na]+ 1771.3236, found 1771.3231.

TLC: $\boldsymbol{R_f}$ = 0.36 (95:5 hexane/EtOAc, stained with *p*-anisaldehyde).

IR (ν_{max}/cm^{-1}, neat): 2953, 2936, 2912, 2875, 1728 (C=O), 1607, 1459, 1377, 1238, 1074, 1006 (Si-O, 724 (Si-C).

$[\boldsymbol{\alpha}]_{\mathbf{D}}^{\mathbf{25}}$: −6 (*c* = 1, $CHCl_3$).

(6S,8R,12R,16S,20R)-21-((4S,6S)-2,2-dimethyl-6-((4R,8R,12R,14S)-14-methyl-4,8,12-tris((triethylsilyl)oxy)heptadec-16-en-1-yl)-1,3-dioxan-4-yl)-5-methyl-8,12,16,20-tetrakis((triethylsilyl)oxy)henicosan-6-ol ((3*S*, 36*S*/*R*)-CF_1-4)

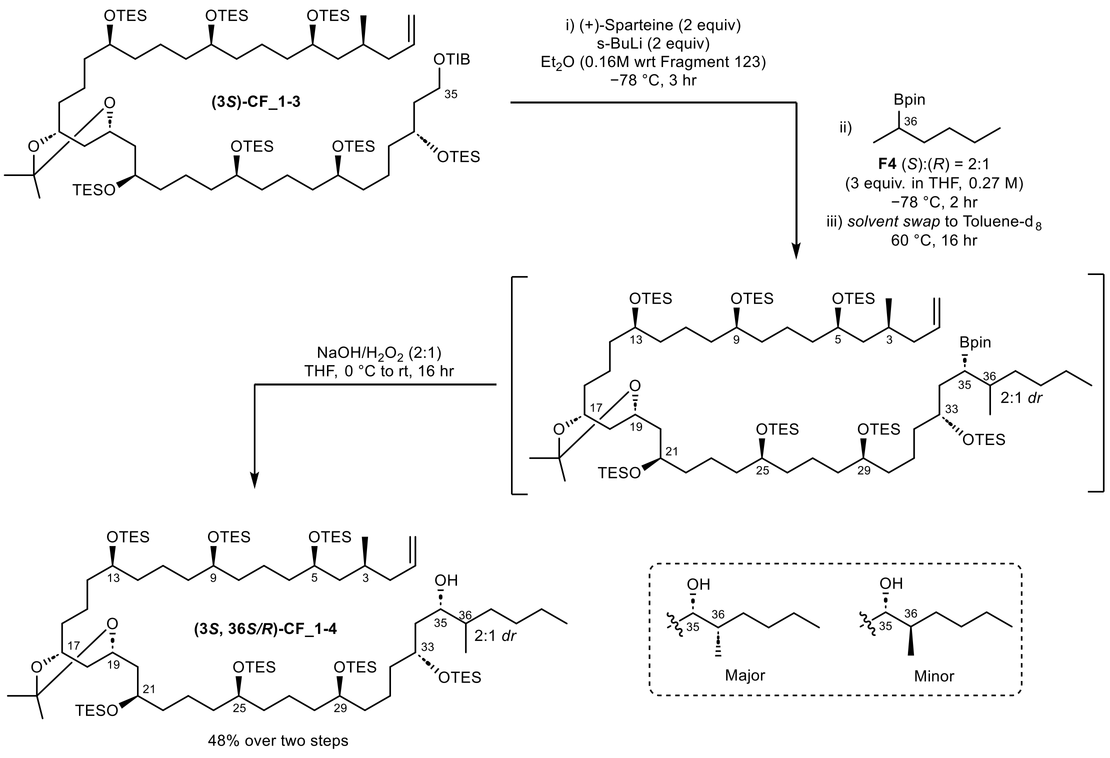

To a solution of TIB ester **(3*S*)-CF_1-3** (140 mg, 80 µmol, 1.00 equiv.) and (+)-sparteine (38 mg, 37 µL, 0.16 mmol, 2.0 equiv.) in diethyl ether (500 µL, 0.16 M), *s*-BuLi (1.31 M in hexanes) (122 µL, 0.16 mmol, 2.0 equiv.) was added dropwise at −78 °C and the reaction mixture was stirred at same temperature for 3 h. A solution of **F4** (2:1 mix of (*S*):(*R*) enantiomers), in THF (0.27 M, 890 µL) (51 mg, 0.240 mmol) was added dropwise (1 mL/min) to the reaction mixture at –78 °C, the reaction mixture was stirred for 2 h at −78 °C after which time the reaction mixture was warmed to room temperature, the solvent removed under vacuum and swapped with Toluene-*d8*. The reaction mixture was heated to 60 °C for 16 h. A 2:1 solution of aqueous sodium hydroxide (3 M) and hydrogen peroxide (30% aqueous solution) was added directly to the reaction mixture at 0 °C, and the reaction mixture then stirred at rt for 16 h. The reaction mixture was quenched by the addition of saturated sodium thiosulfate and was extracted with ethyl acetate (5X). The organic layers were combined, and washed with brine, then dried over anhydrous Na_2SO_4 and concentrated under reduced pressure. The crude residue was oxidised according to general procedure 4 and then purified by preparative HPLC (hexane: ethyl acetate 0-10%) to afford the title compound (61 mg, 48% over two steps) as a colourless oil.

^{1}H NMR (600 MHz, $CDCl_3$) *Spectrum not assigned due to 2:1 mixture of diastereomers of being formed.* See spectrum.

^{13}C NMR (101 MHz, $CDCl_3$) *Spectrum not assigned due to 2:1 mixture of diastereomers of being formed.* See spectrum.

HRMS (m/z): (nanospray) calculated for $C_{88}H_{188}O_{10}Si_7$ [M+Na]+ 1624.2485, found 1624.2477.

TLC: ***R*f** = 0.67 (85:15 hexane/EtOAc, stained with ceric ammonium molybdate (CAM))

IR (ν_{max}/cm^{-1}, neat): 3456 (OH), 2952, 2938, 2923, 2876, 1456, 1372, 1230, 1054, 1033.

4.6 Caylobolide A (3*R*, 36*S*/*R*) endgame

(3R,5R,9R,13R)-16-((4S,6S)-6-((2R,6S,10R,14R,16S)-16-hydroxy-17-methyl-2,6,10,14-tetrakis((triethylsilyl)oxy)henicosyl)-2,2-dimethyl-1,3-dioxan-4-yl)-3-methyl-5,9,13-tris((triethylsilyl)oxy)hexadecanoic acid ((3*R*, 36*S*/*R*)-18)

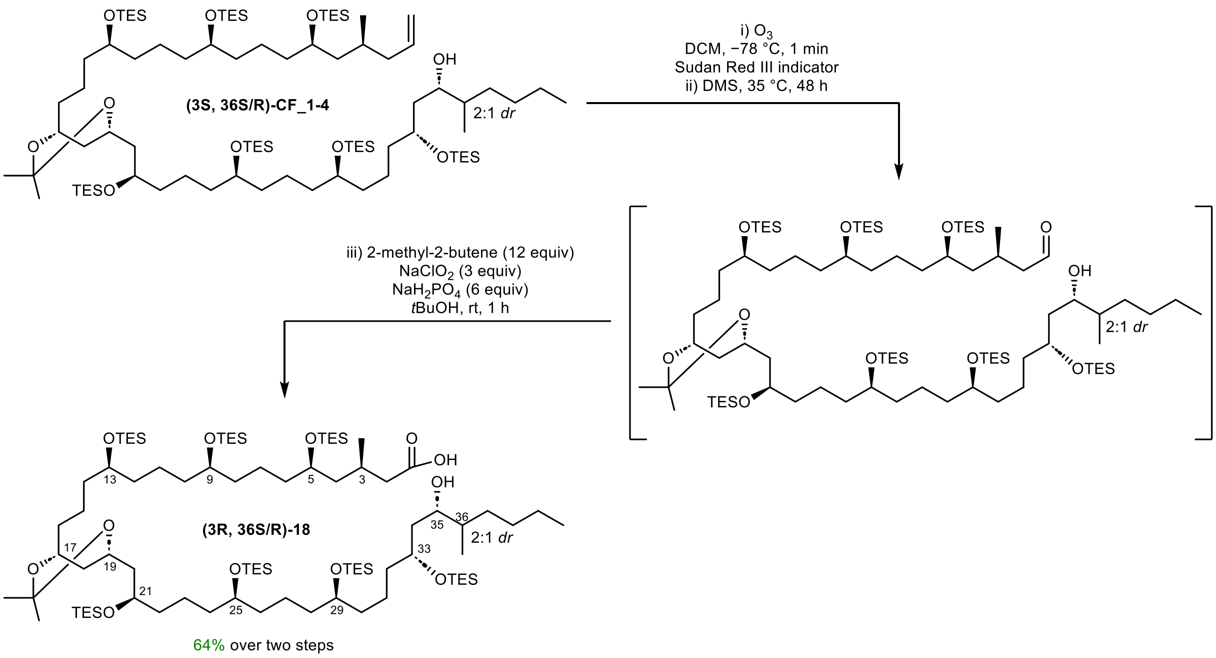

Ozone from a generator was bubbled through a solution of alkene **(3*S*, 36*S*/*R*)-CF_1-4** (60 mg, 37 µmol, 1.0 equiv.) in CH_2Cl_2 (190 µL, 0.20 M) and a small amount of Sudan III red stain (< 1 mg) at −78 °C for approximately 2 h until the solution turned from red to pale yellow. Oxygen was then bubbled through the solution at −78 °C for 5 minutes after which time, dimethylsulfide (190 uL, 1:1 with solvent) was added and the reaction mixture stirred at 35 °C for 48 h (until the ozonide was completely consumed). The reaction mixture was

evaporated under a stream of nitrogen, and evaporated under hi-vac to give crude aldehyde. A solution of oxidant was made by combining $NaClO_2$ (3.4 mg, 37 µmol, 3.0 equiv.), NaH_2PO_4 (9.0 mg, 75 µmol, 6.0 equiv.), and water (130 µL). Separately, the crude aldehyde prepared above (20 mg, 13 µmol) was dissolved in *t*-BuOH (250 µL), treated with 2-methyl-2-butene (11 mg, 150 µmol, 12 equiv.), and cooled to 0 °C. The stock solution of oxidant was added dropwise, and the resulting reaction mixture was stirred at rt until TLC showed consumption of starting material (1 h). Upon completion, the reaction was diluted with brine (1 mL) and extracted with ethyl acetate (3 x 2 mL). The combined organics were dried over Na_2SO_4 and evaporated *in vacuo*. The crude mixture was purified by normal phase flash column chromatography (Biotage HC-10 g, pentane: EtOAc 0-15%) to afford the title compound (13 mg, 64%) as a colourless oil.

^{1}H NMR (600 MHz, $CDCl_3$) *Spectrum not assigned due to 2:1 mixture of diastereomers of being formed* **See spectrum.**

^{13}C NMR (101 MHz, $CDCl_3$) *Spectrum not assigned due to 2:1 mixture of diastereomers of being formed.* **See spectrum.**

HRMS (m/z): (nanospray) calculated for $C_{87}H_{186}O_{12}Si_7$ [M+Na]+ 1642.2227, found 1642.2235.

TLC: ***R*f** aldehyde = 0.36, ***R*f** product (COOH) = 0.23 (92:8 hexane/EtOAc, stained with *p*-anisaldehyde).

IR (ν_{max}/cm^{-1}, neat): 3382 (OH), 2953, 2919, 2875, 2854, 1711 (C=O), 1459, 1378, 1261, 1075, 1009, 724.

(1S,5R,9R,13R,15R,19S,21R,25R,29S,33R,35S)-19-(hexan-2-yl)-15,37,37-trimethyl-5,9,13,21,25,29,33-heptakis((triethylsilyl)oxy)-18,36,38-trioxabicyclo[33.3.1]nonatriacontan-17-one ((3*R*, 36*S*/*R*)-19)

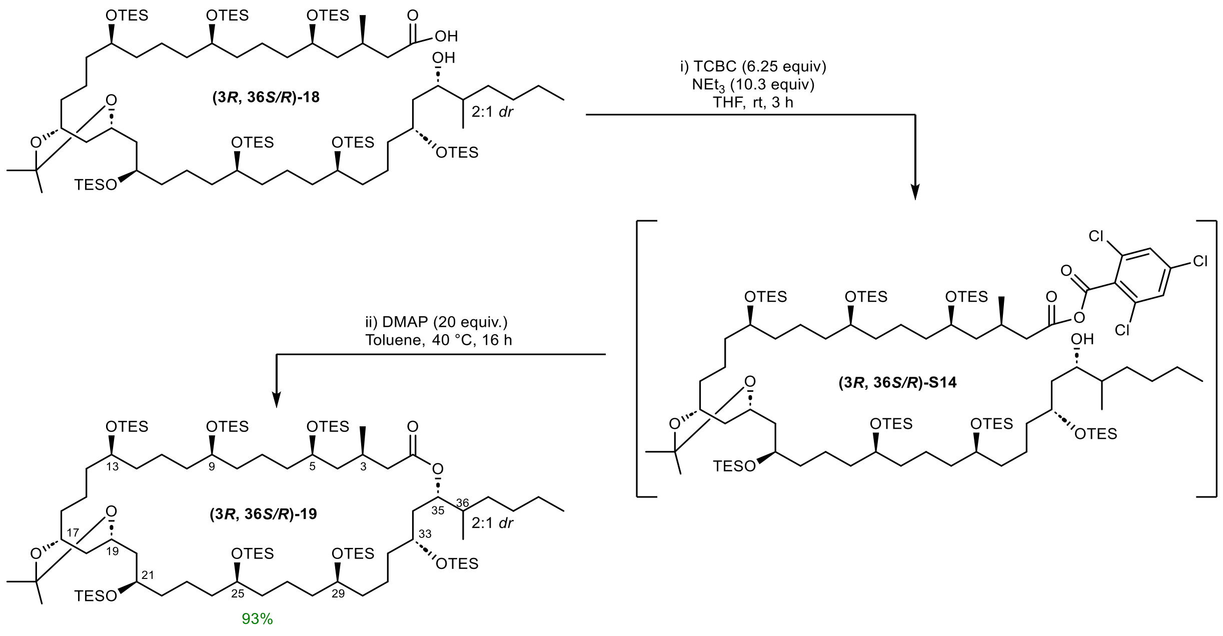

Formation of mixed anhydride (3*R*, 36*S*/*R*)-S14

According to a modified literature procedure, NEt_3 (0.04 M in THF, 0.95 mL, 38 µmol, 10 equiv.) then 2,4,6-trichlorobenzoyl chloride (0.04 M, 0.58 mL, 23 µmol, 6.3 equiv.) (TCBC) were added to acid **(3*R*, 36*S*/*R*)-18** (6.0 mg, 3.7 µmol) under an atmosphere of nitrogen. The resulting mixture was stirred at room temperature for 3 h after which time. The reaction mixture was concentrated and used in the next step crude.

Macrolactonisation

A solution of mixed anhydride **(3*R*, 36*S*/*R*)-S14** in anhydrous toluene (0.002 M, 1.85 mL) was added via syringe pump (2 mL/h) to a stirred solution of DMAP (9.0 mg, 74 µmol, 20 equiv.) in anhydrous toluene (0.01 M, 7.4 mL) at rt under an atmosphere of nitrogen. After addition was complete, the syringe was washed with anhydrous toluene (0.5 mL) and was added to the reaction. The resulting mixture was stirred at 40 °C for 16 h, after which time the reaction mixture was filtered through a plug of silica (to elute TCBC and TCBA). The silica plug was then washed with ethyl acetate and the filtrate collected, and the solvent evaporated. The crude mixture was purified by normal phase flash column chromatography (Biotage HC-10 g, pentane: EtOAc 0-10%) to afford the title compound (5.5 mg, 93%) as a colourless film.

^{1}H NMR (600 MHz, CDCl$_3$) *Spectrum not assigned due to 2:1 mixture of diastereomers of being formed.* See spectrum.

^{13}C NMR (101 MHz, CDCl$_3$) *Spectrum not assigned due to 2:1 mixture of diastereomers of being formed.* See spectrum.

HRMS (m/z): (nanospray) calculated for $C_{87}H_{184}O_{11}Si_7$ [M+Na]+ 1624.2121 found 1642.2115.

TLC: $\boldsymbol{R_f}$ = 0.36 (95:5 hexane/EtOAc, stained with ceric ammonium molybdate (CAM)).

IR (ν_{max}/cm^{-1}, neat): 2952, 2923, 2854, 1739 (C=O), 1458, 1376, 1217, 1066, 729.

(4R,6R,10R,14S,18R,22R,26S,30R,34R,36S)-36-(hexan-2-yl)-6,10,14,18,20,22,26,30,34-nonahydroxy-4-methyloxacyclohexatriacontan-2-one ((3*R*, 36*S*/*R*)-CA)

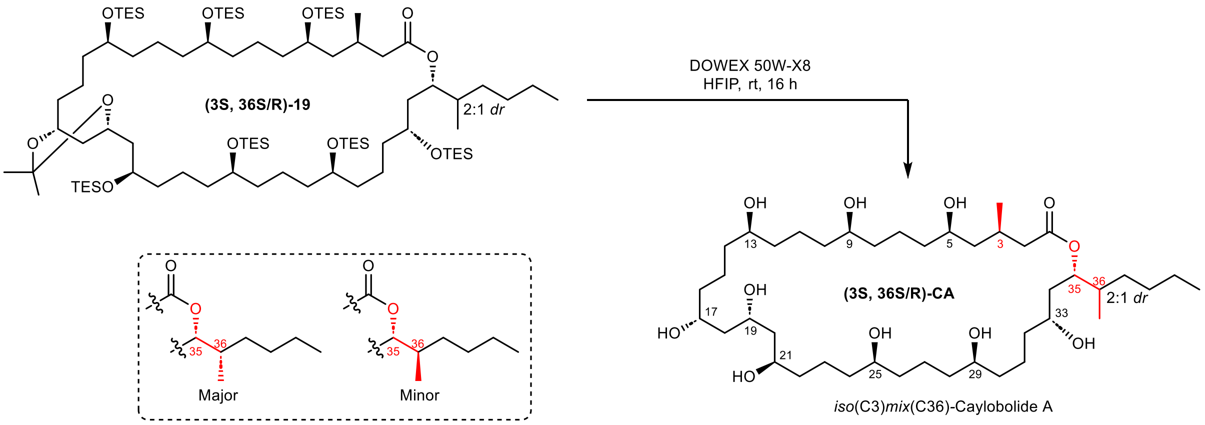

According to a modified literature procedure[12], acidic resin DOWEX-50W X8 (16 mg), previously washed with hexafluoroisopropanol, was added to a solution of protected Caylobolide analogue **(3*R*, 36*S*/*R*)-19** (4.0 mg, 2.5 µmol, 1.0 equiv.) in hexafluoroisopropanol (0.003 M, 0.83 mL). The reaction mixture was stirred at 16 h at rt then was filtered through a PTFE syringe filter, which was washed with hexafluoroisopropanol (1 mL). The combined organics were concentrated under a stream of nitrogen. The resulting residue was washed with HPLC pentane (3 x 1 mL), and then dried under high vacuum to provide a 2:1 mixture of stereoisomers (at C36) of caylobolide A (1.8 mg, 96%) as a white powder.

^{1}H NMR (600 MHz, CDCl$_3$) *Spectrum not assigned due to 2:1 mixture of diastereomers of being formed.* See spectrum.

^{13}C NMR (151 MHz, $CDCl_3$) *Spectrum not assigned due to 2:1 mixture of diastereomers of being formed.* [See spectrum](#).

HRMS (m/z): (ESI) calculated for $C_{42}H_{82}O_{11}$ [M+H]+ 763.5930, found 763.5917.

IR (ν_{max}/cm^{-1}, neat): 3333 (OH), 2954, 2919, 2861, 1735 (C=O), 1571, 1455, 1251, 1121, 1066.

^{13}C NMR comparison (natural sample vs. **(3*R*, 36*S*/*R*)-CA**)

*C*HOH region

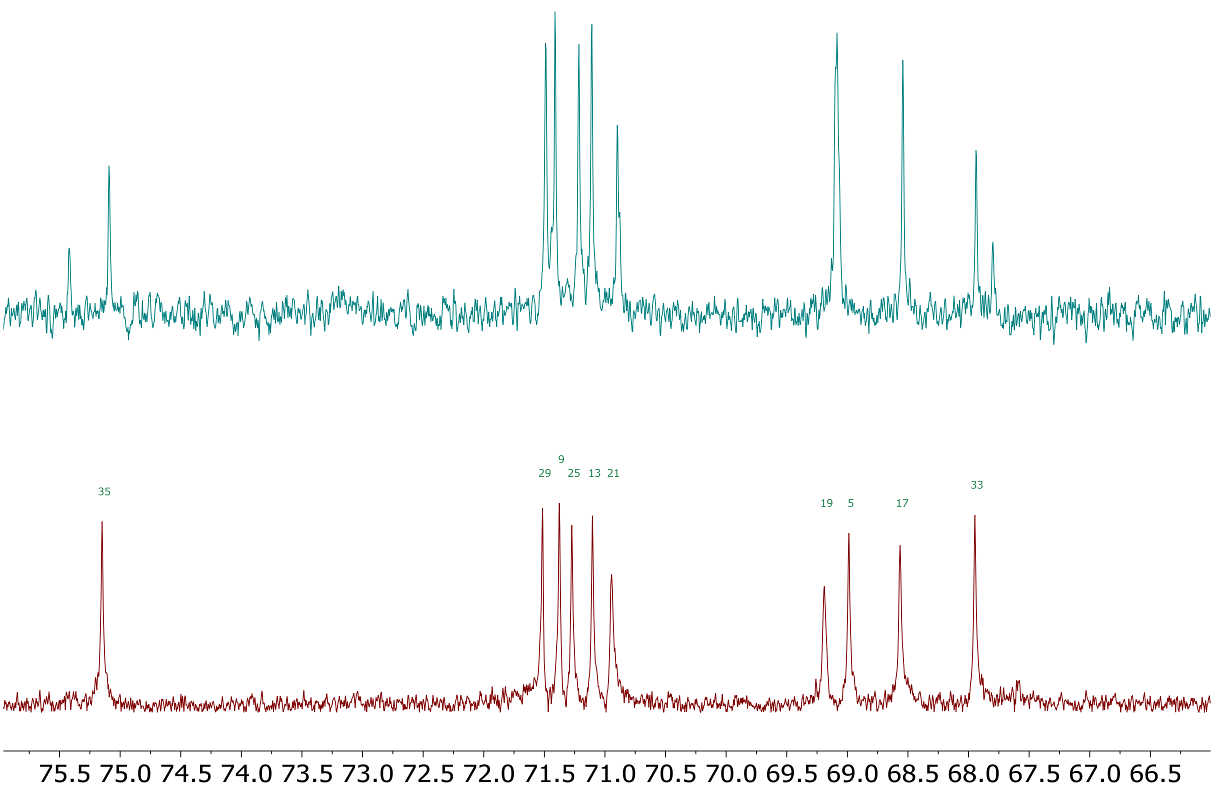

$\mathit{C}H_3$-42

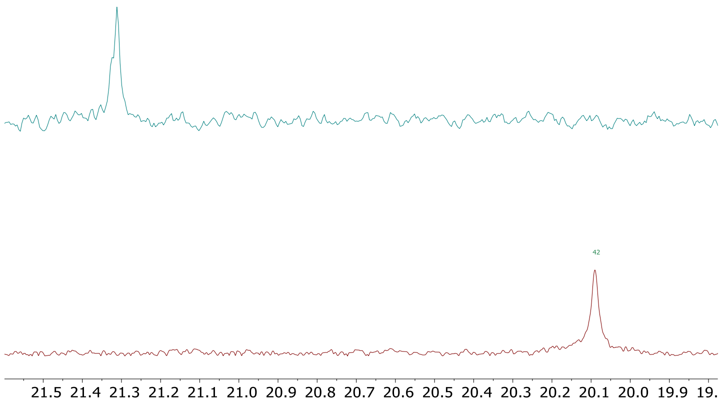

$\mathit{C}H_2$-2

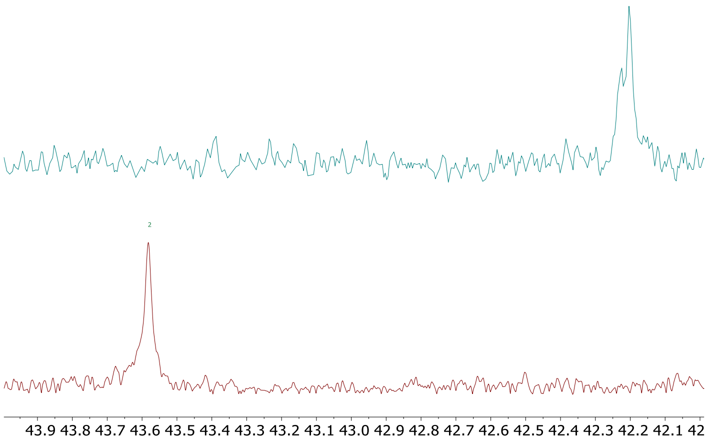

4.7 Caylobolide A (3*S*, 36*S*/*R*) fragment couplings

2-((6S)-6-((4R,8R,12R,14R)-4-hydroxy-14-methyl-8,12-bis((triethylsilyl)oxy)heptadec-16-en-1-yl)-2,2-dimethyl-1,3-dioxan-4-yl)ethyl 2,4,6-triisopropylbenzoate ((3*R*)-S12)

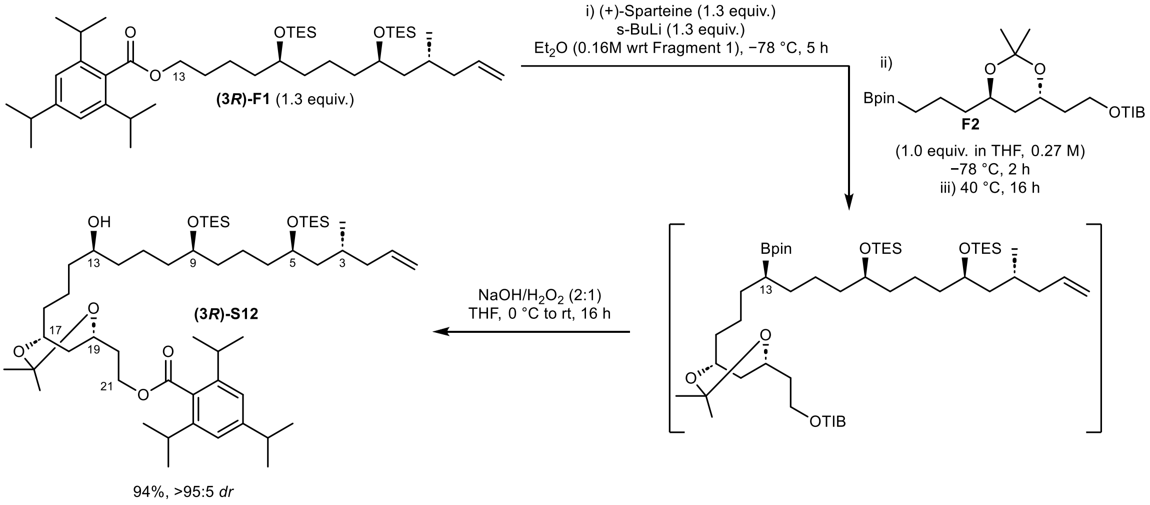

Prepared and purified following the same procedure as described for diastereomer **(3*S*)-S12** using **(3*R*)-F1** (284 mg, 0.396 mmol, 1.30 equiv.) and (+)-sparteine (84 mg, 82 µL, 0.40 mmol, 1.3 equiv.) in diethyl ether (1.9 mL, 0.16 M), *s*-BuLi (1.42 M in hexanes) (279 µL, 0.396 mmol, 1.30

equiv.) and **F2** in THF (0.27 M, 1.13 mL) (170 mg, 0.304 mmol) to afford the title compound (263 mg, 94%) as a colourless oil.

1H NMR (600 MHz, CDCl3) δ 7.00 (s, 2H, *H*-Ar), 5.77 (ddt, *J* = 17.2, 10.3, 7.1 Hz, 1H, *H*-1), 5.04 – 4.94 (m, 2H, C*H*2 alkene), 4.46 – 4.32 (m, 2H, *H*-21), 4.00 – 3.90 (m, 1H, *H*-19), 3.84 – 3.76 (m, 1H, *H*-17), 3.76 – 3.70 (m, 1H, *H*-5), 3.64 (p, *J* = 5.6 Hz, 1H, *H*-9), 3.59 (p, *J* = 4.9 Hz, 1H, *H*-13), 2.86 2.93 – 2.79 (m, 3H, C*H*-*i*Pr), 2.09 – 2.00 (m, 1H, *H*-2’), 1.95 – 1.82 (m, 3H, *H*-2’’ & *H*-21), 1.72 – 1.36 (m, 22H, C*H*2-alkyls), 1.35 (s, 3H, C*H*3-acetonide), 1.33 (s, 3H, C*H*3-acetonide), 1.24 (d, *J* = 6.9 Hz, 18H, C*H*3-*i*Pr), 1.17 (ddd, *J* = 13.5, 8.8, 4.4 Hz, 1H, *H*-4’), 0.96 (t, *J* = 7.9 Hz, 18H, C*H*3-OTES), 0.87 (d, *J* = 6.6 Hz, 3H, C*H*3-42), 0.59 (q, *J* = 7.9 Hz, 12H, C*H*2-OTES). See spectrum.

13C NMR (151 MHz, CDCl3) δ 171.06 (*C*O-benzoate), 150.28 (*C*-*para*), 144.85 (*C*-*ortho*), 137.56 (*C*H-1), 130.65 (*C*-*ipso*), 120.99 (*C*-*meta*), 115.82 (*C*H2- alkene), 100.54 (CO anti-acetonide), 72.39 (*C*H-9), 71.87 (*C*H-13), 70.43 (*C*H-5), 66.76 (*C*H-17), 63.64 (*C*H-19), 61.85 (*C*H-21), 44.29 (*C*H-4), 42.14 *C*H-2), 38.84 (*C*H-18), 38.48 (*C*H2-6 *or* CH2-8, *or* CH2-

10 *or* CH_2-12 *or* CH_2-14 *or* CH_2-16), 37.91 (CH_2-6 *or* CH_2-8, *or* CH_2-10 *or* CH_2-12 *or* CH_2-14 *or* CH_2-16), 37.61 (CH_2-6 *or* CH_2-8, *or* CH_2-10 *or* CH_2-12 *or* CH_2-14 *or* CH_2-16), 37.39 (CH_2-6 *or* CH_2-8, *or* CH_2-10 *or* CH_2-12 *or* CH_2-14 *or* CH_2-16), 37.27 (CH_2-6 *or* CH_2-8, *or* CH_2-10 *or* CH_2-12 *or* CH_2-14 *or* CH_2-16), 35.93 (CH_2-6 *or* CH_2-8, *or* CH_2-10 *or* CH_2-12 *or* CH_2-14 *or* CH_2-16), 35.03 (*H*-20), 34.57 (*C*H-*i*Pr *para*), 31.66 (*C*H-*i*Pr *ortho*), 29.20 (*C*H-3), 24.84 (CH_3-*anti*-acetonide), 24.79 (CH_3-*anti*-acetonide), 24.35 (CH_3-*i*Pr), 24.24 (CH_3-*i*Pr), 24.09 (CH_3-*i*Pr), 21.83 (CH_2-7 *or* CH_2-11, *or* CH_2-15), 21.56 (CH_2-7 *or* CH_2-11, *or* CH_2-15), 21.21 (CH_2-7 *or* CH_2-11, *or* CH_2-15), 19.73 (CH_3-42), 7.13 (OTES-CH_3), 7.12 (OTES-CH_3), 5.36 (OTES-CH_2), 5.27 (OTES-CH_2). See spectrum.

HRMS (m/z): (ESI) calculated for $C_{54}H_{100}O_7Si_2$ [M+H]+ 917.7080, found 917.7091.

TLC: $\boldsymbol{R_f}$ Bpin = 0.19 (95:5 hexane/EtOAc, stained with *p*-anisaldehyde), $\boldsymbol{R_f}$ product (OH) = 0.17 (90:10 hexane/EtOAc, stained with *p*-anisaldehyde).

IR (ν_{max}/cm^{-1}, neat): 3450 (OH), 2956, 2934, 2875, 1728, 1607, 1460, 1379, 1240, 1136, 1076, 1008, 725.

$[\alpha]_D^{25}$: −4 (*c* = 1, $CHCl_3$).

2-((6S)-2,2-dimethyl-6-((4R,8R,12R,14R)-14-methyl-4,8,12-tris((triethylsilyl)oxy)heptadec-16-en-1-yl)-1,3-dioxan-4-yl)ethyl 2,4,6-triisopropylbenzoate ((3*R*)-CF_1-2)

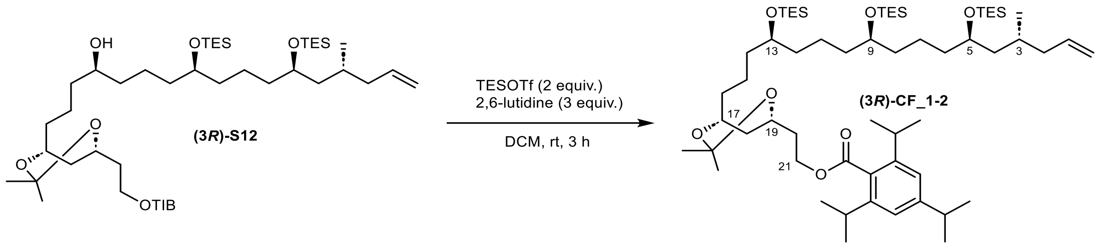

Prepared following General Procedure 5 using alcohol **(3*R*)-S12** (250 mg, 0.272 mmol), TESOTf (144 mg, 0.545 mmol, 123 µL), 2,6-lutidine (87 mg, 0.817 mmol, 94 µL). The crude mixture was purified by normal phase flash column chromatography (Biotage HC-10 g, pentane: EtOAc 0-10%) to afford the title compound (270 mg, 96%) as a colourless oil.

^{1}H NMR (500 MHz, CDCl3) δ 7.00 (s, 2H, *H*-Ar), 5.78 (dt, *J* = 16.9, 7.2 Hz, 1H, *H*-1), 5.05 – 4.95 (m, 2H, CH_2 alkene), 4 4.47 – 4.31 (m, 2H, *H*-21), 4.01 – 3.90 (m, 1H, *H*-19), 3.83 – 3.76 (m, 1H, *H*-17), 3.76 – 3.69 (m, 1H, *H*-5), 3.68 – 3.56 (m, 2H, *H*-9 & *H*-13), 2.96 – 2.77

(m, 3H, C*H*-*i*Pr), 2.11 – 1.99 (m, 1H, *H*-2'), 1.96 – 1.80 (m, 3H, *H*-2'' & *H*-21), 1.76 – 1.36 (m, 20H, CH_2-alkyls), 1.34 (s, 3H, CH_3-acetonide), 1.33 (s, 3H, CH_3-acetonide), 1.32 – 1.27 (m, 2H, CH_2-alkyls), 1.24 (d, J = 6.7 Hz, 18H, CH_3-*i*Pr), 1.17 (ddd, J = 13.6, 8.8, 4.3 Hz, 1H, *H*-4'), 1.03 – 0.91 (m, 27H, CH_3-OTES), 0.91 – 0.81 (m, 3H, CH_3-42), 0.59 (q, J = 7.8 Hz, 18H, CH_2-OTES). See spectrum.

^{13}C NMR (126 MHz, CDCl3) δ 171.06 (*C*O-benzoate), 150.28 (*C*-*para*), 144.86 (*C*-*ortho*), 137.57 (*C*H-1), 130.67 (*C*-*ipso*), 120.99 (*C*-*meta*), 115.81 (*C*H_2- alkene), 100.49 (CO anti-acetonide), 72.46 (*C*H-9 *or C*H-13), 72.45 (*C*H-9 *or C*H-13), 70.44 (*C*H-5), 66.71 (*C*H-17), 63.63 (*C*H-19), 61.88 (*C*H-21), 44.29 (*C*H-4), 42.16 (*C*H-2), 38.88 (*C*H-18), 38.52 (*C*H_2-6 *or C*H_2-8, *or C*H_2-10 *or C*H_2-12 *or C*H_2-14 *or C*H_2-16), 37.78 (*C*H_2-6 *or C*H_2-8, *or C*H_2-10 *or C*H_2-12 *or C*H_2-14 *or C*H_2-16), 37.67 (*C*H_2-6 *or C*H_2-8, *or C*H_2-10 *or C*H_2-12 *or C*H_2-14 *or C*H_2-16), 37.64 (*C*H_2-6 *or C*H_2-8, *or C*H_2-10 *or C*H_2-12 *or C*H_2-14 *or C*H_2-16), 37.22 (*C*H_2-6 *or C*H_2-8, *or C*H_2-10 *or C*H_2-12 *or C*H_2-14 *or C*H_2-16), 36.24 (*C*H_2-6 *or C*H_2-8, *or C*H_2-10 *or C*H_2-12 *or C*H_2-14 *or C*H_2-16), 35.05 (*C*H_2-20), 34.57 (*C*H-*i*Pr *para*), 31.66 (*C*H-*i*Pr *ortho*), 29.20 (*H*-3), 24.83 (*C*H_3-*anti*-acetonide), 24.71 (*C*H_3-*anti*-acetonide), 24.35 (*C*H_3-*i*Pr), 24.24 (*C*H_3-*i*Pr), 24.09 (*C*H_3-*i*Pr), 21.64 (*C*H_2-7 *or C*H_2-11, *or C*H_2-15), 21.41 (*C*H_2-7 *or C*H_2-11, *or C*H_2-15), 21.24 (*C*H_2-7 *or C*H_2-11, *or C*H_2-15), 19.71 (*C*H_3-42), 7.13 (OTES-*C*H_3), 7.11 (OTES-*C*H_3), 5.36 (OTES-*C*H_2), 5.28 (OTES-*C*H_2), 5.26 (OTES-*C*H_2). *See spectrum*.

HRMS (m/z): (ESI) calculated for $C_{60}H_{114}O_7Si_3$ [M+H]+ 1031.7945, found 1031.7955.

TLC: $\boldsymbol{R_f}$ = 0.85 (80:20 hexane/EtOAc), $\boldsymbol{R_f}$ = 0.57 (90:10 hexane/EtOAc, stained with *p*-anisaldehyde)

IR (ν_{max}/cm^{-1}, neat): 2955, 2937, 2914, 2875, 1729 (C=O), 1607, 1460, 1376, 1227, 1136, 1055, 1033, 740.

$[\alpha]_D^{25}$: −4 (c = 1, $CHCl_3$).

(1R)-2-((6S)-2,2-dimethyl-6-((4R,8R,12R,14R)-14-methyl-4,8,12-tris((triethylsilyl)oxy)heptadec-16-en-1-yl)-1,3-dioxan-4-yl)-1-(p-tolylsulfinyl)ethyl 2,4,6-triisopropylbenzoate ((3*R*)-17)

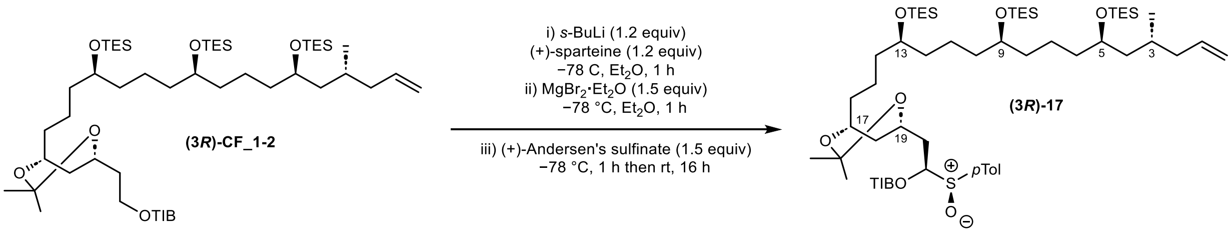

Prepared following General Procedure 1 using **(3*R*)-CF_1-2** (350 mg, 0.339 mmol), (+)-sparteine (95 mg, 94 µL, 0.407 mmol), *s*-BuLi (1.30 M in hexanes) (313 µL, 0.407 mmol), (+)-Andersen's sulfinate (150 mg, 0.509 mmol), magnesium turnings (66 mg, 2.71 mmol), dibromoethane (191 mg, 88 µL, 1.01 mmol), Et_3N (69 mg, 95 µL, 0.678 mmol), trimethylsilyl chloride (55 mg, 65 µL, 0.508 mmol). The crude residue was purified by normal phase preparative HPLC (hexane: EtOAc 0-20%) to afford the title compound (278 mg, 70%) as a colourless oil.

^{1}H NMR (600 MHz, CDCl3) δ 7.70 – 7.63 (m, 2H, H-Ar), 7.36 (d, *J* = 8.0 Hz, 2H, H-Ar), 7.05 (s, 2H, H-Ar), 5.83 – 5.72 (m, 2H, *H*-21 & *H*-1), 5.02 – 4.96 (m, 2H, (m, 2H, *H*-alkene), 3.76 – 3.69 (m, 1H, *H*-5), 3.67 – 3.56 (m, 3H, *H*-9 & *H*-13 & *H*-17), 3.53 – 3.46 (m, 1H, *H*-19), 3.01 – 2.86 (m, 3H, C*H*-iPr), 2.42 (s, 3H, CH_3-tolyl), 2.12 (dt, *J* = 15.1, 5.5 Hz, 1H, *H*-20'), 2.08 – 2.01 (m, 1H, *H*-2'), 1.98 (ddd, *J* = 14.9, 8.0, 6.6 Hz, 1H, *H*-20''), 1.90 (dt, *J* = 14.3, 7.4 Hz, 1H, *H*-2''), 1.75 – 1.63 (m, 1H, *H*-3), 1.49 – 1.31 (m, 19H, -CH_2), 1.31 – 1.25 (m, 23H, -CH_2 & CH_3-iPr), 1.22 (s, 3H, CH_3-acetonide), 1.17 (ddd, *J* = 13.4, 8.7, 4.3 Hz, 1H, *H*-4'), 1.00 (s, 3H, CH_3-acetonide), 0.99 – 0.91 (m, 27H, CH_3-OTES), 0.88 – 0.86 (m, 3H, *H*-42), 0.64 – 0.54 (m, 18H, CH_2-OTES). See spectrum.

^{13}C NMR (151 MHz, CDCl3) δ 170.25 (*C*O-benzoate), 151.06 (*C*H-TIB-*para*), 145.46 (*C*H-TIB-*ortho*), 141.71 (*C*-tolyl-*ipso*), 137.56 (*C*-tolyl-*para*), 137.54 (*C*H-1), 130.21 (*C*H-tolyl-*meta*), 128.82 (*C*H-TIB-*ipso*), 124.42 (*C*H-tolyl-*ortho*), 121.15 (TIB-*C*H-*meta*), 115.80 (*C*H_2-alkene), 100.44 (CO *anti*-acetonide), 90.11 (*C*H-21), 72.45 (*C*H-9 *or* *C*H-13), 72.41 (*C*H-9 *or* *C*H-13), 70.43 (*C*H-5), 66.44 (*C*H-17), 63.91 (*C*H-19), 44.28 (*C*H-4), 42.15 (*C*H-2), 38.59 (*C*H_2-6 *or* *C*H_2-8, *or* *C*H_2-10 *or* *C*H_2-12 *or* *C*H_2-14 *or* *C*H_2-16), 38.50 (*C*H_2-6 *or* *C*H_2-8, *or* *C*H_2-10 *or* *C*H_2-12 *or* *C*H_2-14 *or* *C*H_2-16), 37.78 (*C*H_2-6 *or* *C*H_2-8, *or* *C*H_2-10 *or* *C*H_2-12 *or* *C*H_2-14 *or* *C*H_2-16), 37.67 (*C*H_2-6 *or* *C*H_2-8, *or* *C*H_2-10 *or* *C*H_2-12 *or* *C*H_2-14 *or*

CH_2-16), 37.62 (*C*H_2-6 *or* *C*H_2-8, *or* *C*H_2-10 *or* *C*H_2-12 *or* *C*H_2-14 *or* *C*H_2-16), 37.19 (*C*H_2-6 *or* *C*H_2-8, *or* *C*H_2-10 *or* *C*H_2-12 *or* *C*H_2-14 *or* *C*H_2-16), 36.16 (*C*H_2-6 *or* *C*H_2-8, *or* *C*H_2-10 *or* *C*H_2-12 *or* *C*H_2-14 *or* *C*H_2-16), 34.60 (*C*H-*i*Pr *para*), 31.66 (*C*H-*i*Pr *ortho*), 30.27 (*C*H-2), 29.19 (*C*-3), 24.50 (*C*H_3-*i*Pr), 24.48 (*C*H_3-*i*Pr), 24.40 (*C*H_3-*i*Pr), 24.33 (*C*H_3-*i*Pr), 24.05 (*C*H_3--*i*Pr), 21.64 (*C*H_2-7 *or* *C*H_2-11, *or* *C*H_2-15), 21.55 (*C*H_3-tolyl), 21.39 (*C*H_2-7 *or* *C*H_2-11, *or* *C*H_2-15), 21.22 (*C*H_2-7 *or* *C*H_2-11, *or* *C*H_2-15), 19.70 (*C*H_3-42), 7.11 (*C*H_3-OTES), 7.09 (*C*H_3-OTES), 6.72 (*C*H_3-OTES), 5.94 (*C*H_2-OTES), 5.35 (*C*H_2-OTES), 5.28 (*C*H_2-OTES), 5.24 (*C*H_2-OTES). See spectrum.

HRMS (m/z): (nanospray) calculated for $C_{67}H_{120}O_8SSi_3$ [M+Na]+ 1191.7904, found 1191.7859.

TLC: *R*f = 0.51 (80:20 hexane/EtOAc, stained with *p*-anisaldehyde).

IR (ν_{max}/cm^{-1}, neat): 2956, 2935, 2912, 2875, 1736 (C=O), 1606, 1460, 1235, 1044 (S=O).

$[\alpha]_D^{25}$: −32 (*c* = 1, $CHCl_3$).

(3R,7R,11S,15R)-16-((4R,6S)-2,2-dimethyl-6-((4R,8R,12R,14R)-14-methyl-4,8,12-tris((triethylsilyl)oxy)heptadec-16-en-1-yl)-1,3-dioxan-4-yl)-3,7,11,15-tetrakis(4,4,5,5-tetramethyl-1,3,2-dioxaborolan-2-yl)hexadecyl 2,4,6-triisopropylbenzoate ((3*R*)-S13)

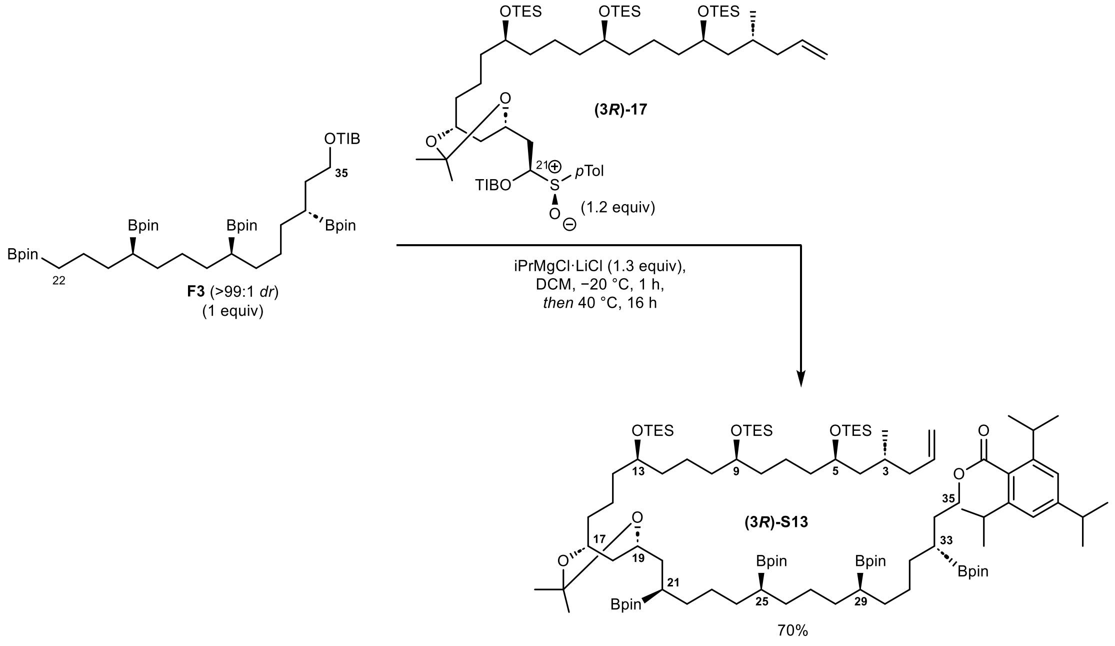

Prepared following General Procedure 2 using **F3** (165 mg, 0.174 mmol), sulfoxide **(3*R*)-17** (244 mg, 0.209 mmol), *i*PrMgCl·LiCl (1.3 M in THF, 174 µL, 0.226 mmol). The crude mixture was purified by normal phase flash column chromatography (Biotage HC-10 g, pentane: EtOAc 0-10%) to afford the title compound (210 mg, 70%) as a colourless oil.

^{1}H NMR (600 MHz, CDCl3) δ 6.98 (s, 2H, *H*-Ar), 5.81 – 5.71 (m, 1H, *H*-1), 5.03 – 4.94 (m, 2H, CH_2 alkene), 4.34 – 4.24 (m, 2H, *H*-35), 3.80 – 3.67 (m, 3H, *H*-5, *H*-17 & *H*-19), 3.66 – 3.56 (m, 2H, *H*-9 & *H*-13), 2.92 – 2.80 (m, 3H, C*H*-*i*Pr), 2.04 (ddd, *J* = 12.4, 6.8, 3.3 Hz, 1H, *H*-2'), 1.90 (dt, *J* = 14.3, 7.4 Hz, 1H, *H*-2''), 1.85 – 1.73 (m, 2H, *H*-34), 1.72 – 1.65 (m, 1H, *H*-3), 1.65 – 1.59 (m, 1H, -CH_2'), 1.58 – 1.50 (m, 2H, *H*-18), 1.50 – 1.03 (m, 122H, CH_2-alkyls, CH_3-acetonide, CH_3-*i*Pr & CH_3-Bpin), 0.95 0.99 – 0.92 (m, 27H, CH_3-OTES), 0.90 – 0.85 (m, 3H, CH_3-42), 0.62 – 0.55 (m, 18H, CH_2-OTES). See spectrum.

^{13}C NMR (151 MHz, CDCl3) δ 171.08 (*C*O-benzoate), 150.01 (*C*-*para*), 144.86 (*C*-*ortho*), 137.56 (*C*H-1), 131.01 (*C*-*ipso*), 120.88 (*C*-*meta*), 115.80 (*C*H_2- alkene), 100.31 (CO anti-acetonide), 83.11 (*C*-Bpin), 82.90 (*C*-Bpin), 82.81 (*C*-Bpin), 82.79 (*C*-Bpin), 72.53 (*C*H-9 *or* *C*H-13), 72.47 (*C*H-9 *or* *C*H-13), 70.44 (*C*H-5), 66.84 (*C*H-17), 66.37 (*C*H-19), 64.87 (*C*H-35), 44.29 (*C*H-4), 42.16 (*C*H-2), 39.43 (*C*H-18), 38.52 (*C*H_2-6, 8, 10, 12, 14, 16 *or* 20), 37.80 (*C*H_2-6, 8, 10, 12, 14, 16 *or* 20), 37.66 (*C*H_2-6, 8, 10, 12, 14, 16 *or* 20), 37.30 (*C*H_2-6, 8, 10, 12, 14, 16 *or* 20), 36.32 (*C*H_2-6, 8, 10, 12, 14, 16 *or* 20), 34.57 (*C*H-*i*Pr *para*), 32.06 (*C*H_2-22, 24, 26, 28, 30 *or* 32), 31.92 (*C*H_2-22, 24, 26, 28, 30 *or* 32), 31.85 (*C*H_2-22, 24, 26, 28, 30 *or* 32), 31.83 (*C*H_2-22, 24, 26, 28, 30 *or* 32), 31.82 (*C*H_2-22, 24, 26, 28, 30 *or* 32), 31.63 (*C*H_2-22, 24, 26, 28, 30 *or* 32), 31.54 (*C*H-*i*Pr *ortho*), 30.11 (*C*H_2-34), 29.19 (*H*-3), 29.05 (*C*H_2-23 *or* 27 *or* 31), 28.83 (*C*H_2-23 *or* 27 *or* 31), 28.76 (*C*H_2-23 *or* 27 *or* 31), 25.14 (*C*H_3), 24.97 (*C*H_3), 24.96 (*C*H_3), 24.95 (*C*H_3), 24.91 (*C*H_3), 24.88 (*C*H_3), 24.85 (*C*H_3-*anti*-acetonide), 24.83 (*C*H_3-*anti*-acetonide), 24.32 (*C*H_3-*i*Pr), 24.30 (*C*H_3-*i*Pr), 24.11 (*C*H_3-*i*Pr), 21.79 (*C*H_2-7 *or* *C*H_2-11, *or* *C*H_2-15), 21.42 (*C*H_2-7 *or* *C*H_2-11, *or* *C*H_2-15), 21.24 (*C*H_2-7 *or* *C*H_2-11, *or* *C*H_2-15), 19.70 (*C*H_3-42), 7.12 (OTES-*C*H_3), 5.36 (OTES-*C*H_2), 5.28 (OTES-*C*H_2), 5.26 (OTES-*C*H_2). See spectrum.

HRMS (m/z): (nanospray) calculated for $C_{98}H_{186}B_4O_{15}Si_3$ [M+Na]+ 1754.3369, found 1754.3436.

TLC: R_f = 0.48 (80:20 hexane/EtOAc, stained with *p*-anisaldehyde).

IR (ν_{max}/cm^{-1}, neat): 2956, 2931, 2876, 1727 (C=O), 1461, 1379, 1316, 1239, 1145, 1075, 1010, 742.

$[\alpha]_D^{25}$: −8 (c = 1, $CHCl_3$).

(3R,7R,11S,15R)-16-((4S,6S)-2,2-dimethyl-6-((4R,8R,12R,14R)-14-methyl-4,8,12-tris((triethylsilyl)oxy)heptadec-16-en-1-yl)-1,3-dioxan-4-yl)-3,7,11,15-tetrakis((triethylsilyl)oxy)hexadecyl 2,4,6-triisopropylbenzoate ((3*R*)-CF_1-3)

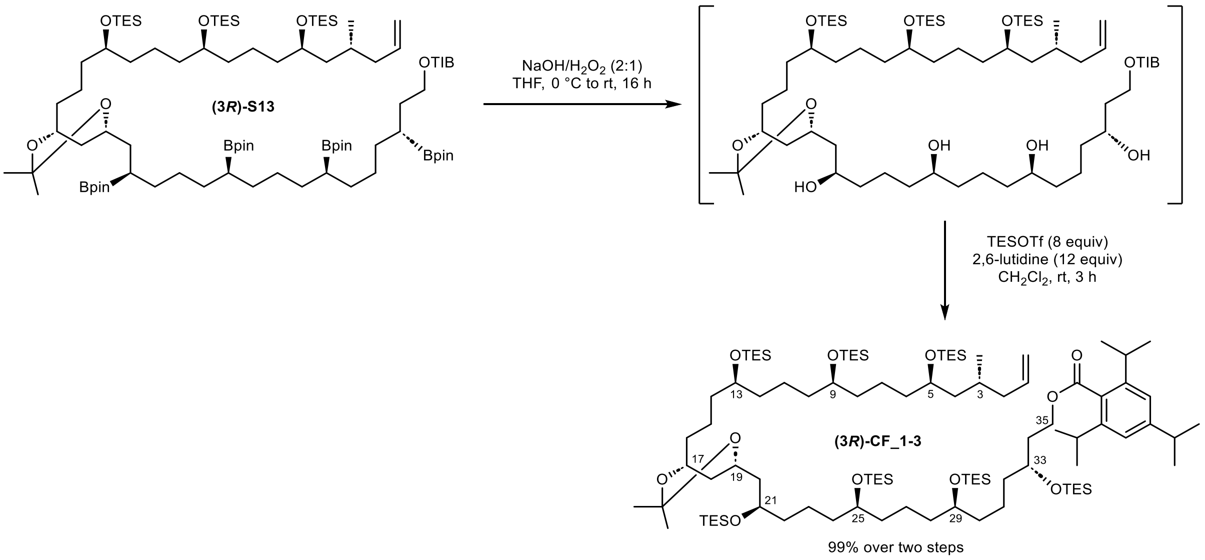

Prepared following the same procedure as described for diastereomer **(3*S*)-CF_1-2** using tetraboronic ester **(3*R*)-S13** (205 mg, 118 µmol), triethylsilyl triflate (375 mg, 321 µL, 1.42 mmol), 2,6-lutidine (201 mg, 217 µL, 1.89 mmol). The crude mixture was purified by normal phase flash column chromatography (Biotage HC-10 g, pentane: EtOAc 0-10%) to afford the title compound (205 mg, 99%) as a colourless oil.

^{1}H NMR (500 MHz, CDCl3) δ 7.00 (s, 2H, *H*-Ar), 5.84 – 5.71 (m, 1H, *H*-1), 5.03 – 4.93 (m, 2H, C*H*$_2$ alkene), 4.48 – 4.39 (m, 1H, *H*-35'), 4.34 (dt, *J* = 10.9, 7.2 Hz, 1H, *H*-35''), 3.95 – 3.86 (m, 1H, *H*-21), 3.86 – 3.80 (m, 1H, *H*-33), 3.80 – 3.69 (m, 3H, *H*-5, *H*-17, *H*-19), 3.67 – 3.58 (m, 4H, *H*-9, *H*-13, *H*-25, *H*-29), 2.94 – 2.79 (m, 3H, C*H*-*i*Pr), 2.09 – 2.01 (m, 1H, *H*-2'), 1.94 – 1.82 (m, 3H, *H*-2'' & *H*-34), 1.77 – 1.65 (m, 2H, *H*-3 & *H*-20'), 1.62 – 1.33 (m, 39H, C*H*$_2$), 1.32 (s, 3H, C*H*$_3$-acetonide), 1.31 (s, 3H, C*H*$_3$-acetonide), 1.30 – 1.25 (m, 4H, C*H*$_2$), 1.26 – 1.21 (m, 18H, C*H*$_3$-*i*Pr), 1.17 (ddd, *J* = 13.5, 8.8, 4.3 Hz, 1H, *H*-4'), 1.02 – 0.91 (m, 63H, C*H*$_3$-OTES), 0.87 (d, *J* = 6.6 Hz, 3H, C*H*$_3$-42), 0.65 – 0.54 (m, 42H, C*H*$_2$-OTES). See spectrum.

^{13}C NMR (126 MHz, CDCl3) δ 171.04 (*C*O-benzoate), 150.16 (*C-para*), 144.88 (*C-ortho*), 137.55 (*C*H-1), 130.76 (*C-ipso*), 120.93 (*C-meta*), 115.80 (*C*H_2- alkene), 100.18 (CO anti-acetonide), 72.53 (*C*H-9, *C*H-13, *C*H-25 *or C*H-29), 72.51 (*C*H-9, *C*H-13, *C*H-25 *or C*H-29), 72.47 (*C*H-9, *C*H-13, *C*H-25 *or C*H-29), 72.45 (*C*H-9, *C*H-13, *C*H-25 *or C*H-29), 70.44 (*C*H-5, *C*H-17 *or C*H-19), 69.55 (*C*H-33), 69.19 (*C*H-5, *C*H-17 *or C*H-19), 66.70 (*C*H-5, *C*H-17 *or C*H-19), 63.77 (*C*H-21), 62.31 (*C*H-35), 44.28 (*C*H-4), 43.80 (*C*H-20), 42.15 (*C*H-2), 39.35 (*C*H-18), 38.51 (α-*C*H_2), 38.09 (α-*C*H_2), 37.79 (α-*C*H_2), 37.76 (α-*C*H_2), 37.69 (α-*C*H_2), 37.66 (α-*C*H_2), 37.64 (α-*C*H_2), 37.62 (α-*C*H_2), 37.60 (α-*C*H_2), 37.27 (α-*C*H_2), 36.31 (α-*C*H_2), 36.11 (*C*H-34), 34.55 (*C*H-*i*Pr *para*), 31.64 (*C*H-*i*Pr *ortho*), 29.19 (*H*-3), 24.85 (*C*H_3-*anti*-acetonide), 24.80 (*C*H_3-*anti*-acetonide), 24.35 (*C*H_3-*i*Pr), 24.22 (*C*H_3-*i*Pr), 24.08 (*C*H_3-*i*Pr), 21.73 (β-*C*H_2), 21.48 (β-*C*H_2), 21.42 (β-*C*H_2), 21.30 (β-*C*H_2), 21.23 (β-*C*H_2), 21.12 (β-*C*H_2), 19.70 (*C*H_3-42), 7.11 (OTES-*C*H_3), 7.10 (OTES-*C*H_3), 7.09 (OTES-*C*H_3), 7.08 (OTES-*C*H_3), 7.03 (OTES-*C*H_3), 6.72 (OTES-*C*H_3), 5.94 (OTES-*C*H_2), 5.36 (OTES-*C*H_2), 5.28 (OTES-*C*H_2), 5.27 (OTES-*C*H_2), 5.26 (OTES-*C*H_2), 5.20 (OTES-*C*H_2). *See spectrum*.

HRMS (m/z): (MALDI) calculated for $C_{98}H_{198}O_{11}Si_7$ [M+Na]+ 1771.3236, found 1771.3229.

TLC: $\boldsymbol{R_f}$ = 0.36 (95:5 hexane/EtOAc, stained with *p*-anisaldehyde).

IR (ν_{max}/cm^{-1}, neat): 2952, 2921, 2874, 2856, 1729 (C=O), 1607, 1458, 1377, 1235, 1074, 1007 (Si-O), 724 (Si-C).

$[\alpha]_D^{25}$: −4 (*c* = 1, $CHCl_3$).

(6S,8R,12R,16S,20R)-21-((4S,6S)-2,2-dimethyl-6-((4R,8R,12R,14R)-14-methyl-4,8,12-tris((triethylsilyl)oxy)heptadec-16-en-1-yl)-1,3-dioxan-4-yl)-5-methyl-8,12,16,20-tetrakis((triethylsilyl)oxy)henicosan-6-ol ((3*R*, 36*S*/*R*)-CF_1-4)

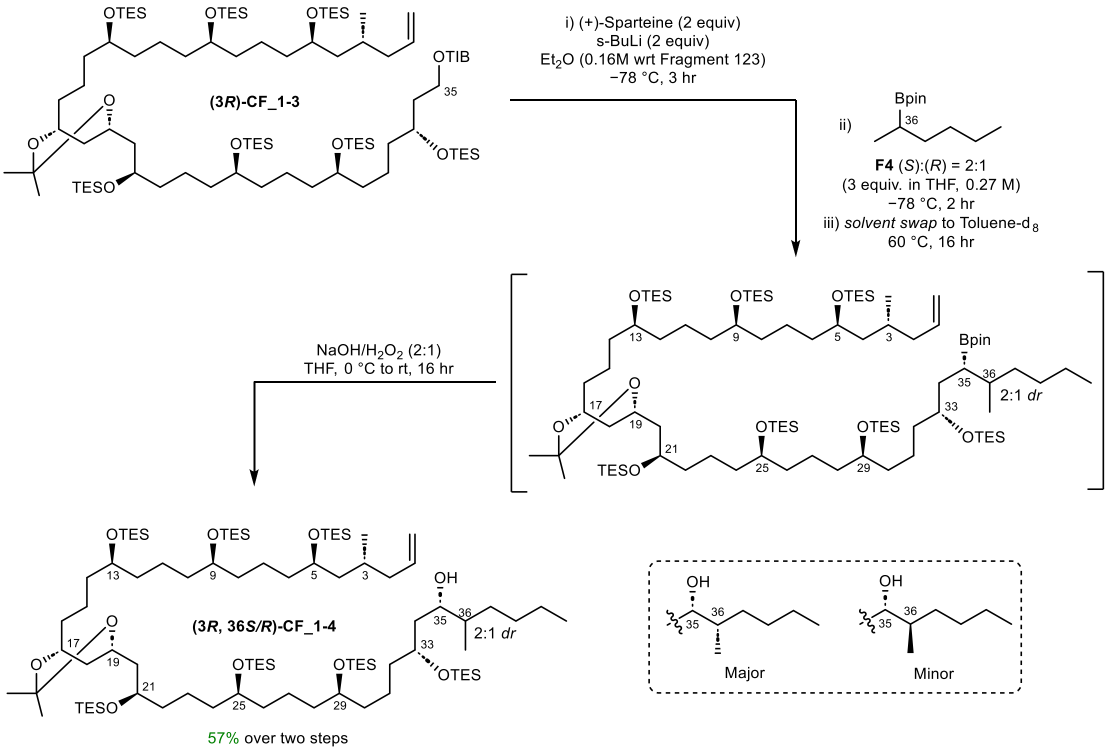

Prepared following the same procedure as described for diastereomer **(3*S*, 36*S*/*R*)-CF_1-4** using TIB ester **(3*R*)-CF_1-3** (60 mg, 34.3 μmol), (+)-sparteine (17 mg, 16 μL, 68.6 μmol) in diethyl ether (214 μL, 0.16 M), *s*-BuLi (1.31 M in hexanes) (52 μL, 68.6 μmol), **F4** (2:1 mix of (*S*):(*R*) enantiomers), in THF (0.27 M, 381 μL) (22 mg, 103 μmol). The crude residue was purified by preparative HPLC (hexane: ethyl acetate 5-15%) to afford the title compound (33.7 mg, 57% over two steps) as a colourless oil.

^{1}H NMR (600 MHz, $CDCl_3$) *Spectrum not assigned due to 2:1 mixture of diastereomers of being formed. See spectrum.*

^{13}C NMR (151 MHz, $CDCl_3$) *Spectrum not assigned due to 2:1 mixture of diastereomers of being formed. See spectrum.*

HRMS (m/z): (nanospray) calculated for $C_{88}H_{188}O_{10}Si_7$ [M+Na]+ 1624.2485, found 1624.2476.

TLC: R_f = 0.67 (85:15 hexane/EtOAc, stained with ceric ammonium molybdate (CAM))

IR (ν_{max}/cm^{-1}, neat): 3514 (OH), 2953, 2937, 2913, 2876, 1459, 1378, 1072, 1008, 726 (Si-O).

4.8 Caylobolide A (3*S*, 36*S*/*R*) endgame

(3S,5R,9R,13R)-16-((4S,6S)-6-((2R,6S,10R,14R,16S)-16-hydroxy-17-methyl-2,6,10,14-tetrakis((triethylsilyl)oxy)henicosyl)-2,2-dimethyl-1,3-dioxan-4-yl)-3-methyl-5,9,13-tris((triethylsilyl)oxy)hexadecanoic acid ((3*S*, 36*S*/*R*)-18)

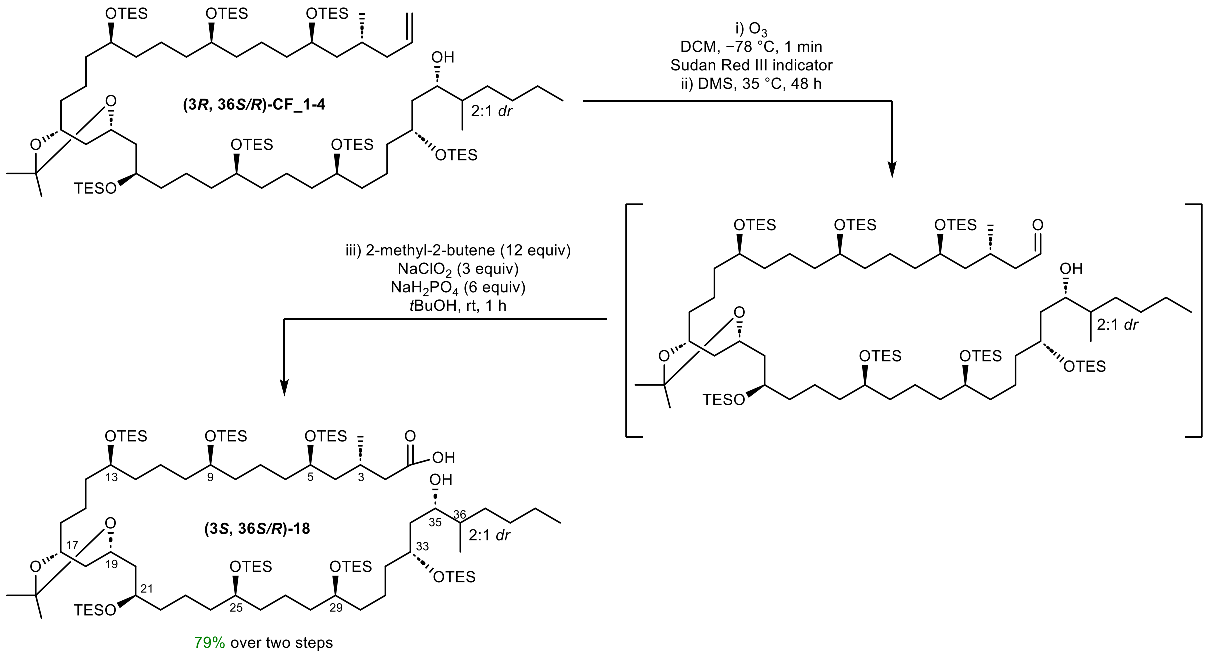

Prepared following the same procedure as described for diastereomer **((3*R*, 36*S*/*R*)-18)** using alkene **(3*R*, 36*S*/*R*)-CF_1-4** (30 mg, 18.7 µmol) in CH_2Cl_2 (94 µL, 0.2 M), dimethylsulfide (94 uL), $NaClO_2$ (5.1 mg, 56.1 µmol), NaH_2PO_4 (13.5 mg, 112 µmol), water (187 µL), *t*-BuOH (374 µL), 2-methyl-2-butene (15.7 mg, 225 µmol). The crude mixture was purified by normal phase flash column chromatography (Biotage HC-10 g, pentane: EtOAc 0-15%) to afford the title compound (24 mg, 79%) as a colourless oil.

^{1}H NMR (600 MHz, $CDCl_3$) *Spectrum not assigned due to 2:1 mixture of diastereomers of being formed. See spectrum.*

^{13}C NMR (151 MHz, $CDCl_3$) *Spectrum not assigned due to 2:1 mixture of diastereomers of being formed. See spectrum.*

HRMS (m/z): (nanospray) calculated for $C_{87}H_{186}O_{12}Si_7$ [M+Na]+ 1642.2227, found 1642.2219.

TLC: ***R*f** aldehyde = 0.36, ***R*f** product (COOH) = 0.23 (92:8 hexane/EtOAc, stained with *p*-anisaldehyde).

IR (ν_{max}/cm^{-1}, neat): 3452 (OH), 2952, 2925, 2875, 2856, 1738 (C=O), 1457, 1366, 1217, 1007 (Si-O), 724 (Si-C).

(1S,5R,9R,13R,15S,19S,21R,25R,29S,33R,35S)-19-(hexan-2-yl)-15,37,37-trimethyl-5,9,13,21,25,29,33-heptakis((triethylsilyl)oxy)-18,36,38-trioxabicyclo[33.3.1]nonatriacontan-17-one ((3*S*, 36*S*/*R*)-19)

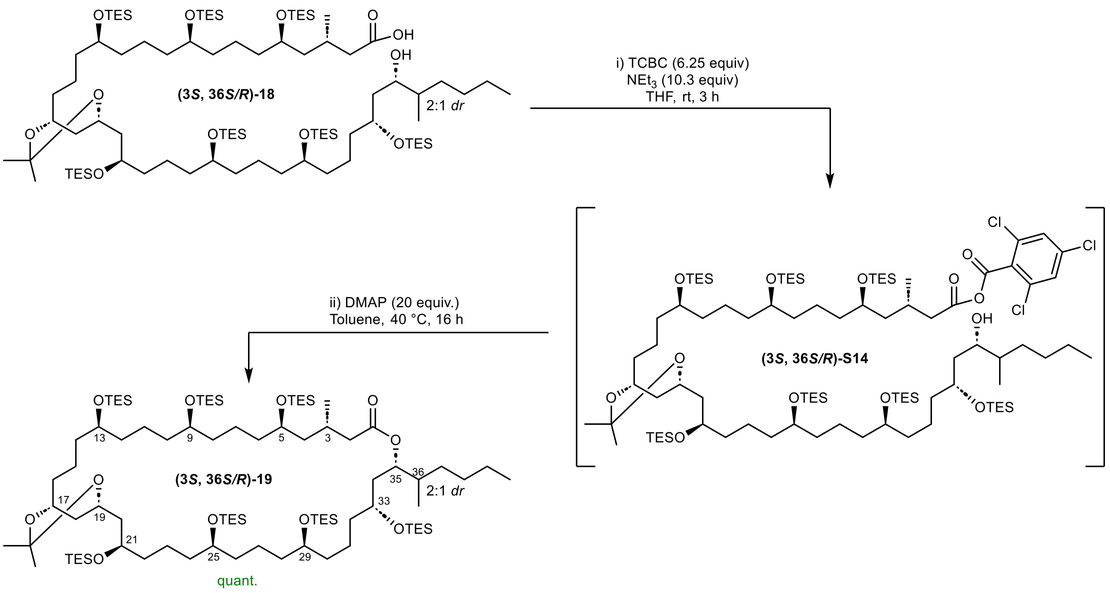

Formation of mixed anhydride (3*S*, 36*S*/*R*)-S14

Prepared following the same procedure as described for diastereomer **(3*R*, 36*S*/*R*)-S14** using NEt_3 (0.04 M in THF, 3.81 mL, 152 µmol), 2,4,6-trichlorobenzoyl chloride (0.04 M, 2.31 mL, 92.5 µmol) and acid **(3*S*, 36*S*/*R*)-18** (24 mg, 14.8 µmol). The reaction mixture was concentrated and used in the next step crude.

Macrolactonisation

Prepared following the same procedure as described for diastereomer **((3*R*, 36*S*/*R*)-19)** using mixed anhydride **(3*S*, 36*S*/*R*)-S14** in anhydrous toluene (0.002 M, 7.40 mL), DMAP (36.2 mg, 296 µmol) in anhydrous toluene (0.01 M, 29.6 mL). The crude mixture was purified by normal phase flash column chromatography (Biotage HC-10 g, pentane: EtOAc 0-10%) to afford the title compound (20 mg, 96%) as a colourless film.

^{1}H NMR (600 MHz, $CDCl_3$) *Spectrum not assigned due to 2:1 mixture of diastereomers of being formed.* See spectrum.

^{13}C NMR (151 MHz, $CDCl_3$) *Spectrum not assigned due to 2:1 mixture of diastereomers of being formed.* See spectrum.

HRMS (m/z): (nanospray) calculated for $C_{87}H_{184}O_{11}Si_7$ [M+Na]+ 1624.2121 found 1642.2124.

TLC: ***R*f** = 0.36 (95:5 hexane/EtOAc, stained with ceric ammonium molybdate (CAM)).

IR (ν_{max}/cm^{-1}, neat): 2952, 2937, 2923, 2875, 1733 (C=O), 1457, 1377, 1235, 1055, 1033.

(4S,6R,10R,14S,18R,22R,26S,30R,34R,36S)-36-(hexan-2-yl)-6,10,14,18,20,22,26,30,34-nonahydroxy-4-methyloxacyclohexatriacontan-2-one (3*S*, 36*S*/*R*)-CA

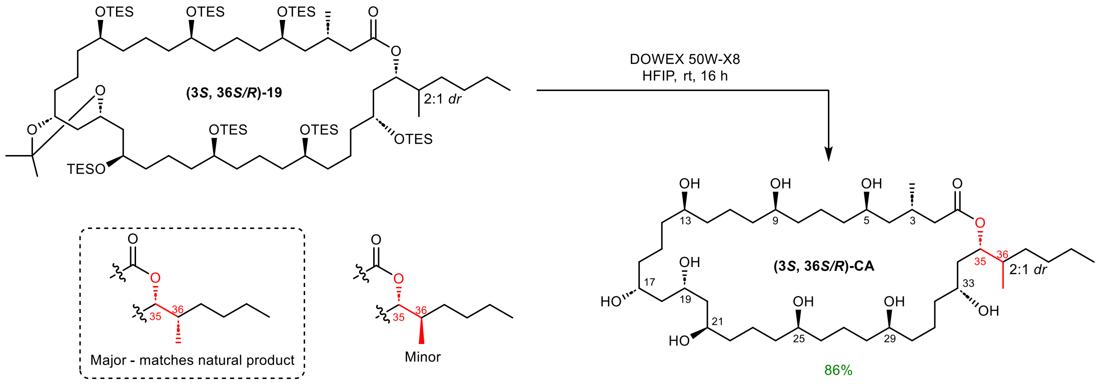

Prepared following the same procedure as described for diastereomer **(3*R*, 36*S*/*R*)-CA** using acidic resin DOWEX-50W X8 (48 mg), protected Caylobolide mix **(3*S*, 36*S*/*R*)-19** (12 mg, 7.49 µmol) in hexafluoroisopropanol (0.003 M, 2.5 mL) to give a 2:1 mixture of stereoisomers (at C36) of Caylobolide A (4.9 mg, 86%) as a white powder.

^{1}H NMR (500 MHz, Pyr) δ *Spectrum not assigned due to 2:1 mixture of diastereomers of being formed.* See spectrum.

^{13}C NMR (126 MHz, Pyr) δ *Spectrum not assigned due to 2:1 mixture of diastereomers of being formed.* See spectrum.

HRMS (m/z): (ESI) calculated for $C_{42}H_{82}O_{11}$ [M+H]+ 763.5930, found 763.5909.

IR (ν_{max}/cm^{-1}, neat): 3341 (OH), 2952, 2918, 2860, 1735 (C=O), 1593, 1454, 1348, 1120.

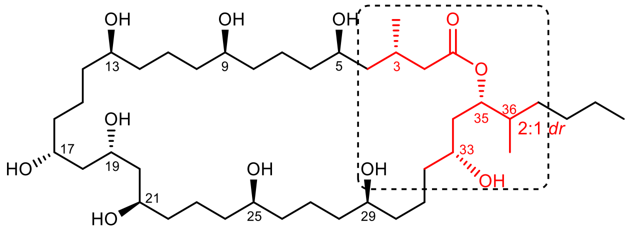

#C	natural product δC (ppm)	Mixture 1					
		Major: (3*R*, 36*S*)-CA			Minor: (3*R*, 36*R*)-CA		
**32**	39.15	39.14	0.01	0.01	39.14	0.01	0.01
**33-COH**	67.42	67.46	-0.04	0.04	67.33	0.09	0.09
**34**	40.05	40.04	0.01	0.01	38.29	1.76	1.76
**35-CO**	74.62	74.62	0	0	74.75	-0.13	0.13
**36**	37.39	37.39	0	0	36.78	0.61	0.61
**37**	32.55	32.58	-0.03	0.03	32.5	0.05	0.05
**41-Me**	14.89	14.85	0.04	0.04	14.74	0.15	0.15
**2**	43.05	41.69	1.36	1.36	41.69	1.36	1.36
**3**	27.59	27.79	-0.2	0.2	27.79	-0.2	0.2
**4**	45.04	45.13	-0.09	0.09	45.13	-0.09	0.09
**5**	68.46	68.64	-0.18	0.18	68.64	-0.18	0.18
**42-Me**	19.56	20.84	-1.28	1.28	20.84	-1.28	1.28
			**MAE**	0.27		**MAE**	0.4925

#C	natural product δC (ppm)	Mixture 2					
		Major: (3*S*, 36*S*)-CA			Minor: (3*S*, 36*R*)-CA		
**32**	39.15	39.14	0.01	0.01	39.21	-0.06	0.06
**33-COH**	67.42	67.41	0.01	0.01	67.27	0.15	0.15
**34**	40.05	40.07	-0.02	0.02	38.23	1.82	1.82
**35-CO**	74.62	74.61	0.01	0.01	74.95	-0.33	0.33
**36**	37.39	37.39	0	0	36.82	0.57	0.57
**37**	32.55	32.54	0.01	0.01	32.45	0.1	0.1
**41-Me**	14.89	14.89	0	0	14.78	0.11	0.11
**2**	43.05	43.05	0	0	43.18	-0.13	0.13
**3**	27.59	27.58	0.01	0.01	27.65	-0.06	0.06
**4**	45.04	45.05	-0.01	0.01	45.09	-0.05	0.05
**5**	68.46	68.47	-0.01	0.01	68.47	-0.01	0.01
**42-Me**	19.56	19.56	0	0	19.56	0	0
			**MAE**	0.0075		**MAE**	0.2825

Table S5: Data used for the comparison of diastereomeric mixtures 1 and 2 to naturally occurring caylobolide A for the determination of stereochemistry at C3 and C36. The major component of Mixture 2 (3S, 36S)-CA matches caylobolide A, with a mean absolute error of 0.0075 ppm for the atoms investigated, and a maximum difference of 0.02 ppm.

4.9 Stereocontrolled caylobolide A (3*S*, 36*S*) endgame

(5S,6S,8R,12R,16S,20R)-21-((4S,6S)-2,2-dimethyl-6-((4R,8R,12R,14R)-14-methyl-4,8,12-tris((triethylsilyl)oxy)heptadec-16-en-1-yl)-1,3-dioxan-4-yl)-5-methyl-8,12,16,20-tetrakis((triethylsilyl)oxy)henicosan-6-ol ((3*R*, 36*S*)-CF_1-4)

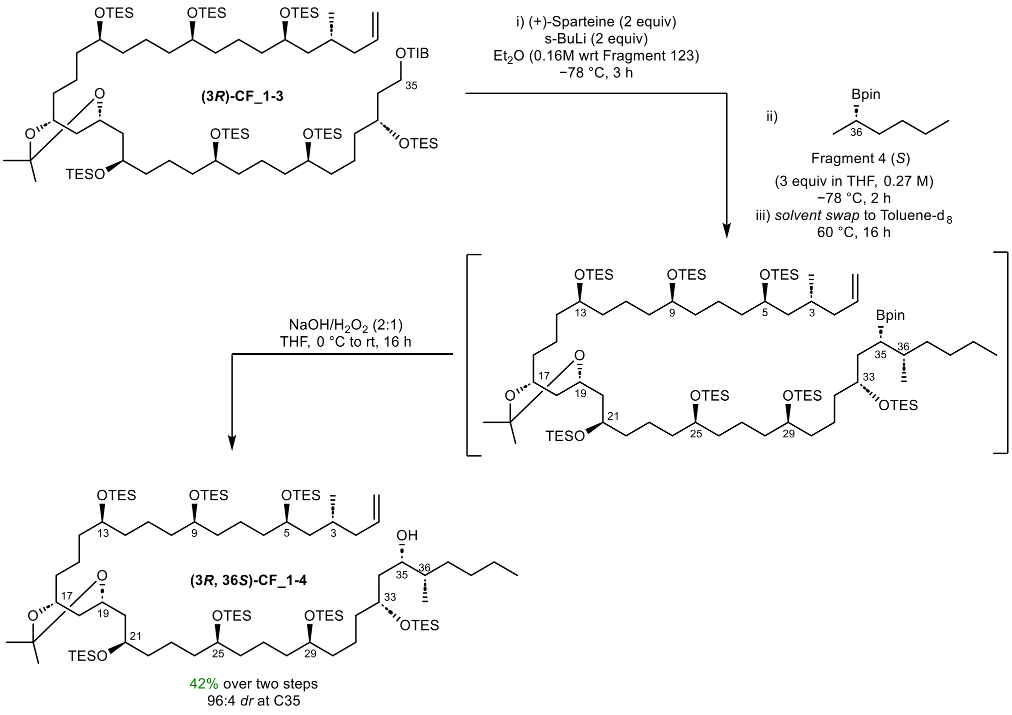

Prepared following the same procedure as described for diastereomer **(3*R*, 36*S*/*R*)-CF_1-4** using TIB ester **(3*R*)-CF_1-3** (60 mg, 34.3 µmol), (+)-sparteine (17 mg, 16 µL, 68.6 µmol) in diethyl ether (214 µL, 0.16 M), *s*-BuLi (1.31 M in hexanes) (52 µL, 68.6 µmol), (*S*)-Fragment 4 in THF (0.27 M, 381 µL) (22 mg, 103 µmol). The crude residue was purified by preparative HPLC (hexane: ethyl acetate 5-15%) to afford the title compound (23 mg, 42% over two steps) as a colourless oil.

^{1}H NMR (600 MHz, CDCl3) δ 5.84 – 5.74 (m, 1H, *H*-1), 5.05 – 4.98 (m, 2H C*H*$_2$ alkene), 4.02 (tt, *J* = 7.7, 4.0 Hz, 1H, *H*-33), 3.92 (dddd, *J* = 7.3, 7.3, 7.0 Hz, 1H, *H*-19), 3.89 – 3.83 (m, 1H, *H*-35), 3.82 – 3.78 (m, 1H, *H*-21), 3.78 – 3.76 (m, 1H, *H*-17), 3.76 – 3.72 (m, 1H, *H*-5), 3.68 – 3.62 (m, 4H, *H*-9, *H*-13, *H*-25 & *H*-29), 3.27 (d, *J* = 2.2 Hz, 1H, O*H*), 2.10 – 2.04 (m, 1H, *H*-2'), 1.96 – 1.89 (m, 1H, *H*-2''), 1.79 – 1.73 (m, 1H, *H*-20'), 1.73 – 1.68 (m, 1H, *H*-3), 1.69 – 1.35 (m, 47H, -C*H*$_2$), 1.34 (s, 3H, C*H*$_3$-acetonide), 1.33 (s, 3H, C*H*$_3$-acetonide), 1.33 – 1.22 (m, 10H, -C*H*$_2$), 1.22 – 1.17 (m, 1H, *H*-4'), 1.17 – 1.11 (m, 1H, *H*-36'), 1.03 – 0.94 (m, 63H, C*H*$_3$-OTES), 0.94 – 0.85 (m, 9H, C*H*$_3$-40, C*H*$_3$-41 & C*H*$_3$-42), 0.68 – 0.57 (m, 42H, C*H*$_2$-OTES). See spectrum.

^{13}C NMR (151 MHz, CDCl3) δ 137.57 (*C*H-1), 115.81 (*C*H$_2$- alkene), 100.19 (CO anti-acetonide), 72.53 (2X *C*H-9, *C*H-13, *C*H-25 *or* *C*H-29), 72.47 (*C*H-9, *C*H-13, *C*H-25 *or* *C*H-29), 72.39 (*C*H-9, *C*H-13, *C*H-25 *or* *C*H-29), 71.95 (*C*H-33), 71.64 (*C*H-35), 70.45 (*C*H-5), 69.19 (*C*H-21), 66.71 (*C*H-17), 63.77 (*C*H-19), 44.29 (*C*H-4), 43.81 (*C*H$_2$-20), 42.16 (*C*H-2), 39.35, 39.09 (α-*C*H$_2$), 38.53 (α-*C*H$_2$), 38.22 (α-*C*H$_2$), 37.81 (α-*C*H$_2$), 37.79 (α-*C*H$_2$), 37.70 (α-*C*H$_2$), 37.67 (α-*C*H$_2$), 37.58 (α-*C*H$_2$), 37.41 (α-*C*H$_2$), 37.28 (α-*C*H$_2$), 36.62 (α-*C*H$_2$), 36.32 (α-*C*H$_2$), 32.68 (*C*H$_2$-37), 29.88 (*C*H$_2$-38), 29.20 (*C*H-3), 24.86 (*C*H$_3$-*anti*-acetonide), 24.81 (*C*H$_3$-*anti*-acetonide), 23.19 (*C*H$_2$-39), 21.74 (2X β-*C*H$_2$), 21.50 (β-*C*H$_2$), 21.43 (β-*C*H$_2$), 21.30 (β-*C*H$_2$), 21.24 (β-*C*H$_2$), 19.71 (*C*H$_3$-42), 14.43 (*C*H$_3$-40 *or* *C*H$_3$-41), 14.28 (*C*H$_3$-40 *or* *C*H$_3$-41), 7.12 (OTES-*C*H$_3$), 7.09 (OTES-*C*H$_3$), 7.00 (OTES-*C*H$_3$), 5.37 (OTES-*C*H$_2$), 5.29 (OTES-*C*H$_2$), 5.26 (OTES-*C*H$_2$), 5.04 (OTES-*C*H$_2$). See spectrum.

HRMS (m/z): (nanospray) calculated for $C_{88}H_{188}O_{10}Si_7$ [M+Na]+ 1624.2485, found 1624.2482.

TLC: ***R*f** = 0.67 (85:15 hexane/EtOAc, stained with ceric ammonium molybdate (CAM))

IR (ν_{max}/cm^{-1}, neat): 3520 (OH), 2952, 2936, 2912, 2876, 1459, 1414, 1377, 1237, 1120, 1073, 1045, 1006 (Si-O), 724 (Si-C).

$[\alpha]_D^{25}$: −18 (c = 1, $CHCl_3$).

(3S,5R,9R,13R)-16-((4S,6S)-6-((2R,6S,10R,14R,16S,17S)-16-hydroxy-17-methyl-2,6,10,14-tetrakis((triethylsilyl)oxy)henicosyl)-2,2-dimethyl-1,3-dioxan-4-yl)-3-methyl-5,9,13-tris((triethylsilyl)oxy)hexadecanoic acid ((3*S*, 36*S*)-18)

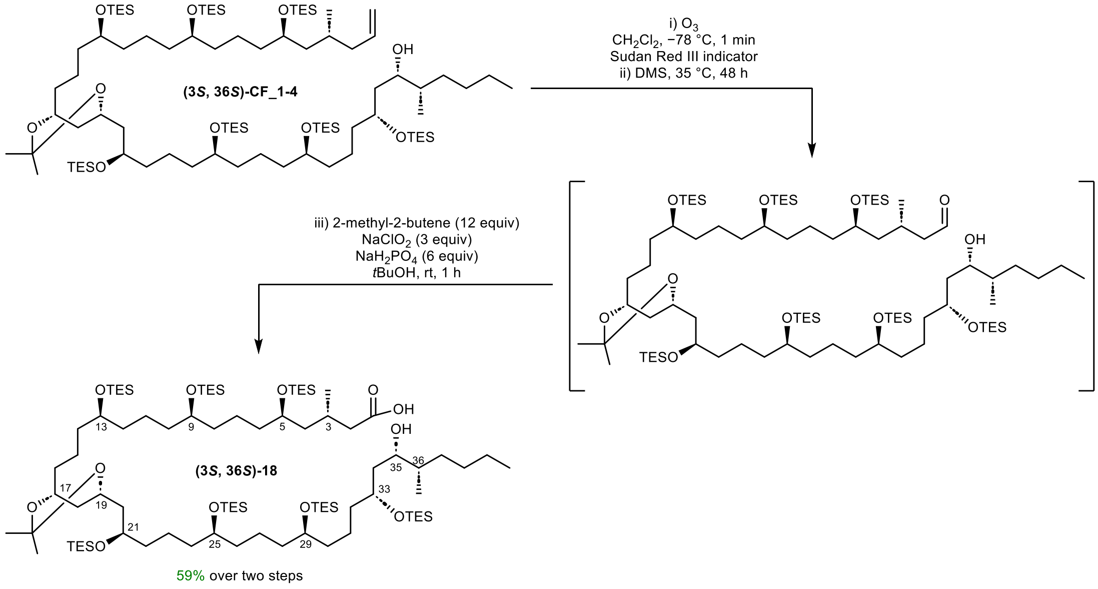

Prepared following the same procedure as described for diastereomer **(3*S*, 36*S/R*)-18** using alkene **(3*R*, 36*S*)-CF_1-4** (15 mg, 9.36 μmol) in CH_2Cl_2 (467 μL, 0.02 M), dimethylsulfide (467 uL), $NaClO_2$ (2.5 mg, 2.81 μmol), NaH_2PO_4 (6.7 mg, 56.1 μmol), water (94 μL), *t*-BuOH (187 μL), 2-methyl-2-butene (7.9 mg, 112 μmol). The crude mixture was purified by normal phase flash column chromatography (Biotage HC-10 g, pentane: EtOAc 0-15%) to afford the title compound (8.9 mg, 59%) as a colourless oil.

^{1}H NMR (600 MHz, CDCl3) δ 4.02 – 3.96 (m, 1H, *H*-33), 3.93 – 3.87 (m, 1H, *H*-19), 3.86 – 3.81 (m, 1H, *H*-35), 3.80 – 3.72 (m, 3H, *H*-5, *H*-17 & *H*-21), 3.66 – 3.60 (m, 4H, *H*-9, *H*-13, *H*-25 & *H*-29), 2.35 (dd, *J* = 14.7, 5.5 Hz, 1H, *H*-2'), 2.20 (dd, *J* = 14.7, 8.1 Hz, 1H, *H*-2'), 2.16 – 2.09 (m, 1H *H*-3), 1.77 – 1.02 (m, 88H), 1.01 – 0.92 (m, 66H, CH_3-42 & CH_3-OTES), 0.90 – 0.87 (m, 6H, CH_3-40 & CH_3-41), 0.65 – 0.56 (m, 42H, CH_2-OTES). *See spectrum*.

^{13}C NMR (151 MHz, CDCl3) δ 175.08 (*C*OOH-1), 100.26 (CO anti-acetonide), 72.56 (*C*H-9, *C*H-13, *C*H-25 *or C*H-29), 72.55 (*C*H-9, *C*H-13, *C*H-25 *or C*H-29), 72.42 (2X *C*H-9, *C*H-13, *C*H-25 *or C*H-29), 71.96 (*C*H-33), 71.70 (*C*H-5), 70.65 (*C*H-35), 69.20 (*C*H-21), 66.78 (*C*H-17), 63.81 (*C*H-19), 43.89 (*C*H-4), 43.79 (*C*H_2-20), 41.77 (*C*H-2), 39.32 (α-*C*H_2), 39.07 (α-*C*H_2), 38.38 (α-*C*H_2), 38.18 (α-*C*H_2), 37.78 (α-*C*H_2), 37.72 (α-*C*H_2), 37.70 (α-*C*H_2), 37.66 (α-*C*H_2), 37.58 (α-*C*H_2), 37.57 (α-*C*H_2), 37.53 (α-*C*H_2), 37.41 (α-*C*H_2), 37.23 (α-*C*H_2), 36.62 (α-*C*H_2), 36.26 (α-*C*H_2), 32.67 (*C*H_2-37), 29.88 (*C*H_2-38), 27.18 (*C*H-3), 24.85 (*C*H_3-*anti*-acetonide), 24.83 (*C*H_3-*anti*-acetonide), 23.20 (*C*H_2-39), 21.76 (β-*C*H_2), 21.73 (β-*C*H_2), 21.51 (β-*C*H_2), 21.32 (β-*C*H_2), 21.14 (β-*C*H_2), 20.13 (*C*H_3-42), 14.44 (*C*H_3-40 *or C*H_3-41), 14.29 (*C*H_3-40 *or C*H_3-41), 7.12 (OTES-*C*H_3), 7.10 (OTES-*C*H_3), 7.09 (OTES-*C*H_3), 7.00 (OTES-*C*H_3), 5.33 (OTES-*C*H_2), 5.30 (OTES-*C*H_2), 5.28 (OTES-*C*H_2), 5.25 (OTES-*C*H_2), 5.04 (OTES-*C*H_2). *See spectrum*.

HRMS (m/z): (nanospray) calculated for $C_{87}H_{186}O_{12}Si_7$ [M+Na]+ 1642.2227, found 1642.2216.

TLC: $\boldsymbol{R_f}$ aldehyde = 0.36, $\boldsymbol{R_f}$ product (COOH) = 0.23 (92:8 hexane/EtOAc, stained with *p*-anisaldehyde).

IR (ν_{max}/cm^{-1}, neat): 3361 (OH), 2953, 2935, 2923, 2876, 1711 (C=O), 1459, 1414, 1378, 1238, 1055, 1033, 1009 (Si-O), 726 (Si-C).

$[\alpha]_D^{25}$: −9 (*c* = 0.9, $CHCl_3$).

(1S,5R,9R,13R,15S,19S,21R,25R,29S,33R,35S)-19-((S)-hexan-2-yl)-15,37,37-trimethyl-5,9,13,21,25,29,33-heptakis((triethylsilyl)oxy)-18,36,38-trioxabicyclo[33.3.1]nonatriacontan-17-one (3*S*, 36*S*)-19

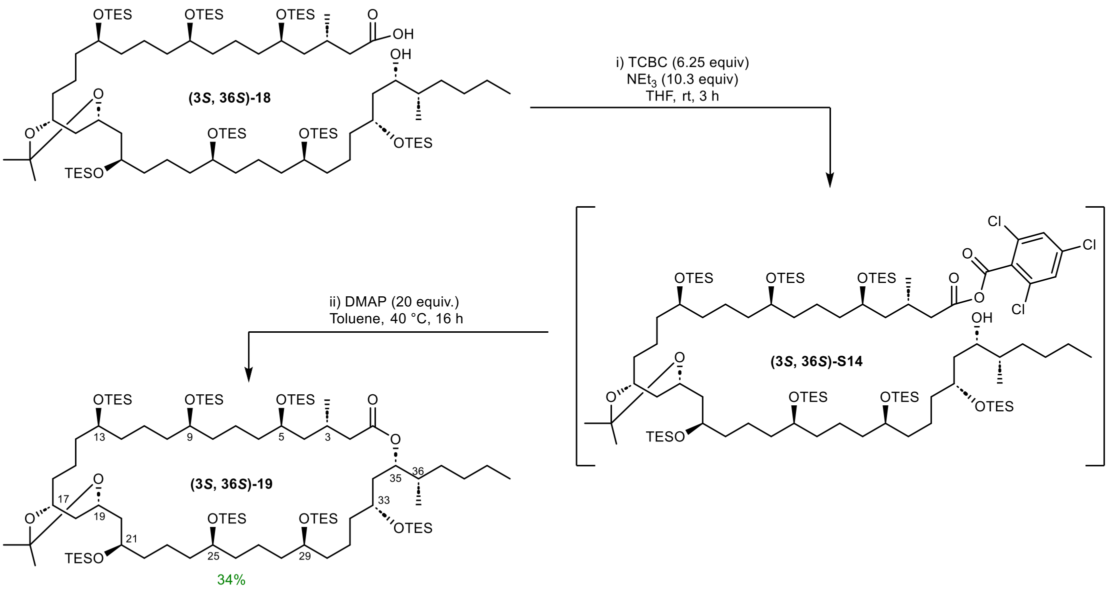

Formation of mixed anhydride (3*S*, 36*S*)-S14

Prepared following the same procedure as described for diastereomer **(3*S*, 36*S*/*R*)-S14** using NEt_3 (0.04 M in THF, 0.95 mL, 38.1 µmol), 2,4,6-trichlorobenzoyl chloride (0.04 M, 0.58 mL, 23.1 µmol) and acid **(3*S*, 36*S*)-18** (6 mg, 3.70 µmol). The reaction mixture was concentrated and used in the next step crude.

Macrolactonisation

Prepared following the same procedure as described for diastereomer **(3*S*, 36*S*/*R*)-19** using mixed anhydride **(3*S*, 36*S*)-S14** in anhydrous toluene (0.002 M, 1.85 mL), DMAP (36.2 mg, 296 µmol) in anhydrous toluene (0.01 M, 7.40 mL). The crude mixture was purified by normal phase flash column chromatography (Biotage HC-10 g, pentane: EtOAc 0-10%) to afford the title compound (2 mg, 34%) as a colourless film.

^{1}H NMR (600 MHz, CDCl3) δ 5.02 – 4.90 (m, 1H, *H*-35), 3.89 (dddd, *J* = 6.8, 6.4, 6.4 Hz, 1H, *H*-19), 3.80 – 3.69 (m, 3H, C*H*OH), 3.67 – 3.58 (m, 5H, C*H*OH), 2.30 (dd, *J* = 14.3, 4.3 Hz, 1H, *H*-2'), 2.14 (m, 1H, *H*-3), 2.09 – 2.02 (dd, *J* = 14.5, 9.3 Hz, 1H, *H*-2''), 1.76 – 1.01 (m, 89H), 1.00 – 0.92 (m, 66H, CH_3-42 & CH_3-OTES), 0.90 – 0.85 (m, 6H, CH_3-40 & CH_3-41), 0.64 – 0.55 (m, 42H, CH_2-OTES). See spectrum.

^{13}C NMR (151 MHz, CDCl3) δ 172.55 (*C*OOR-1), 100.14 (CO anti-acetonide), 75.01 (*C*H-35), 72.58 (*C*H-9, *C*H-13, *C*H-25 *or* *C*H-29), 72.55 (*C*H-9, *C*H-13, *C*H-25 *or* *C*H-29), 72.40

(CH-9, CH-13, CH-25 *or* CH-29), 72.24 (CH-9, CH-13, CH-25 *or* CH-29), 70.23 (CH-5 *or* CH-33) 69.86 (CH-5 *or* CH-33), 69.14 (CH-21), 66.60 (CH-17), 63.86 (CH-19), 44.51 (CH-4), 43.66 (CH_2-20), 42.98 (CH-2), 39.11 (α-CH_2), 38.82 (α-CH_2), 38.66 (α-CH_2), 38.63 (α-CH_2), 37.75 (α-CH_2), 37.72 (α-CH_2), 37.71 (α-CH_2), 37.68 (α-CH_2), 37.65 (α-CH_2), 37.64 (α-CH_2), 37.48 (α-CH_2), 37.42 (α-CH_2), 37.17 (α-CH_2), 36.95 (CH-36), 36.24 (α-CH_2), 32.12 (CH_2-37), 29.79 (CH_2-38), 26.93 (CH-3), 25.00 (CH_3-*anti*-acetonide), 24.94 (CH_3-*anti*-acetonide), 23.05 (CH_2-39), 21.55 (β-CH_2), 21.49 (β-CH_2), 21.29 (2X β-CH_2), 21.18 (β-CH_2), 20.91 (β-CH_2), 19.69 (CH_3-42), 14.98 (CH_3-40 *or* CH_3-41), 14.22 (CH_3-40 *or* CH_3-41), 7.17 (OTES-CH_3), 7.15 (OTES-CH_3), 7.13 (OTES-CH_3), 7.11 (OTES-CH_3), 5.40 (OTES-CH_2), 5.32 (OTES-CH_2), 5.30 (OTES-CH_2), 5.26 (OTES-CH_2). See spectrum.

HRMS (m/z): (nanospray) calculated for $C_{87}H_{184}O_{11}Si_7$ [M+Na]+ 1624.2121 found 1642.2106.

TLC: R_f = 0.36 (95:5 hexane/EtOAc, stained with ceric ammonium molybdate (CAM)).

IR (ν_{max}/cm^{-1}, neat): 2952, 2936, 2914, 2876, 1732 (C=O), 1459, 1378, 1238, 1008 (Si-O), 726 (Si-C).

$[\alpha]_D^{25}$: +20 (c = 0.2, $CHCl_3$).

(4S,6R,10R,14S,18R,22R,26S,30R,34R,36S)-36-((S)-hexan-2-yl)-6,10,14,18,20,22,26,30,34-nonahydroxy-4-methyloxacyclohexatriacontan-2-one ((3*S*, 36*S*)-CA)

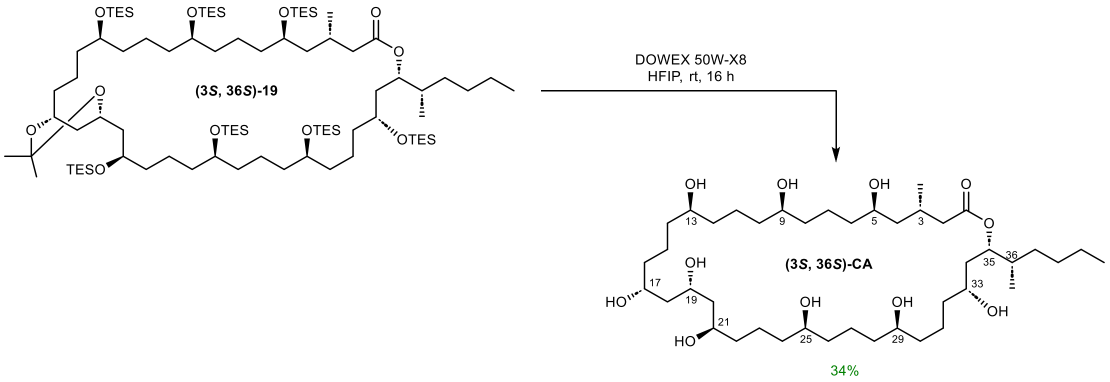

Prepared following the same procedure as described for diastereomer **(3*S*, 36*S*/*R*)-CA** using acidic resin DOWEX-50W X8 (8 mg), protected caylobolide A **(3*S*, 36*S*)-19** (2 mg, 1.25 µmol) in hexafluoroisopropanol (0.003 M, 0.42 mL). The crude product was purified by

reverse phase preparative HPLC portion-wise (8 x) to give analytically pure caylobolide A (0.32 mg, 34%) as a white powder.

<u>All analytical data matched that of the natural product</u>

Reverse phase prep-HPLC trace for caylobolide A, product isolated was the major component (retention time: 12.31 minutes). Water-acetonitrile, 5-95% gradient, 20 minutes using an analytical column.

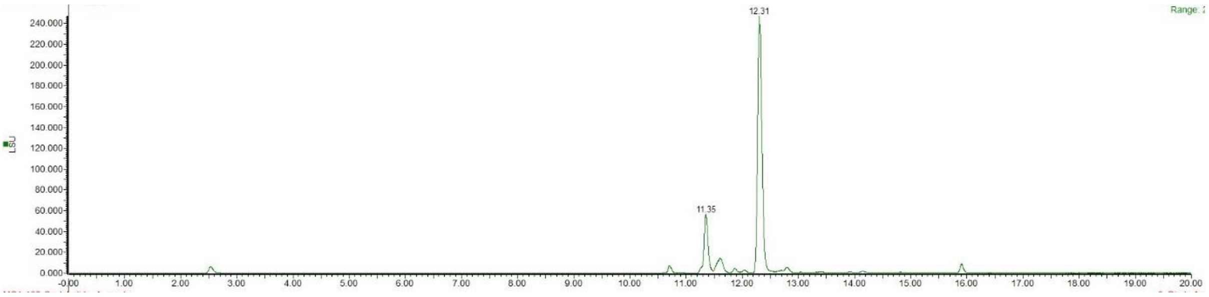

Mass trace for peak at 12.31 minutes matched that of caylobolide A [M+H]+.

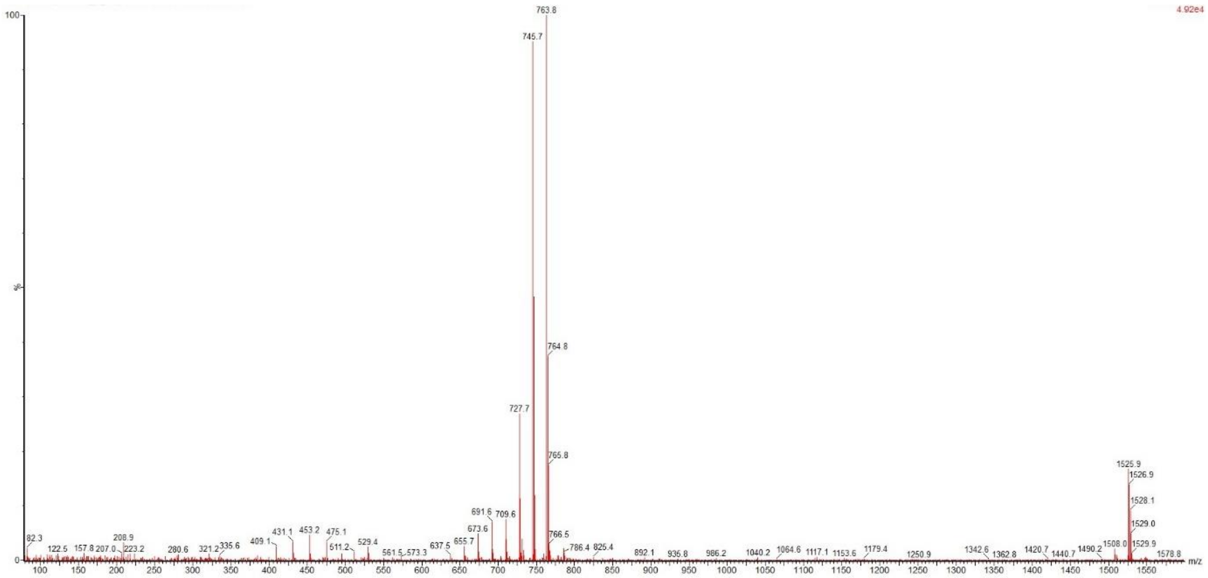

^{1}H NMR (700 MHz, Pyr) δ 6.27 (d, J = 4.2 Hz, 1H), 6.15 (t, J = 4.1 Hz, 1H), 6.00 (d, J = 5.1 Hz, 1H), 5.92 (d, J = 6.0 Hz, 1H), 5.80 (d, J = 6.0 Hz, 1H), 5.75 (qt, J = 8.5, 3.8 Hz, 4H), 4.75 (s, 1H), 4.47 (s, 1H), 4.28 (s, 1H), 4.04 (s, 1H), 4.02 – 3.87 (m, 5H), 2.75 (s, 1H), 2.65 (dd, J = 14.7, 5.7 Hz, 1H), 2.39 (dd, J = 14.6, 8.4 Hz, 1H), 2.20 – 1.60 (m, 43H), 1.58 – 1.52 (m, 1H), 1.50 – 1.42 (m, 1H), 1.40 – 1.19 (m, 8H), 1.17 (d, J = 6.6 Hz, 3H), 1.00 (d, J = 7.0 Hz, 3H), 0.85 (t, J = 7.0 Hz, 3H). See spectrum.

Exchangeable alcohol (OH) chemical shifts move significantly with varying sample water content obscuring the lactone OC-H.

^{13}C NMR (151 MHz, Pyr) δ 173.78, 75.14, 71.51, 71.37, 71.27, 71.10, 70.95, 69.20, 68.98, 68.56, 67.94, 45.58, 45.46, 44.98, 43.59, 40.58, 39.80, 39.69, 39.10, 39.01, 38.92, 38.88, 38.78, 38.69, 38.58, 38.56, 38.27, 37.92, 33.07, 30.05, 28.12, 23.46, 23.32, 23.15, 23.10, 23.07, 22.51, 22.45, 20.09, 15.42, 14.54. See spectrum.

HRMS (m/z): (ESI) calculated for $C_{42}H_{82}O_{11}$ [M+H]+ 763.5930, found 763.5916.

IR (ν_{max}/cm^{-1}, neat): 3341 (OH), 2953, 2917, 2861, 1737 (C=O), 1596, 1454, 1378, 1229, 1119, 1049, 896.

4.9.1 NMR comparison table

Table S6: ^{13}C NMR comparison of caylobolide A to synthetic caylobolide A. Spectra recorded in pyridine-*d*5, referenced to 123.87 ppm

Atom number	Caylobolide A (^{13C})	Synthetic Caylobolide A (^{13C})	Δδ13C (natural-synthetic)
**1**	173.78	173.78	0
**2**	43.58	43.59	-0.01
**3**	28.12	28.12	0
**4**	45.57	45.58	-0.01
**5**	68.99	68.98	0.01
**6**	39.79	39.8	-0.01
**7**	23.05	23.07	-0.02
**8**	38.91	38.92	-0.01
**9**	71.38	71.37	0.01
**10**	38.77	38.78	-0.01
**11**	23.09	23.1	-0.01
**12**	38.55	38.56	-0.01
**13**	71.1	71.1	0
**14**	38.26	38.27	-0.01
**15**	22.44	22.45	-0.01
**16**	38.68	38.69	-0.01
**17**	68.57	68.56	0.01
**18**	44.97	44.98	-0.01
**19**	69.19	69.2	-0.01
**20**	45.45	45.46	-0.01
**21**	70.95	70.95	0
**22**	39.09	39.1	-0.01
**23**	22.5	22.51	-0.01
**24**	38.57	38.59	-0.02
**25**	71.27	71.27	0
**26**	38.77	38.78	-0.01
**27**	23.13	23.15	-0.02

**28**	38.86	38.88	-0.02
**29**	71.51	71.51	0
**30**	38.78	38.78	0
**31**	23.31	23.32	-0.01
**32**	39.67	39.69	-0.02
**33**	67.95	67.94	0.01
**34**	40.58	40.58	0
**35**	75.15	75.14	0.01
**36**	37.92	37.92	0
**37**	33.07	33.07	0
**38**	30.05	30.05	0
**39**	23.45	23.46	-0.01
**40**	14.53	14.54	-0.01
**41**	15.42	15.42	0
**42**	20.09	20.09	0

5 REFERENCES

1. Y. Kobayashi, C.-H. Tan and Y. Kishi, *Angew. Chem. Int. Ed.*, 2000, **39**, 4279-4281.
2. Y. Kobayashi, C.-H. Tan and Y. Kishi, *Helv. Chim. Acta*, 2000, **83**, 2562-2571.
3. Y. Kobayashi, W. Czechtizky and Y. Kishi, *Org. Lett.*, 2003, **5**, 93-96.
4. S. Higashibayashi, W. Czechtizky, Y. Kobayashi and Y. Kishi, *J. Am. Chem. Soc.*, 2003, **125**, 14379-14393.
5. C.-L. Shao, R. G. Linington, M. J. Balunas, A. Centeno, P. Boudreau, C. Zhang, N. Engene, C. Spadafora, T. S. Mutka, D. E. Kyle, L. Gerwick, C.-Y. Wang and W. H. Gerwick, *The Journal of organic chemistry*, 2015, **80**, 7849-7855.
6. K. Konno, T. Fujishima, Z. Liu and H. Takayama, *Chirality*, 2002, **14**, 72-80.
7. J. Seco, M. Martino, E. Quiñoá and R. Riguera, *Organic Letters - ORG LETT*, 2000, **2**, 3261-3264.
8. F. Freire, J. M. Seco, E. Quiñoá and R. Riguera, *The Journal of Organic Chemistry*, 2005, **70**, 3778-3790.
9. G. Casoni, M. Kucukdisli, J. M. Fordham, M. Burns, E. L. Myers and V. K. Aggarwal, *J. Am. Chem. Soc.*, 2017, **139**, 11877-11886.
10. Y. Yamamoto, R. Fujikawa, T. Umemoto and N. Miyaura, *Tetrahedron*, 2004, **60**, 10695-10700.
11. L. T. Kliman, S. N. Mlynarski and J. P. Morken, *J. Am. Chem. Soc.*, 2009, **131**, 13210-13211.
12. D. Fiorito, S. Keskin, J. M. Bateman, M. George, A. Noble and V. K. Aggarwal, *J. Am. Chem. Soc.*, 2022, **144**, 7995-8001.
13. P. Beak and L. G. Carter, *The Journal of Organic Chemistry*, 1981, **46**, 2363-2373.
14. M. Burns, S. Essafi, J. R. Bame, S. P. Bull, M. P. Webster, S. Balieu, J. W. Dale, C. P. Butts, J. N. Harvey and V. K. Aggarwal, *Nature*, 2014, **513**, 183-188.

6 SPECTRAL APPENDIX

^{1}H NMR (400 MHz, $CDCl_3$) of compound **1**. *See procedure*.

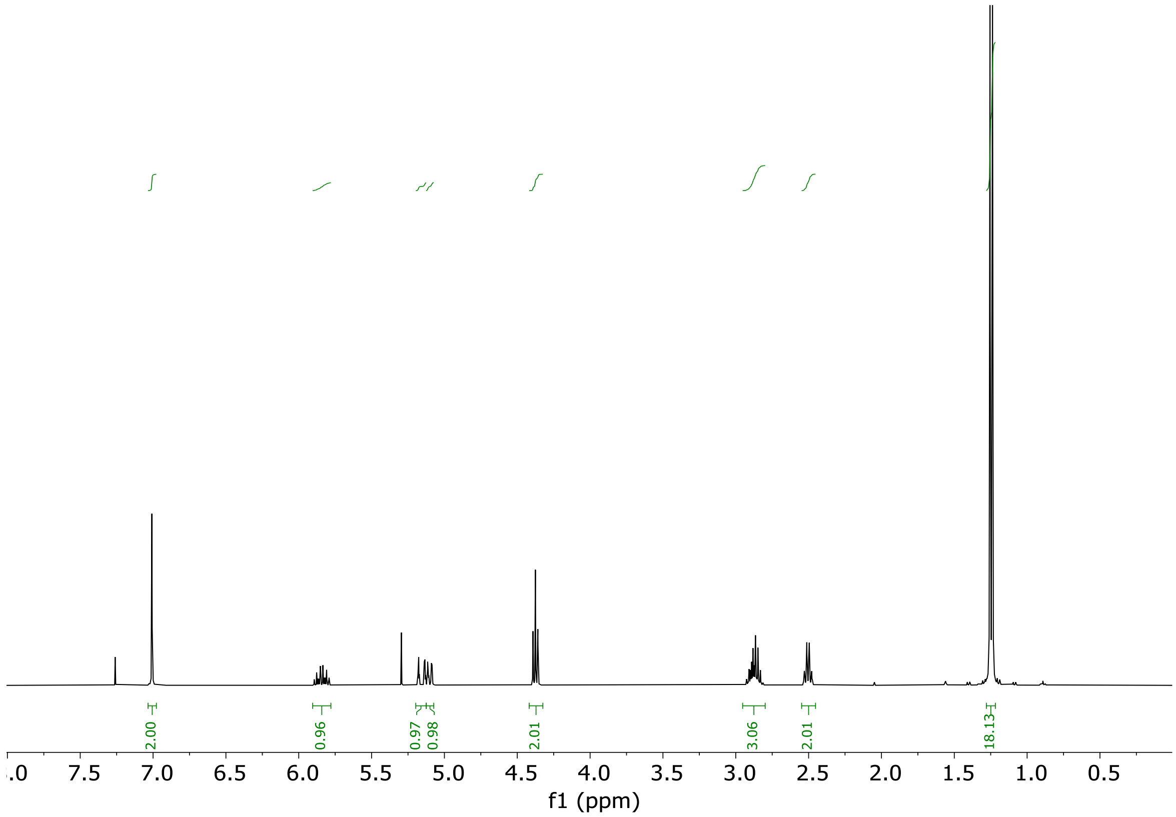

^{13}C NMR (101 MHz, $CDCl_3$) of compound **1**. *See procedure*.

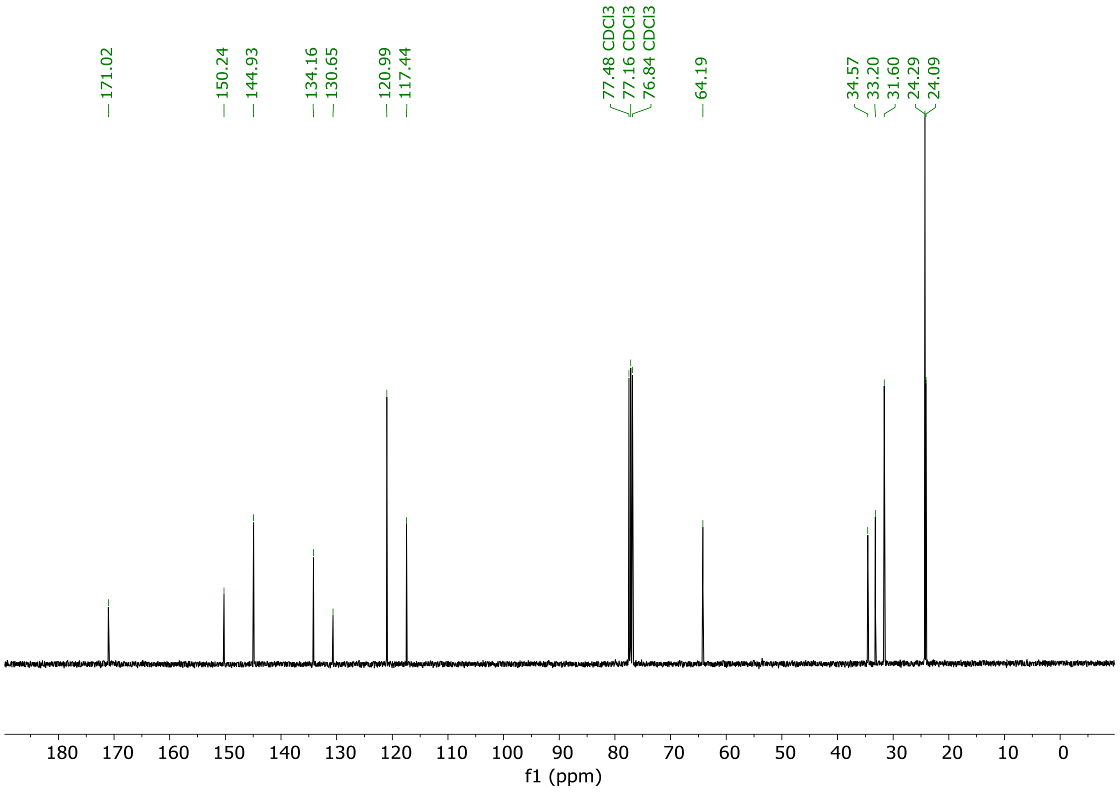

^{1}H NMR (400 MHz, CDCl3) of compound **S1-*syn***. See procedure.

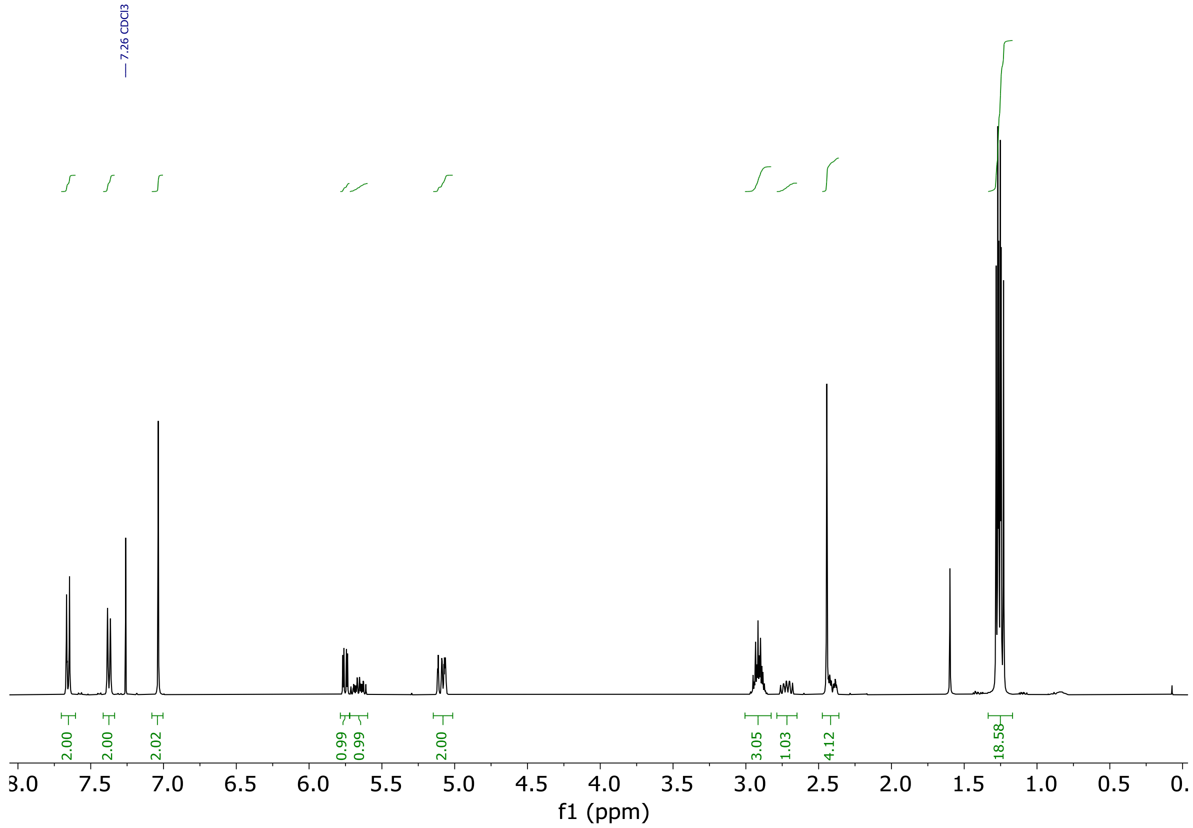

^{13}C NMR (101 MHz, $CDCl_3$) of compound **S1-*syn***. See procedure.

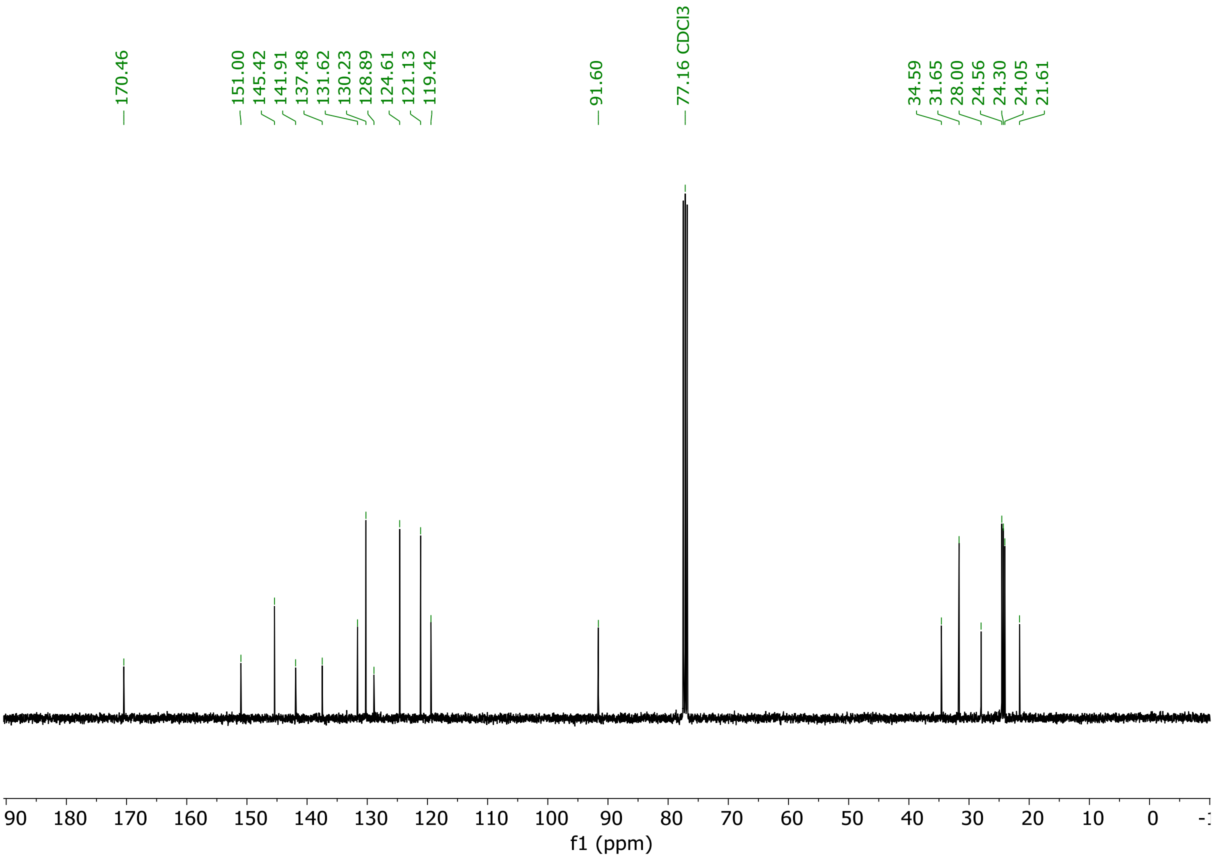

^{1}H NMR (400 MHz, CDCl3) of compound **S1-*anti***. See procedure.

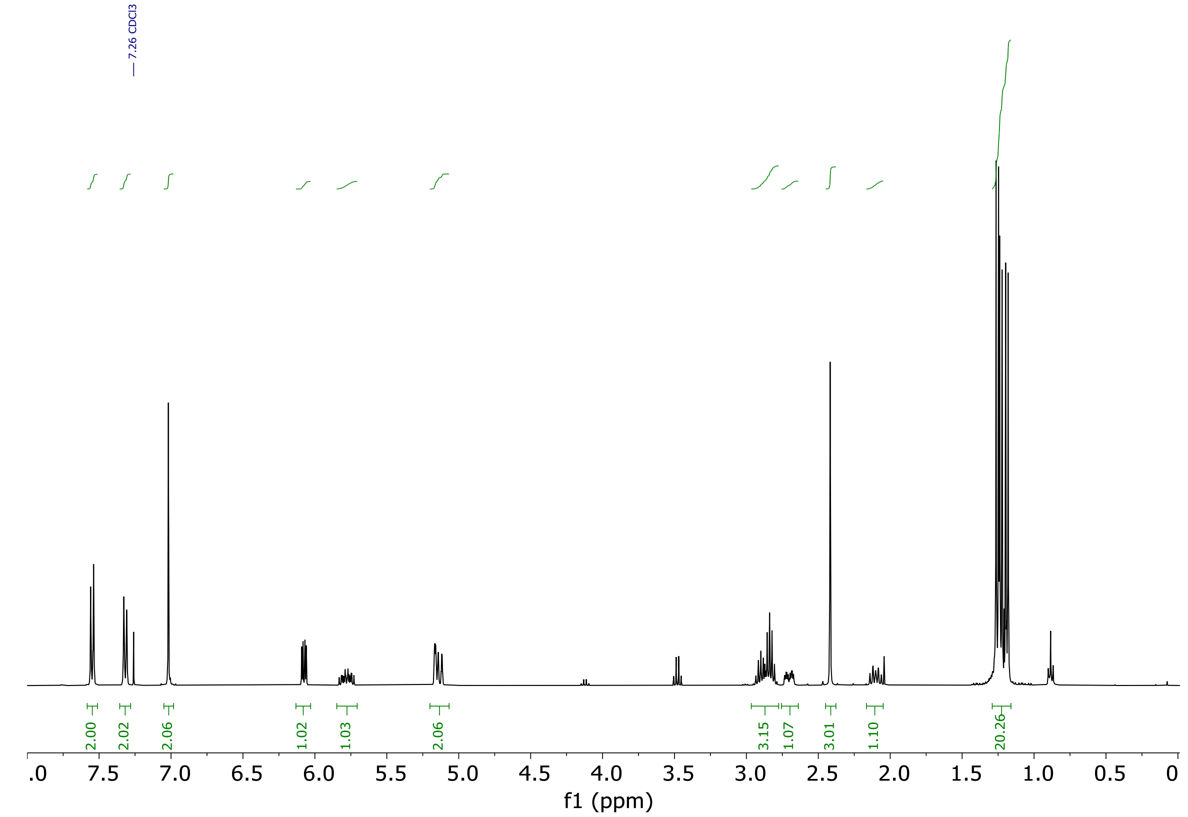

^{13}C NMR (101 MHz, $CDCl_3$) of compound **S1-*anti***. See procedure.

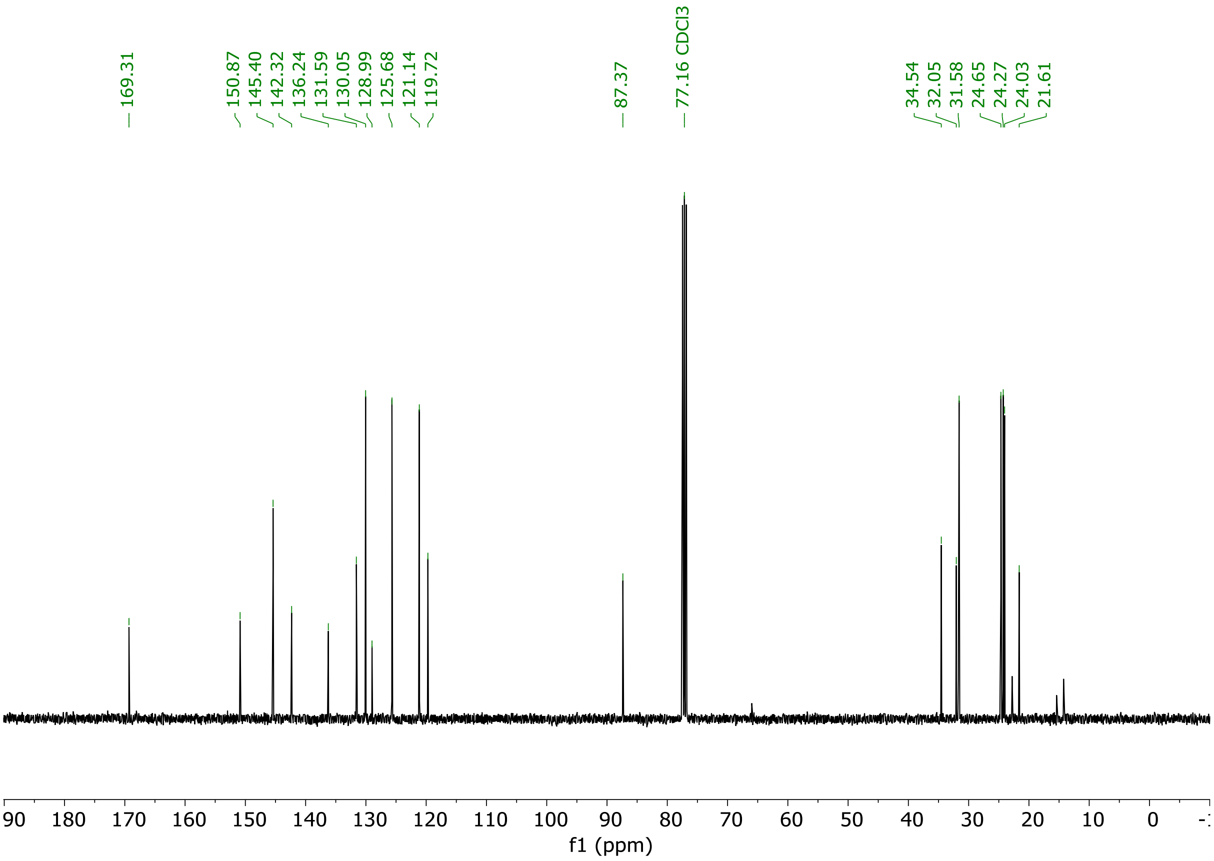

^{1}H NMR (400 MHz, $CDCl_3$) of compound **(3*S*)-S2**. See procedure.

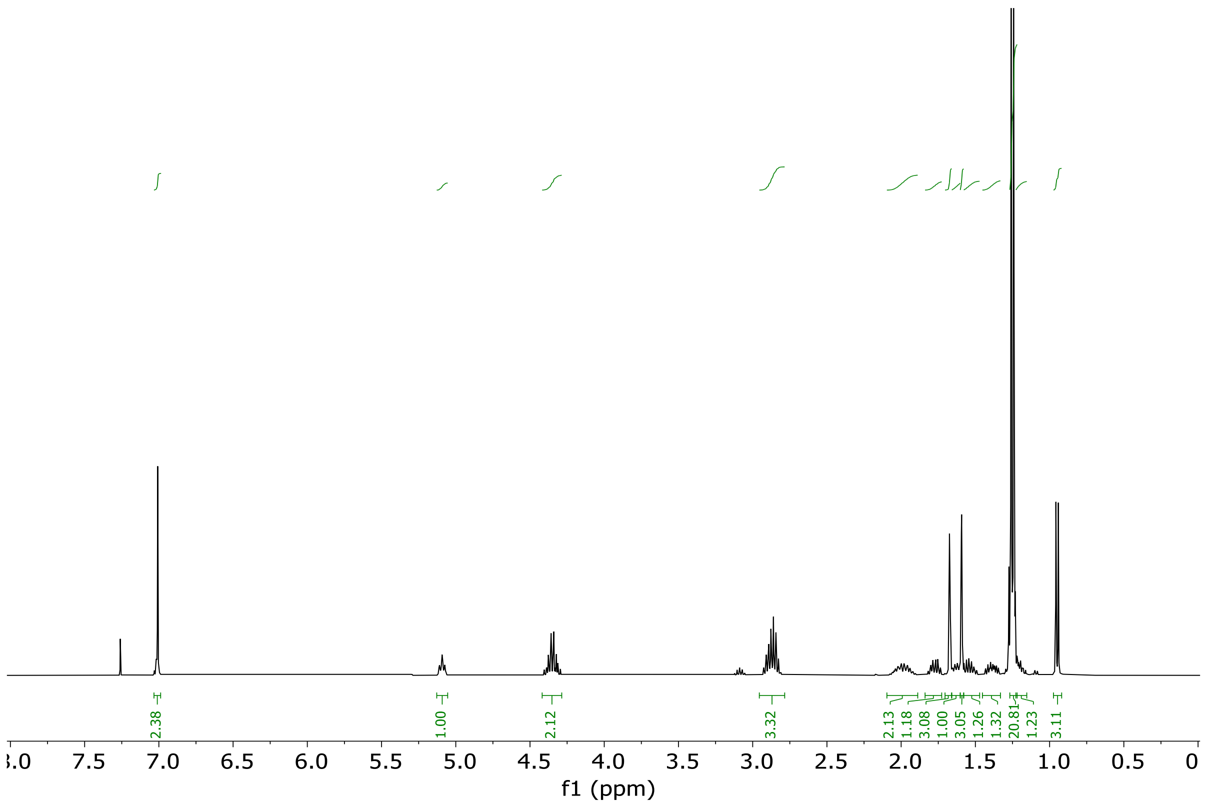

^{13}C NMR (101 MHz, $CDCl_3$) of compound **(3*S*)-S2**. See procedure.

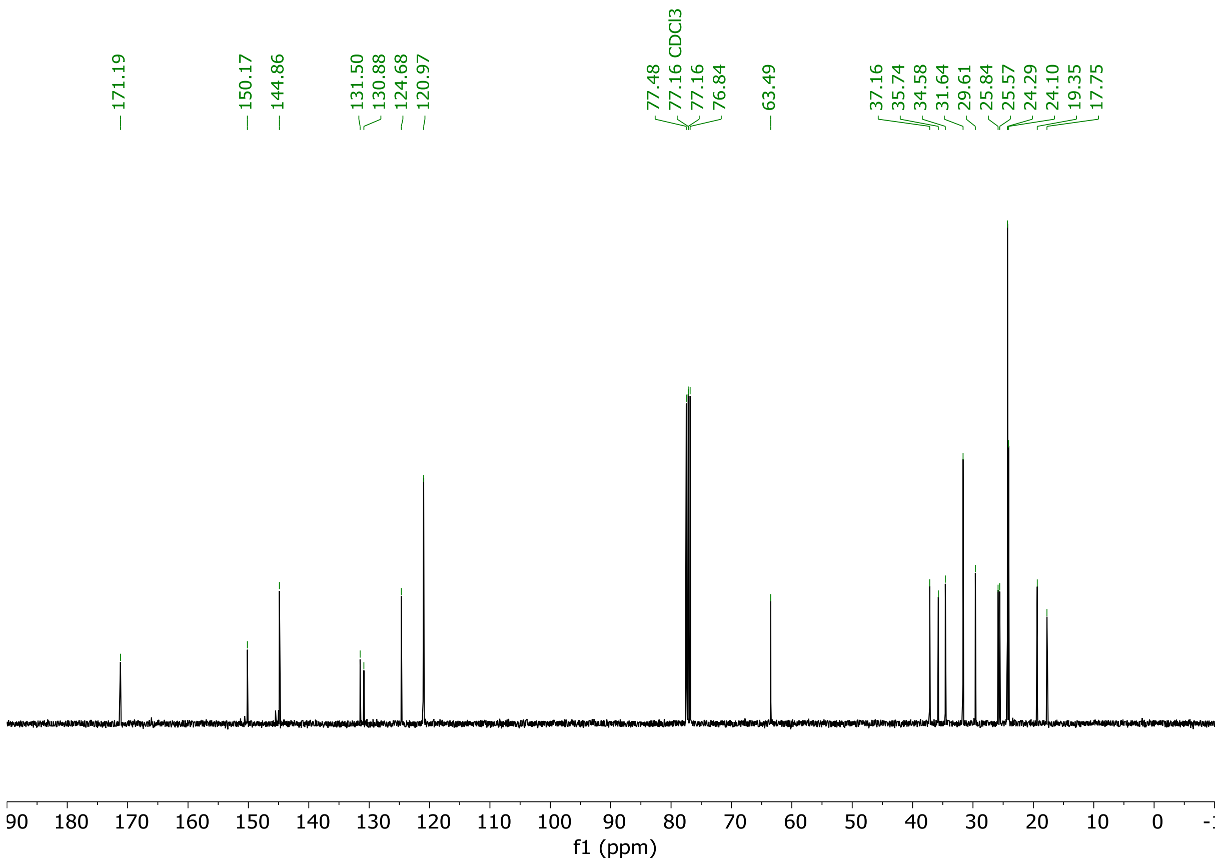

^{1}H NMR (400 MHz, $CDCl_3$) of compound **(3*S*)-S3**. See procedure.

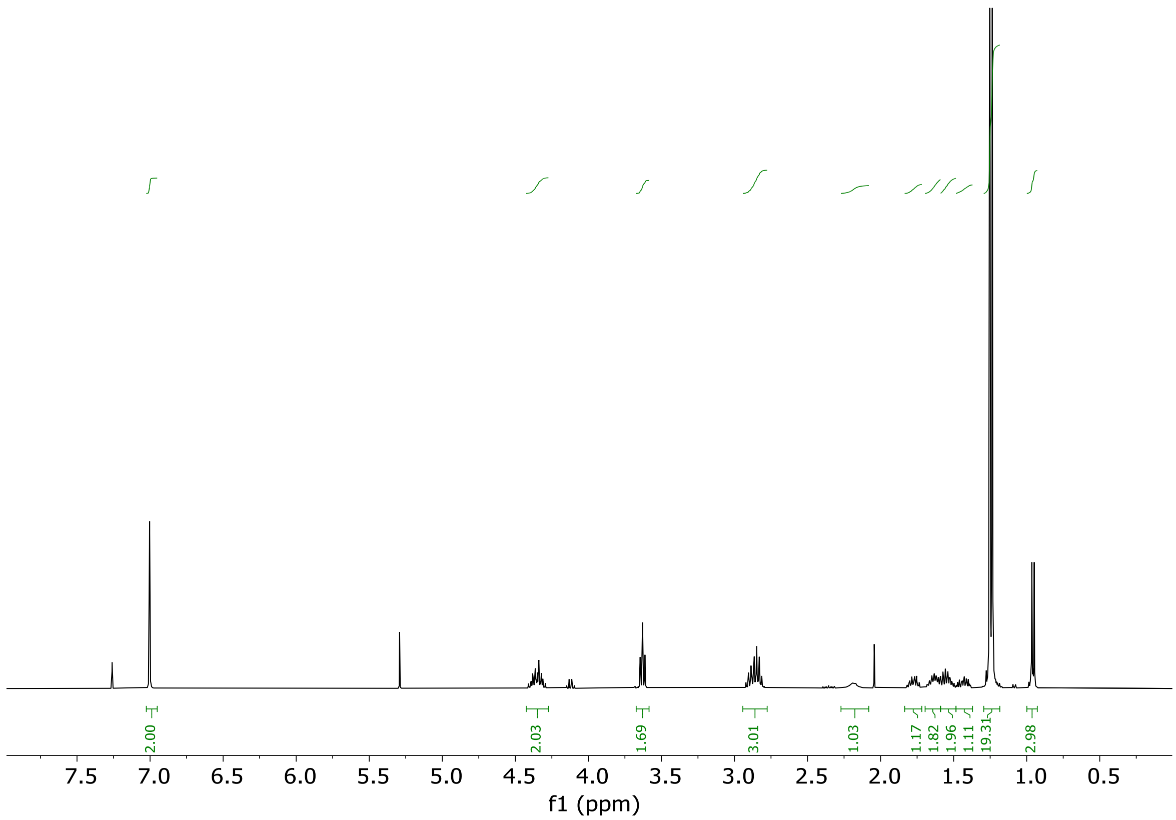

^{13}C NMR (101 MHz, $CDCl_3$) of compound **(3*S*)-S3**. See procedure.

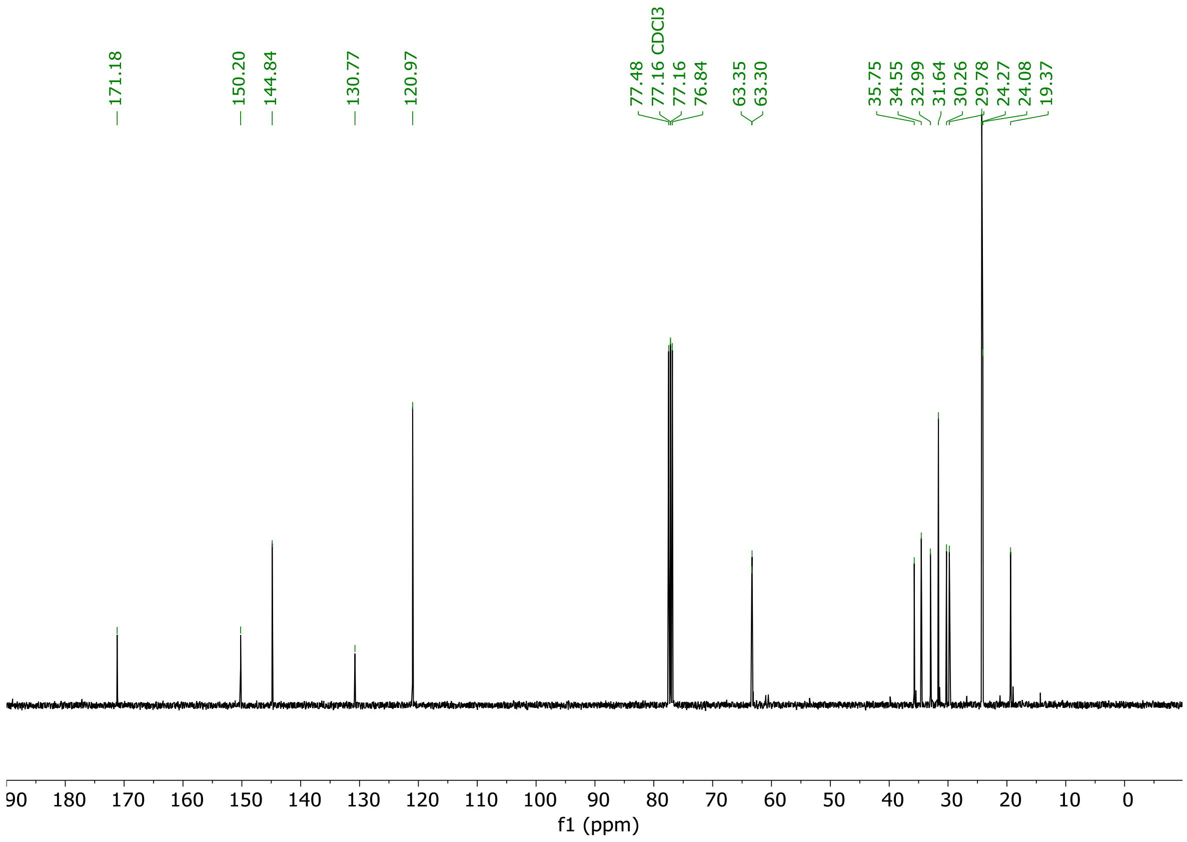

^{1}H NMR (400 MHz, $CDCl_3$) of compound **(3*S*)-S4**. [See procedure.](#)

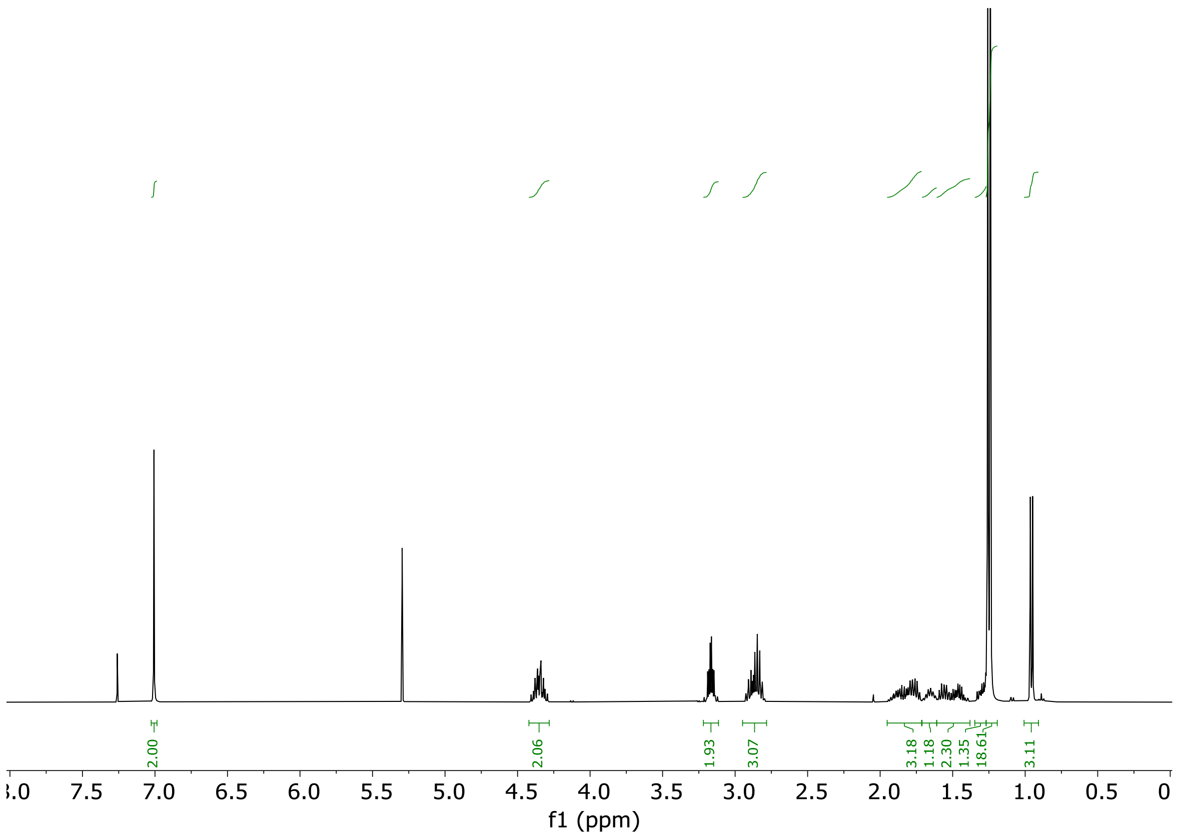

^{13}C NMR (101 MHz, $CDCl_3$) of compound **(3*S*)-S4**. [See procedure.](#)

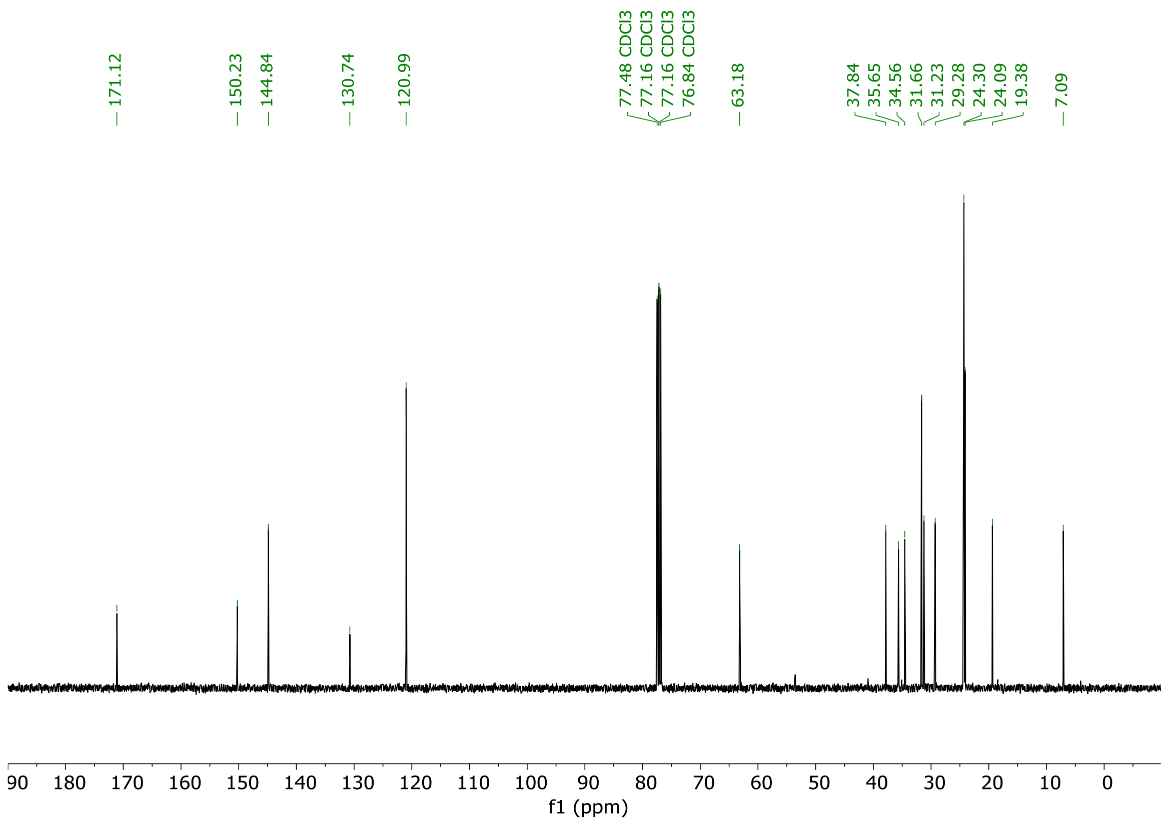

^{1}H NMR (400 MHz, $CDCl_3$) of compound **(3*S*)-S5.** See procedure.

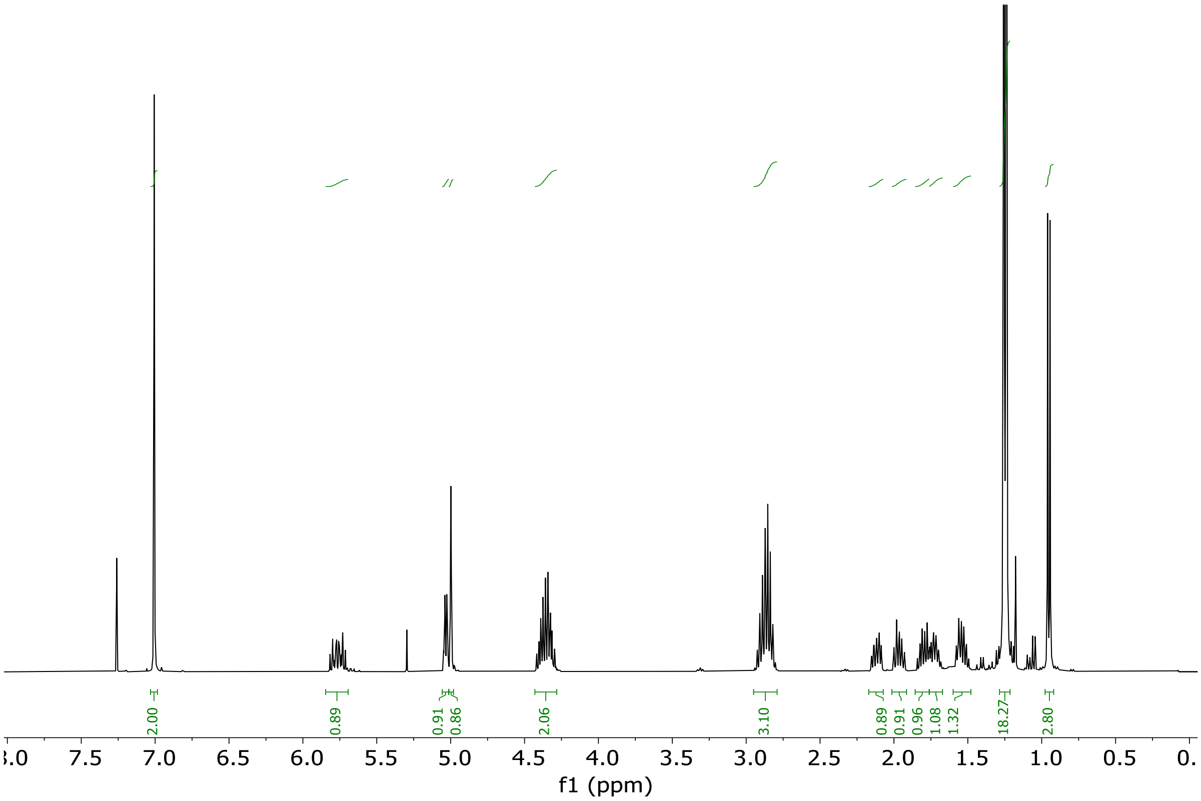

^{13}C NMR (101 MHz, $CDCl_3$) of compound **(3*S*)-S5**. See procedure.

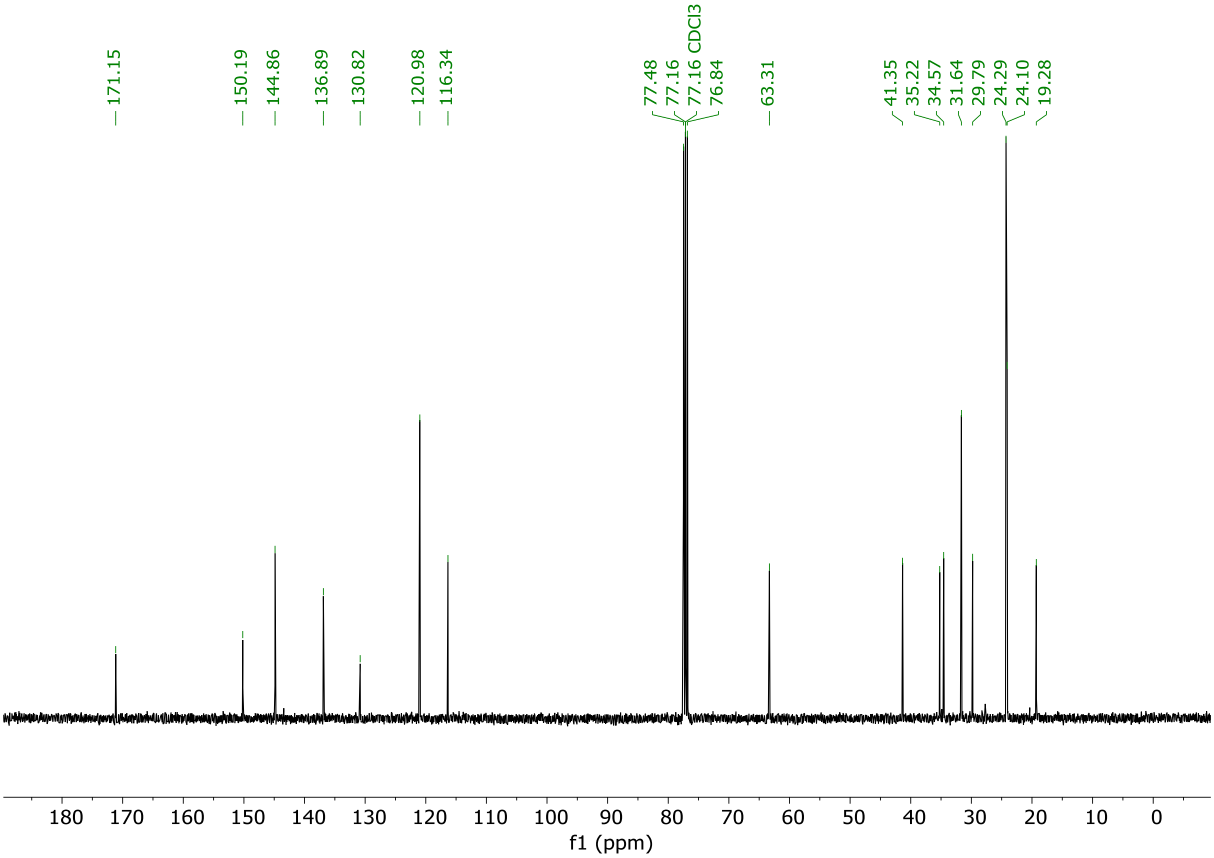

^{1}H NMR (400 MHz, $CDCl_3$) of compound **(3*S*)-6**. See procedure.

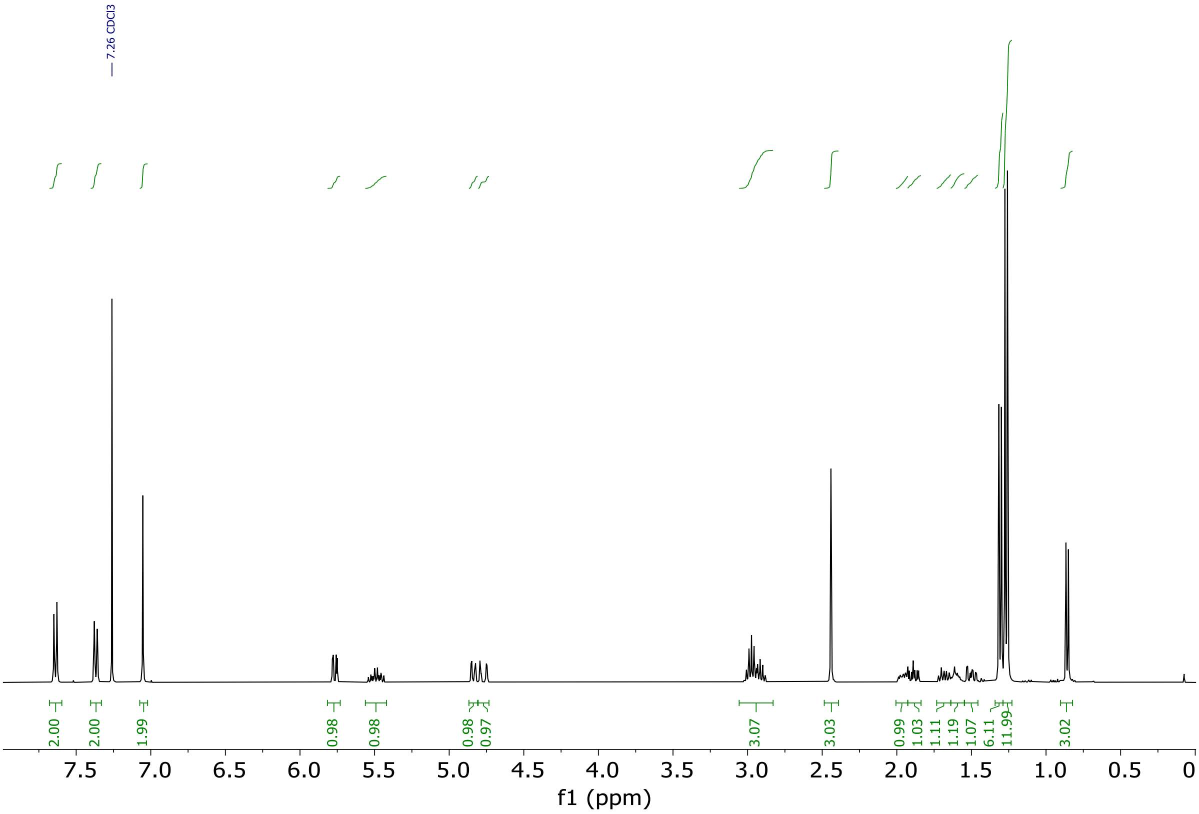

^{13}C NMR (101 MHz, $CDCl_3$) of compound **(3*S*)-6**. See procedure.

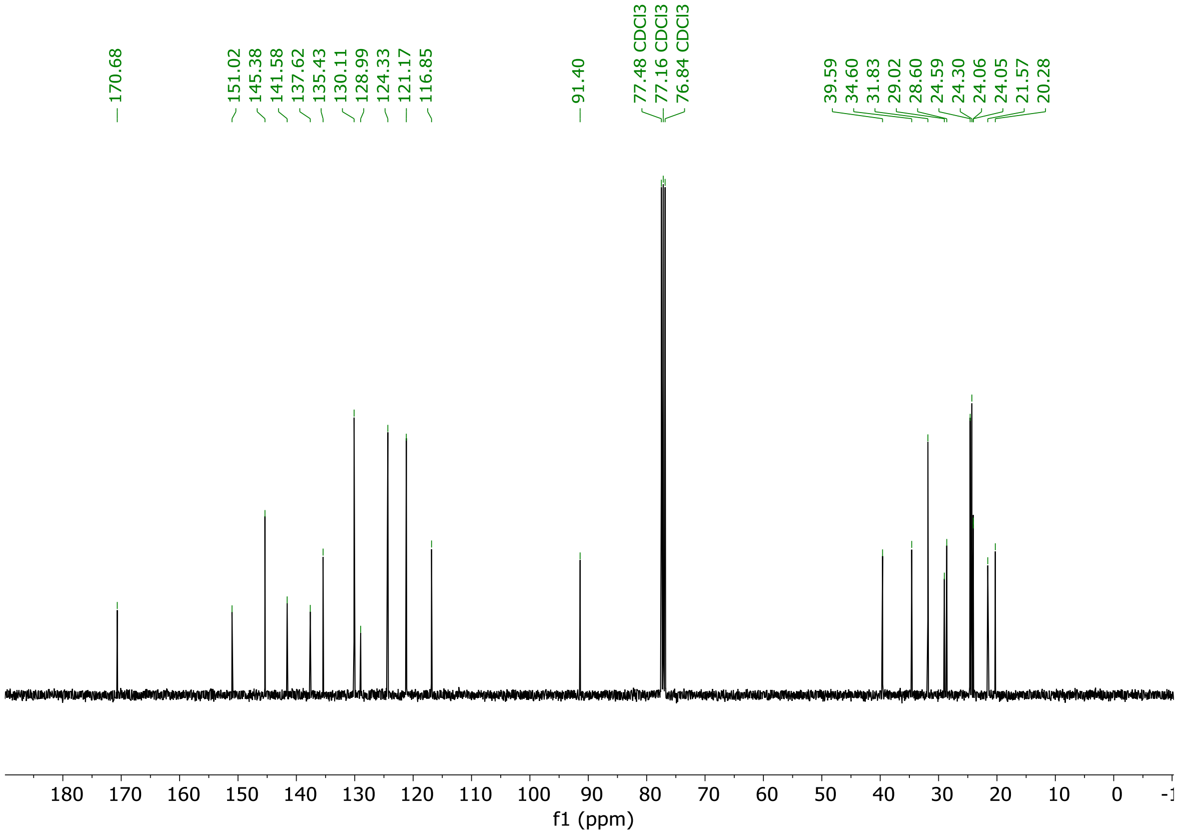

^{1}H NMR (400 MHz, $CDCl_3$) of compound **(3*R*)-6**. *See procedure.*

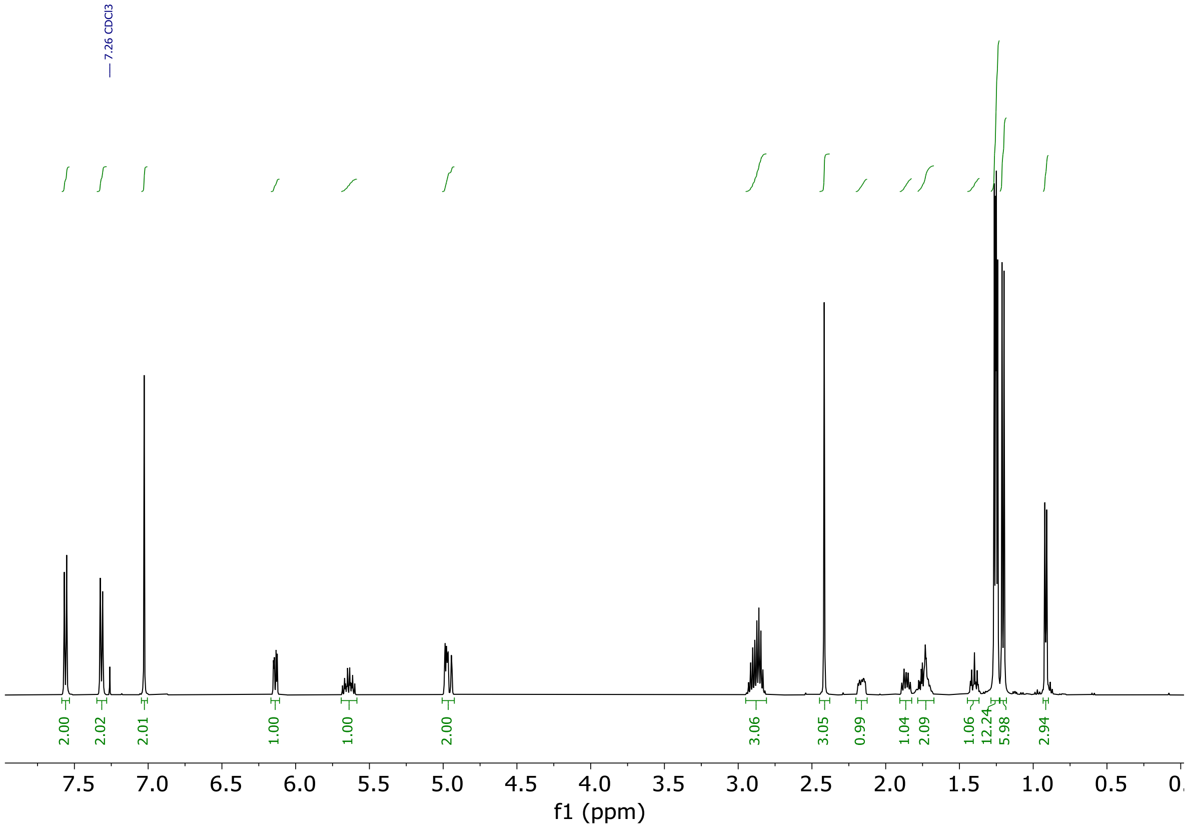

^{13}C NMR (101 MHz, $CDCl_3$) of compound **(3*R*)-6**. *See procedure.*

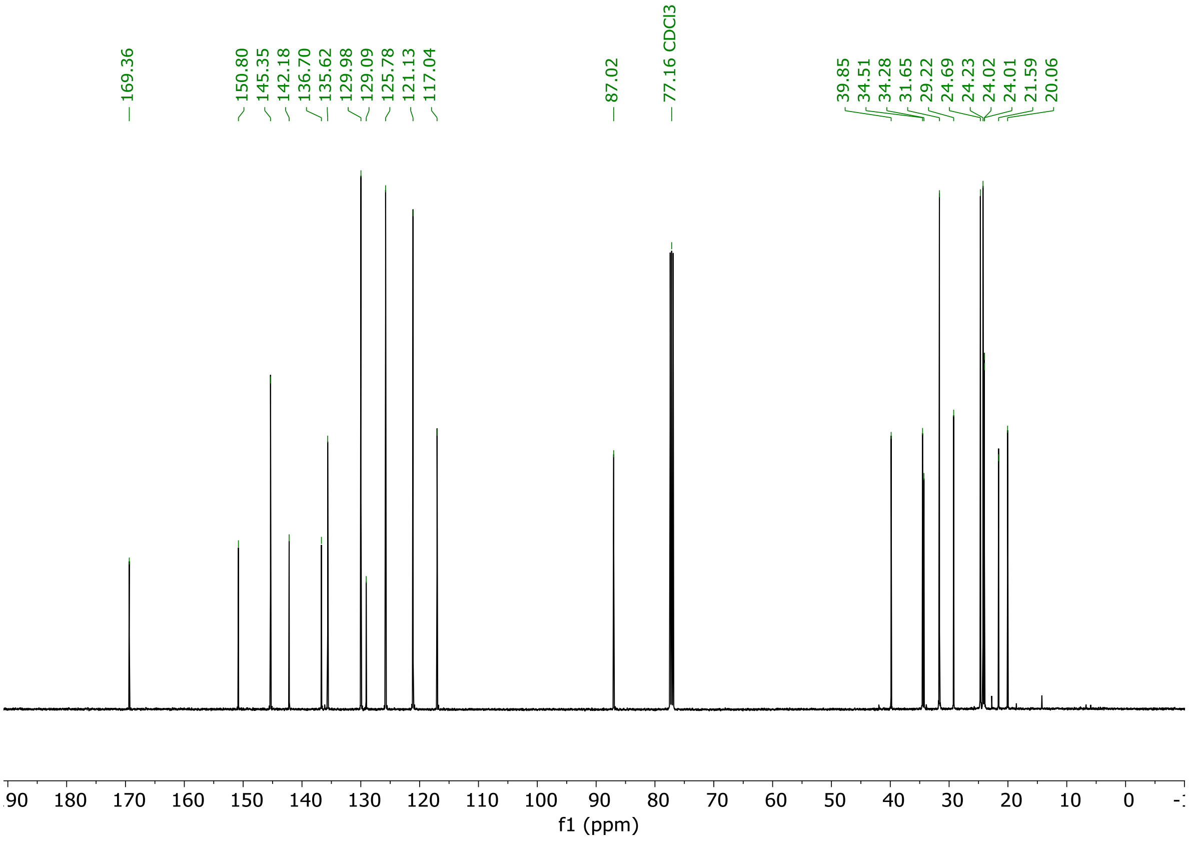

^{1}H NMR (400 MHz, $CDCl_3$) of compound **2**. *See procedure*.

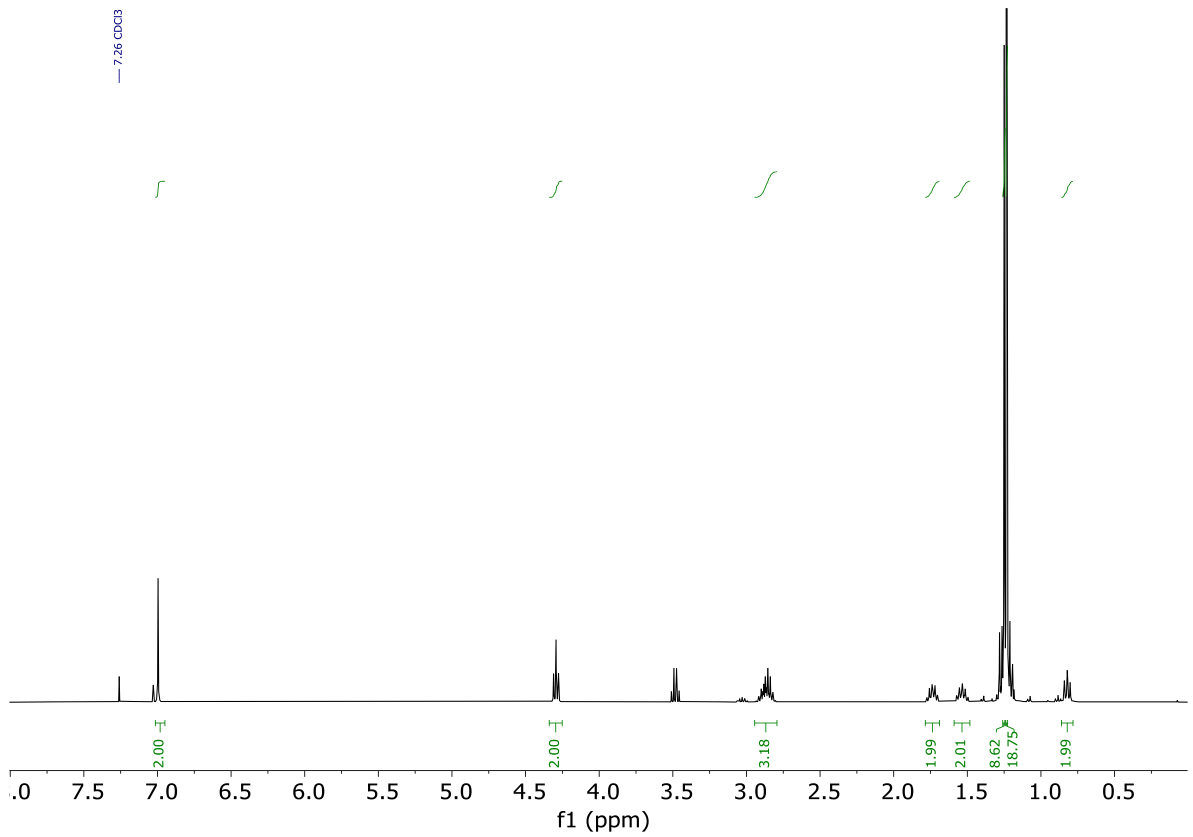

^{13}C NMR (101 MHz, $CDCl_3$) of compound **2**. *See procedure*.

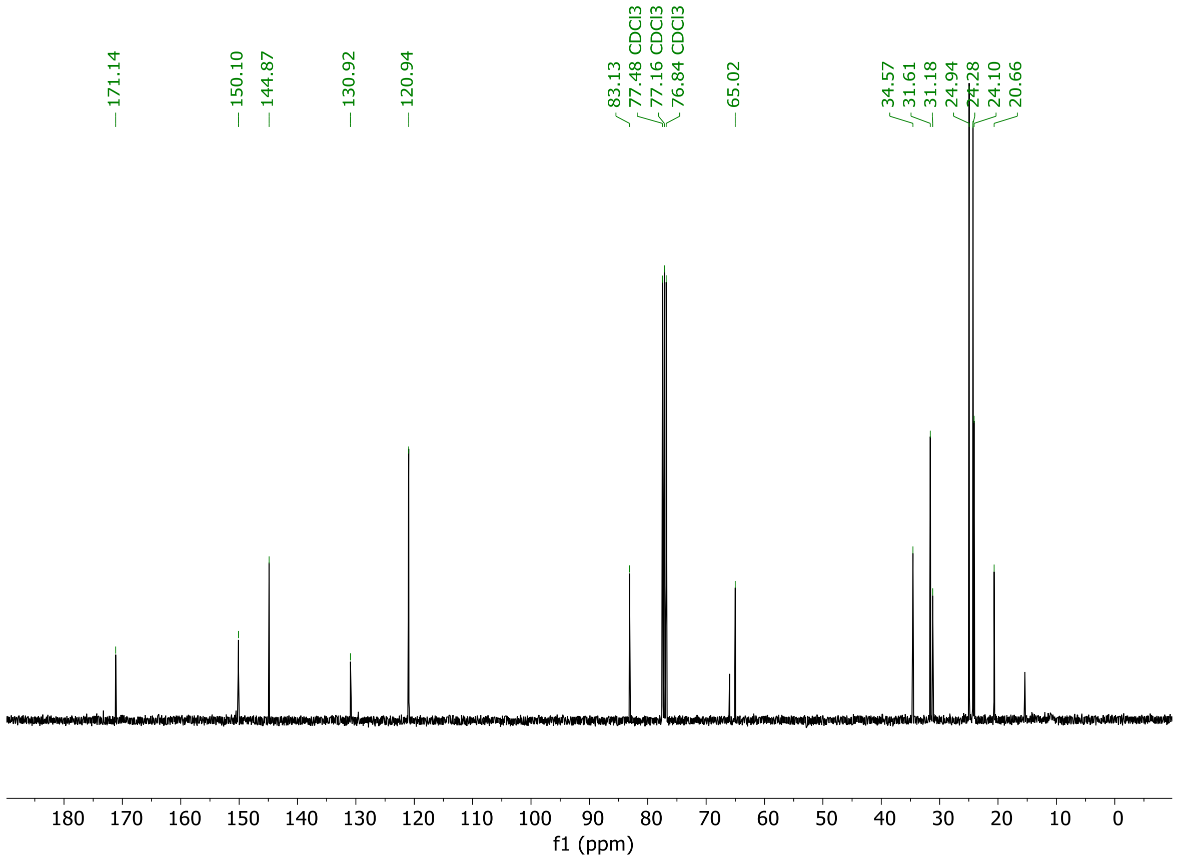

^{1}H NMR (400 MHz, $CDCl_3$) of compound **3**. See procedure.

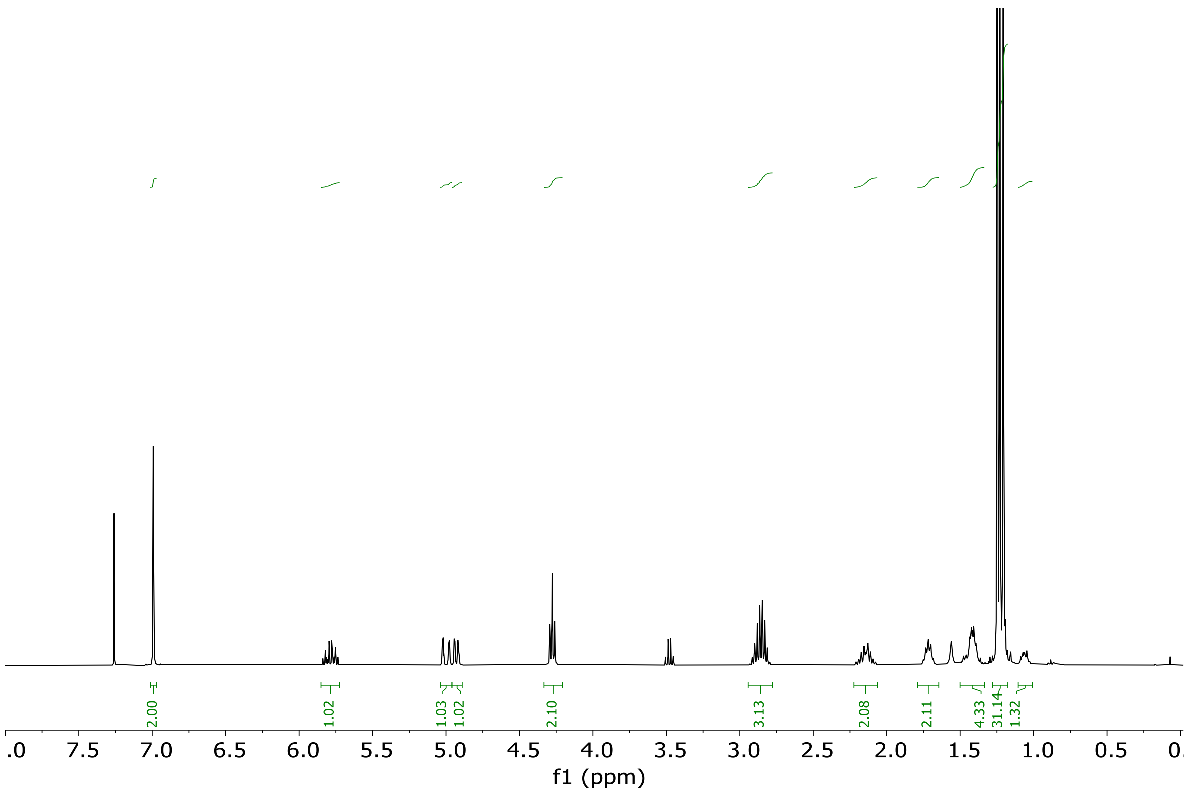

^{13}C NMR (101 MHz, $CDCl_3$) of compound **3**. See procedure.

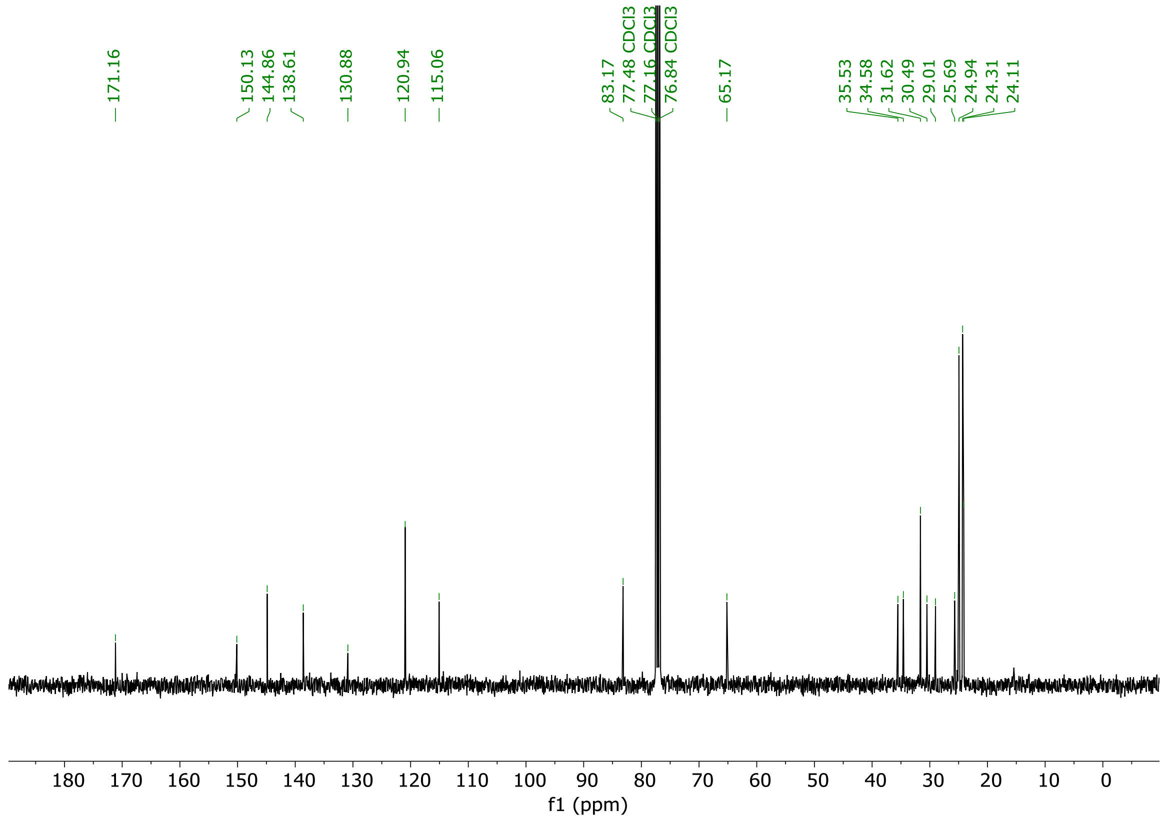

^{1}H NMR (400 MHz, $CDCl_3$) of compound **4**. <u>*See procedure*</u>.

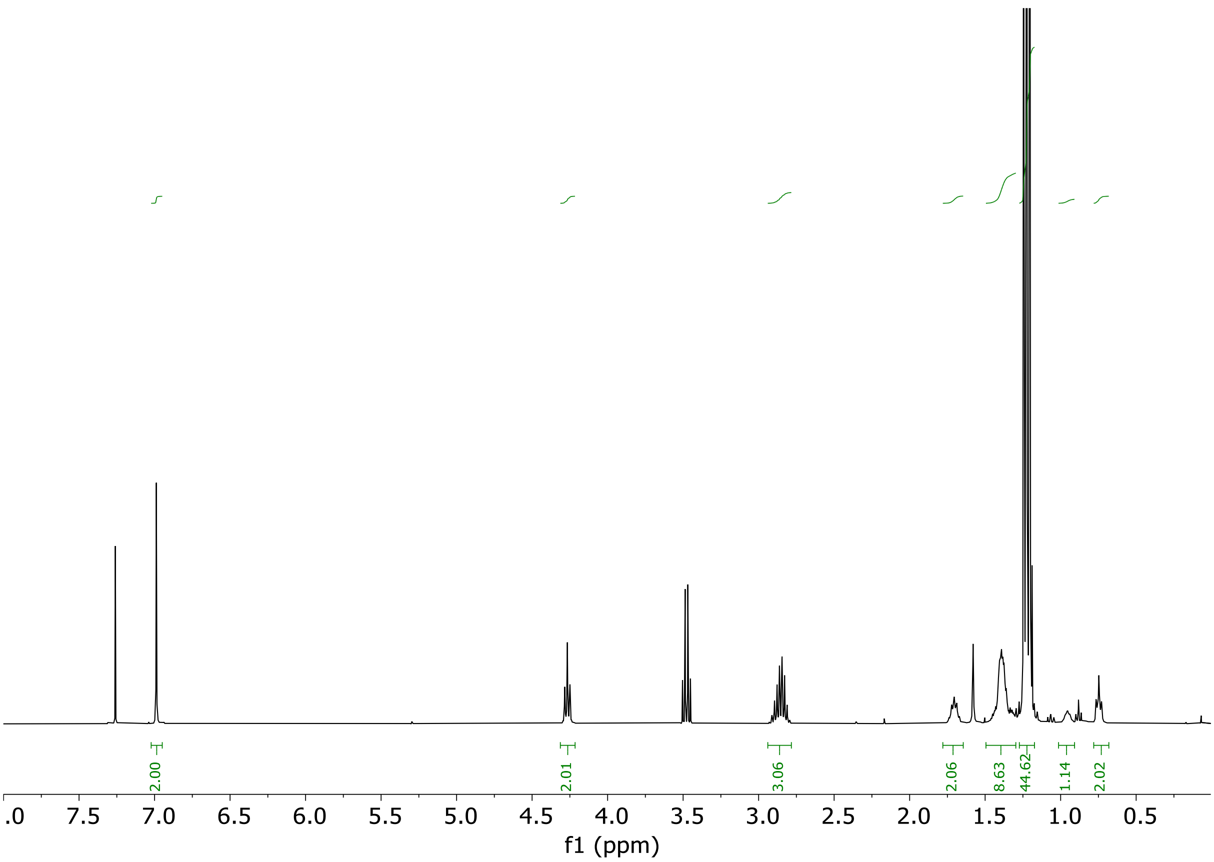

^{13}C NMR (101 MHz, $CDCl_3$) of compound **4**. <u>*See procedure*</u>.

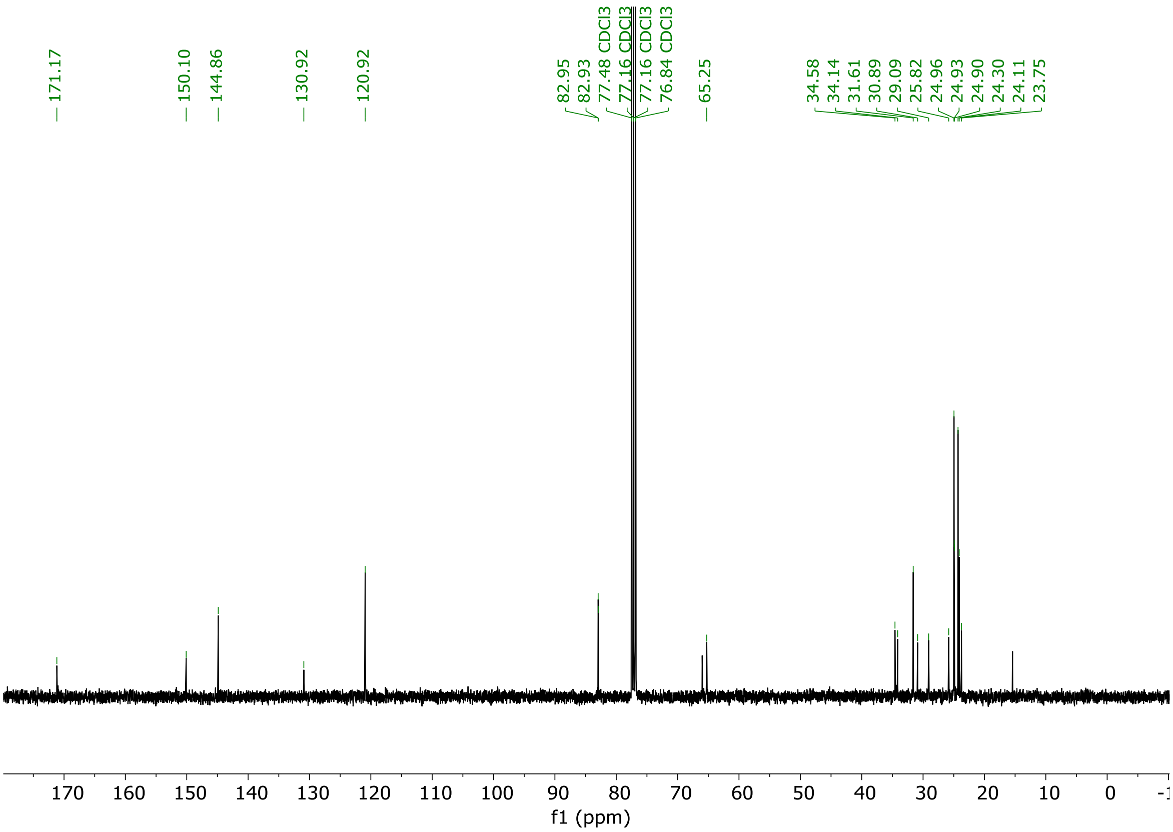

^{1}H NMR (400 MHz, $CDCl_3$) of compound **(3*S*-F1)**. See procedure.

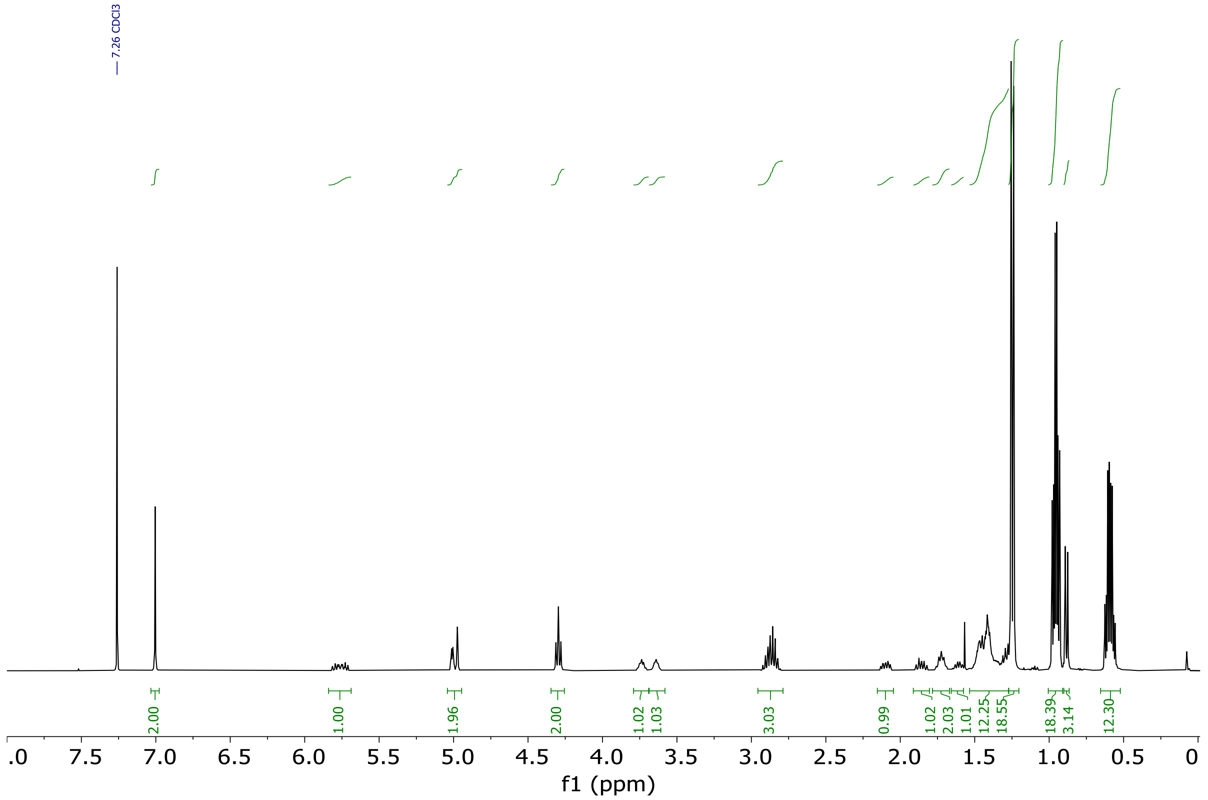

^{13}C NMR (101 MHz, $CDCl_3$) of compound **(3*S*-F1)**. See procedure.

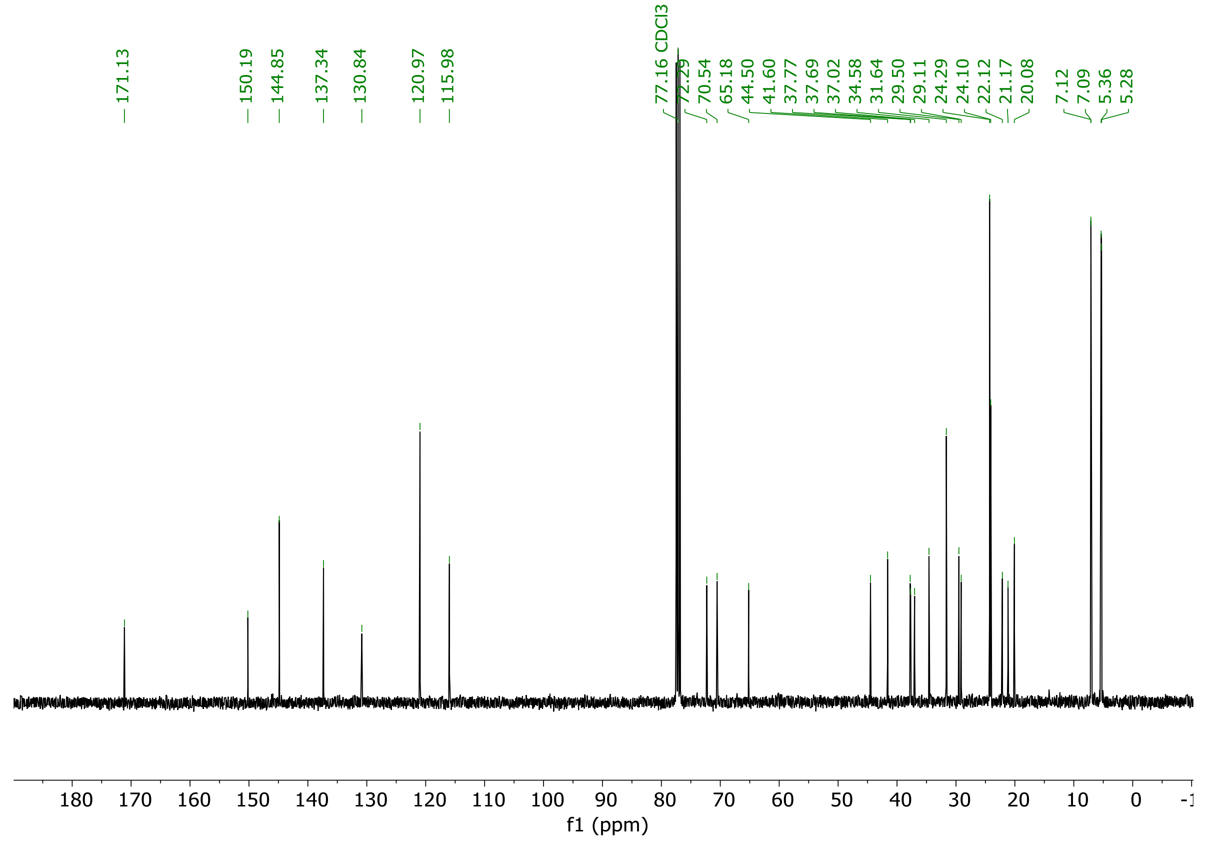

^{1}H NMR (500 MHz, $CDCl_3$) of **(3*R*-F1)**. See procedure.

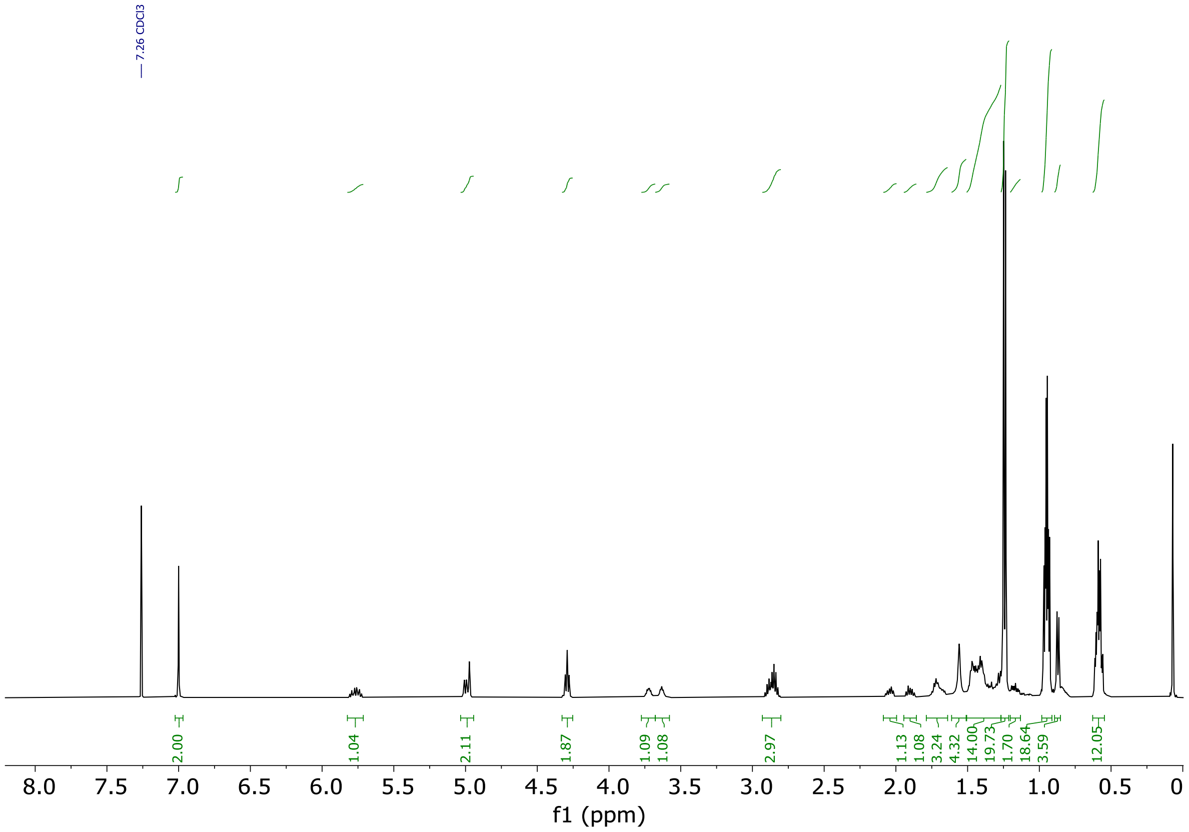

^{13}C NMR (126 MHz, $CDCl_3$) of compound **(3*R*-F1)**. See procedure.

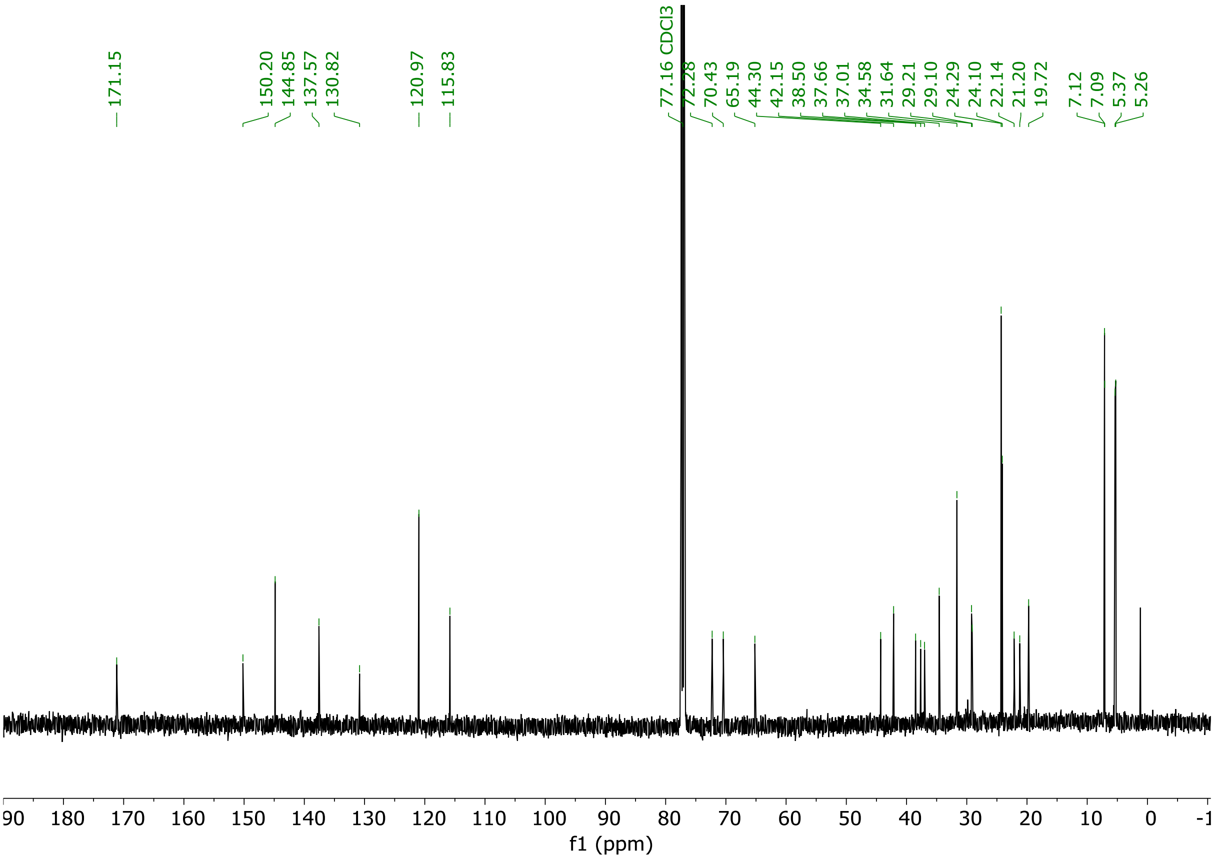

^{1}H NMR (400 MHz, $CDCl_3$) of compound **7**. *See procedure.*

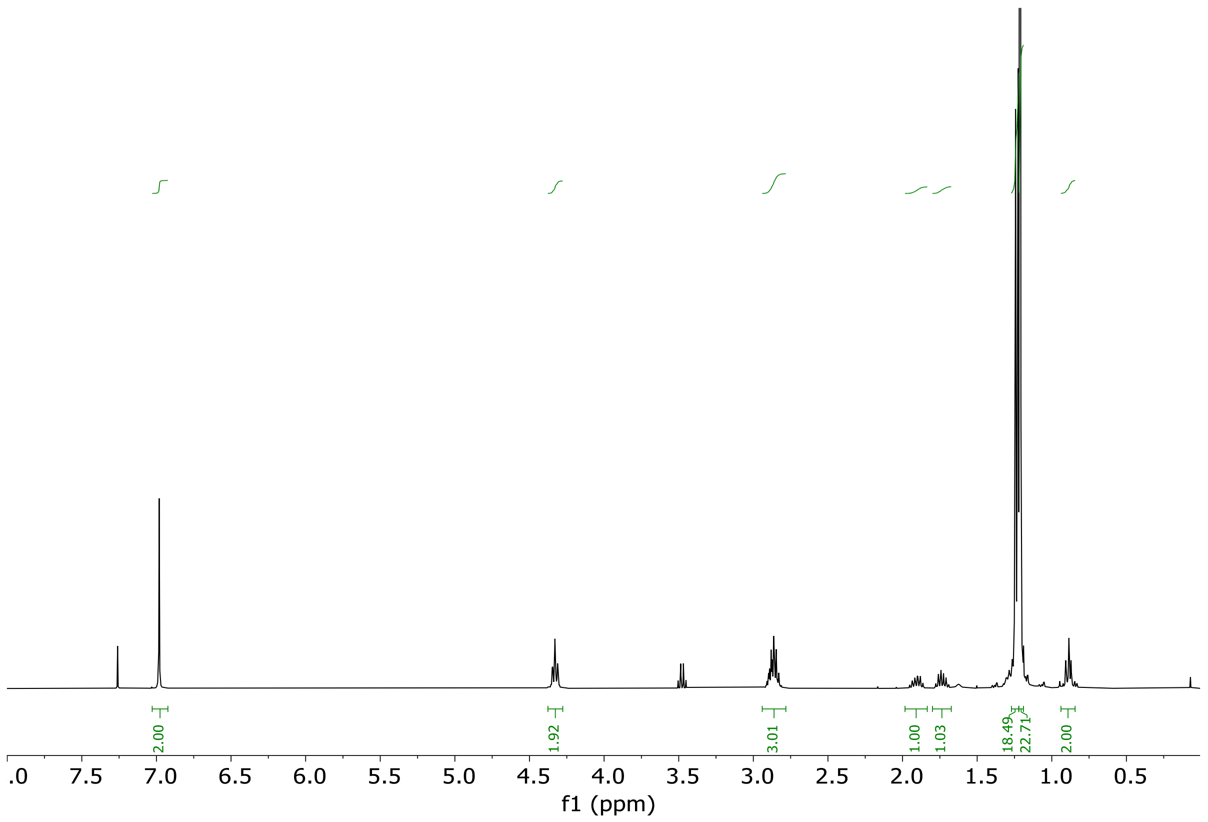

^{13}C NMR (101 MHz, $CDCl_3$) of compound **7**. *See procedure*.

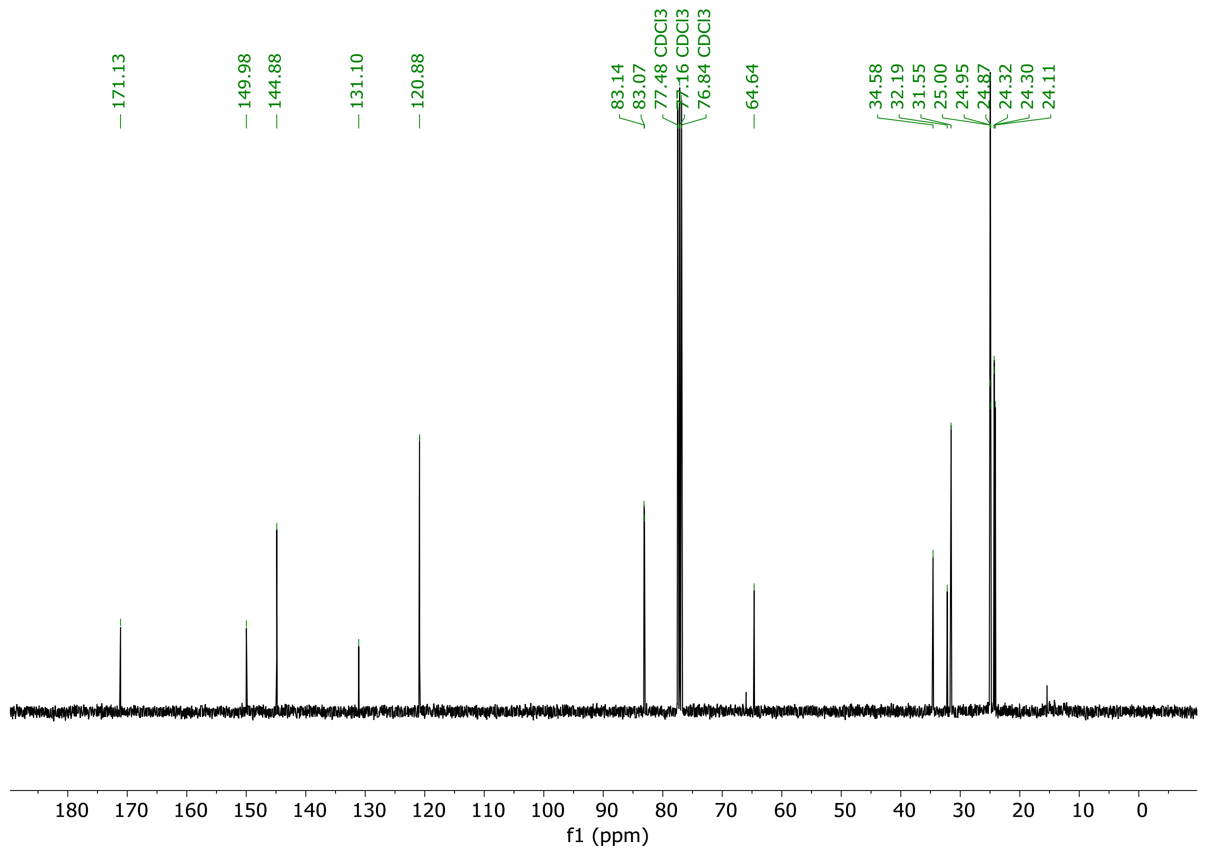

^{1}H NMR (400 MHz, $CDCl_3$) of compound **8**. See procedure.

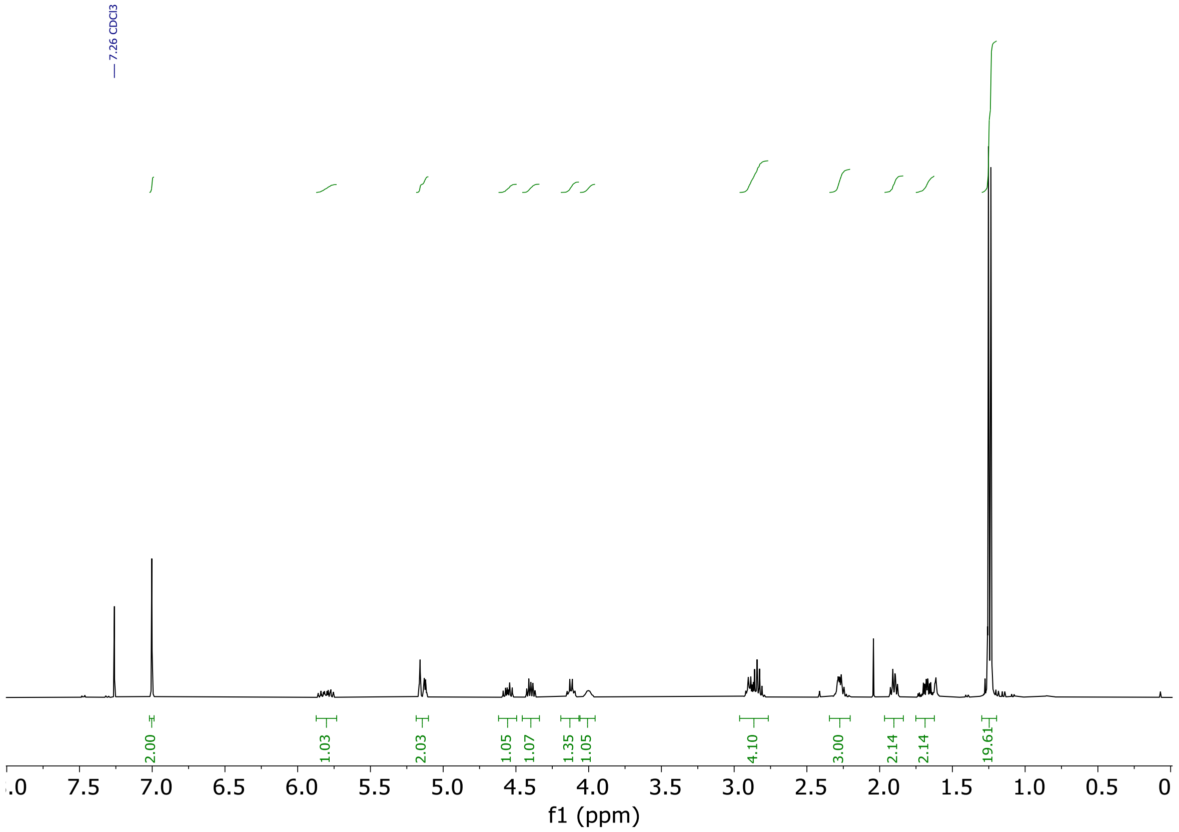

^{13}C NMR (101 MHz, $CDCl_3$) of compound **8**. See procedure.

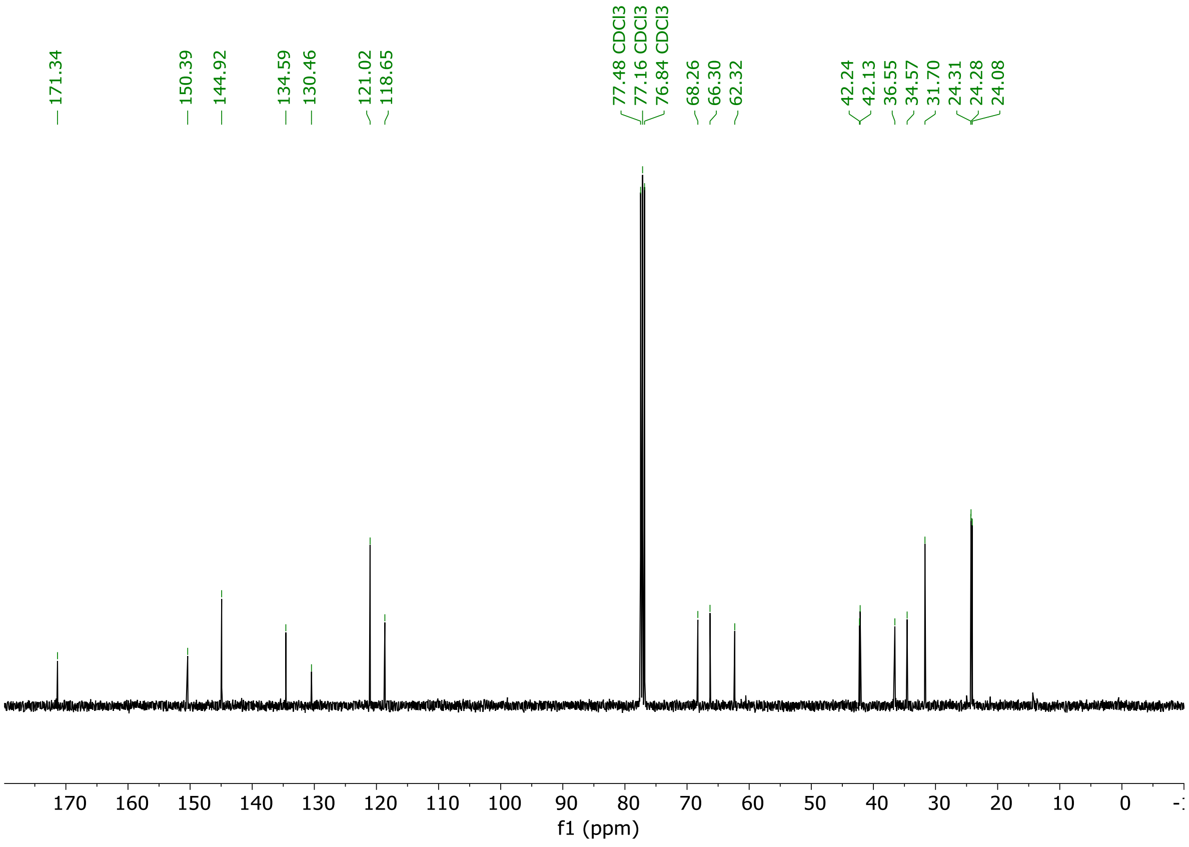

^{1}H NMR (400 MHz, $CDCl_3$) of compound **S9**. See procedure.

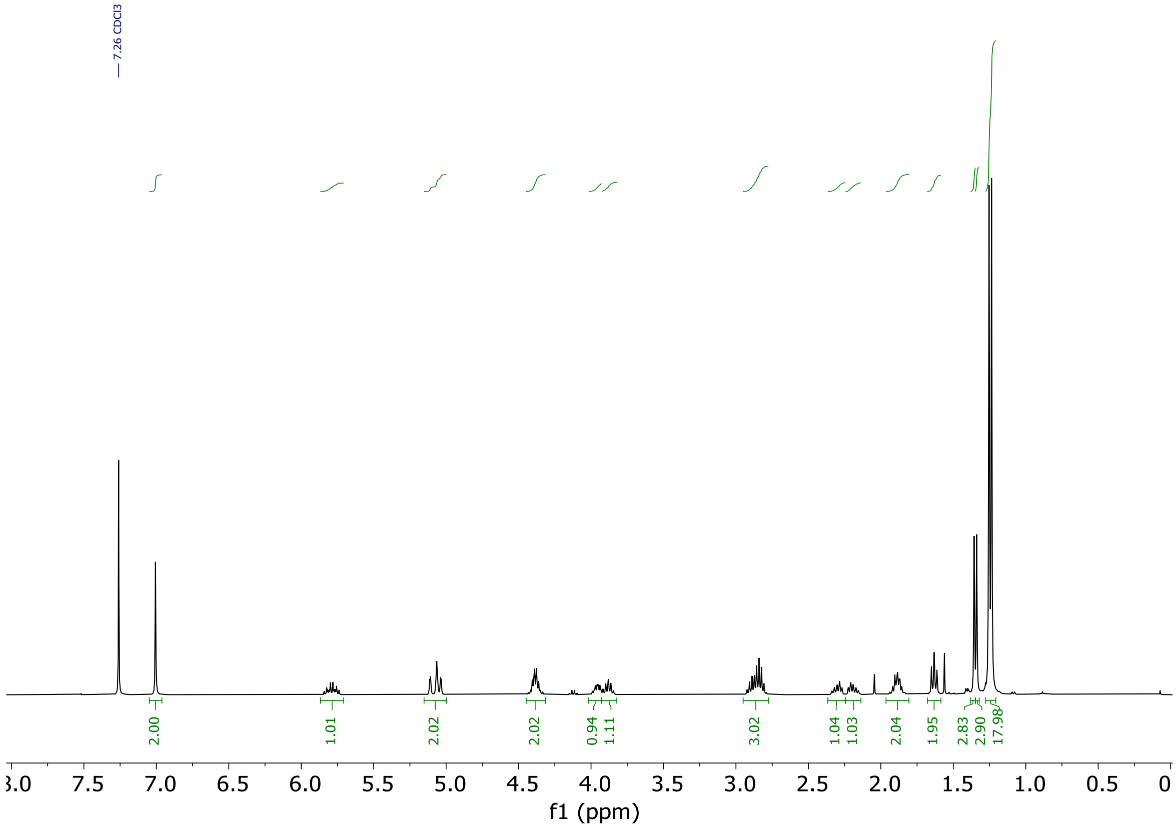

^{13}C NMR (101 MHz, $CDCl_3$) of compound **S9**. See procedure.

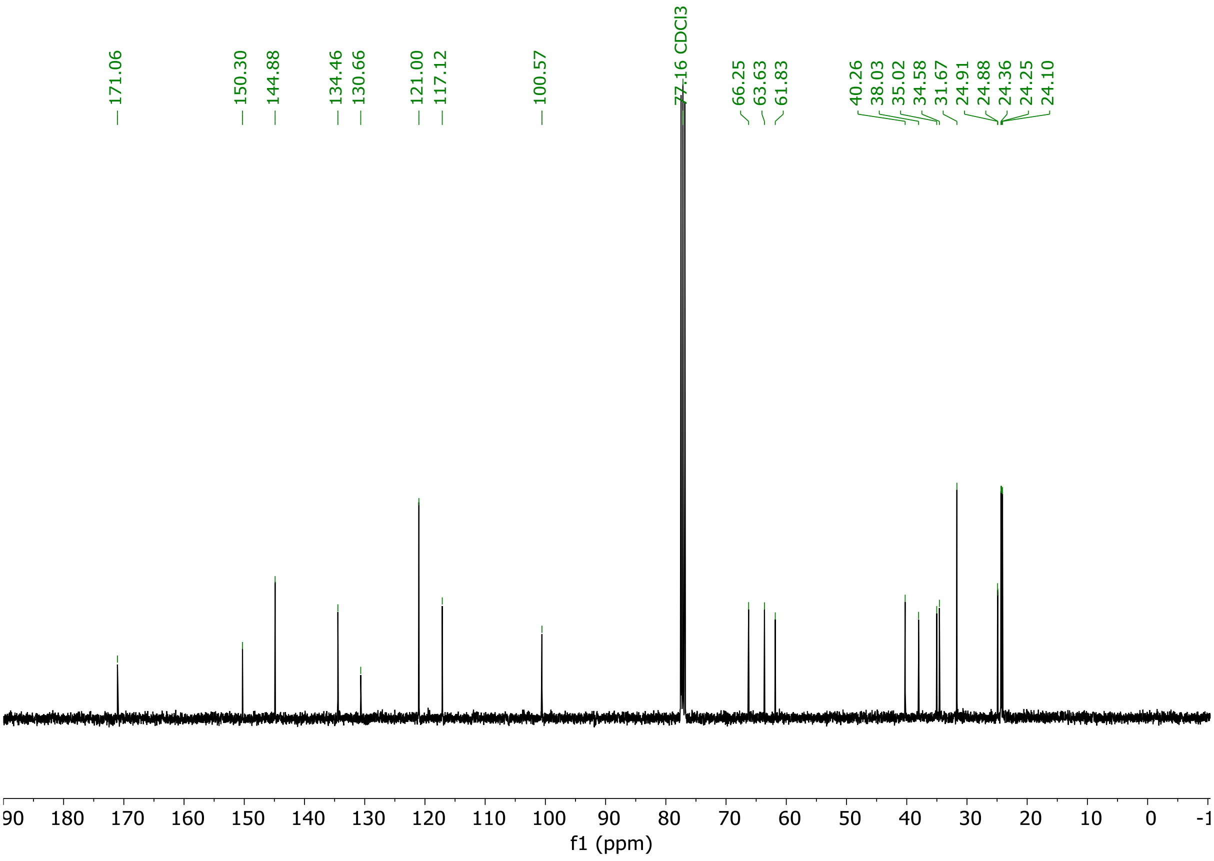

^{1}H NMR (400 MHz, $CDCl_3$) of **F2**. See procedure.

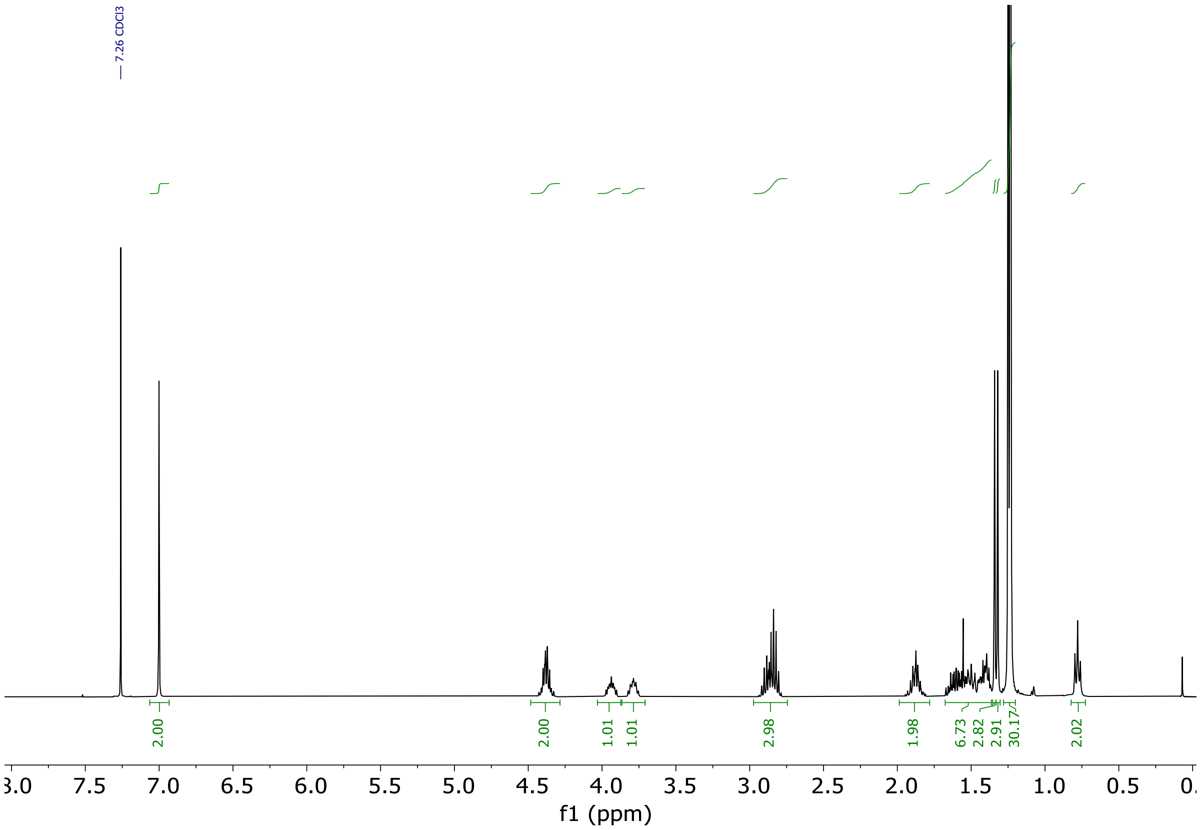

^{13}C NMR (101 MHz, $CDCl_3$) of **F2**. See procedure.

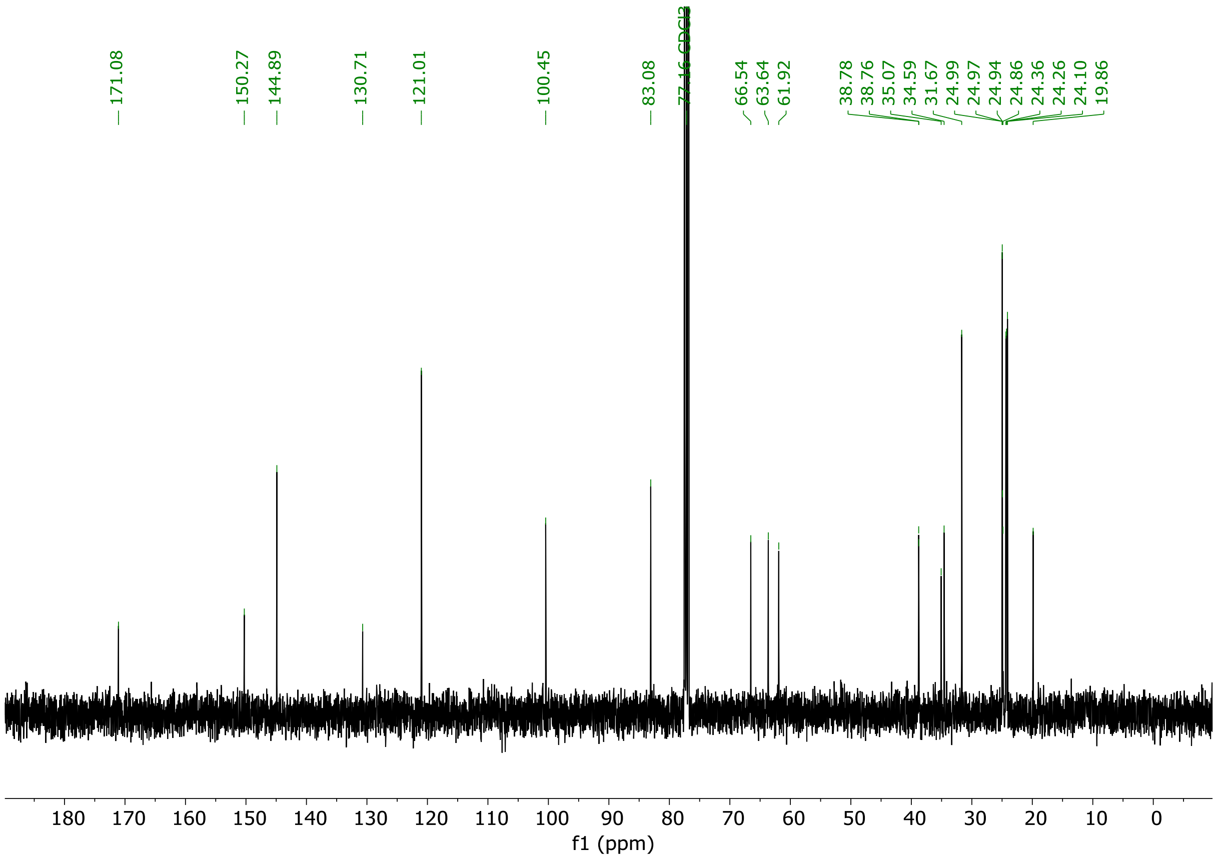

^{1}H NMR (400 MHz, $CDCl_3$) of compound **9**. *See procedure*.

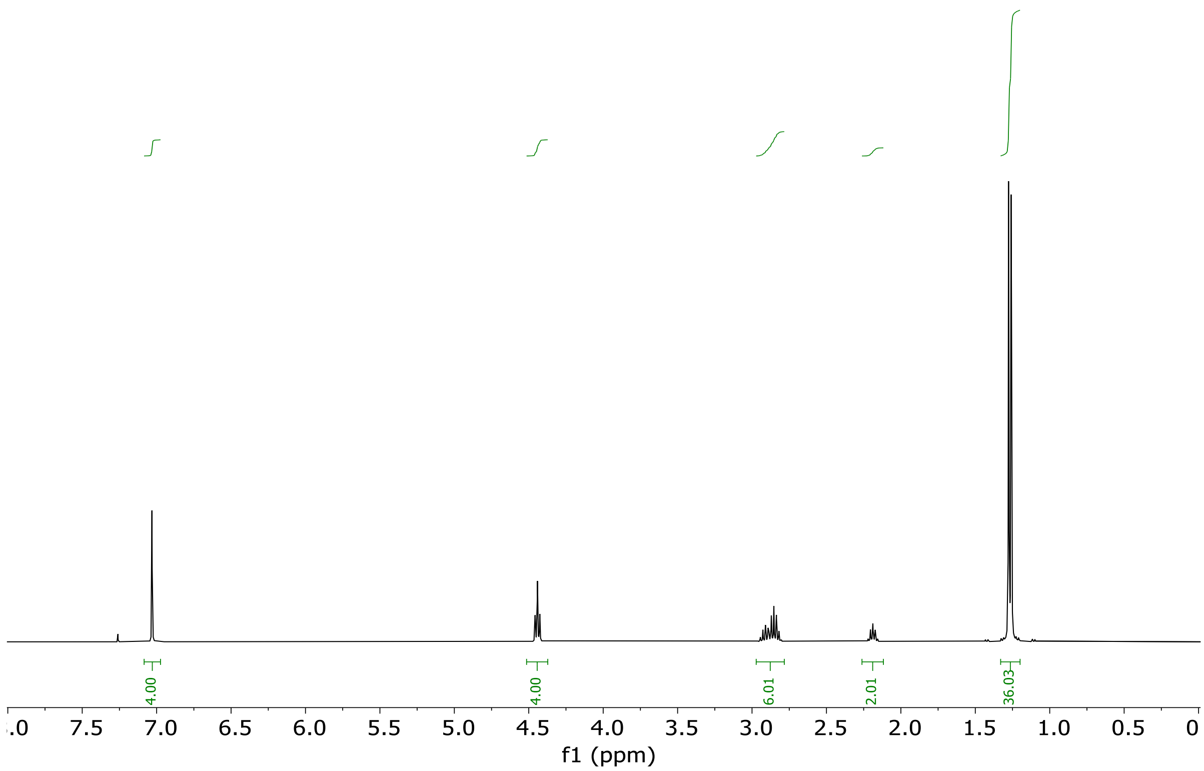

^{13}C NMR (101 MHz, $CDCl_3$) of compound **9**. *See procedure*.

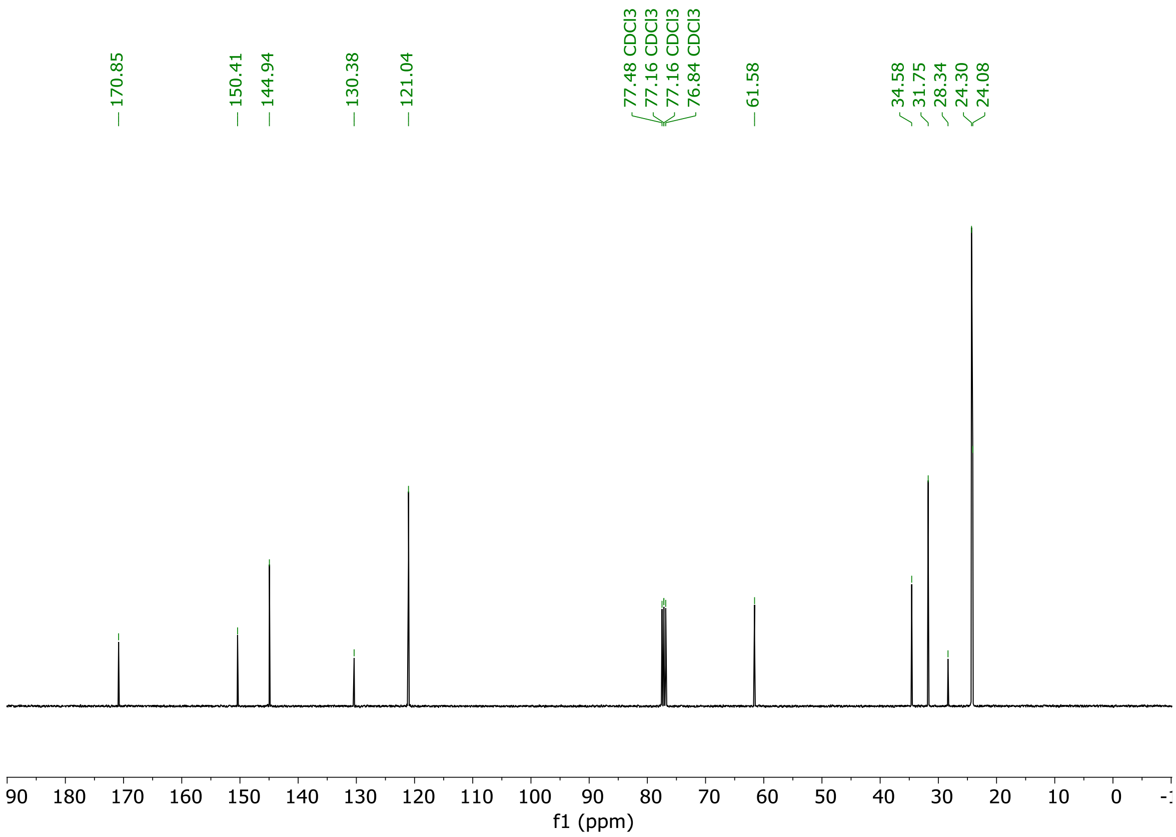

^{1}H NMR (400 MHz, $CDCl_3$) of compound **10-*anti***. See procedure

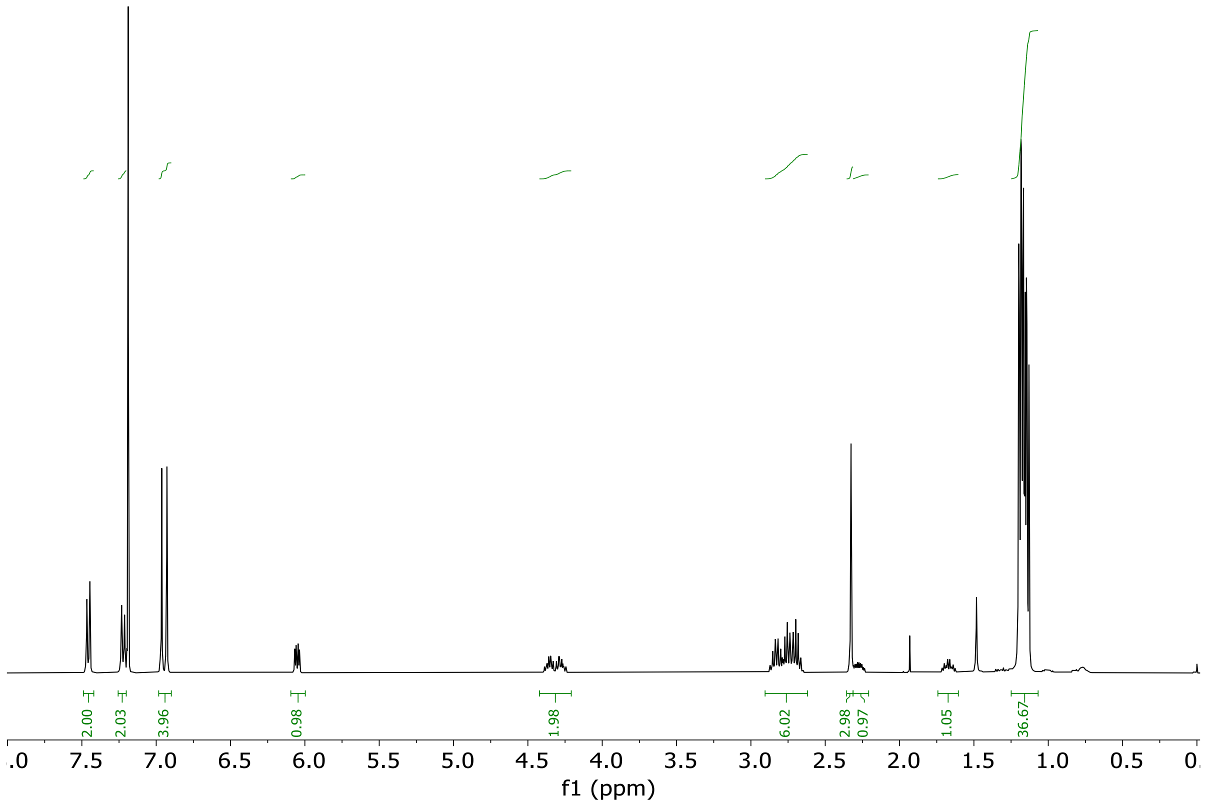

^{13}C NMR (101 MHz, $CDCl_3$) of compound **10-*anti***. See procedure

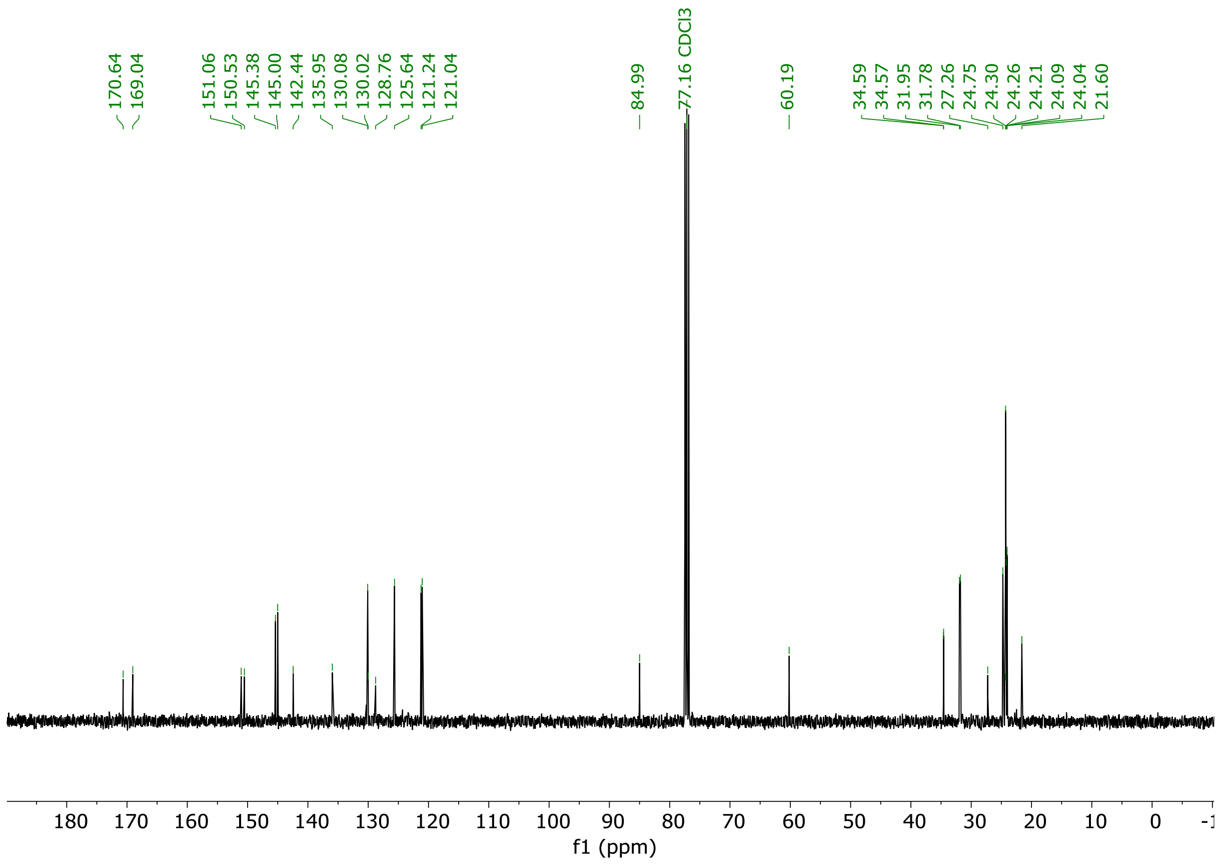

^{1}H NMR (400 MHz, $CDCl_3$) of compound **10-*syn***. See procedure

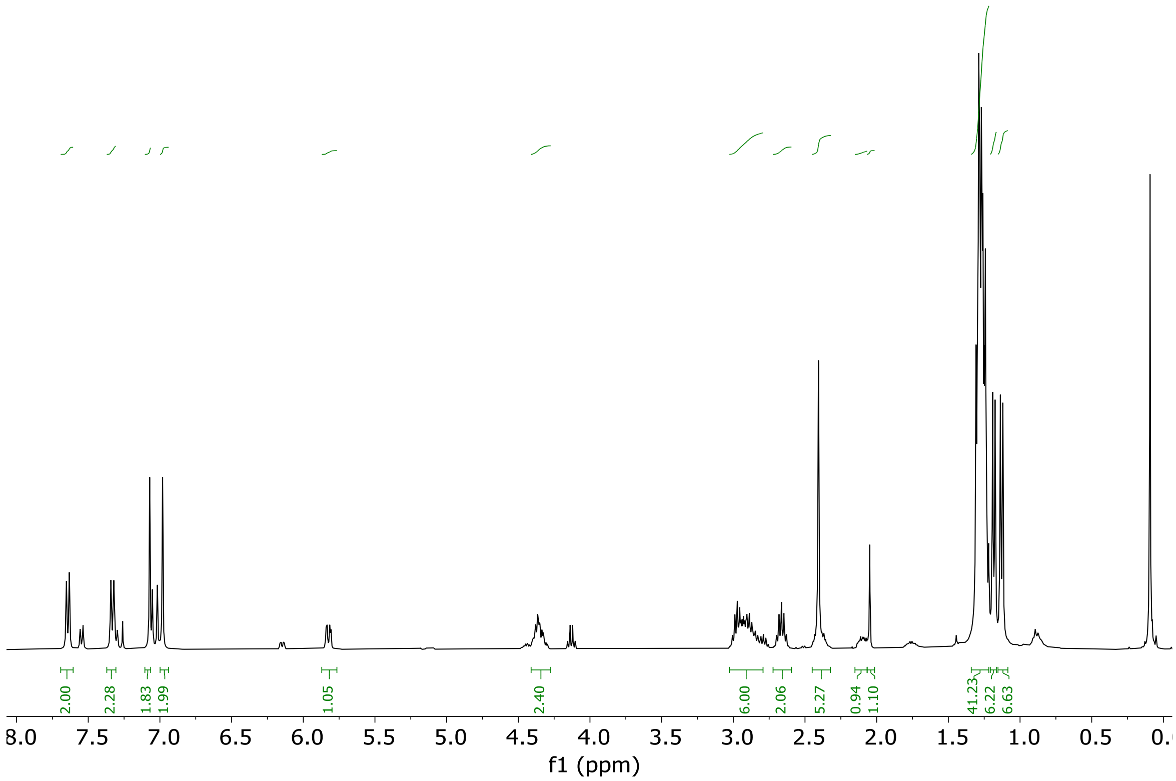

^{13}C NMR (101 MHz, $CDCl_3$) of compound **10-*syn***. See procedure

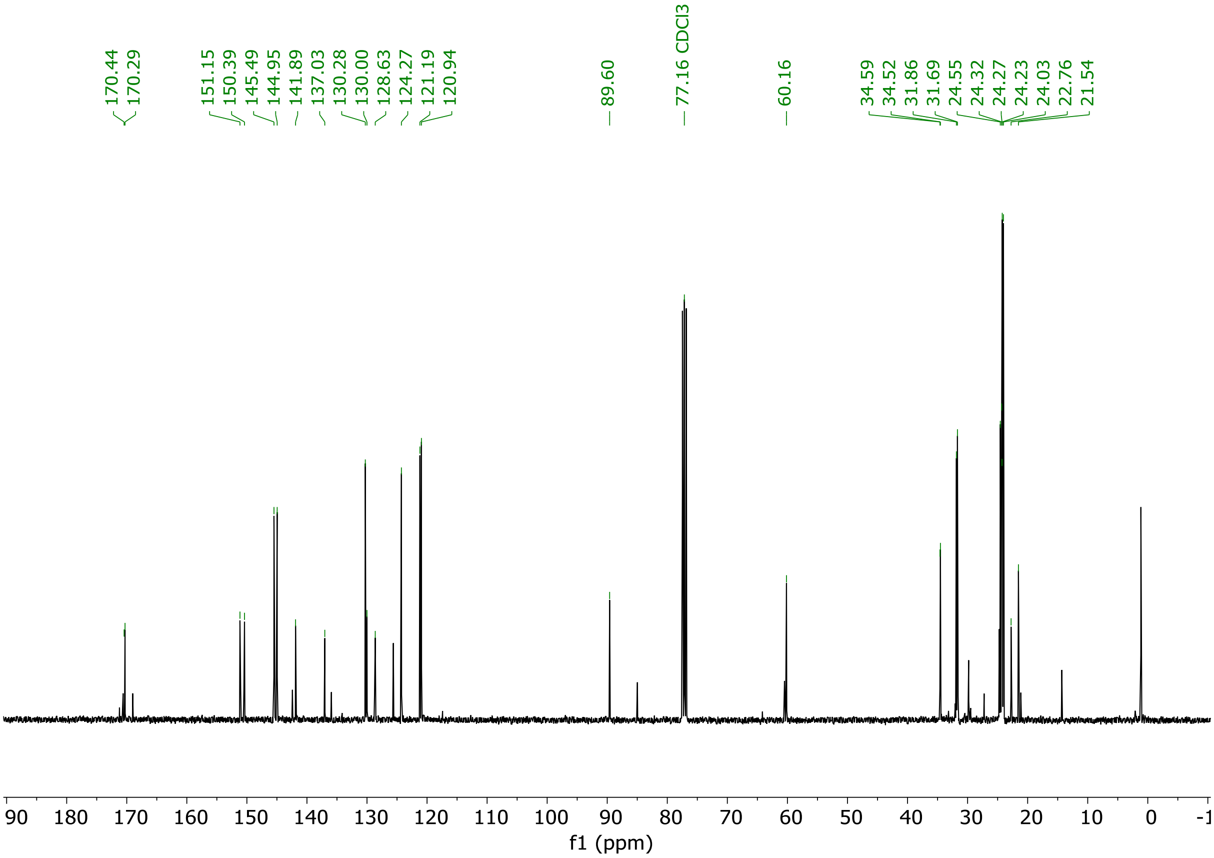

^{1}H NMR (400 MHz, $CDCl_3$) of compound **11** (with bis TIB ester **9** impurity). *See procedure*

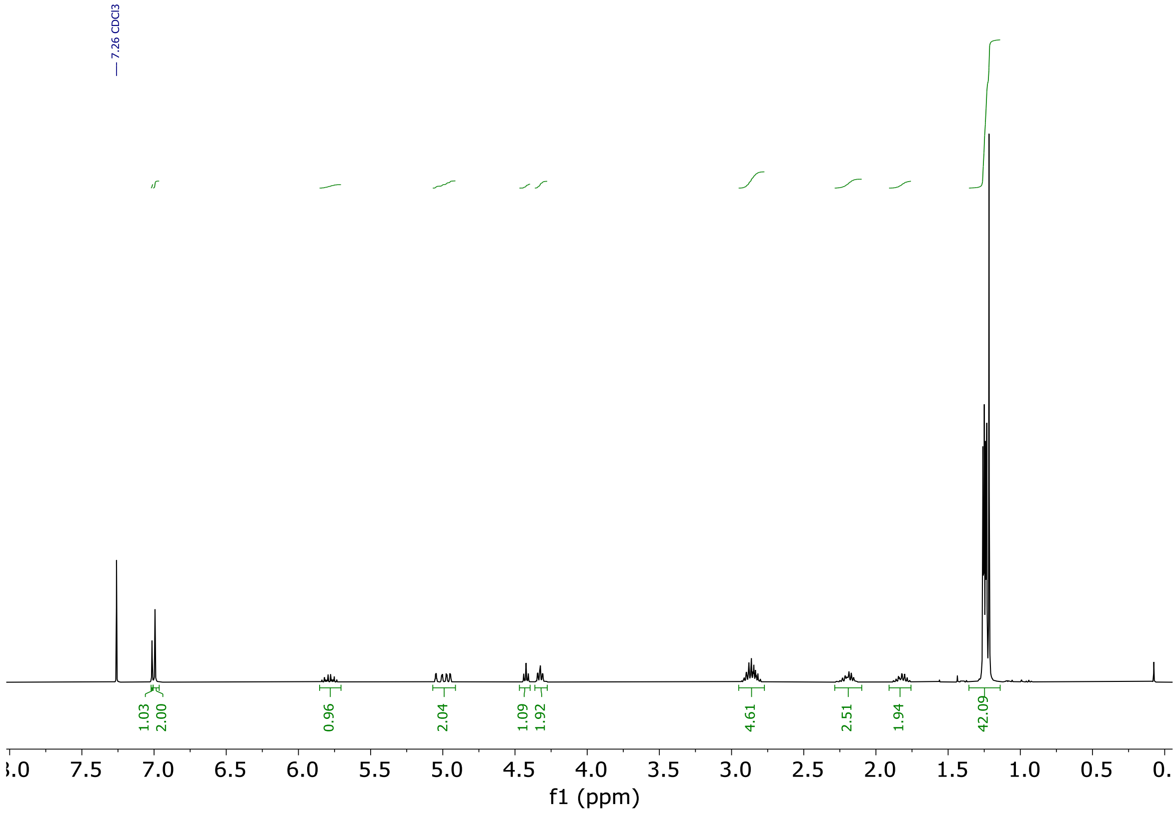

^{1}H NMR (400 MHz, $CDCl_3$) of compound **11** (repurified for analysis)

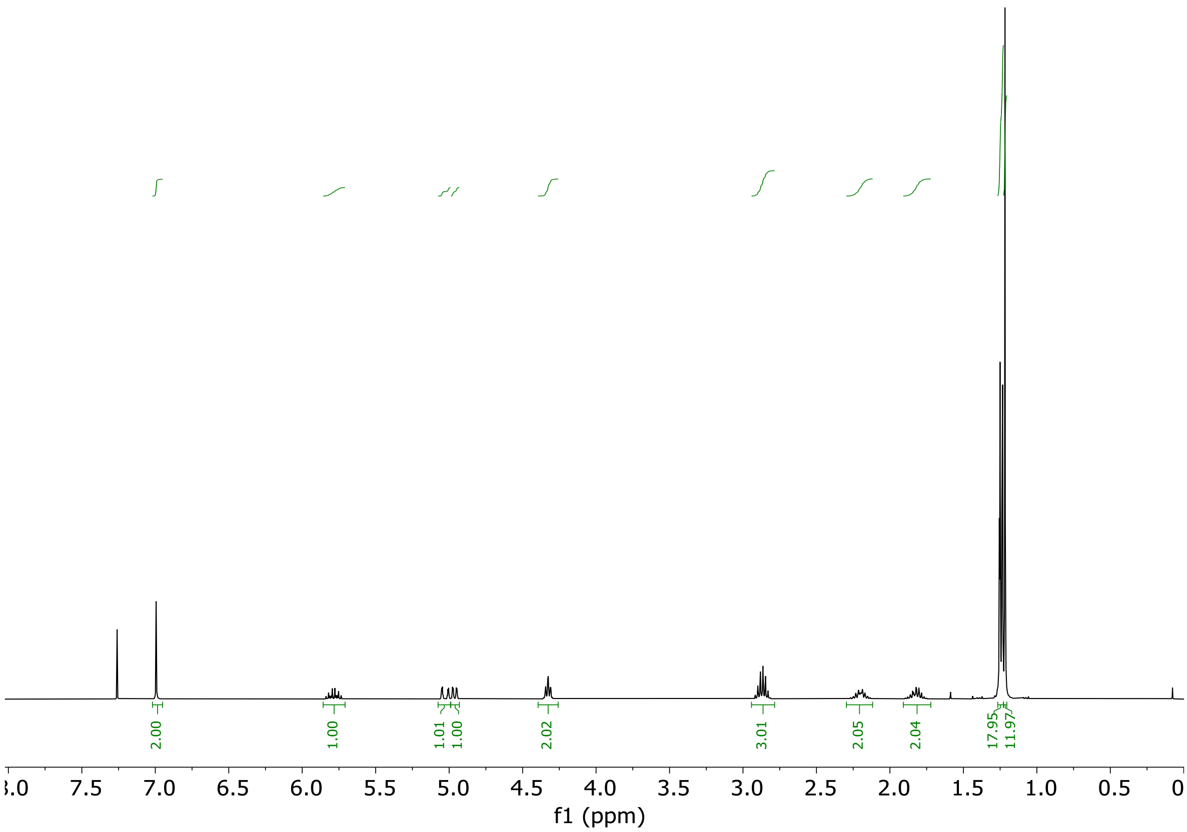

^{13}C NMR (101 MHz, $CDCl_3$) of compound **11** (with bis TIB ester **9** impurity). *See procedure*

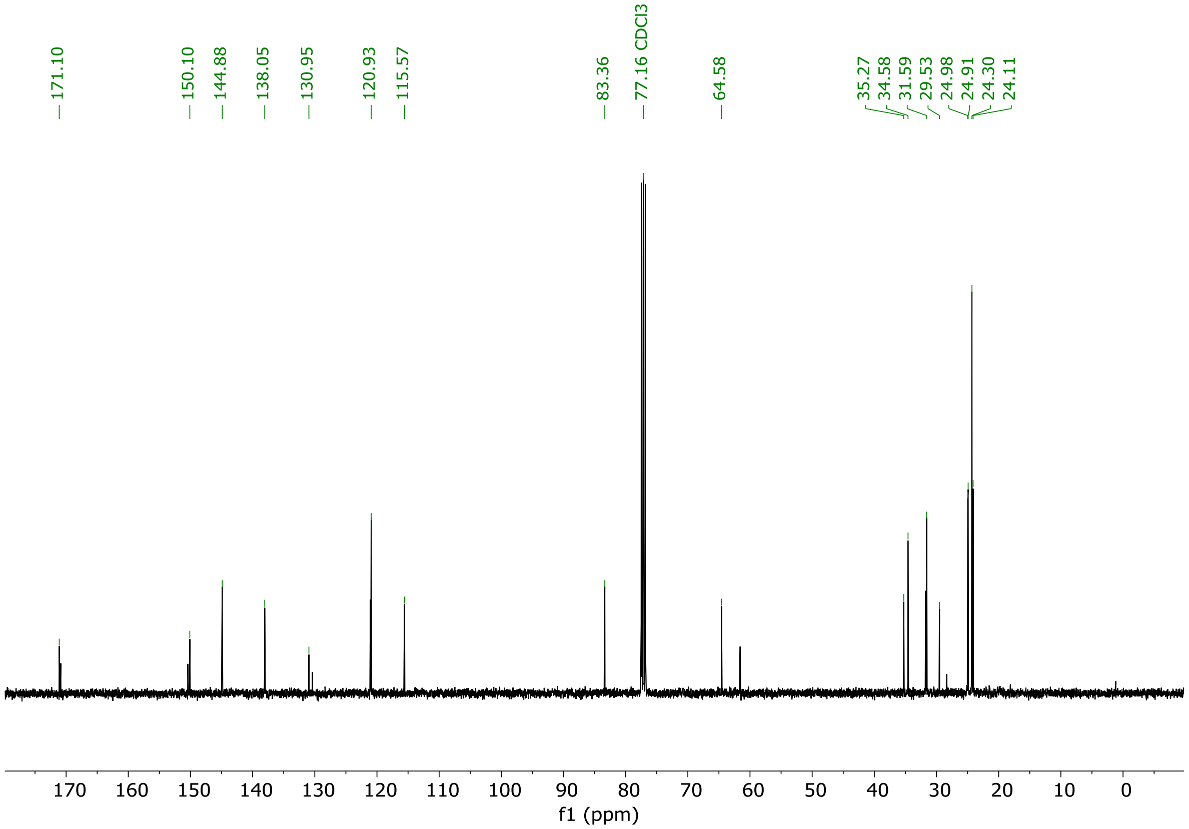

^{1}H NMR (400 MHz, $CDCl_3$) of compound **12**. *See procedure*.

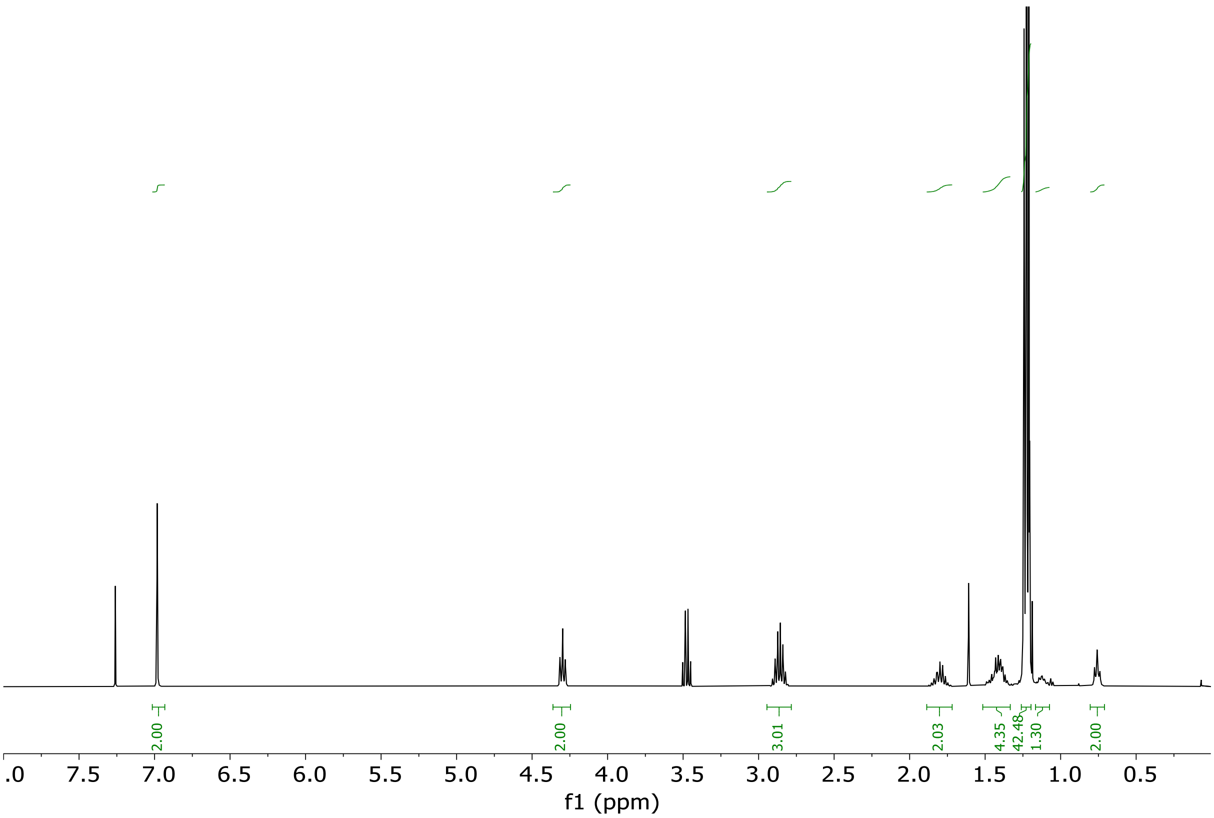

^{13}C NMR (101 MHz, $CDCl_3$) of compound **12**. *See procedure*.

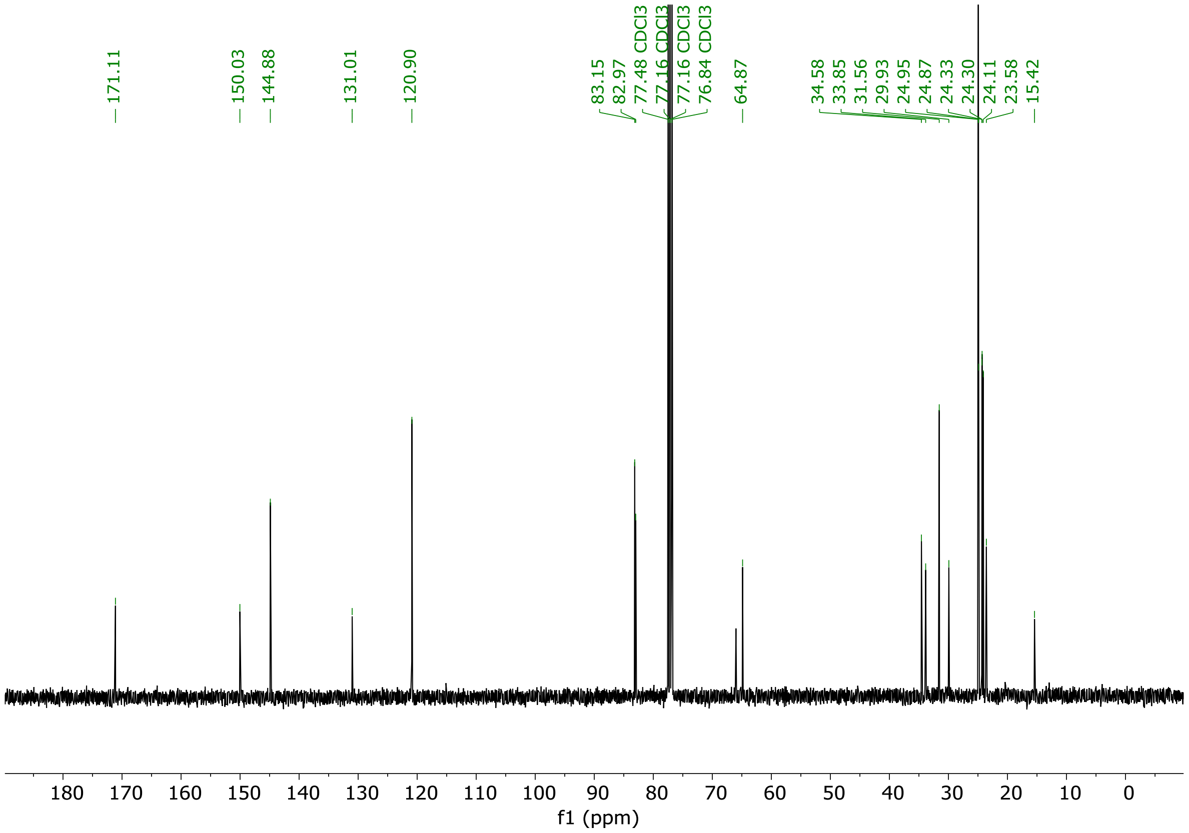

^{1}H NMR (400 MHz, $CDCl_3$) of compound **13**. *See procedure*.

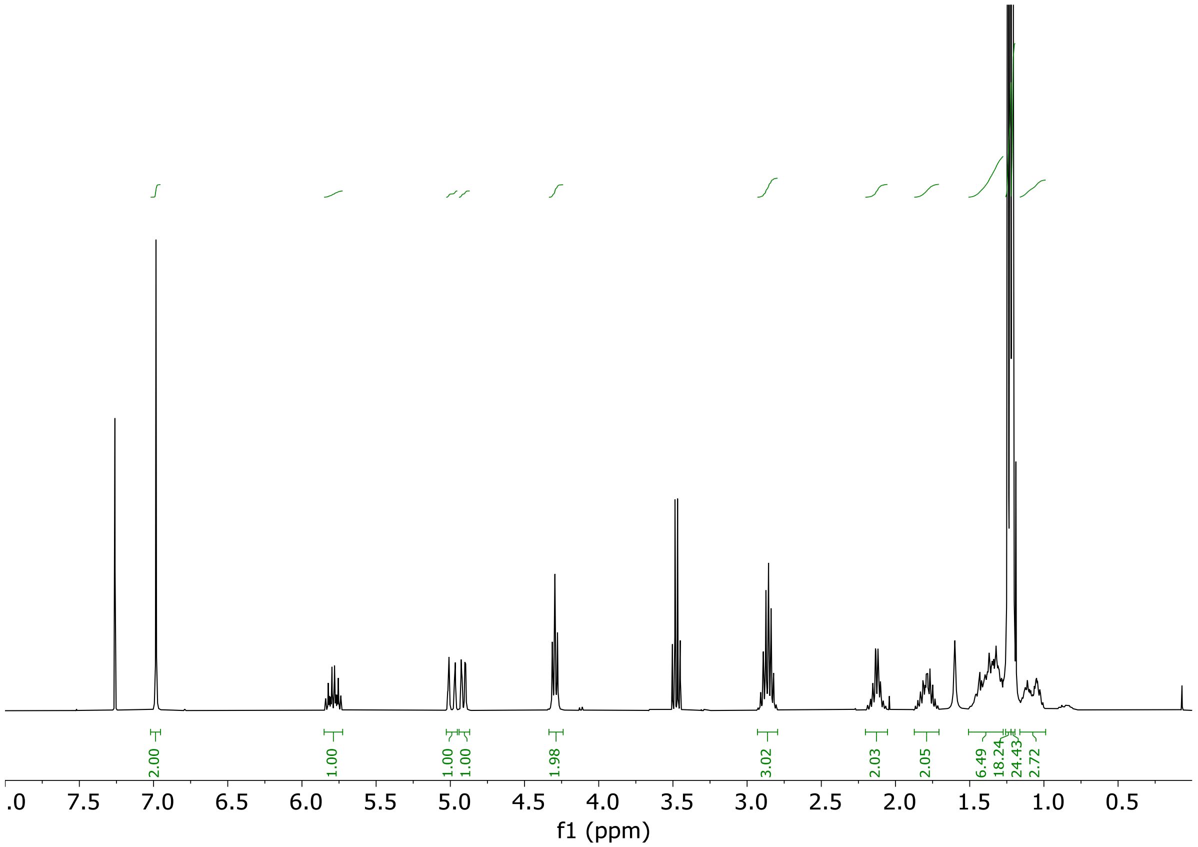

^{13}C NMR (101 MHz, $CDCl_3$) of compound **13**. See procedure.

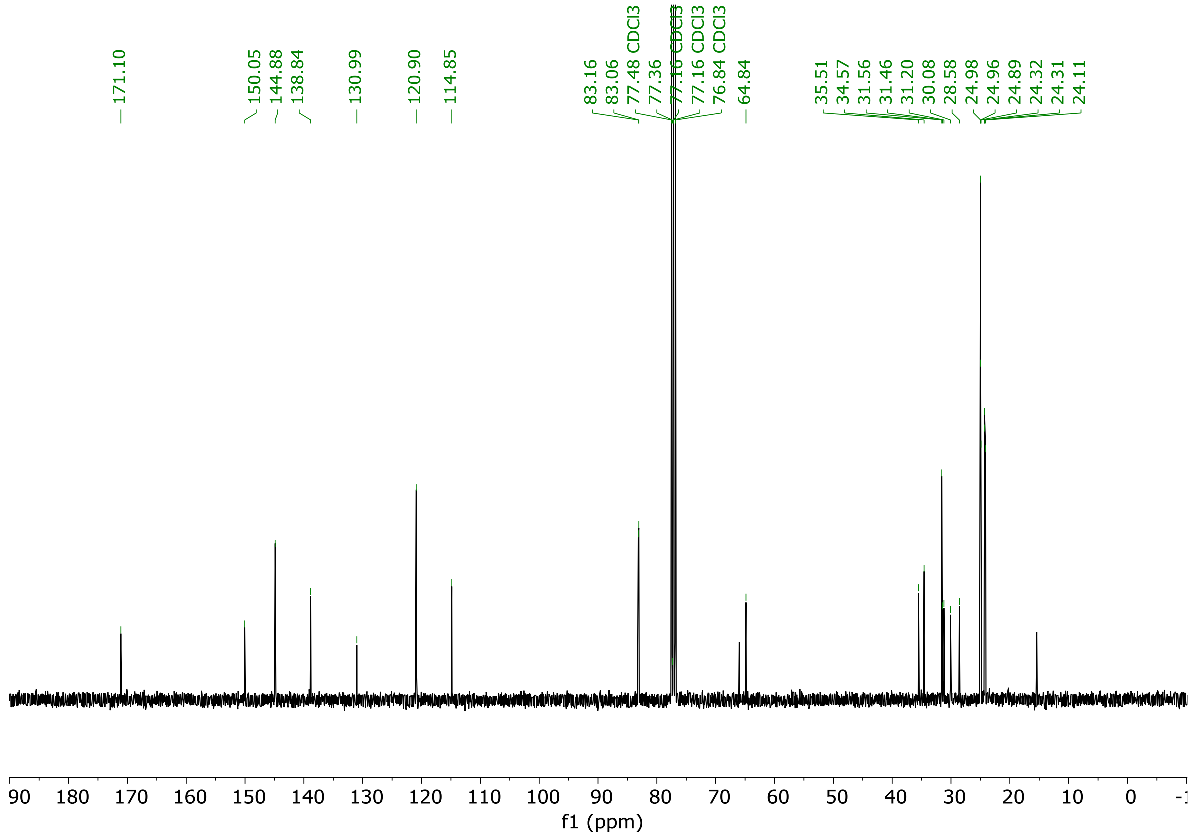

^{1}H NMR (400 MHz, $CDCl_3$) of compound **14**. See procedure.

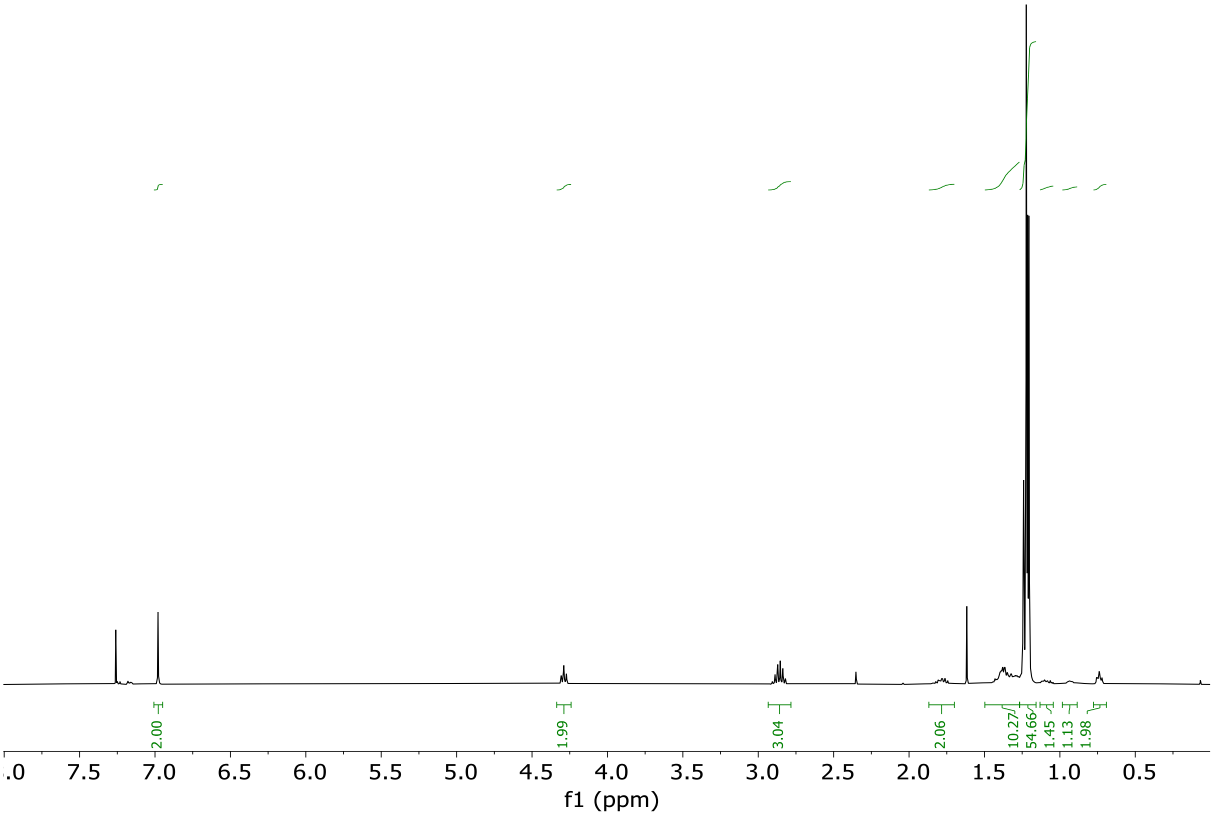

^{13}C NMR (101 MHz, $CDCl_3$) of compound **14**. See procedure.

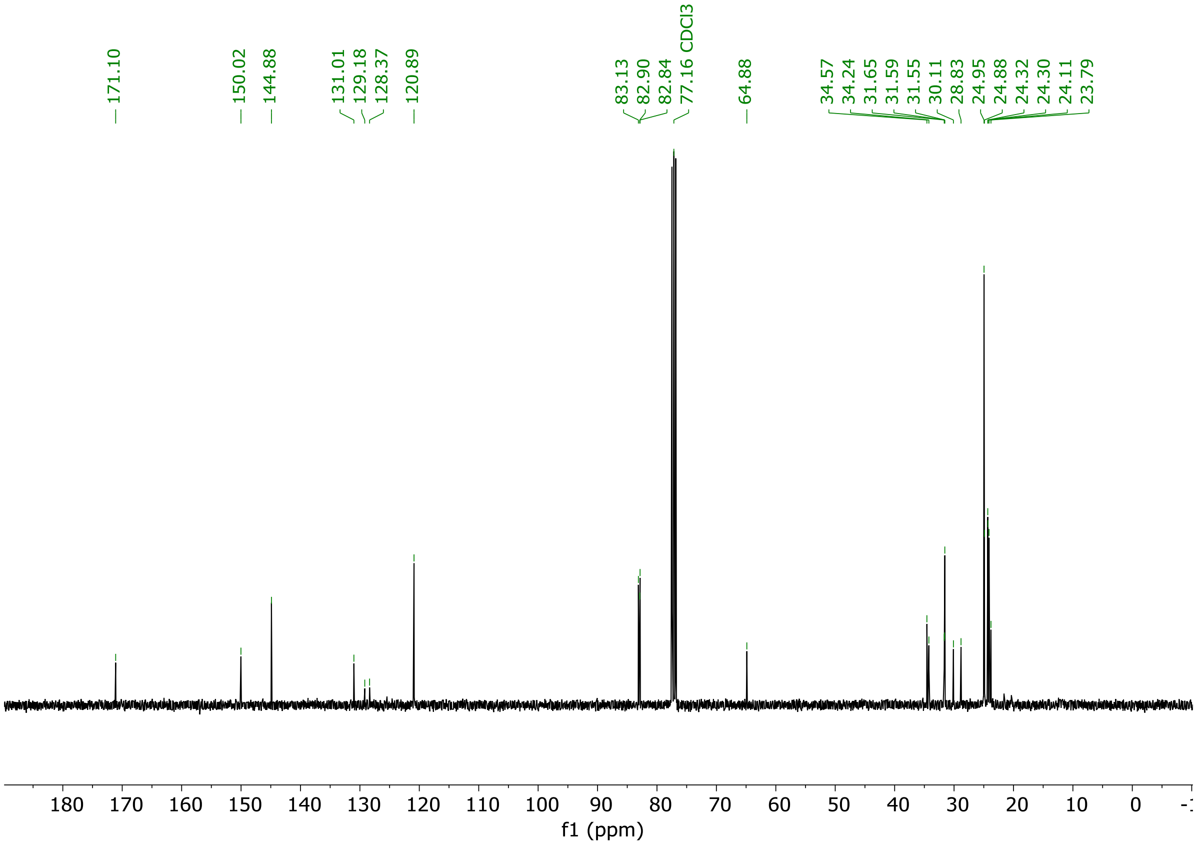

^{1}H NMR (400 MHz, $CDCl_3$) of compound **15**. See procedure.

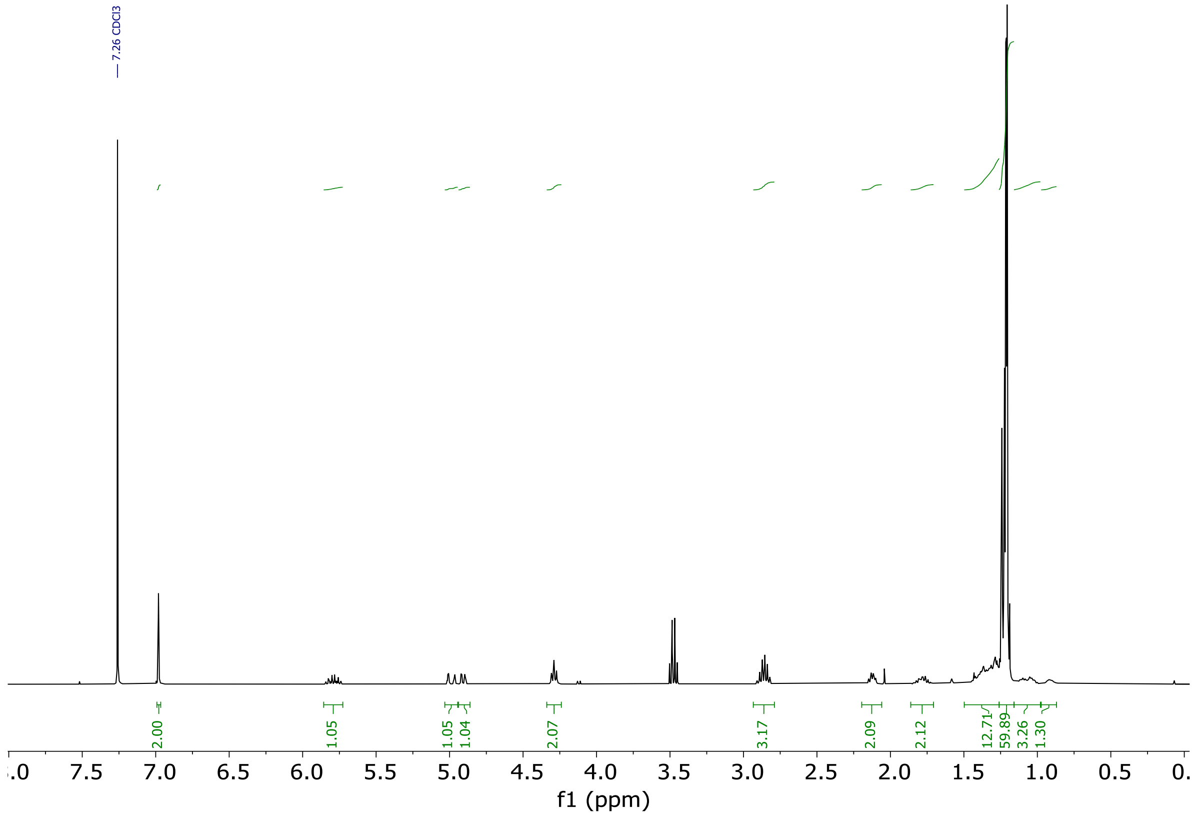

^{13}C NMR (101 MHz, $CDCl_3$) of compound **15**. *See procedure*

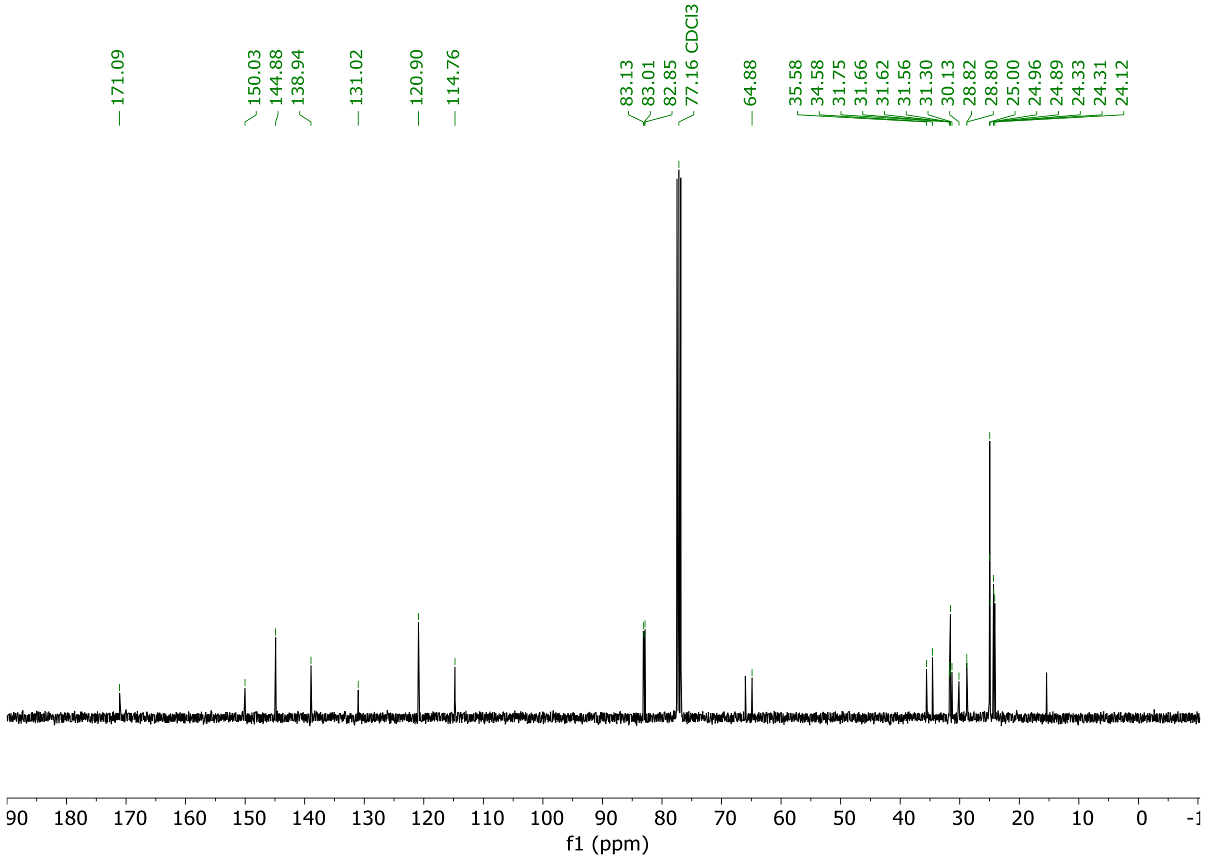

^{1}H NMR (400 MHz, $CDCl_3$) of compound **F3.** *See procedure*.

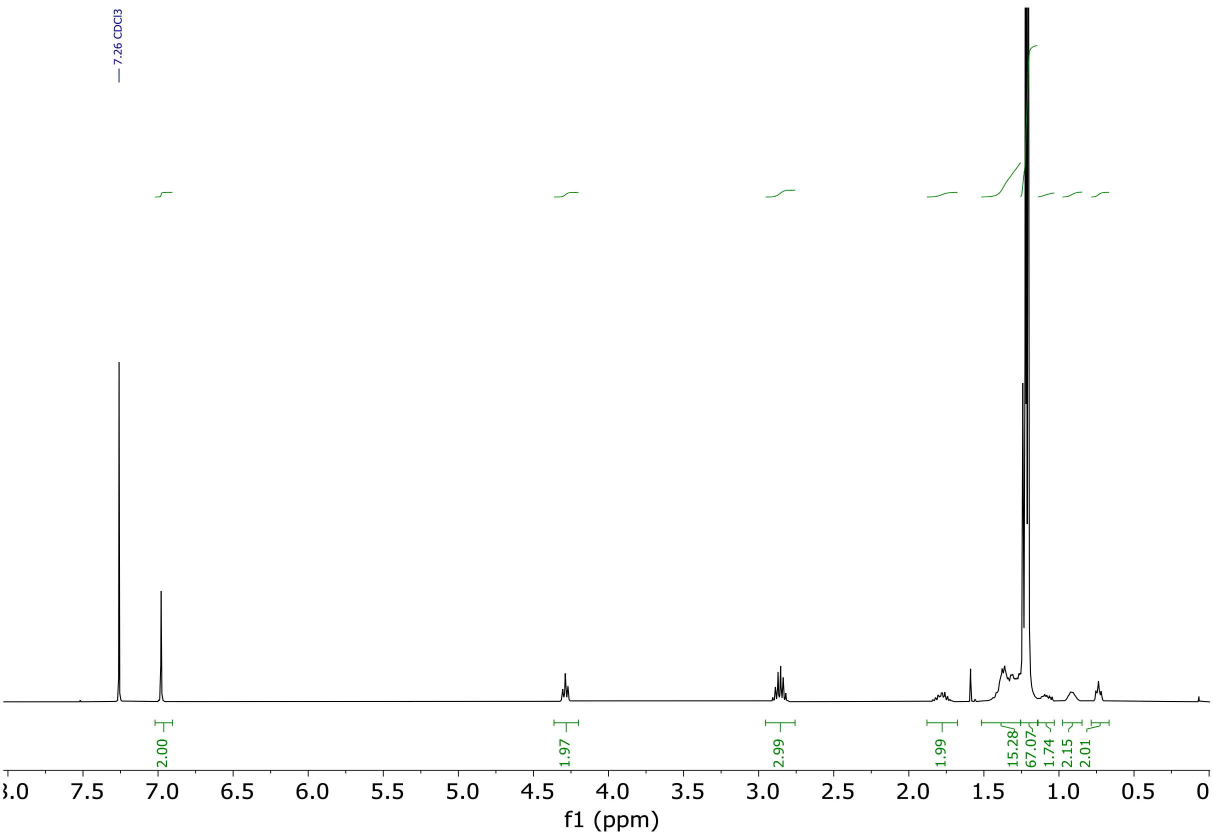

^{13}C NMR (101 MHz, $CDCl_3$) of compound **F3**. *See procedure*.

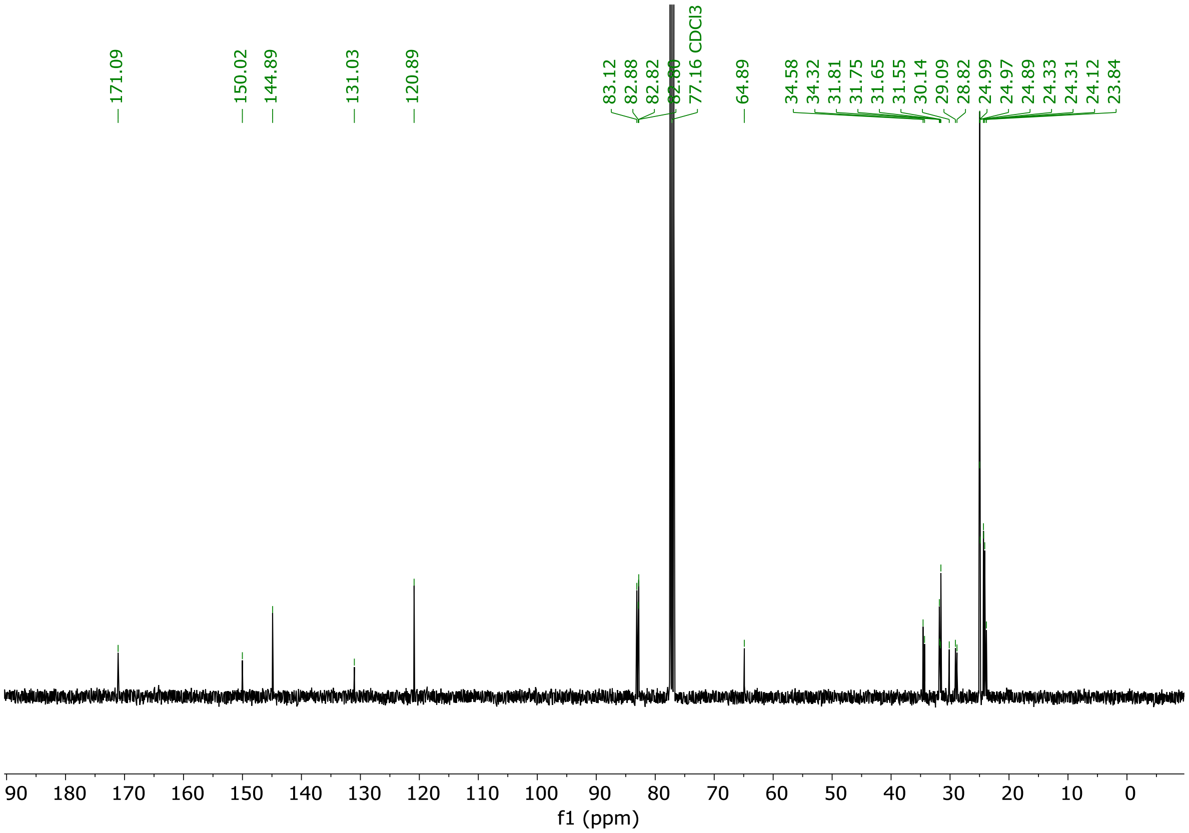

^{1}H NMR (400 MHz, $CDCl_3$) of compound **S10.** *See procedure*.

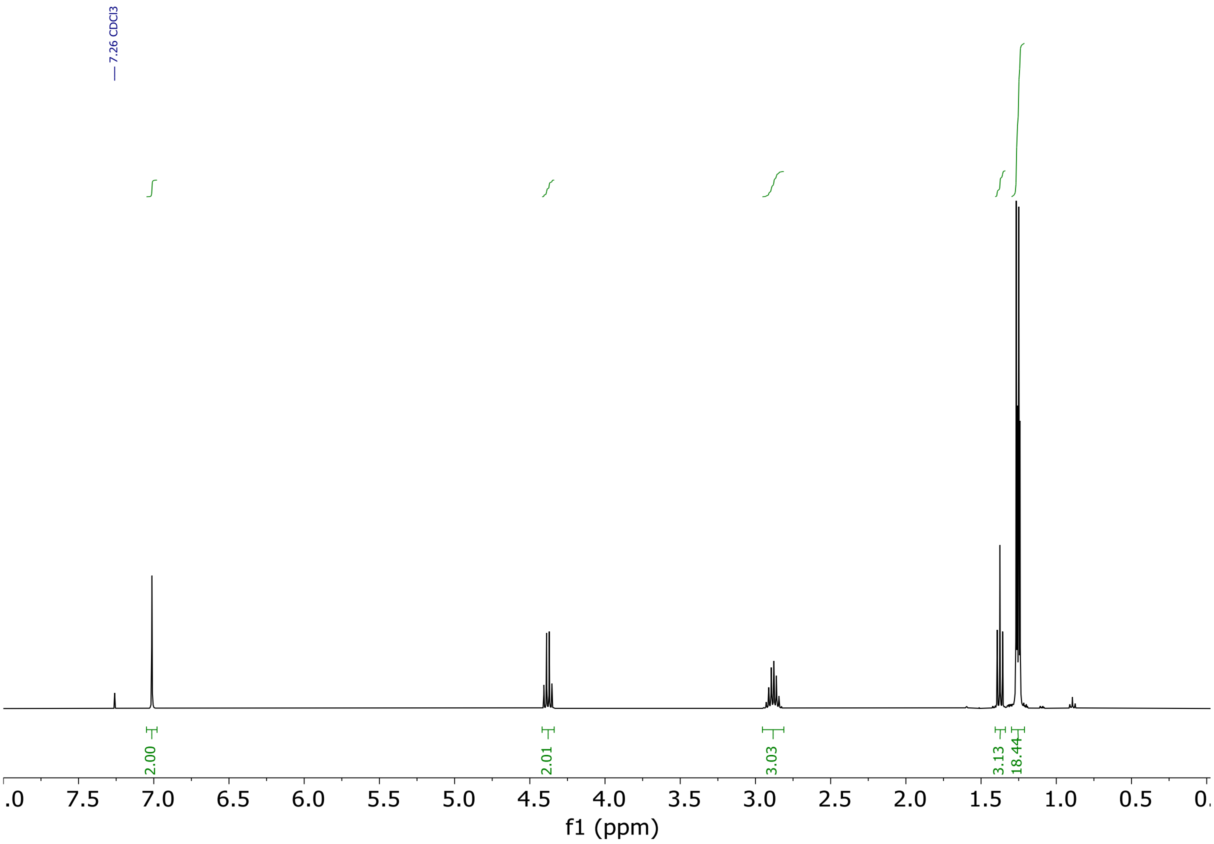

^{13}C NMR (101 MHz, $CDCl_3$) of compound **S10**. *See procedure*.

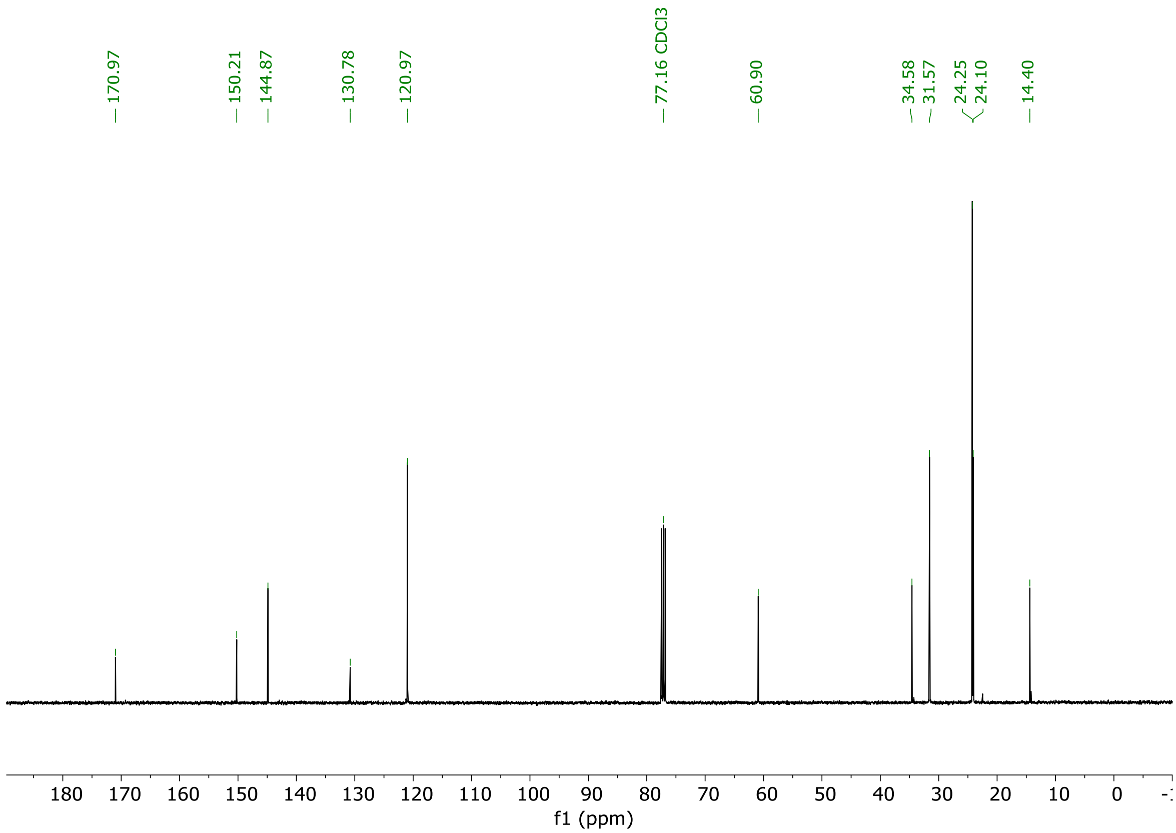

^{1}H NMR (400 MHz, $CDCl_3$) of compound **(*S*)-16.** *See procedure*.

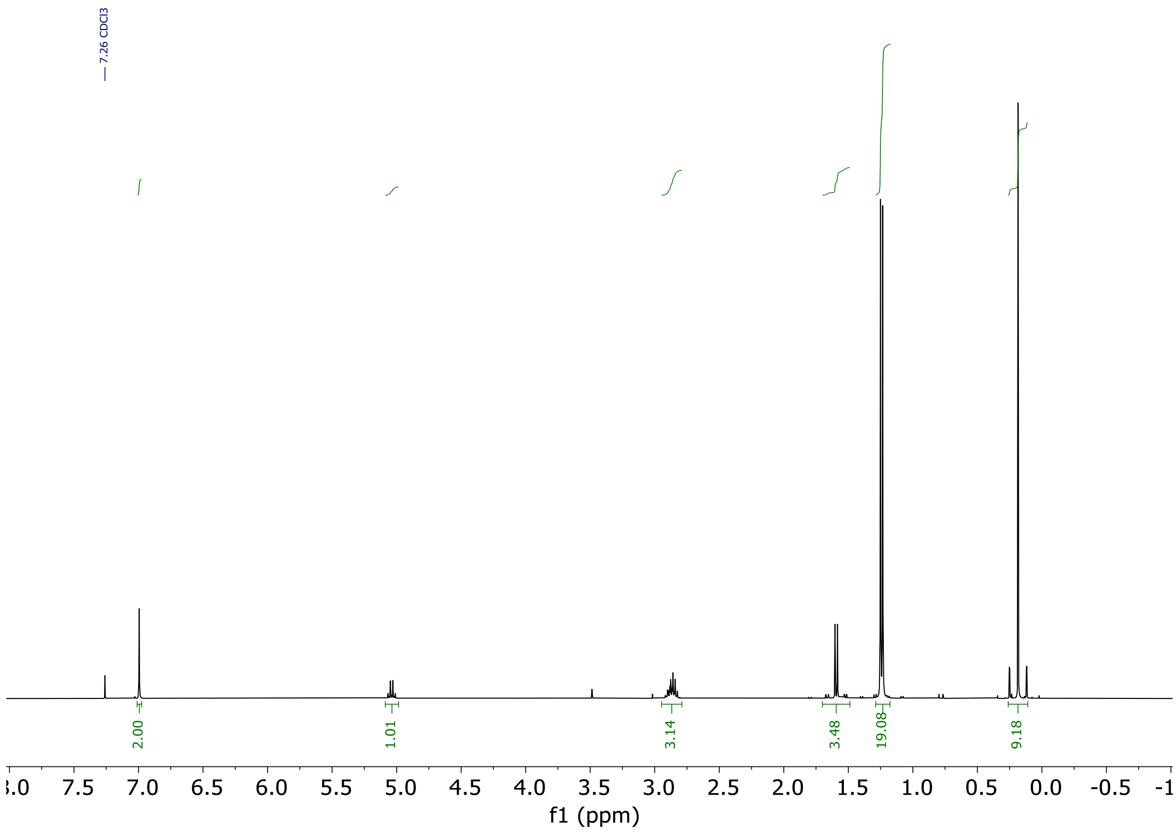

^{13}C NMR (101 MHz, $CDCl_3$) of compound **(*S*)-16**. See procedure.

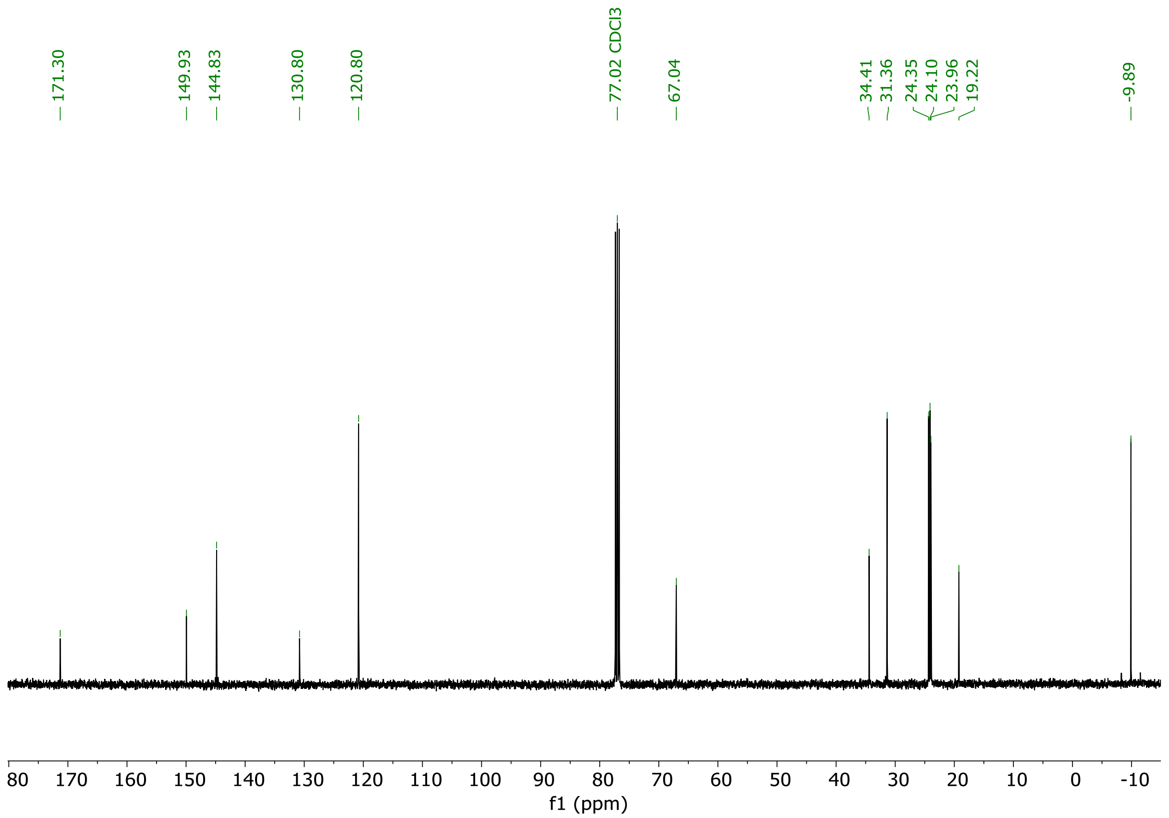

^{1}H NMR (400 MHz, $CDCl_3$) of compound **F4.** See procedure.

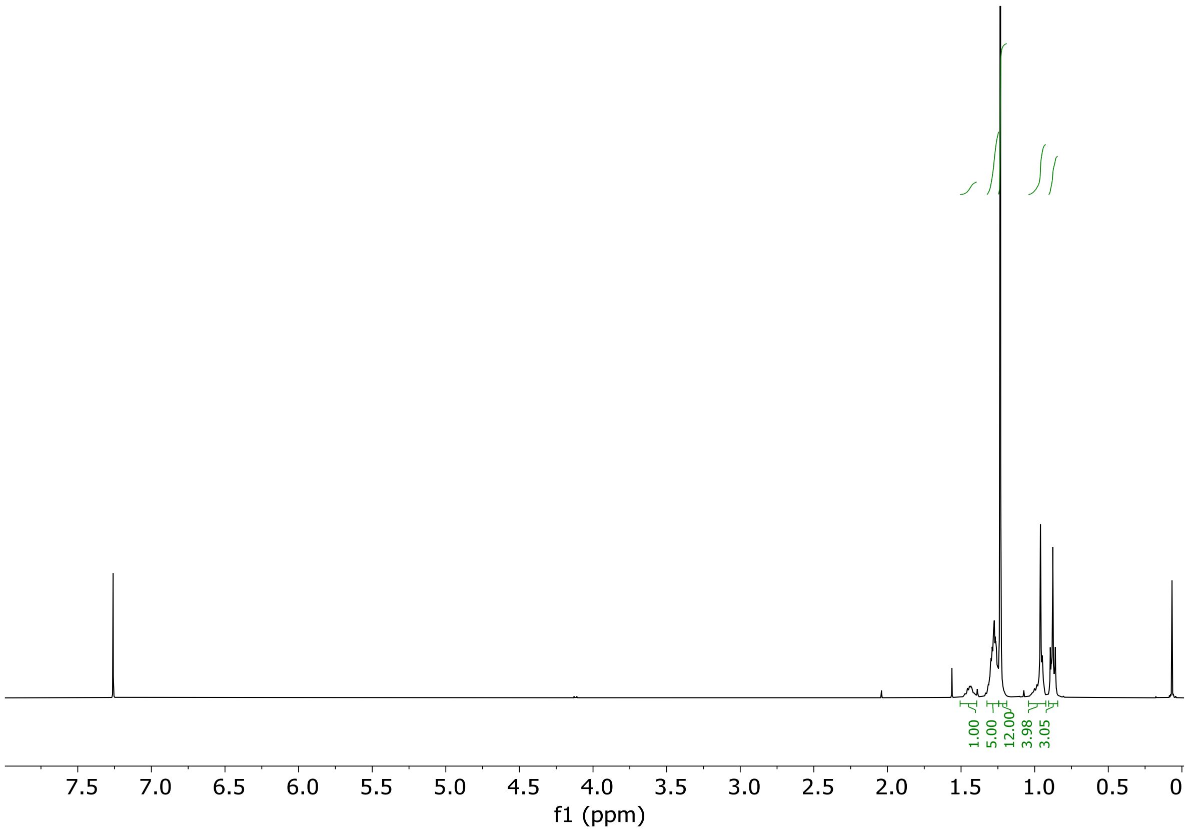

^{13}C NMR (101 MHz, $CDCl_3$) of compound **F4**. See procedure

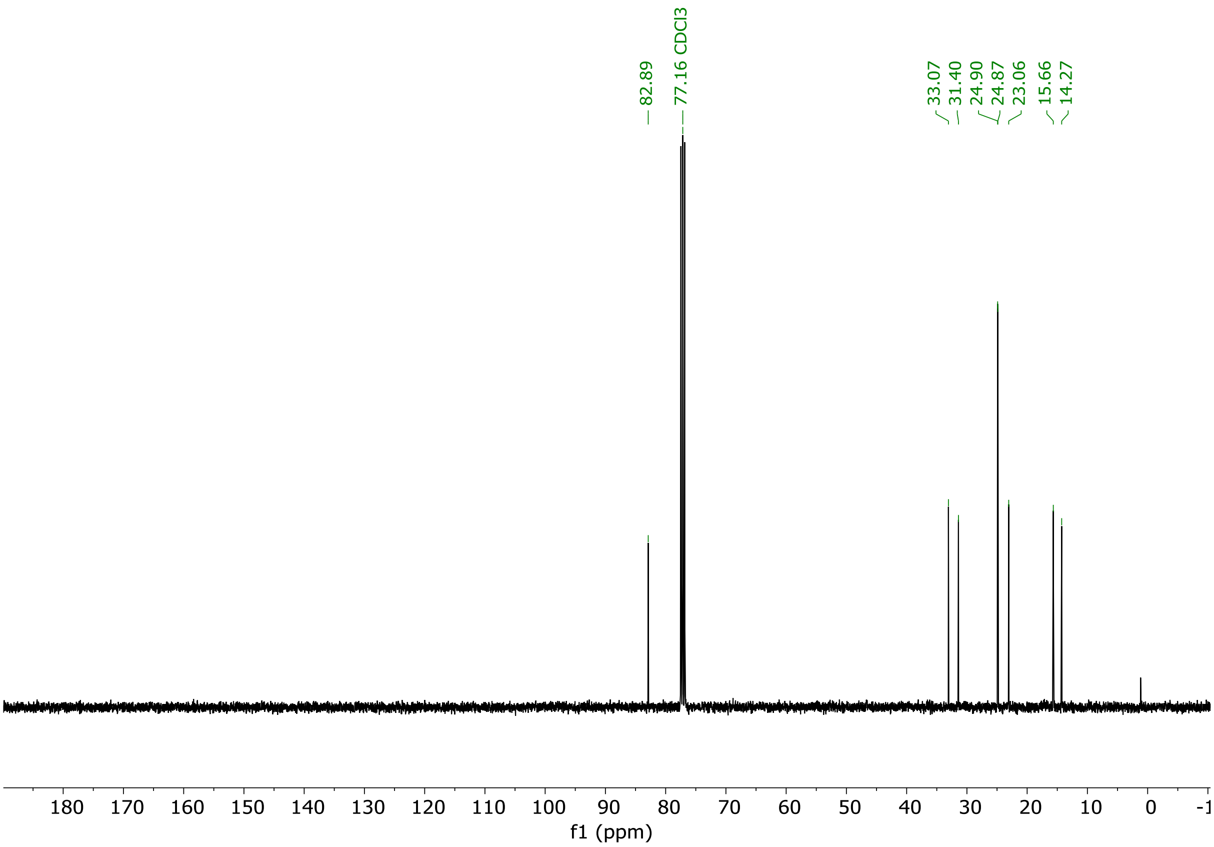

^{1}H NMR (600 MHz, $CDCl_3$) of compound **(3S)-S12**. See procedure.

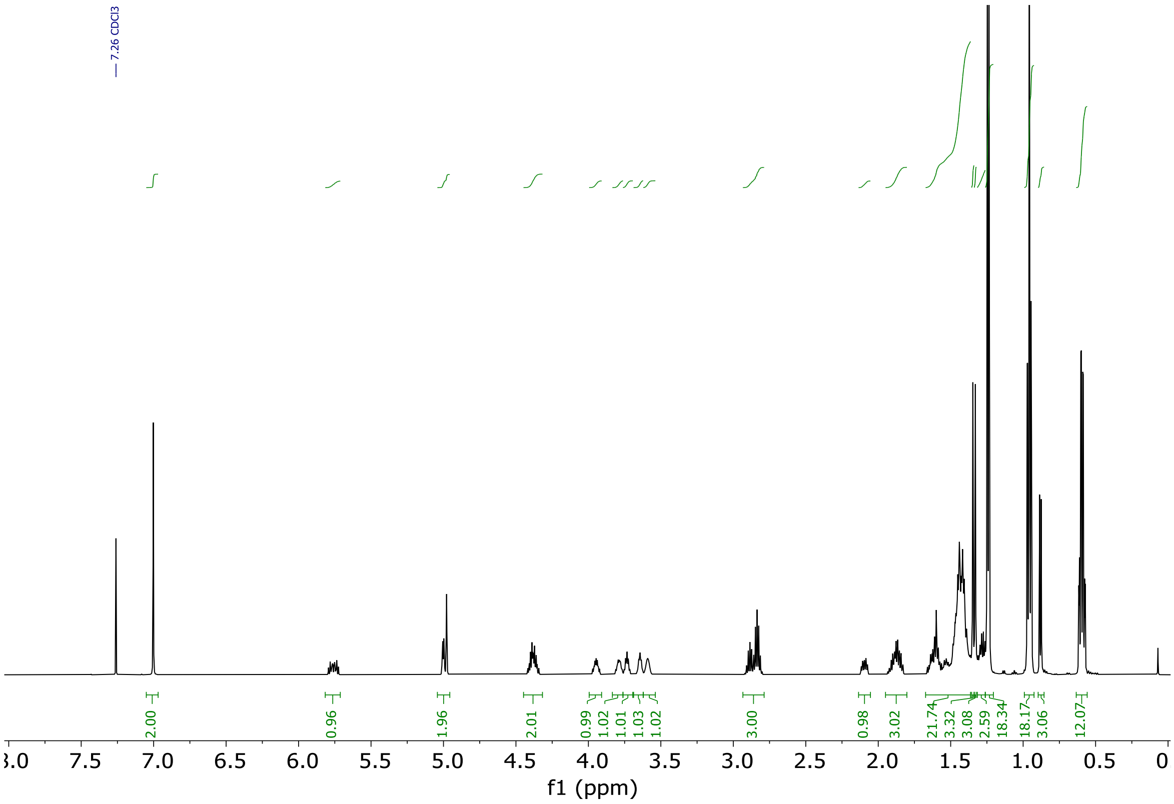

Zoomed in ^{1}H NMR region containing C*H*OH chemical shifts of **(3S)-S12**

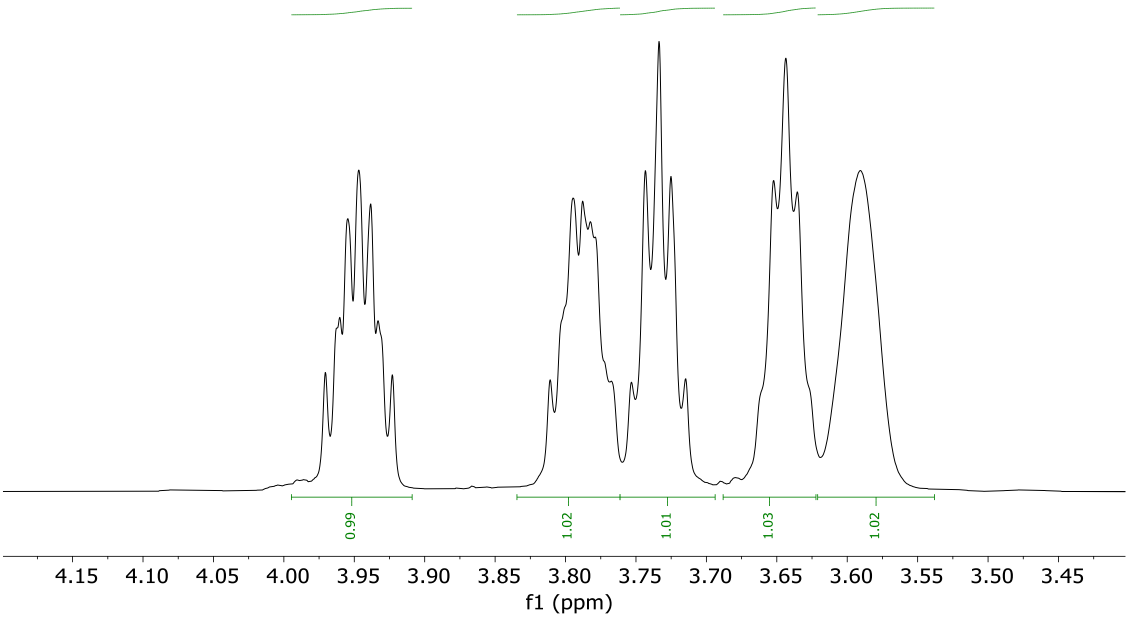

^{13}C NMR (151 MHz, $CDCl_3$) of compound **(3S)-S12**. *See procedure*.

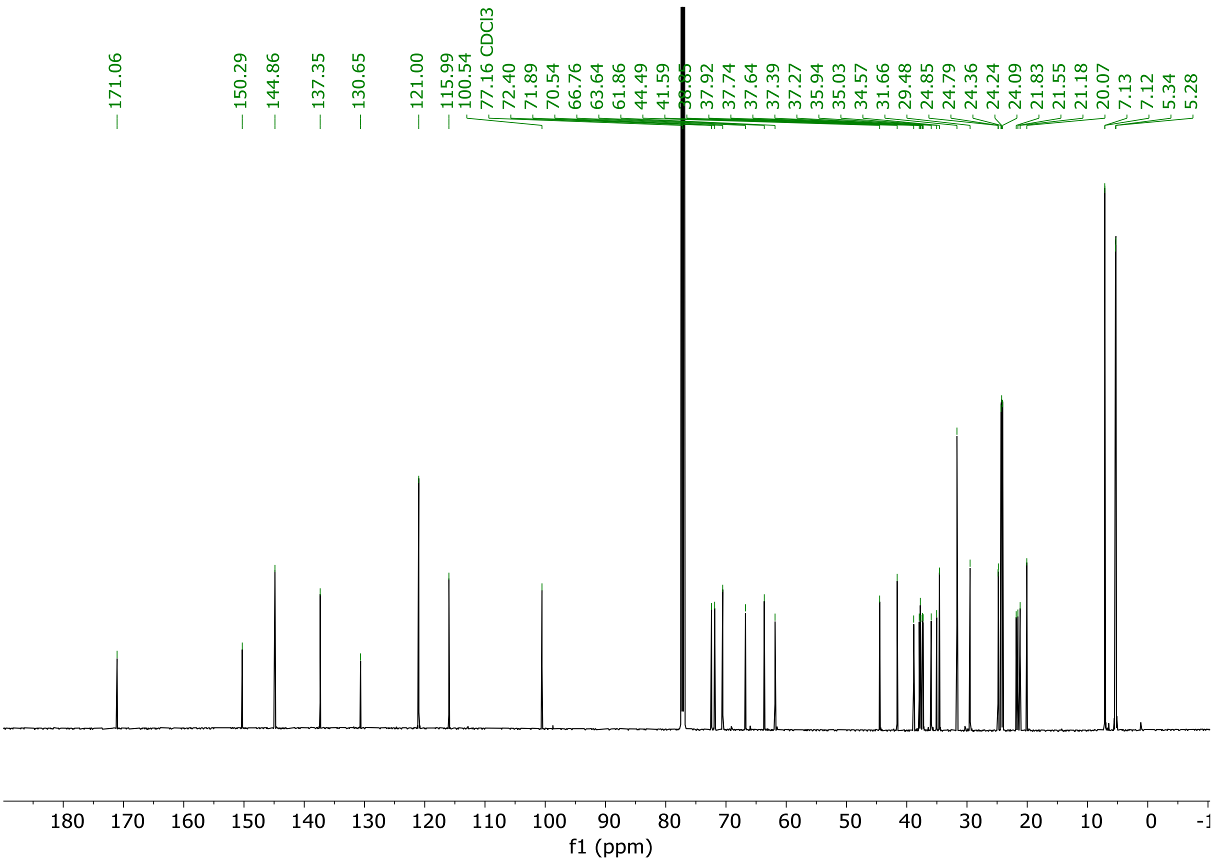

^{1}H NMR (600 MHz, $CDCl_3$) of compound **(3S)-CF_1-2**. *See procedure*.

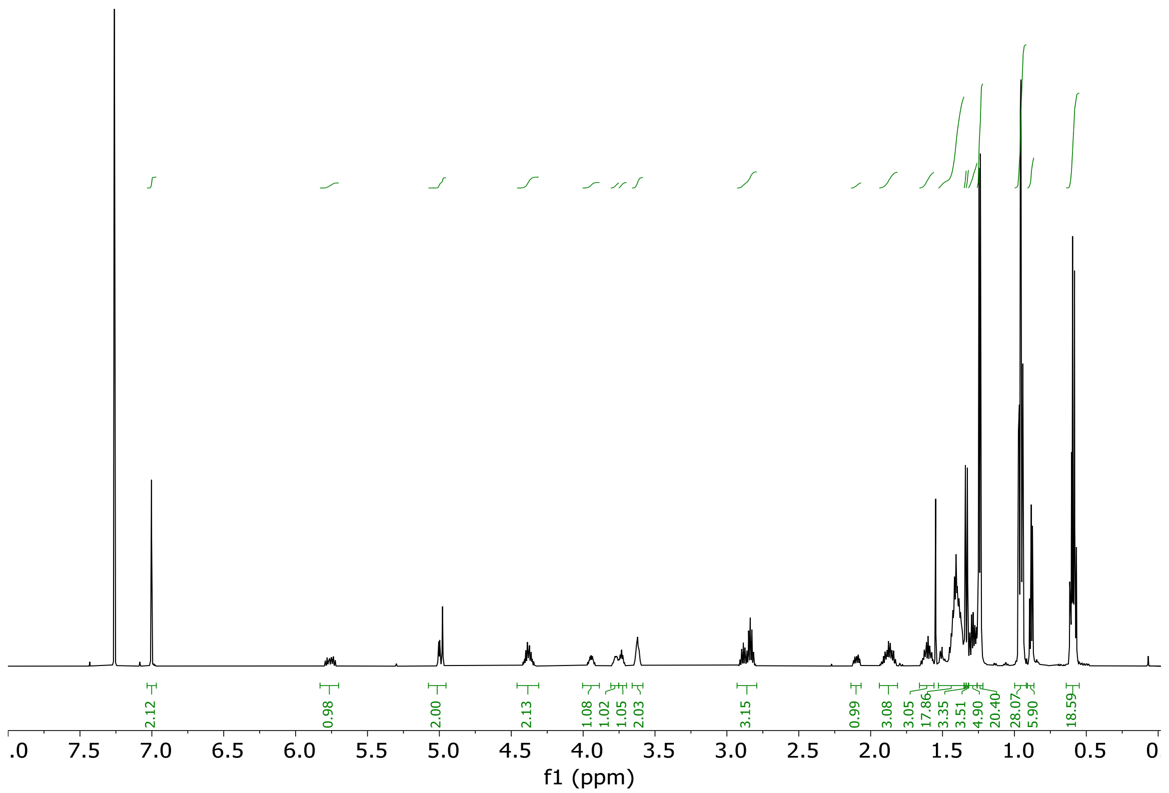

^{13}C NMR (151 MHz, $CDCl_3$) of compound **(3S)-CF_1-2**. *See procedure*.

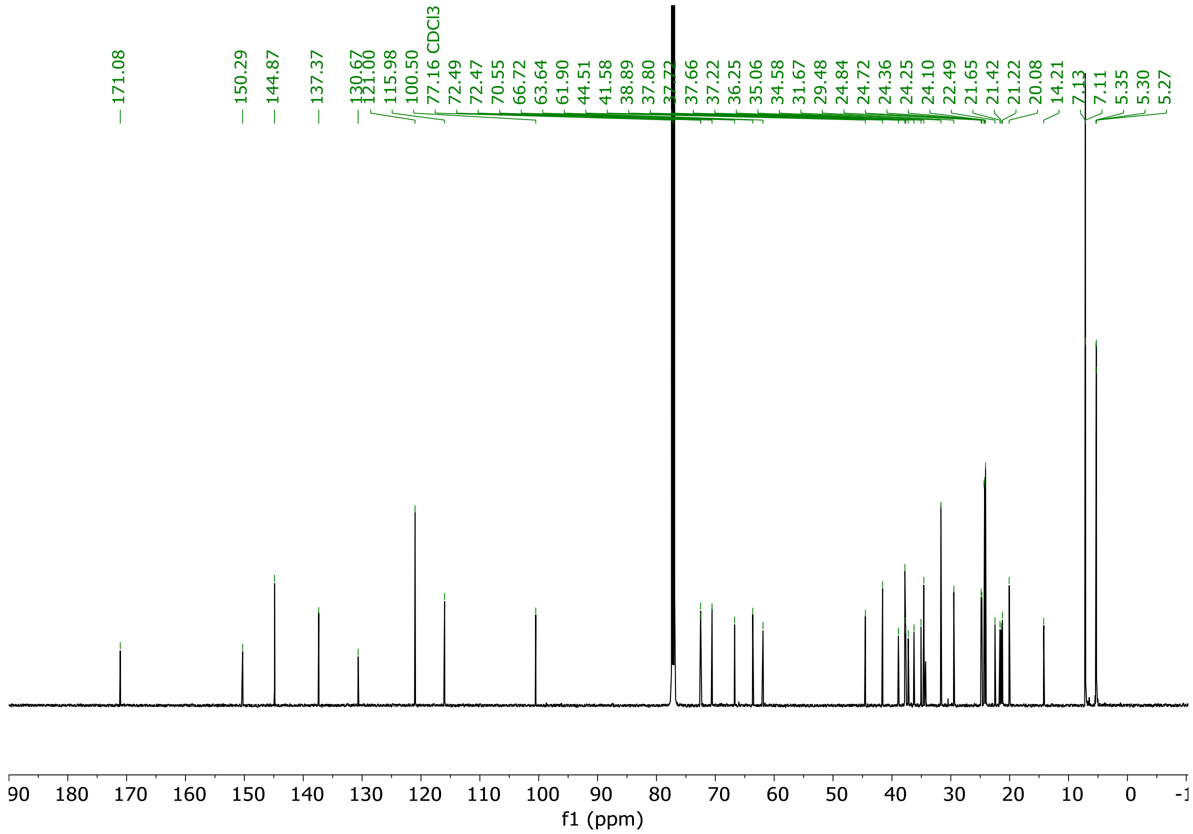

^{1}H NMR (500 MHz, $CDCl_3$) of compound **(3*S*)-17**. *See procedure*.

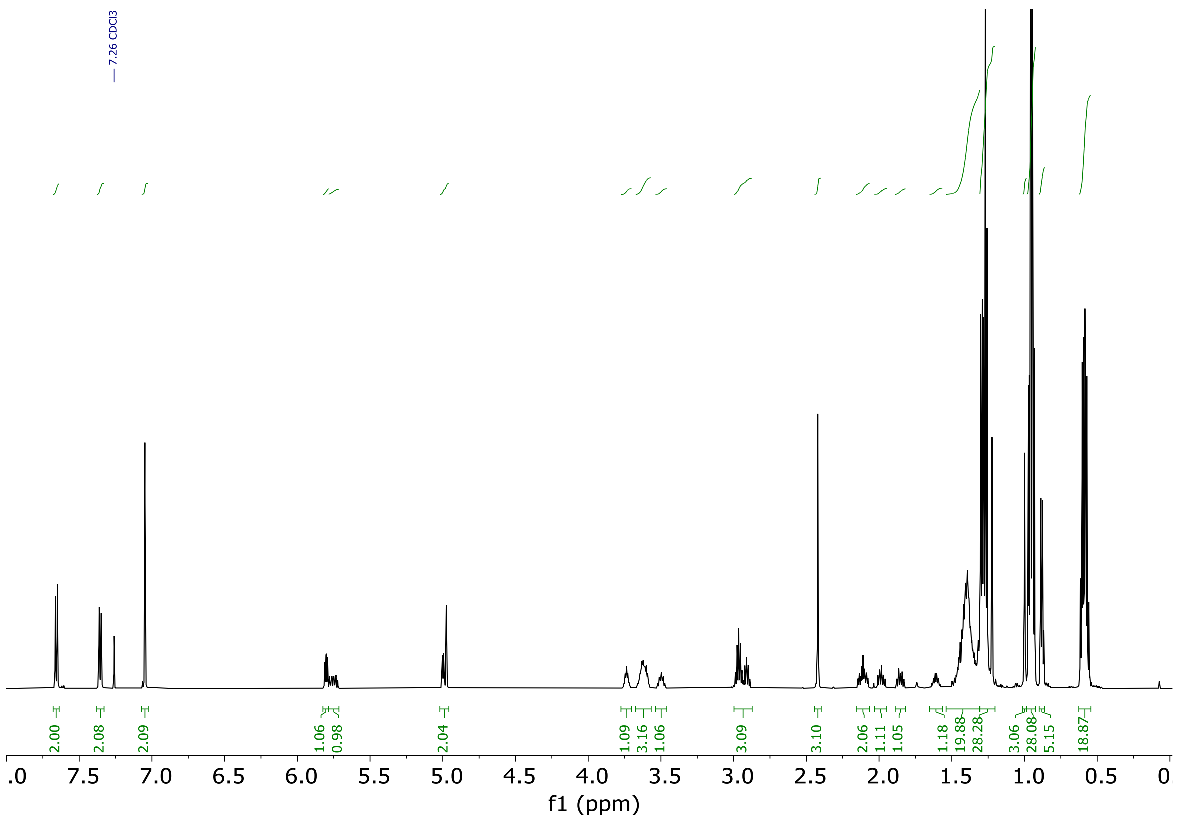

^{13}C NMR (125 MHz, $CDCl_3$) of compound **(3*S*)-17**. *See procedure*.

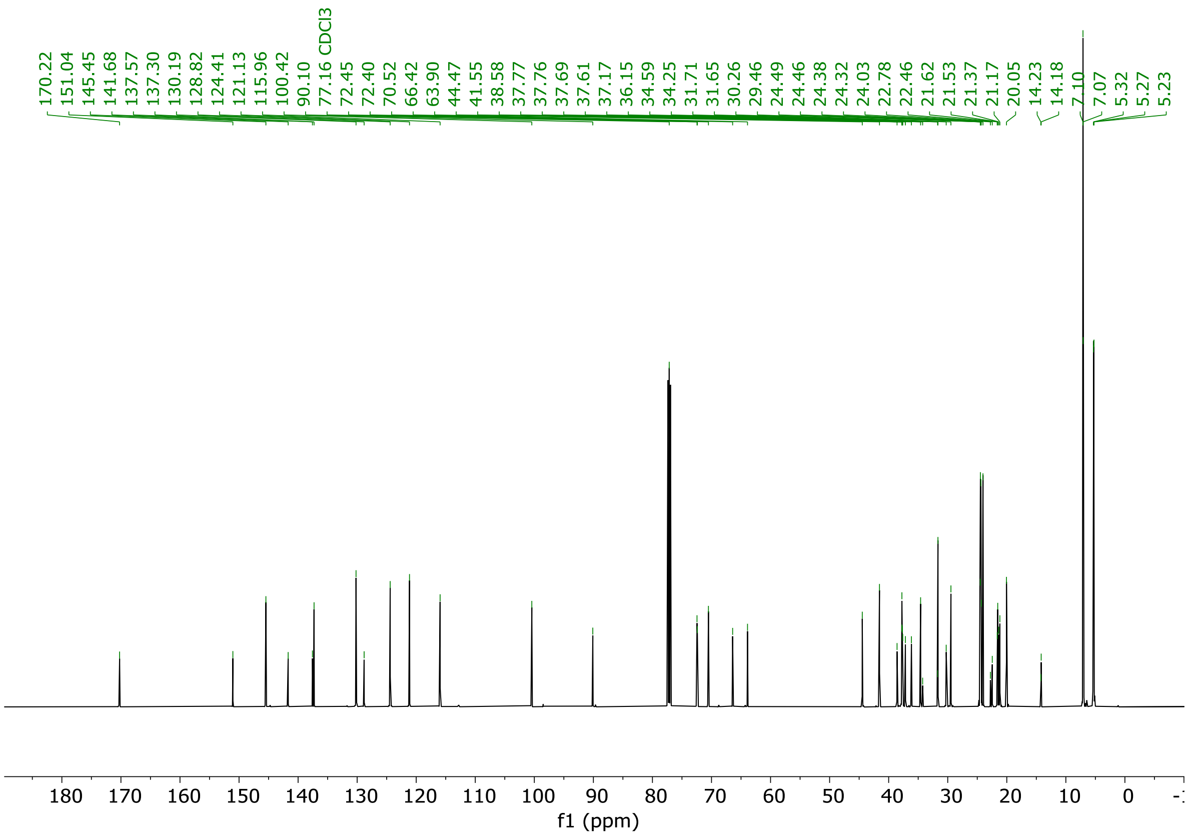

^{1}H NMR (600 MHz, $CDCl_3$) of compound **(3*S*)-S13**. See procedure.

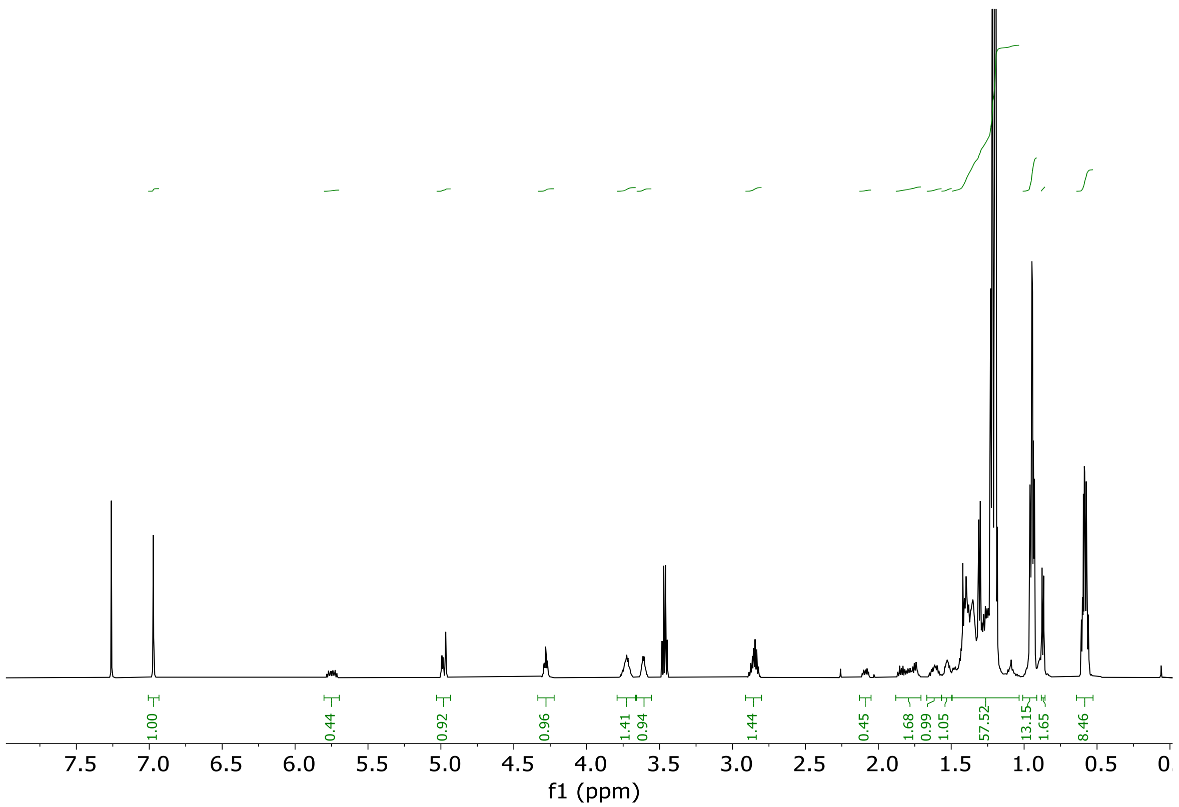

^{13}C NMR (151 MHz, $CDCl_3$) of compound **(3*S*)-S13**. See procedure.

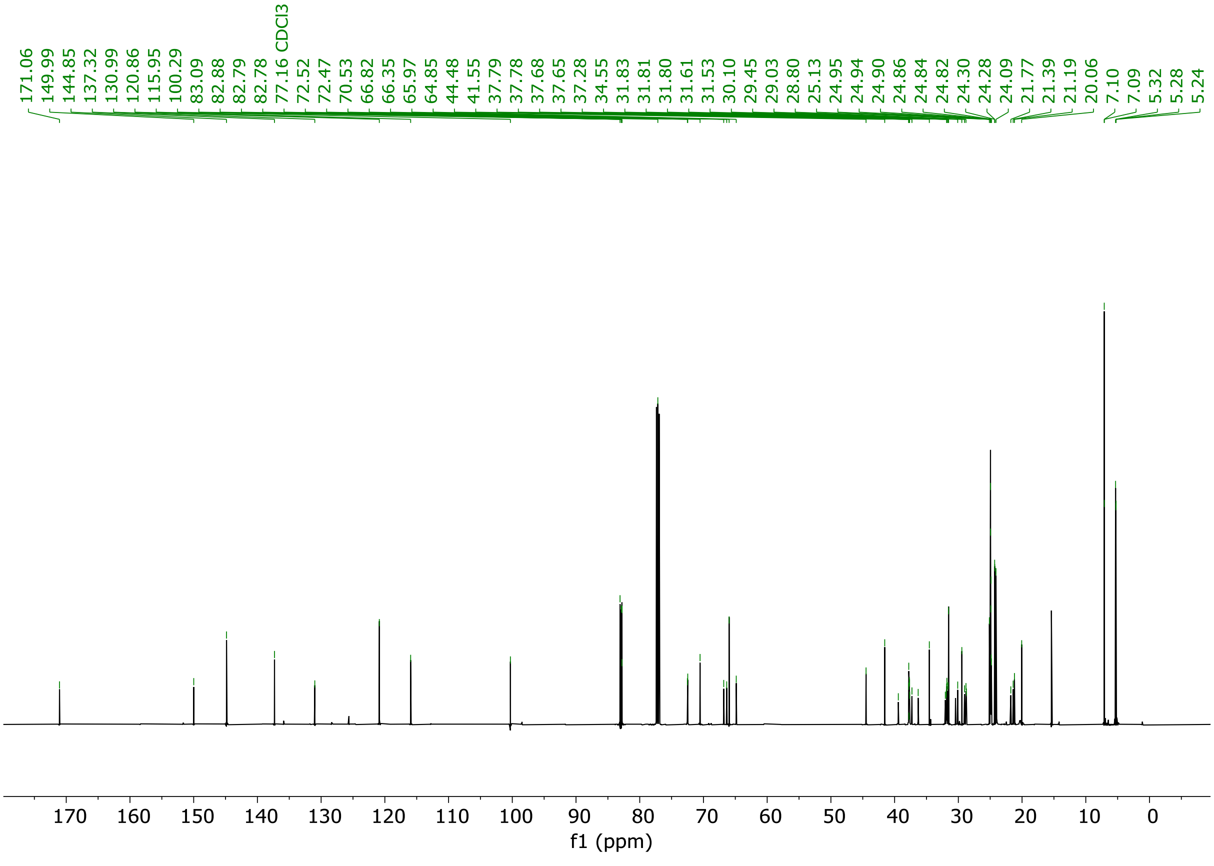

^{1}H NMR (500 MHz, CDCl$_3$) of compound **(3*S*)-CF_1-3**. See procedure.

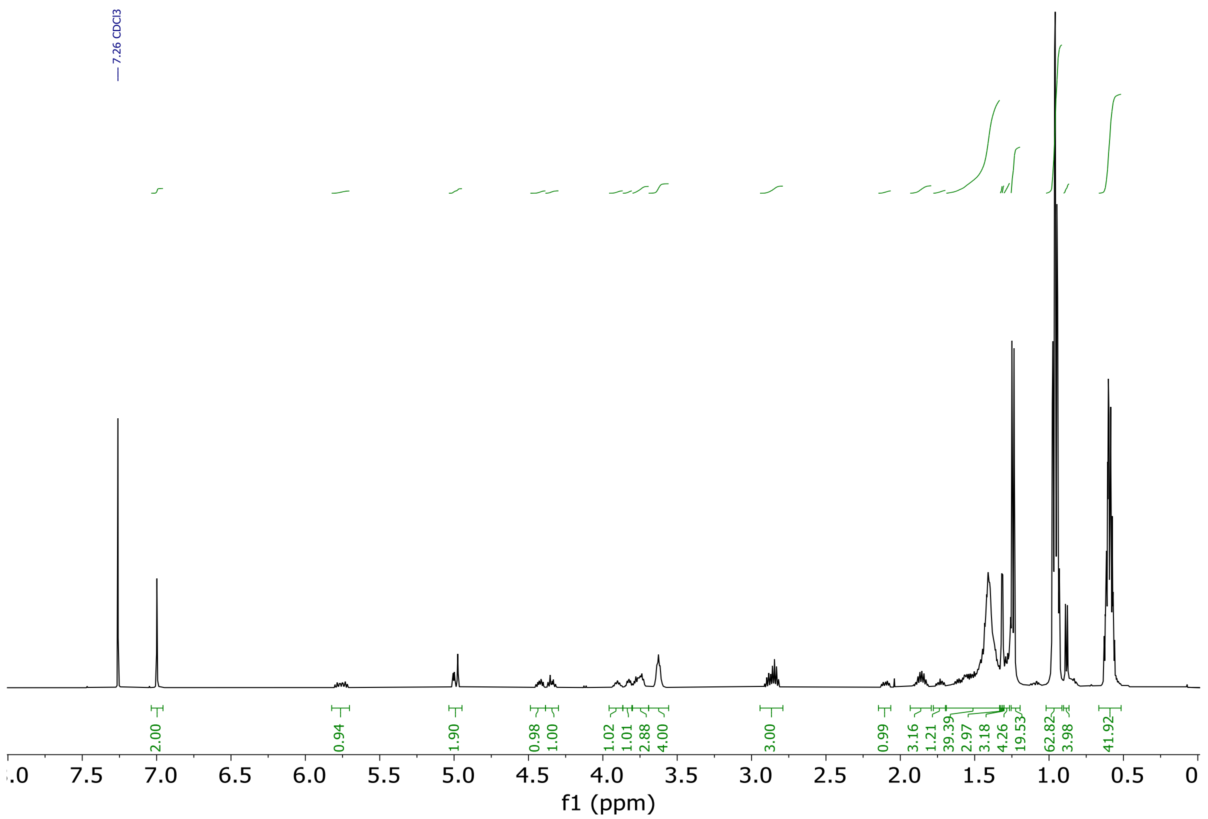

^{13}C NMR (125 MHz, CDCl$_3$) of compound **(3*S*)-CF_1-3**. See procedure.

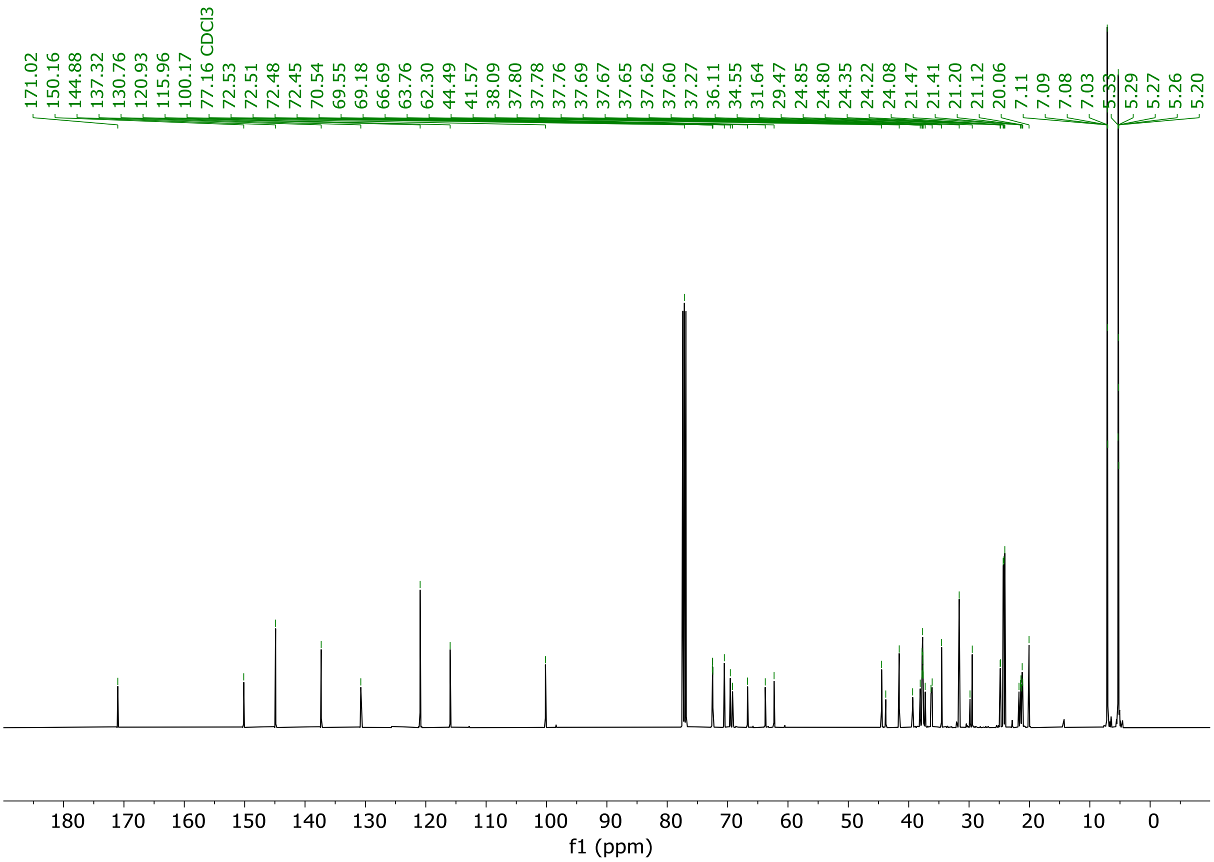

^{1}H NMR (600 MHz, $CDCl_3$) of compound **(3*S*, 36*S*/*R*)-CF_1-4**. See procedure.

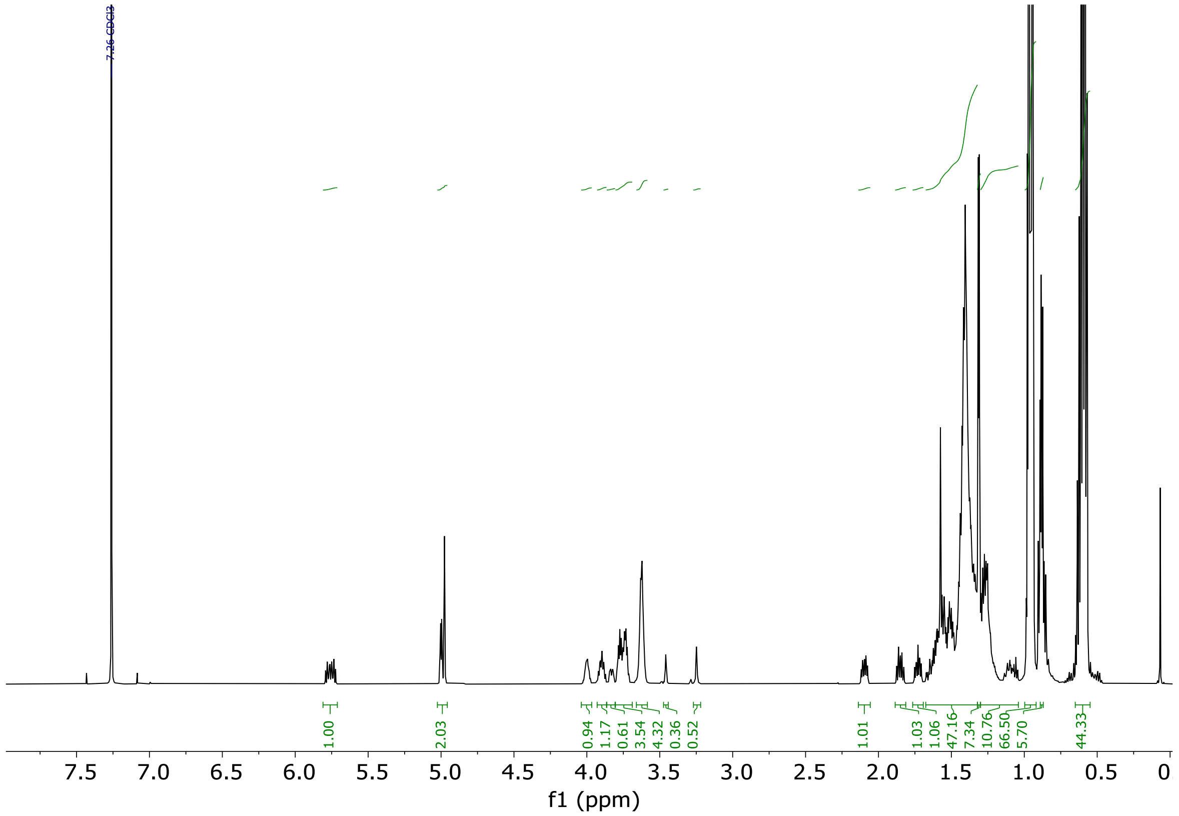

^{13}C NMR (101 MHz, $CDCl_3$) of compound **(3*S*, 36*S*/*R*)-CF_1-4**. See procedure.

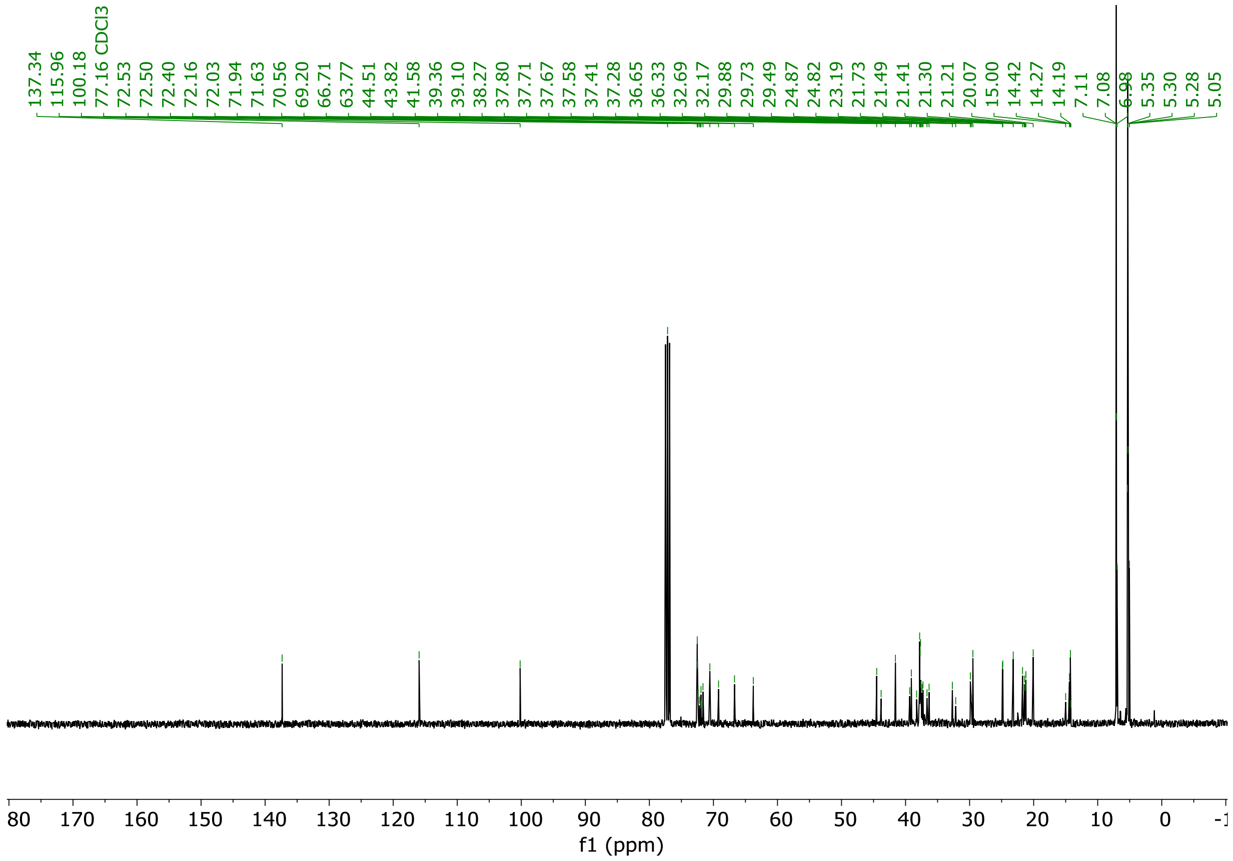

^{1}H NMR (600 MHz, $CDCl_3$) of compound **(3*R*, 36*S*/*R*)-18**. [See procedure](#).

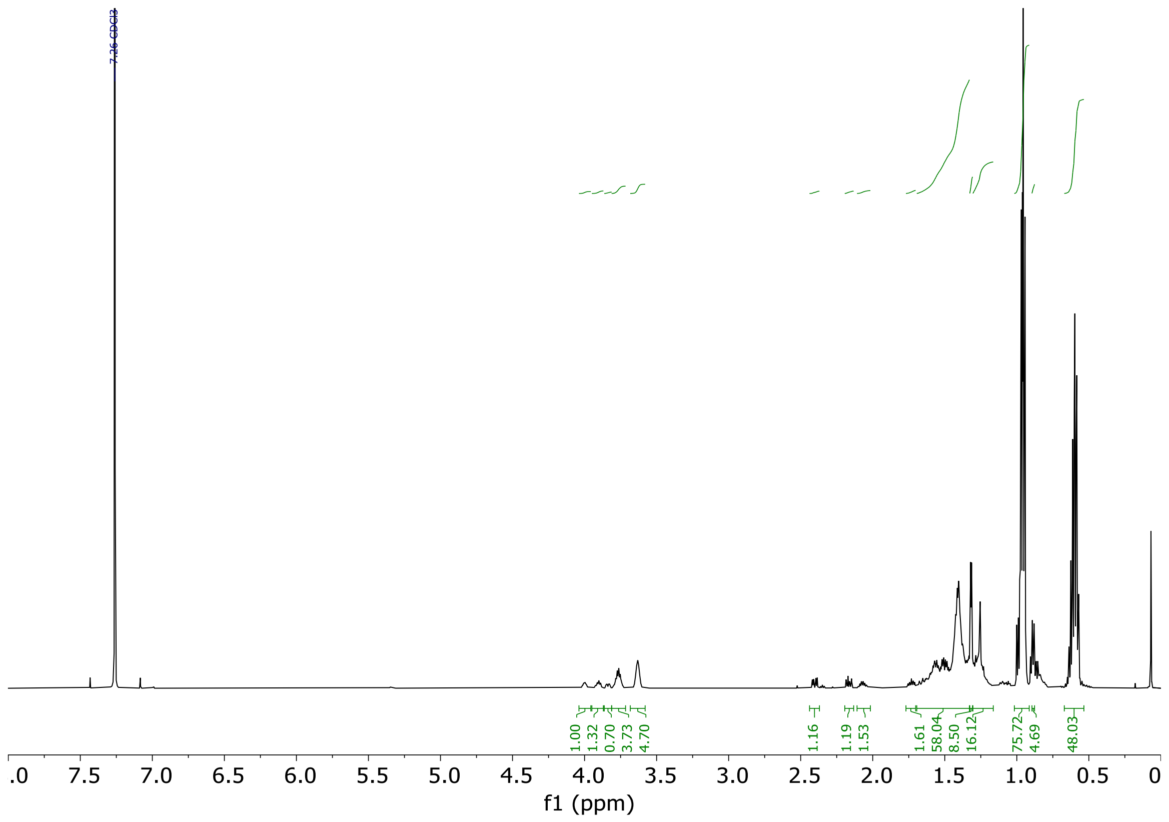

^{13}C NMR (101 MHz, $CDCl_3$) of compound **(3*R*, 36*S*/*R*)-18**. [See procedure](#).

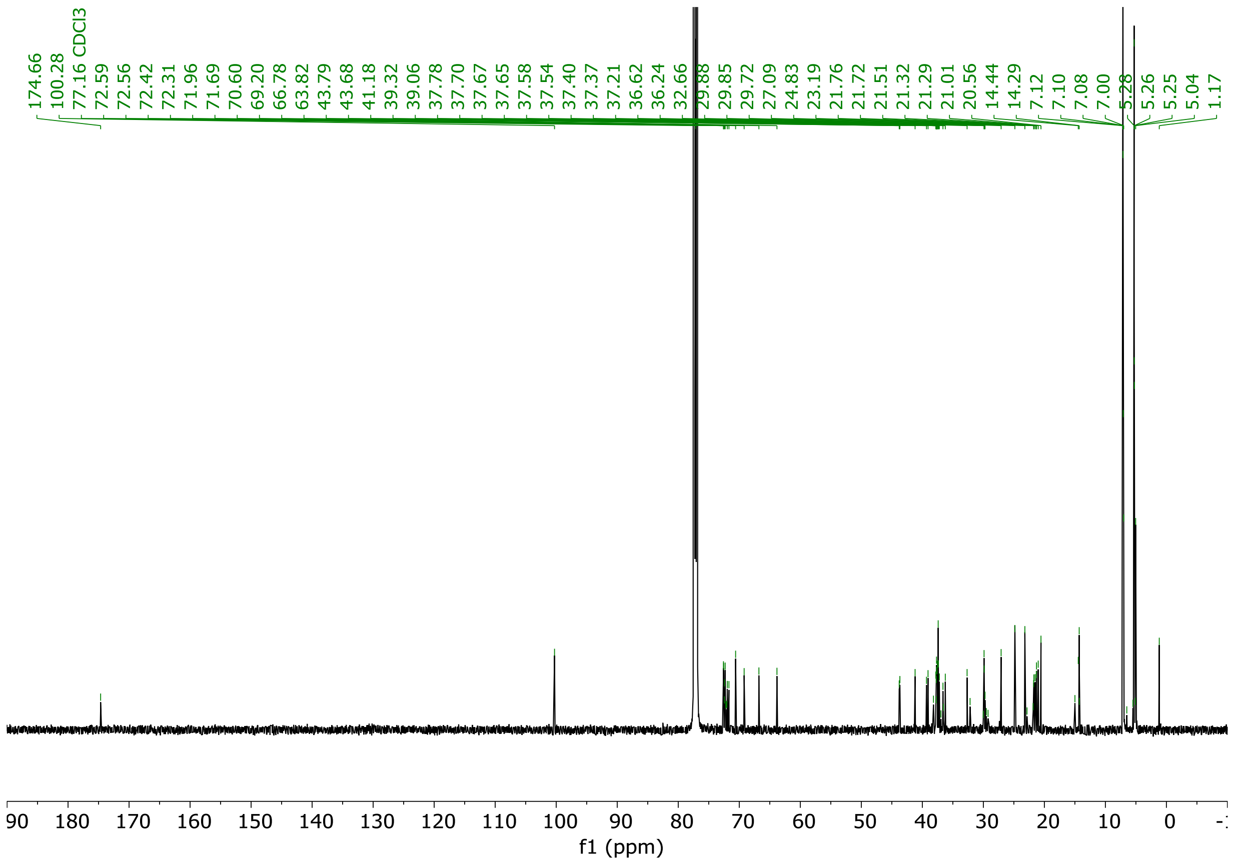

^{1}H NMR (600 MHz, $CDCl_3$) of compound **(3*R*, 36*S*/*R*)-19**. *See procedure*.

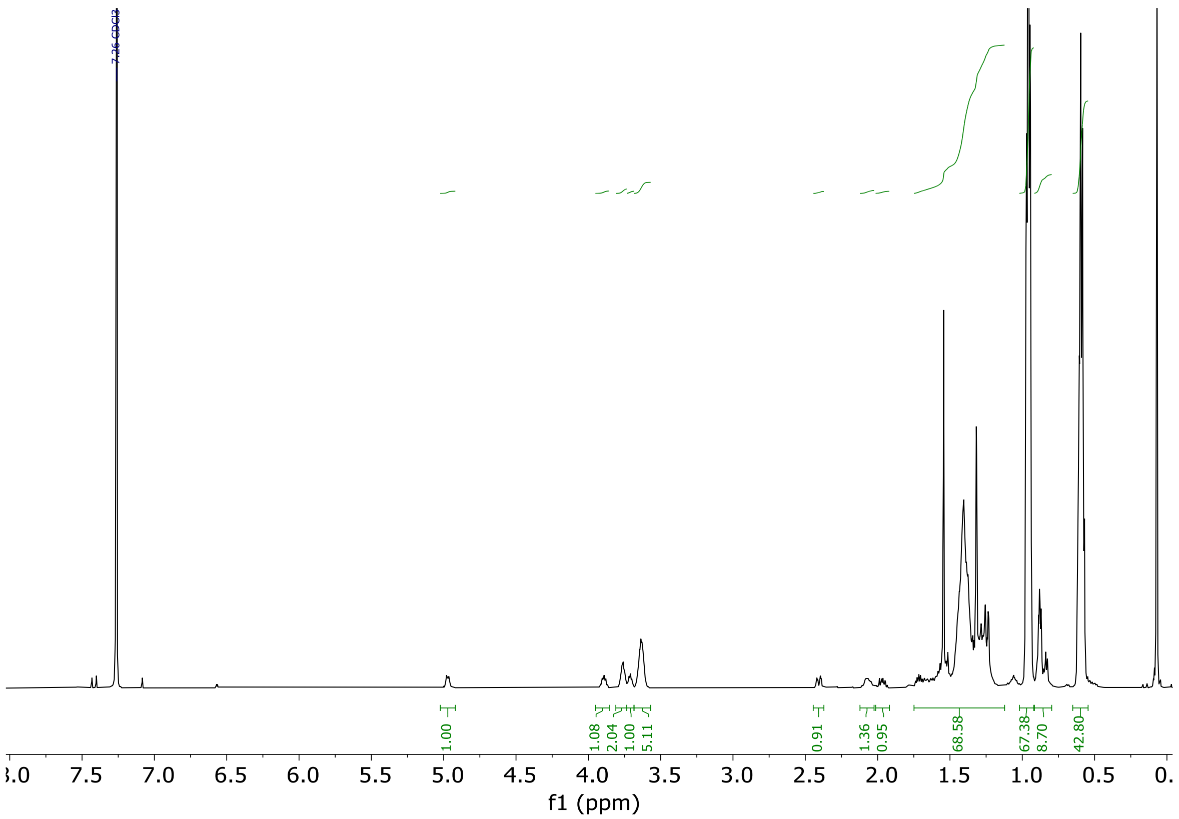

^{13}C NMR (151 MHz, $CDCl_3$) of compound **(3*R*, 36*S*/*R*)-19**. *See procedure*.

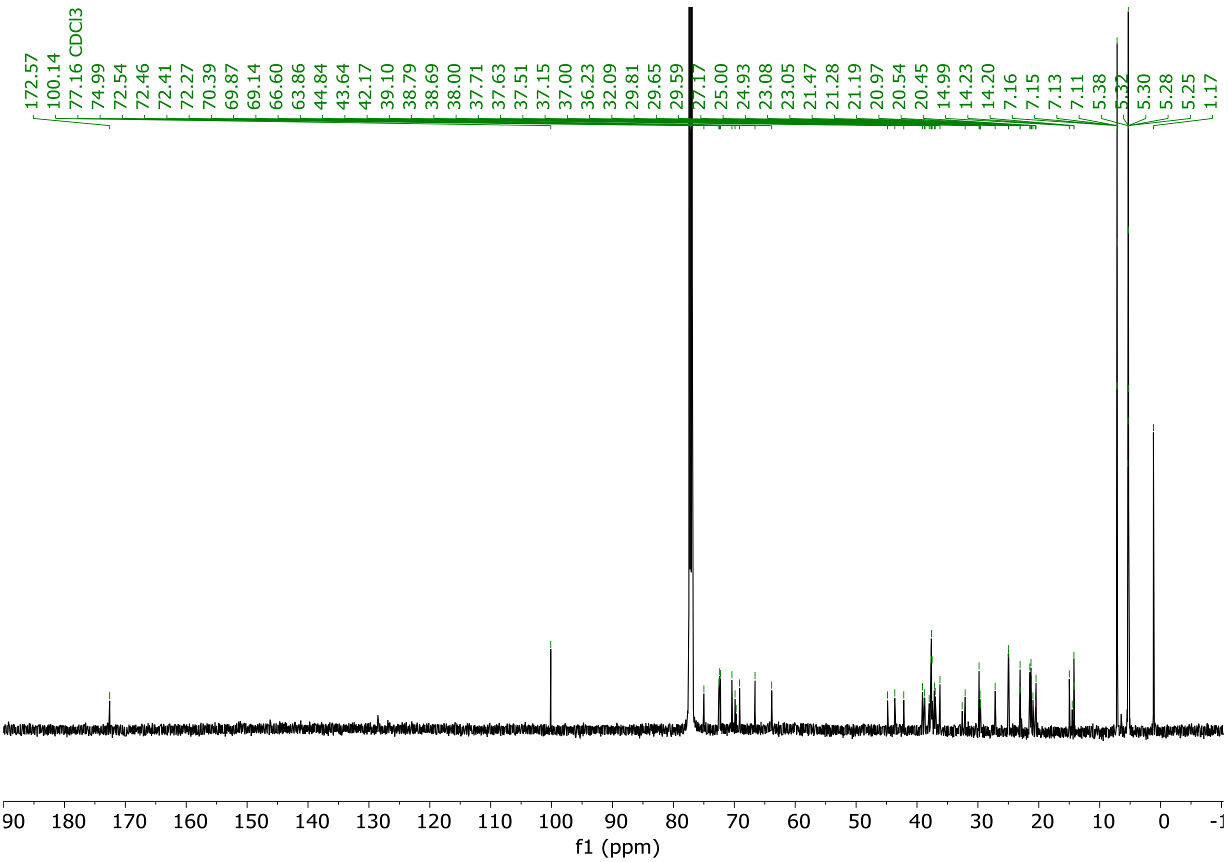

^{1}H NMR (600 MHz, $CDCl_3$) of compound **(3*R*, 36*S*/*R*)-CA**. See procedure.

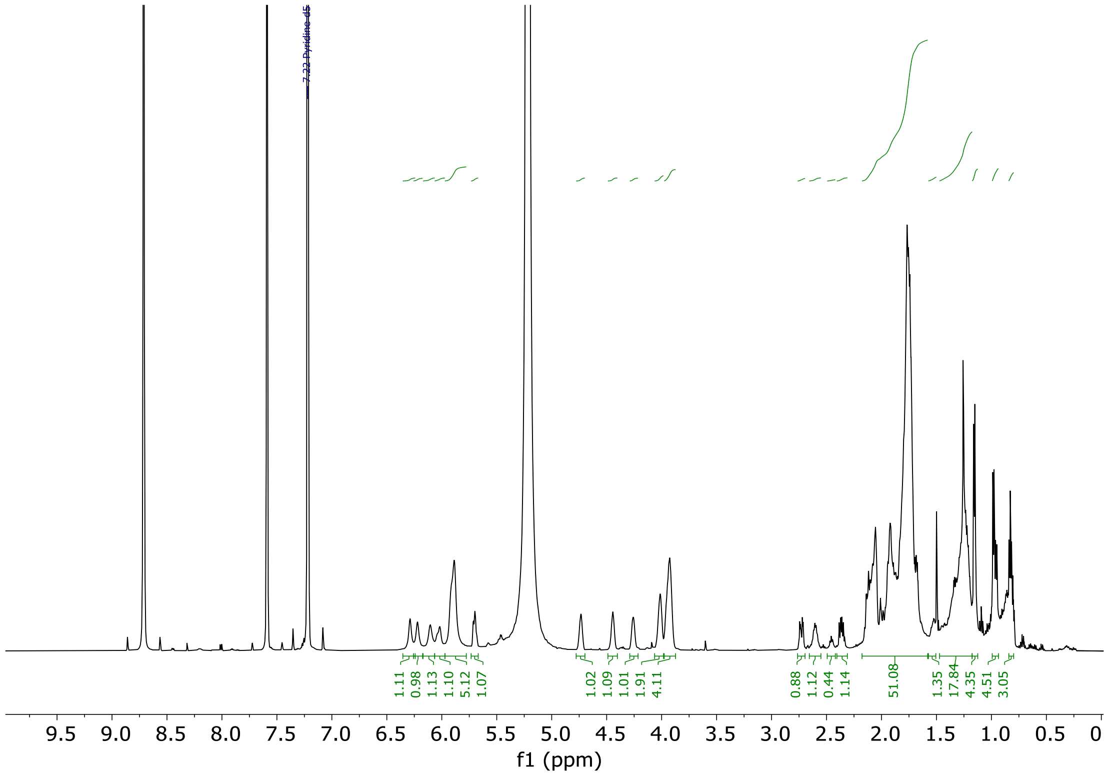

^{13}C NMR (151 MHz, $CDCl_3$) of compound **(3*R*, 36*S*/*R*)-CA**. See procedure.

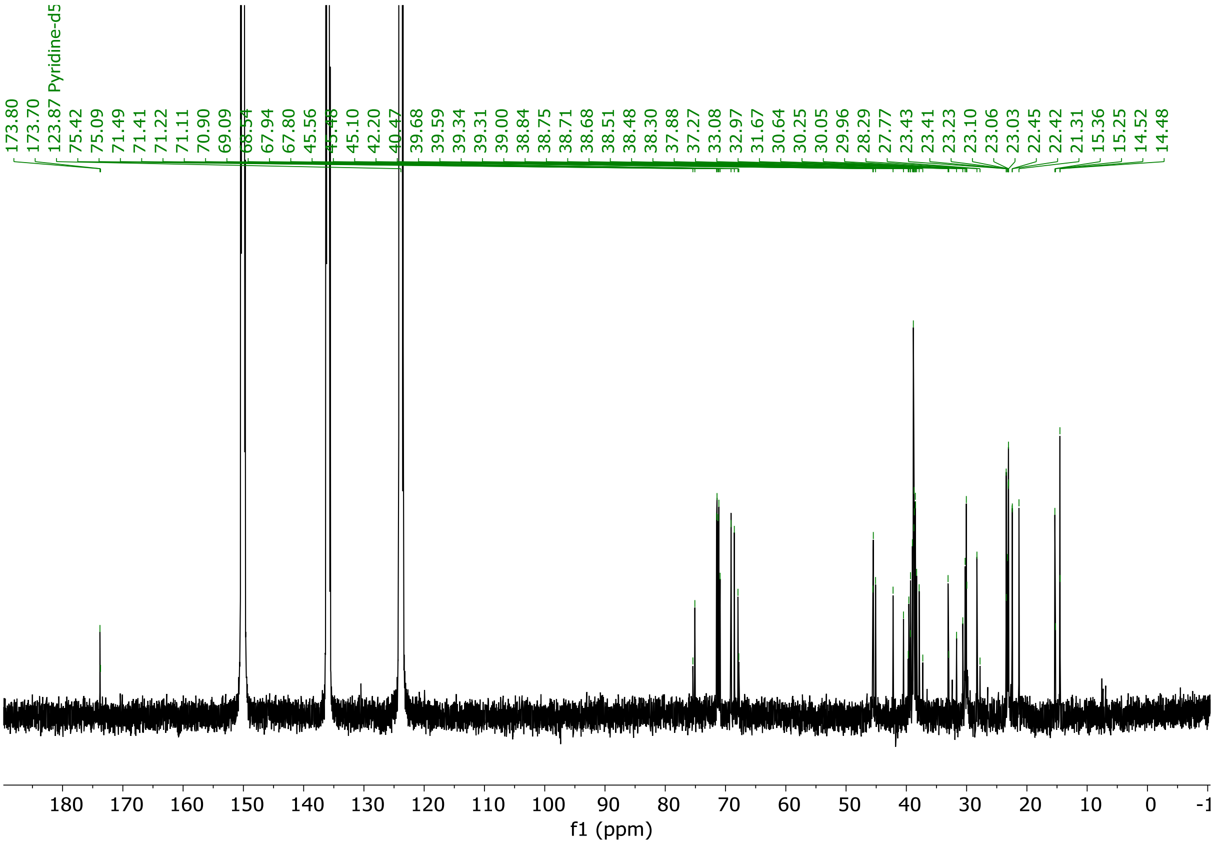

^{1}H NMR (600 MHz, $CDCl_3$) of compound **(3*R*)-S12**. See procedure.

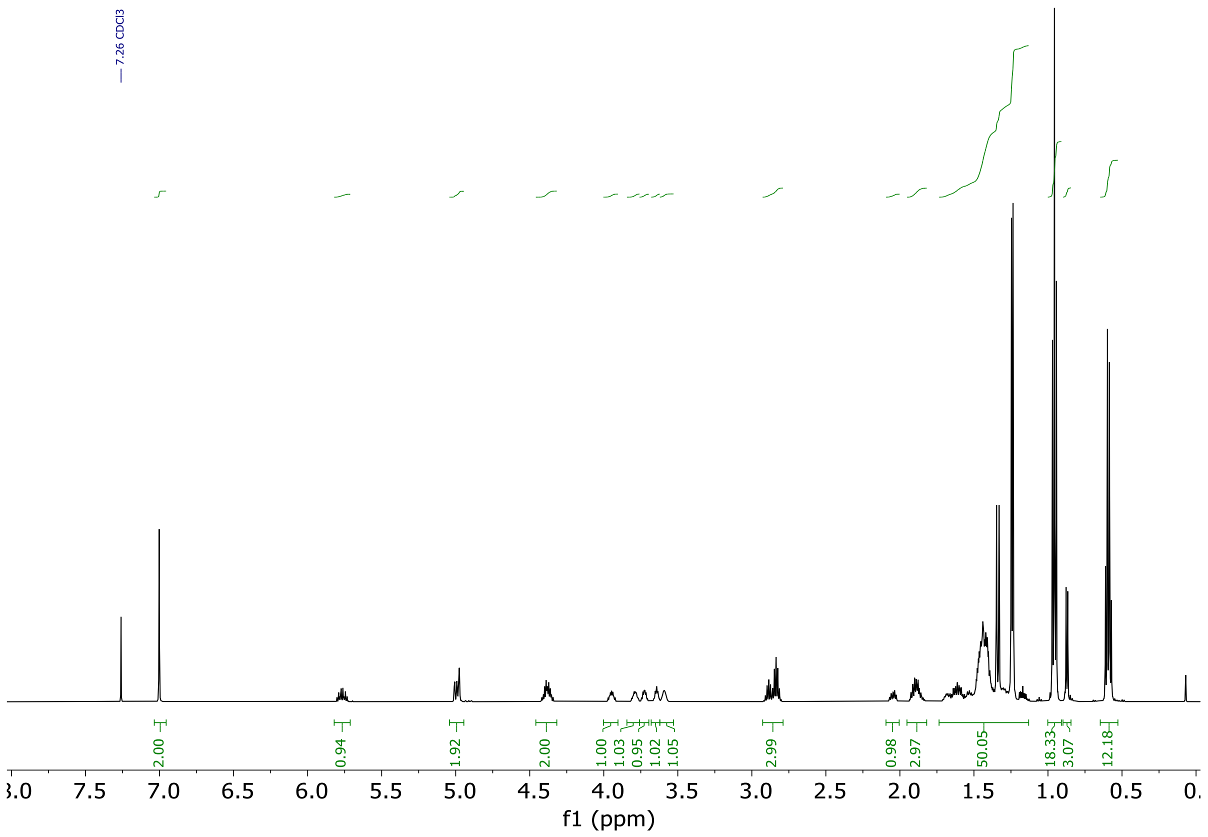

^{13}C NMR (151 MHz, $CDCl_3$) of compound **(3*R*)-S12**. See procedure.

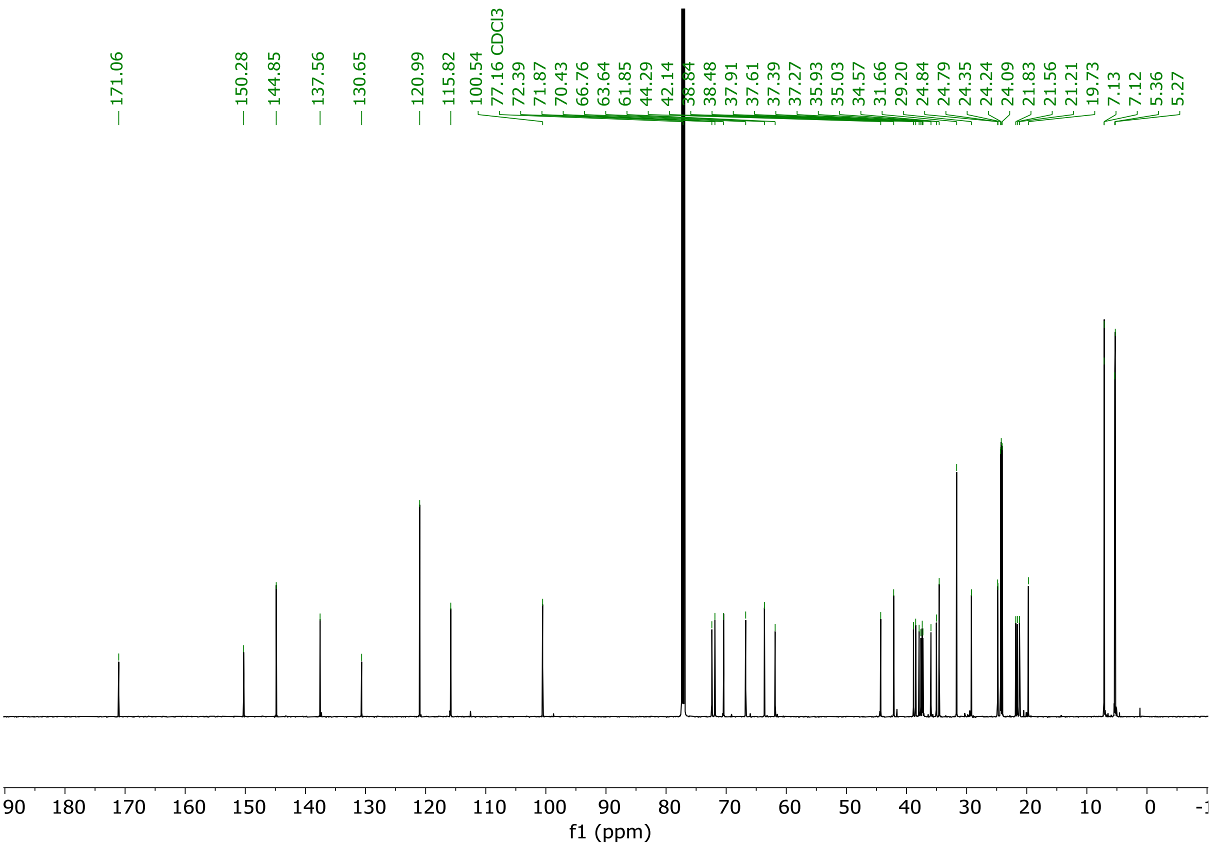

^{1}H NMR (500 MHz, $CDCl_3$) of compound **(3*R*)-CF_1-2**. *See procedure*.

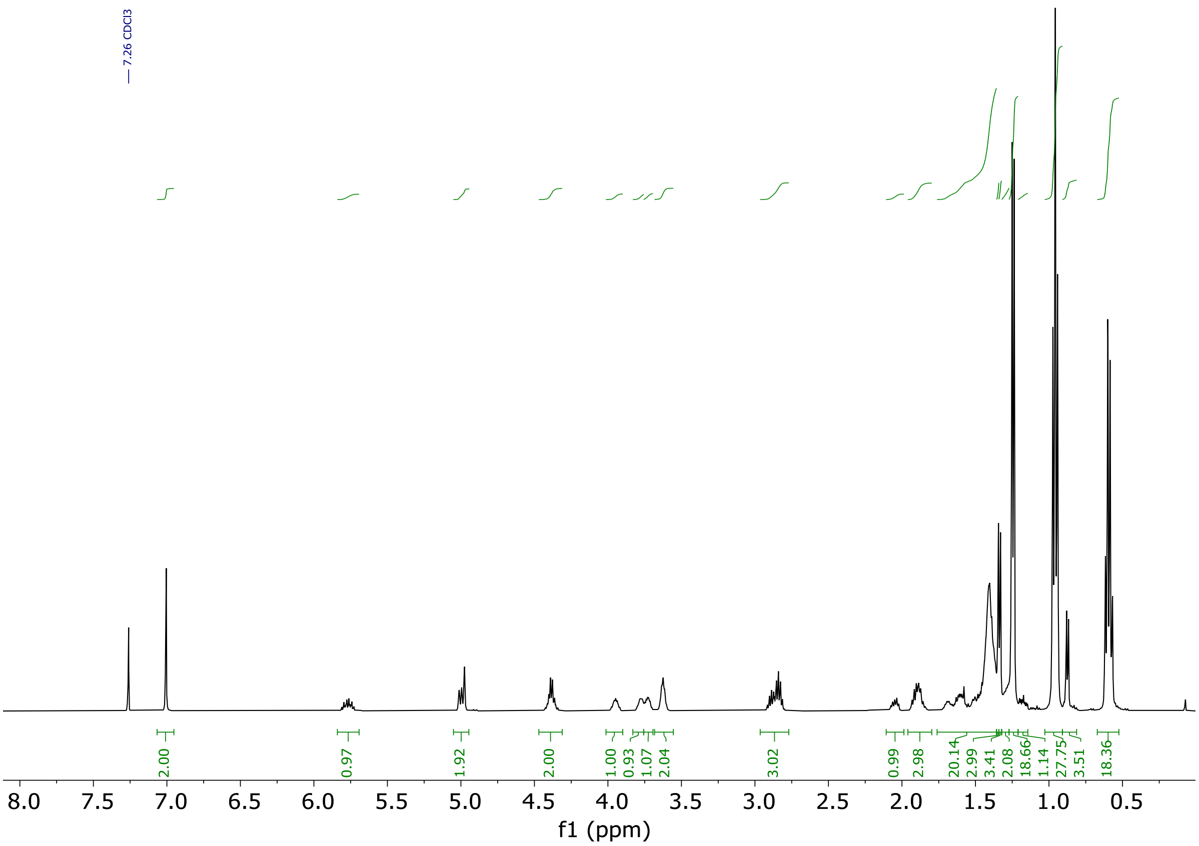

^{13}C NMR (125 MHz, $CDCl_3$) of compound **(3*R*)-CF_1-2**. *See procedure*.

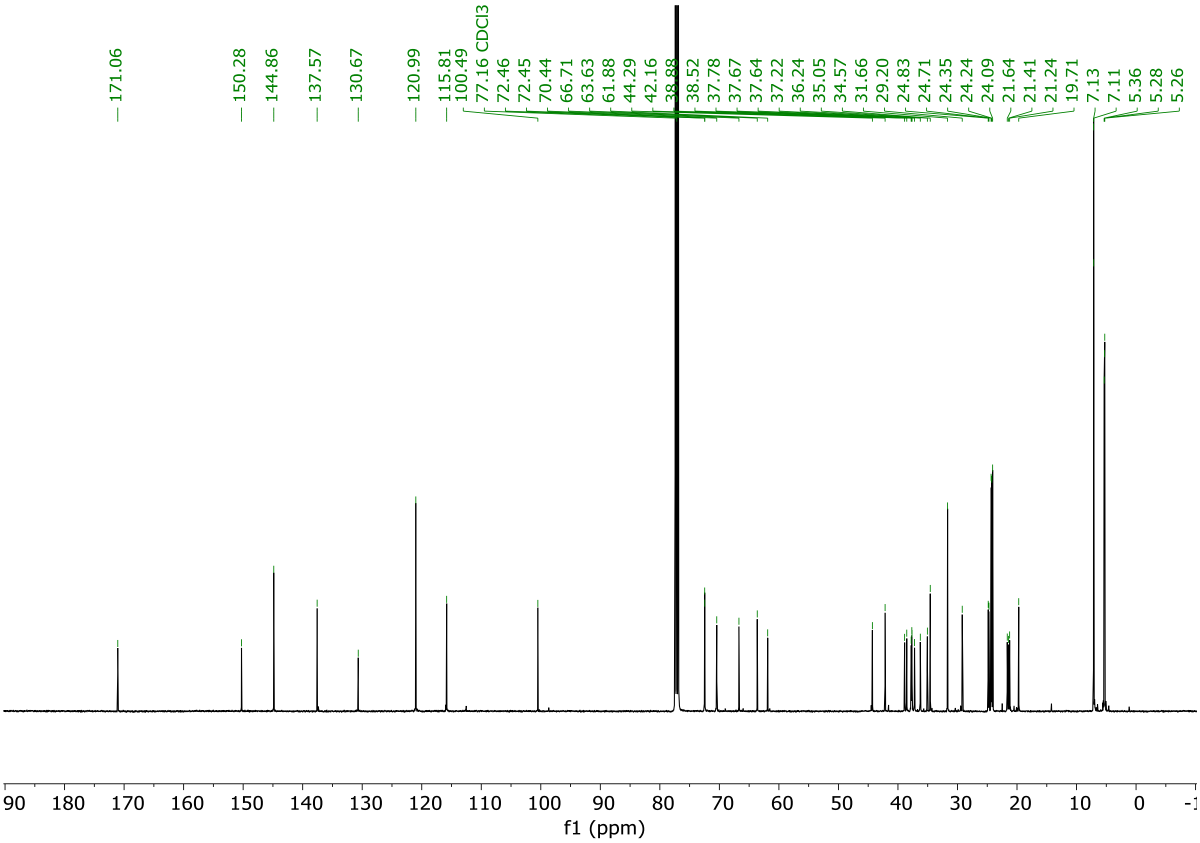

^{1}H NMR (600 MHz, $CDCl_3$) of compound **(3*R*)-17**. See procedure.

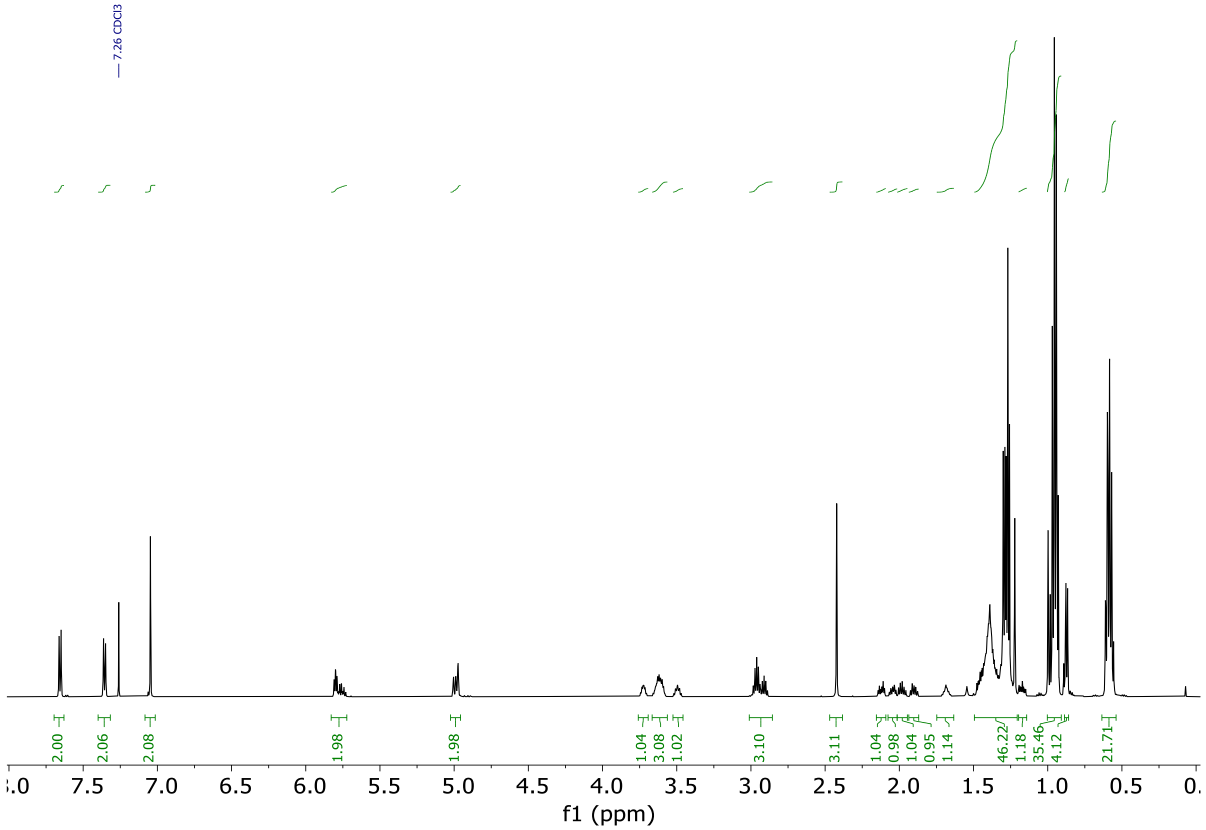

^{13}C NMR (151 MHz, $CDCl_3$) of compound **(3*R*)-17**. See procedure.

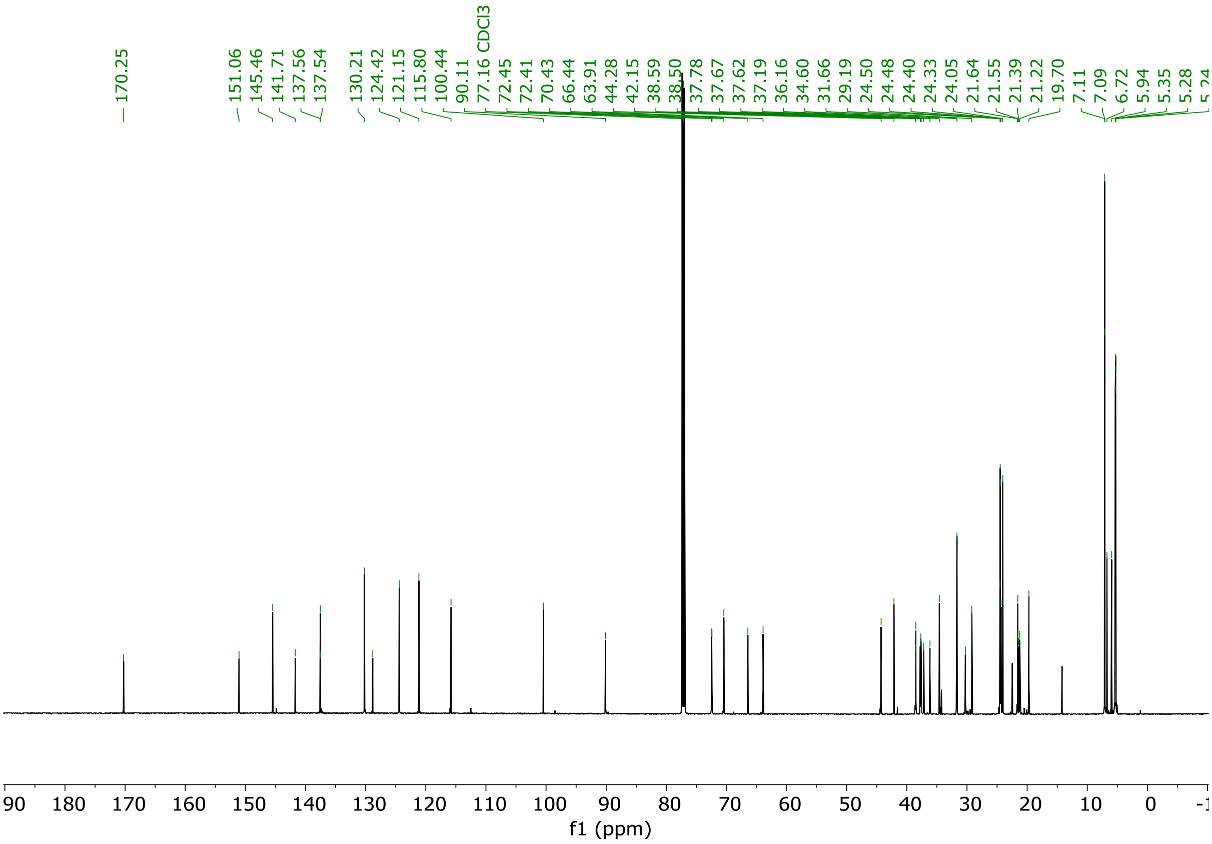

^{1}H NMR (600 MHz, $CDCl_3$) of compound **(3*R*)-S13**. *See procedure*.

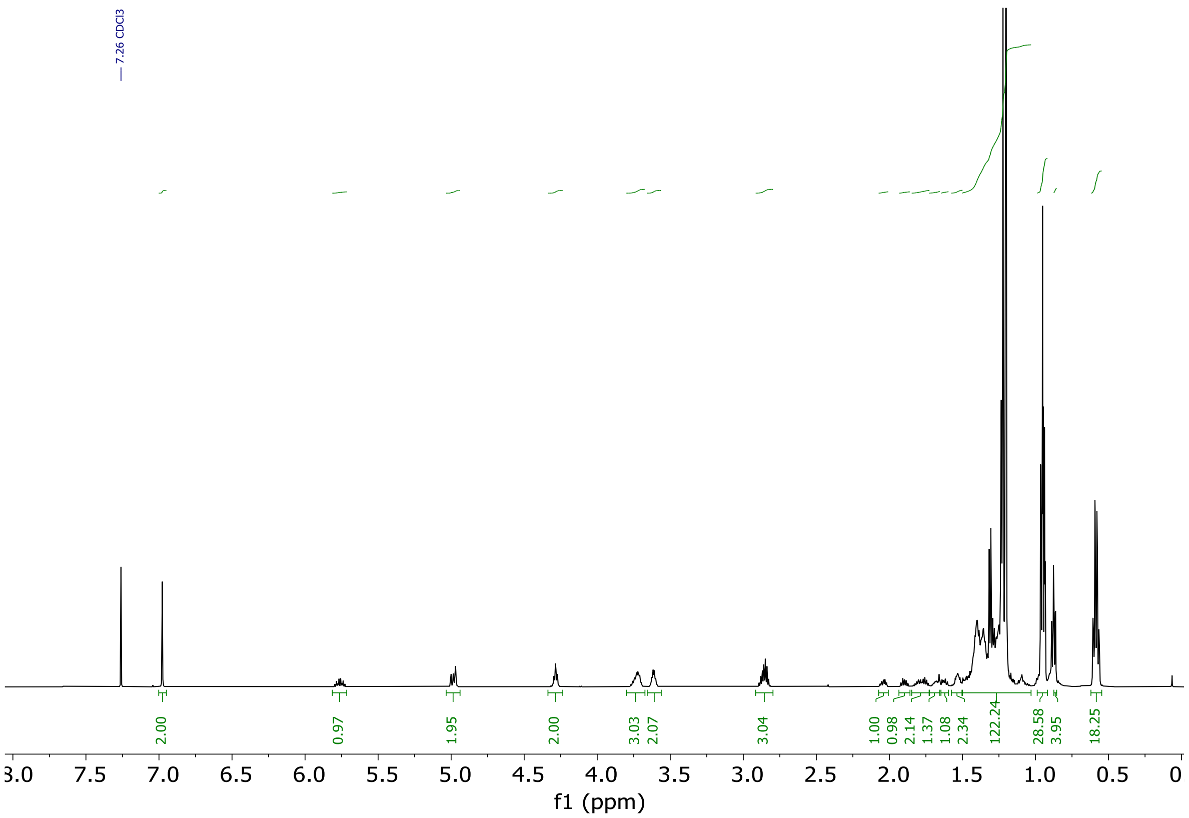

^{13}C NMR (151 MHz, $CDCl_3$) of compound **(3*R*)-S13**. *See procedure*.

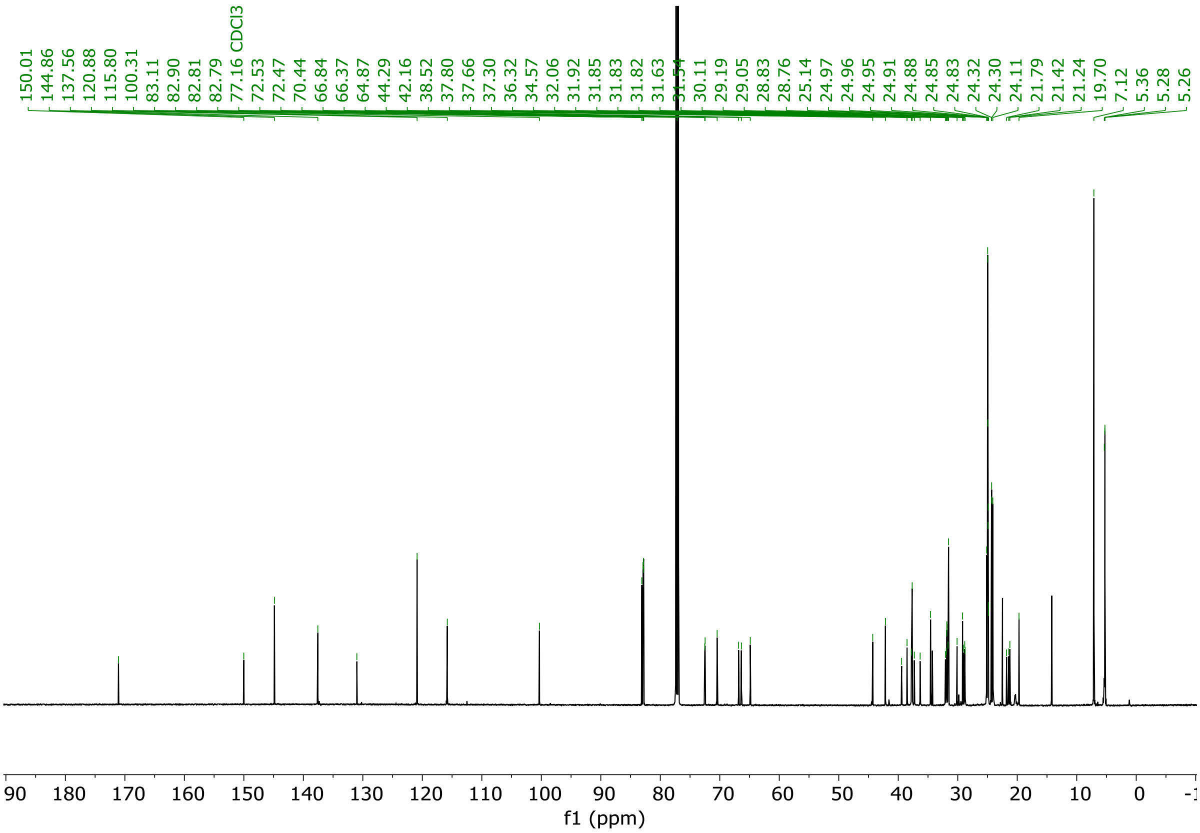

^{1}H NMR (500 MHz, $CDCl_3$) of compound **(3*R*)-CF_1-3**. *See procedure*.

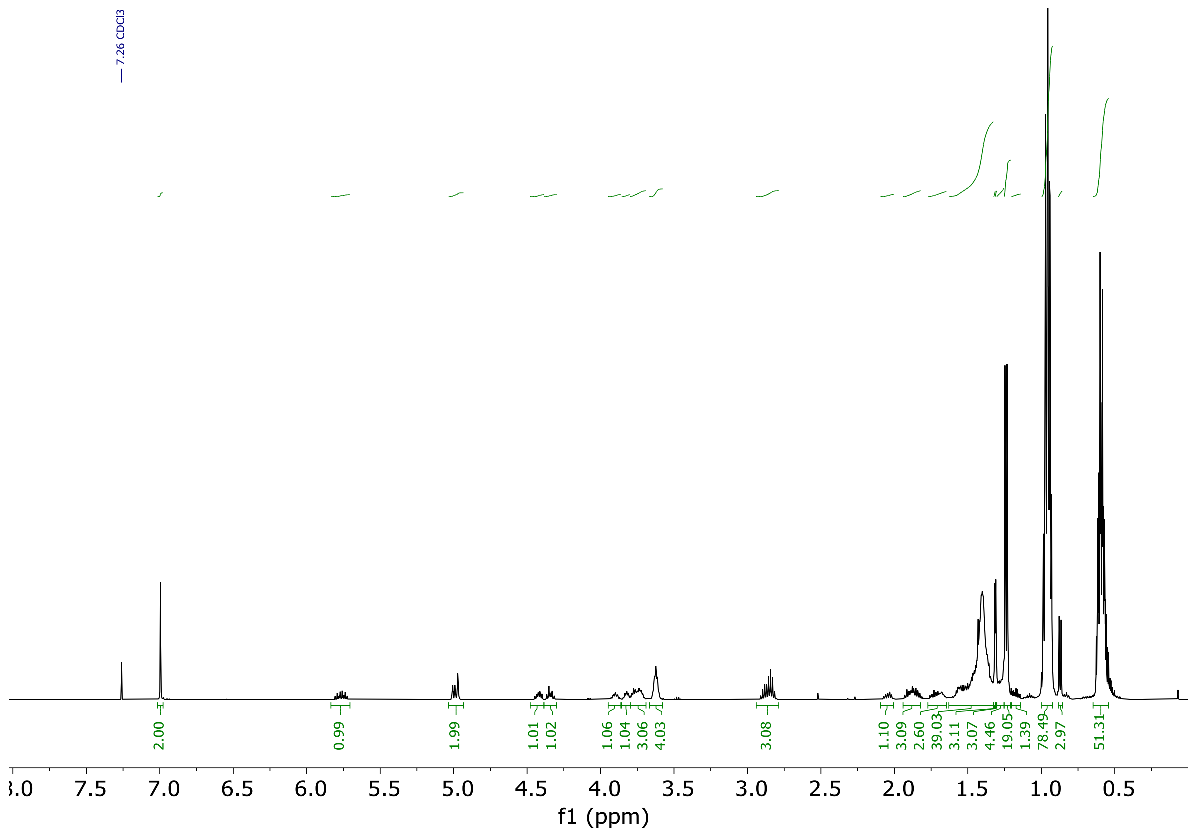

^{13}C NMR (125 MHz, $CDCl_3$) of compound **(3*R*)-CF_1-3**. *See procedure*.

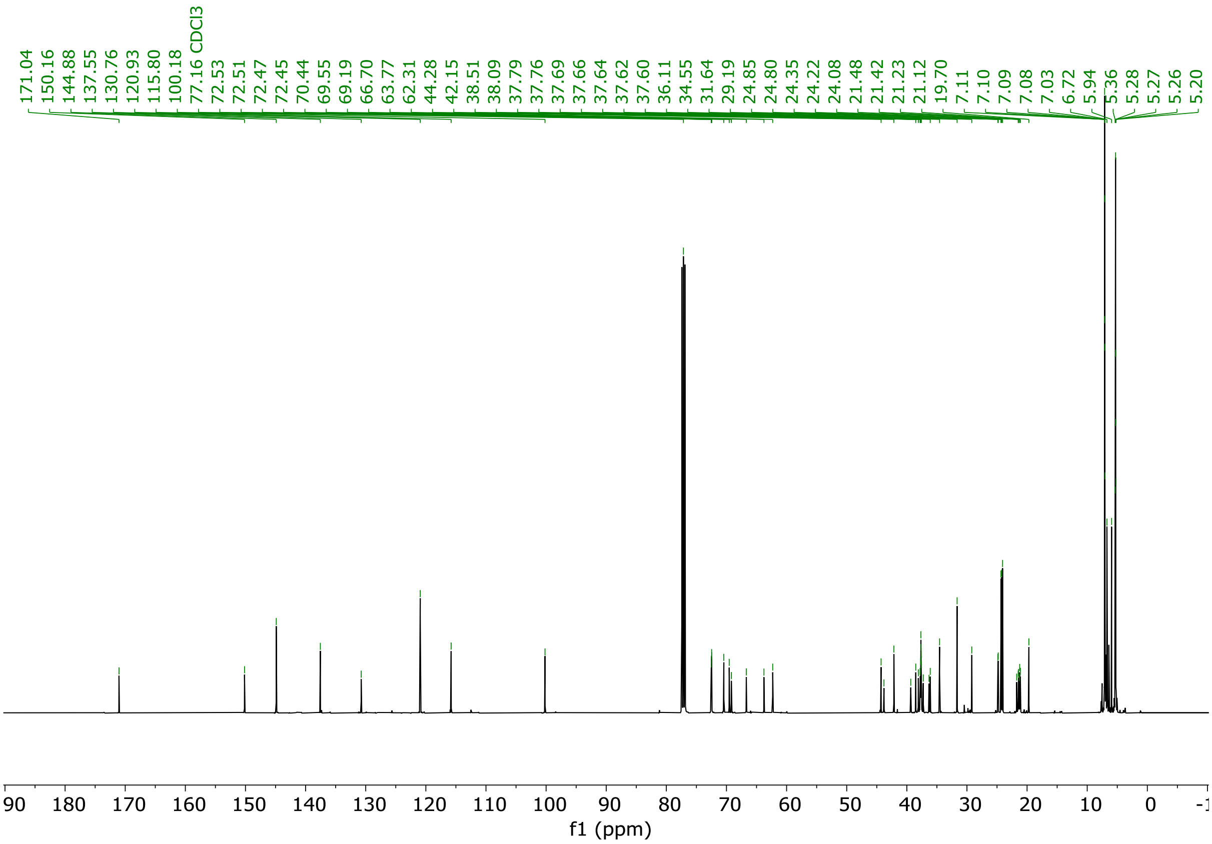

^{1}H NMR (600 MHz, $CDCl_3$) of compound **(3*R*, 36*S*/*R*)-CF_1-4**. See procedure.

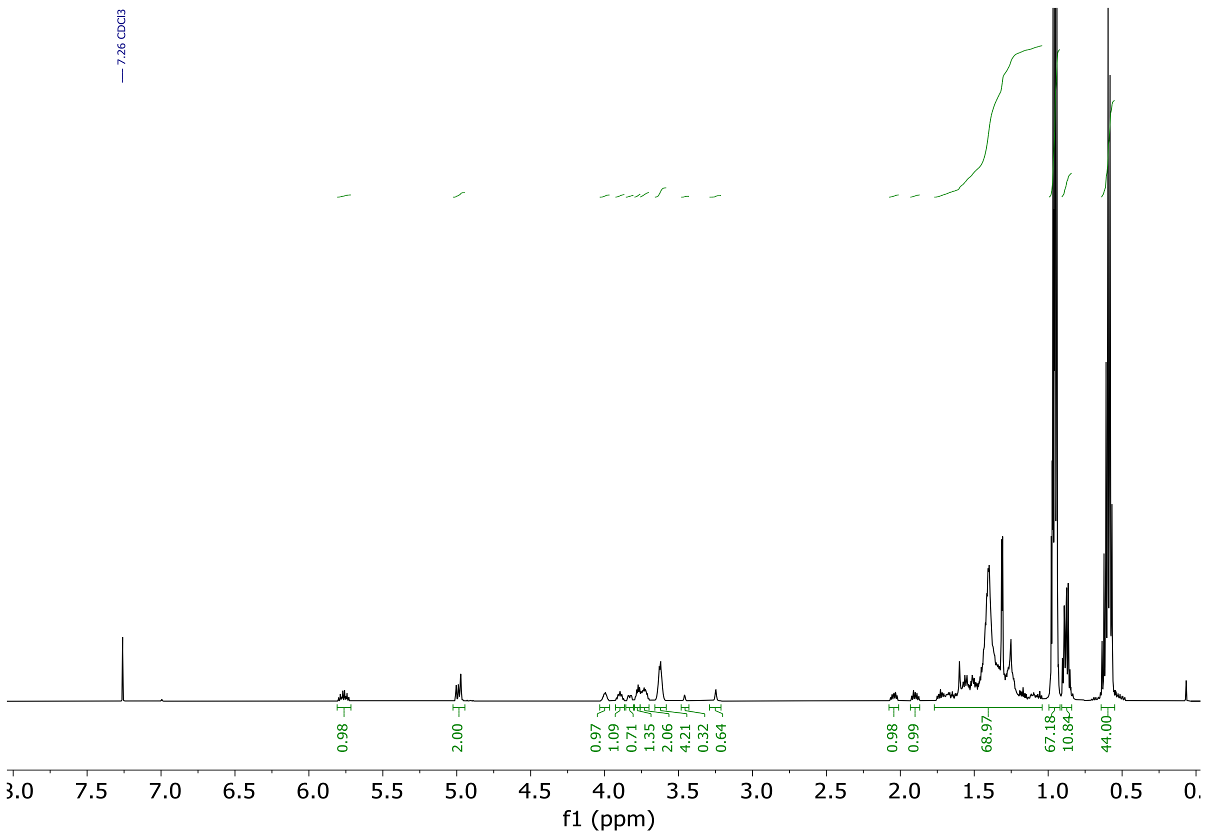

^{13}C NMR (151 MHz, $CDCl_3$) of compound **(3*R*, 36*S*/*R*)-CF_1-4**. See procedure.

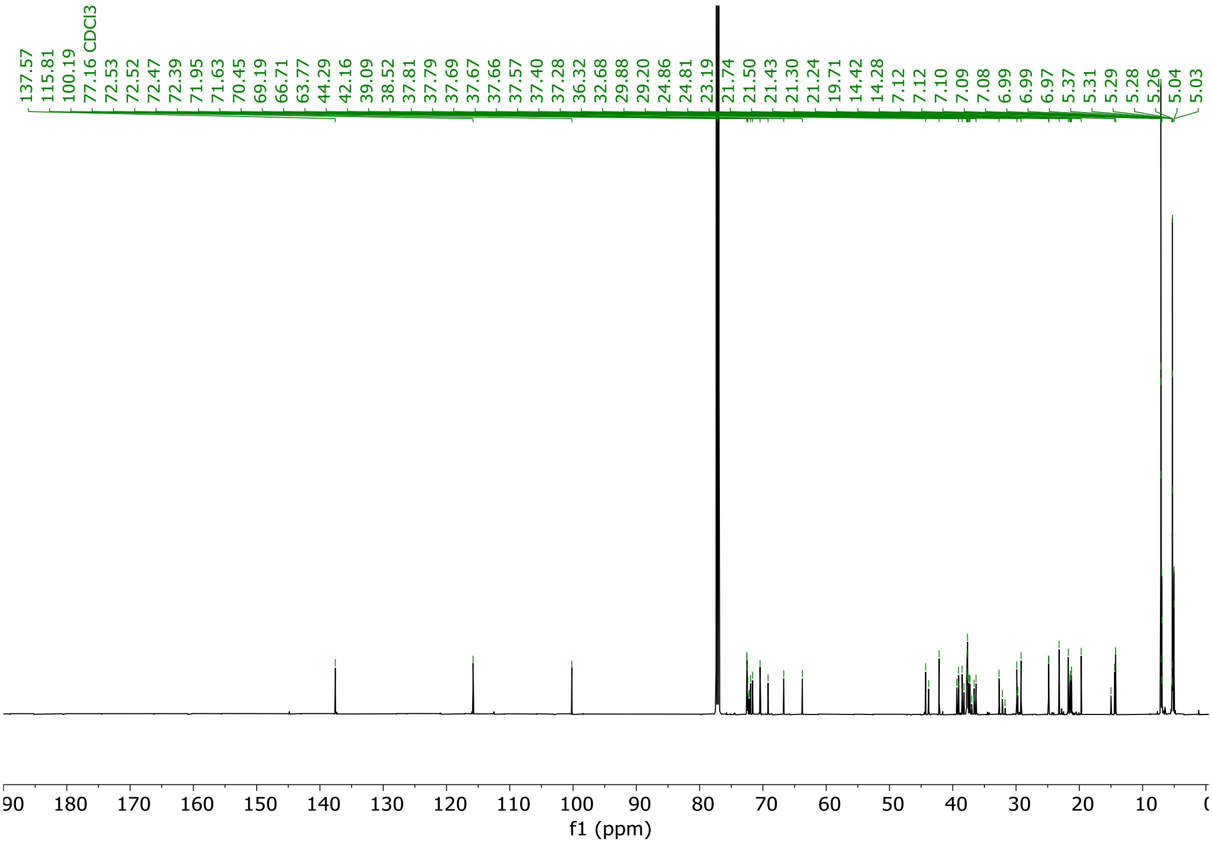

^{1}H NMR (500 MHz, $CDCl_3$) of compound **(3*S*, 36*S*/*R*)-18**. See procedure.

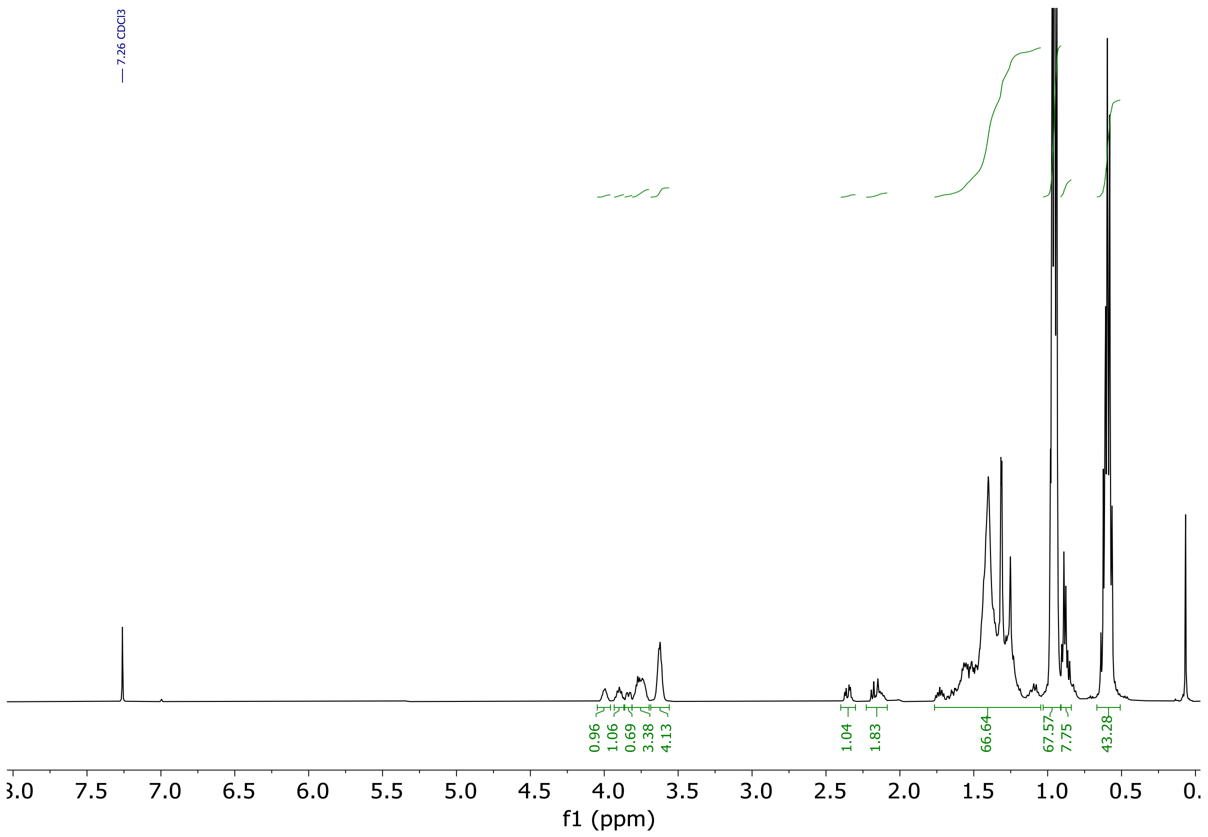

^{13}C NMR (125 MHz, $CDCl_3$) of compound **(3*S*, 36*S*/*R*)-18**. See procedure.

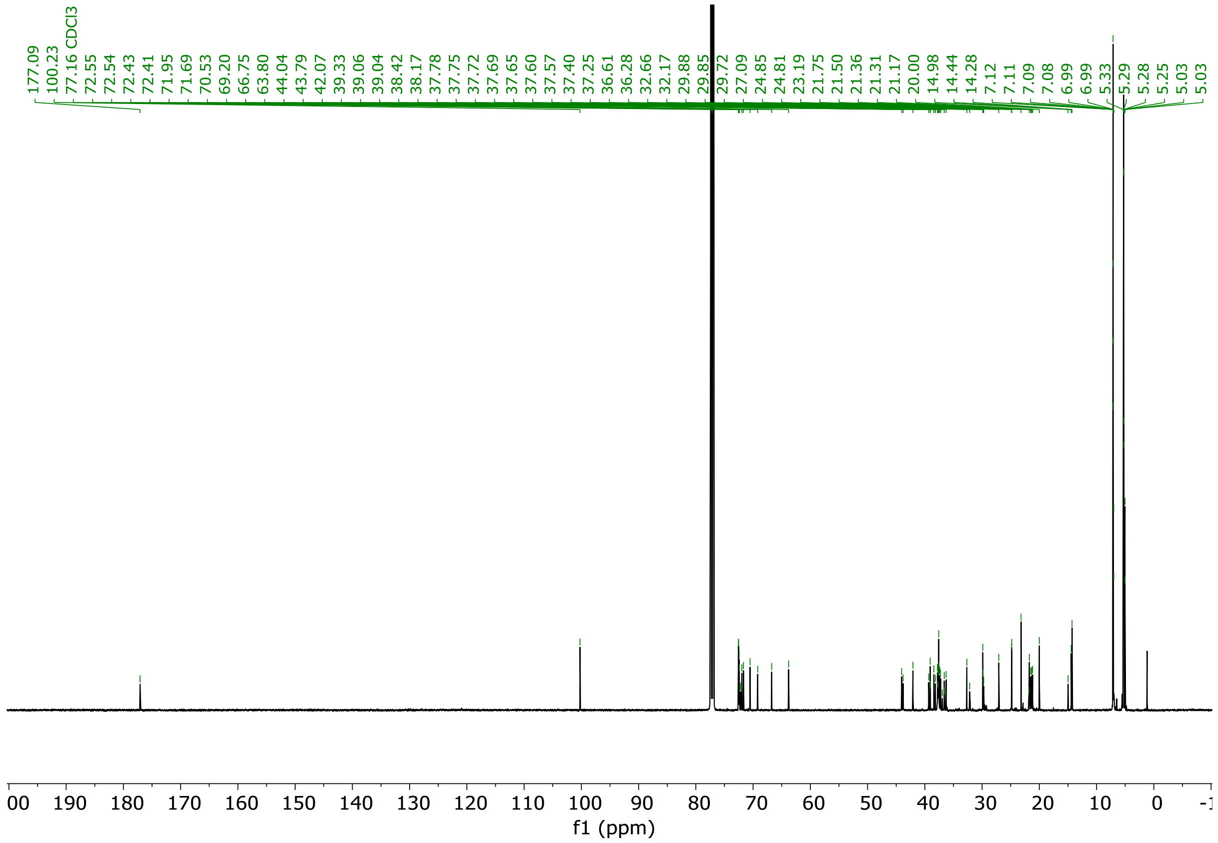

^{1}H NMR (500 MHz, $CDCl_3$) of compound **(3*S*, 36*S*/*R*)-19**. See procedure.

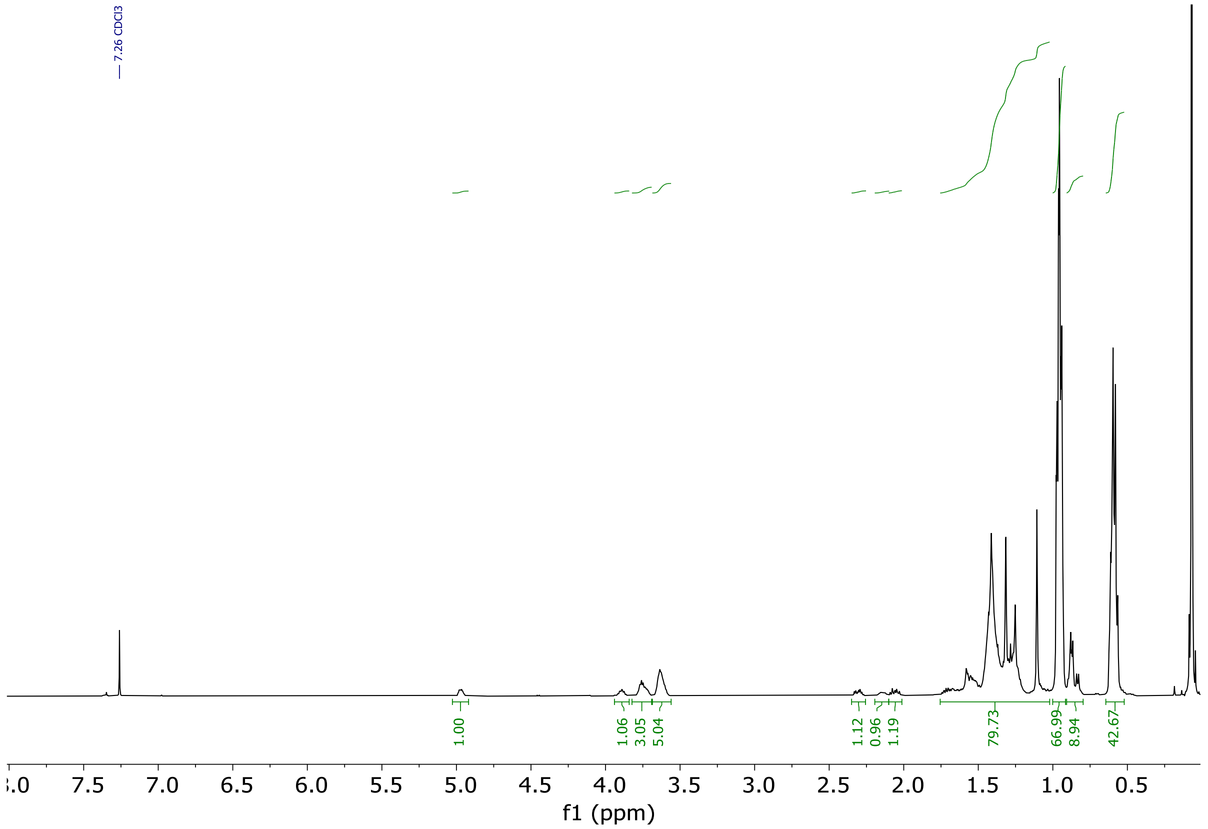

^{13}C NMR (125 MHz, $CDCl_3$) of compound **(3*S*, 36*S*/*R*)-19**. See procedure.

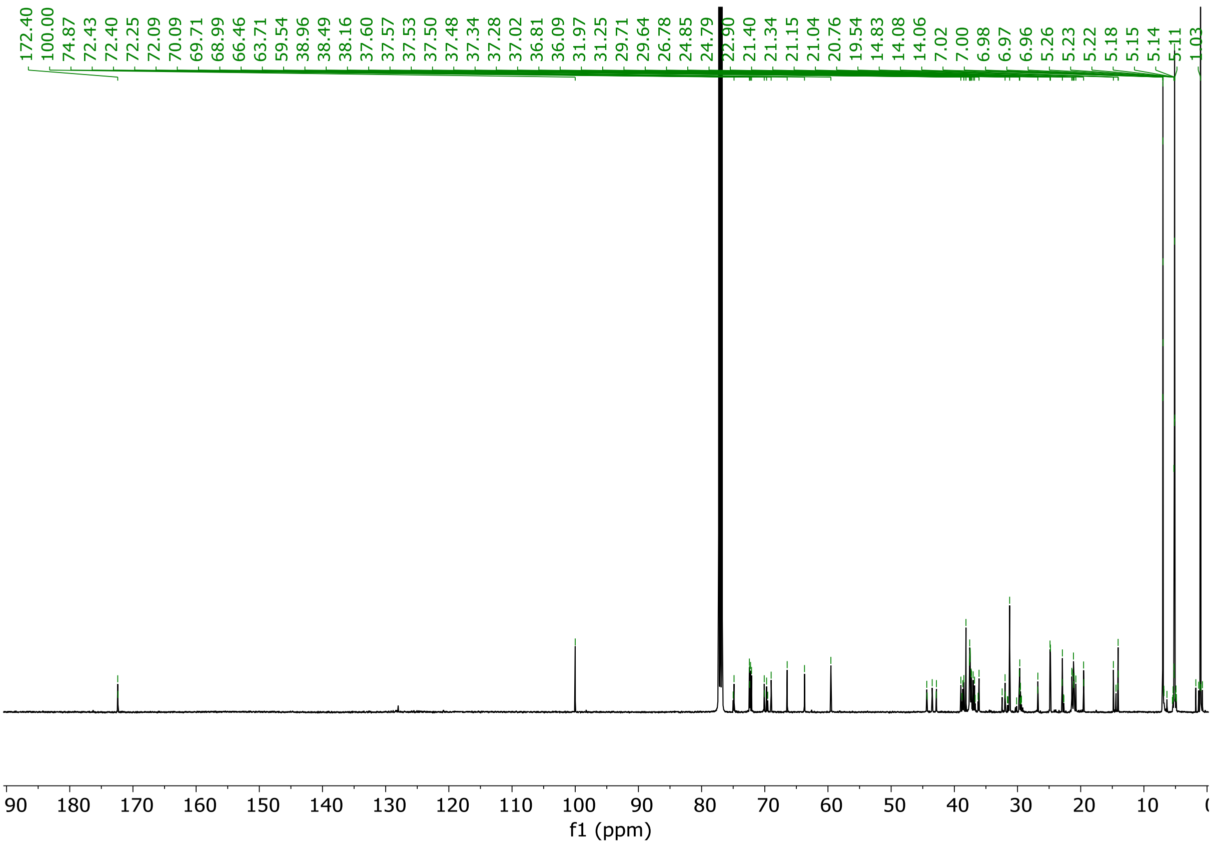

^{1}H NMR (500 MHz, pyridine-*d5*) of compound **(3*S*, 36*S/R*)-CA**. See procedure.

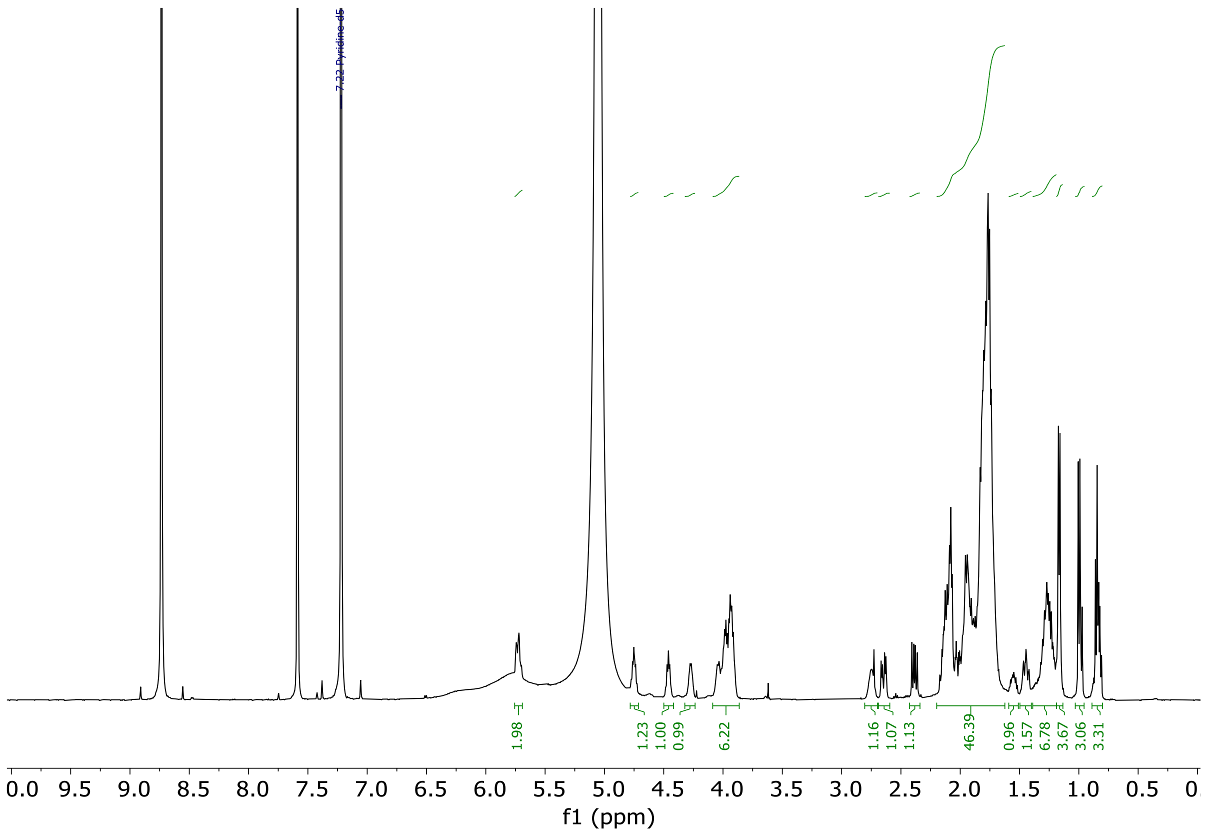

^{13}C NMR (125 MHz, pyridine-*d5*) of compound **(3*S*, 36*S/R*)-CA**. See procedure.

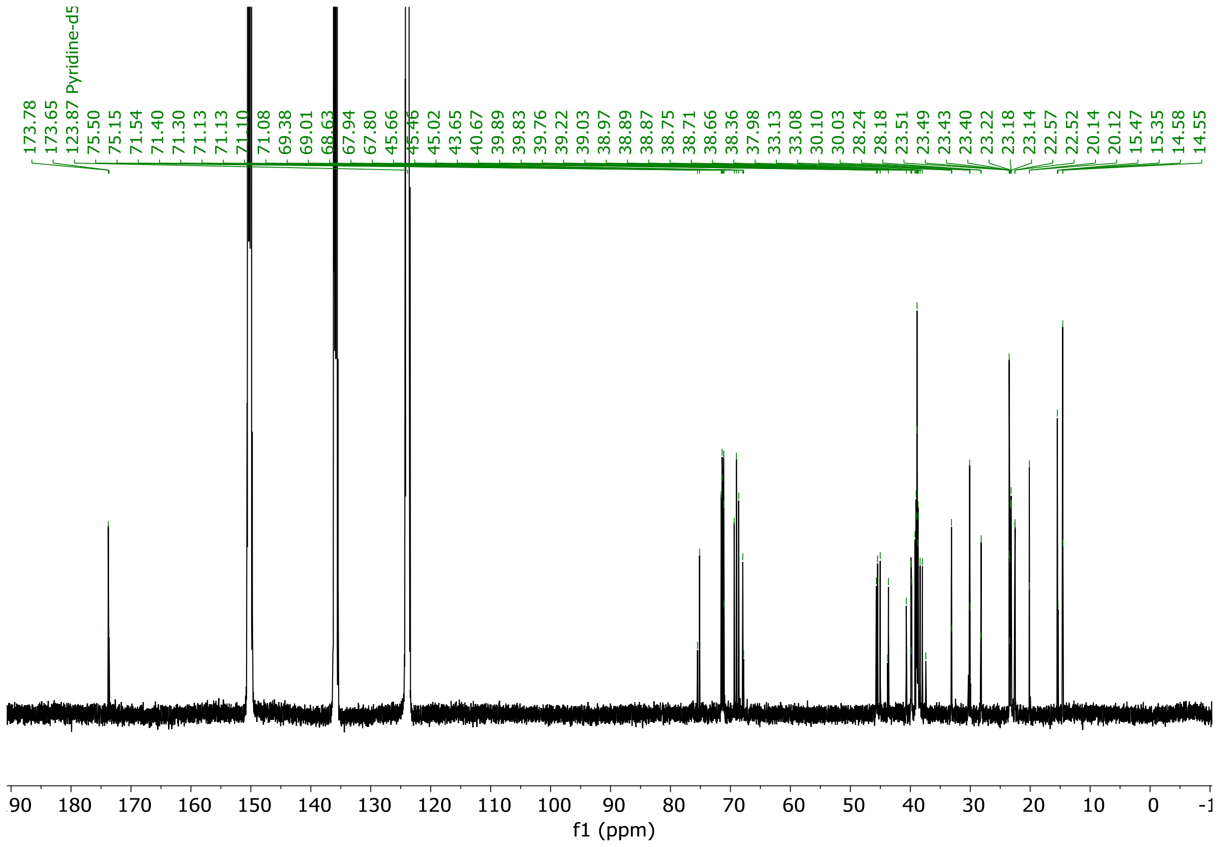

Selected chemical shifts from ^{13}C NMR spectra displaying major and minor diastereomers

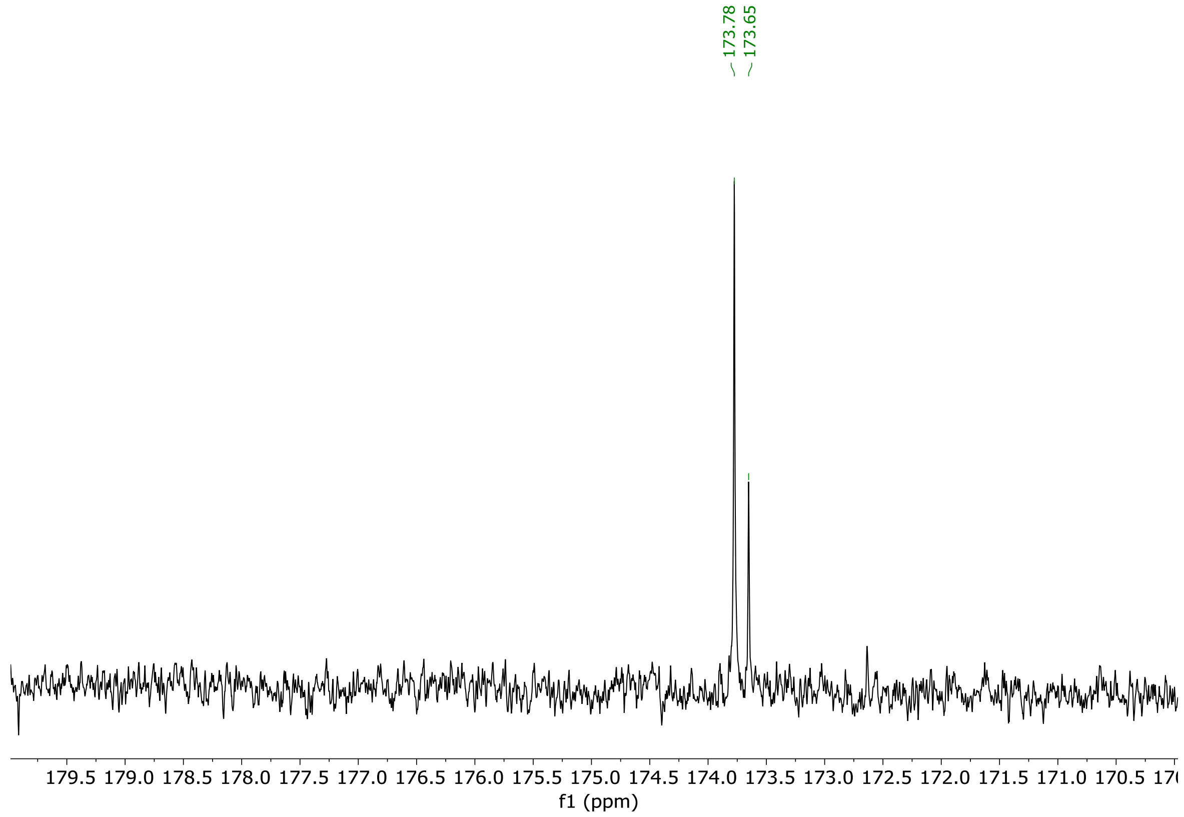

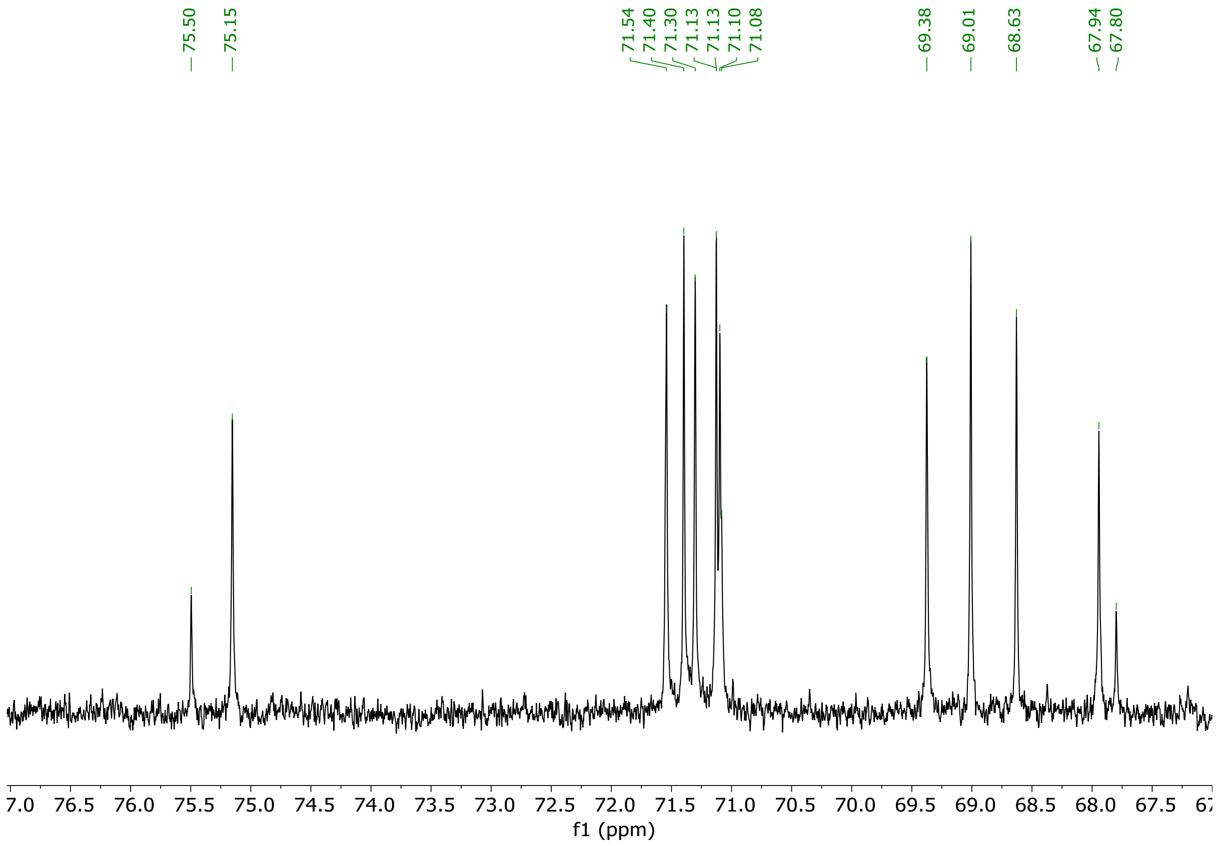

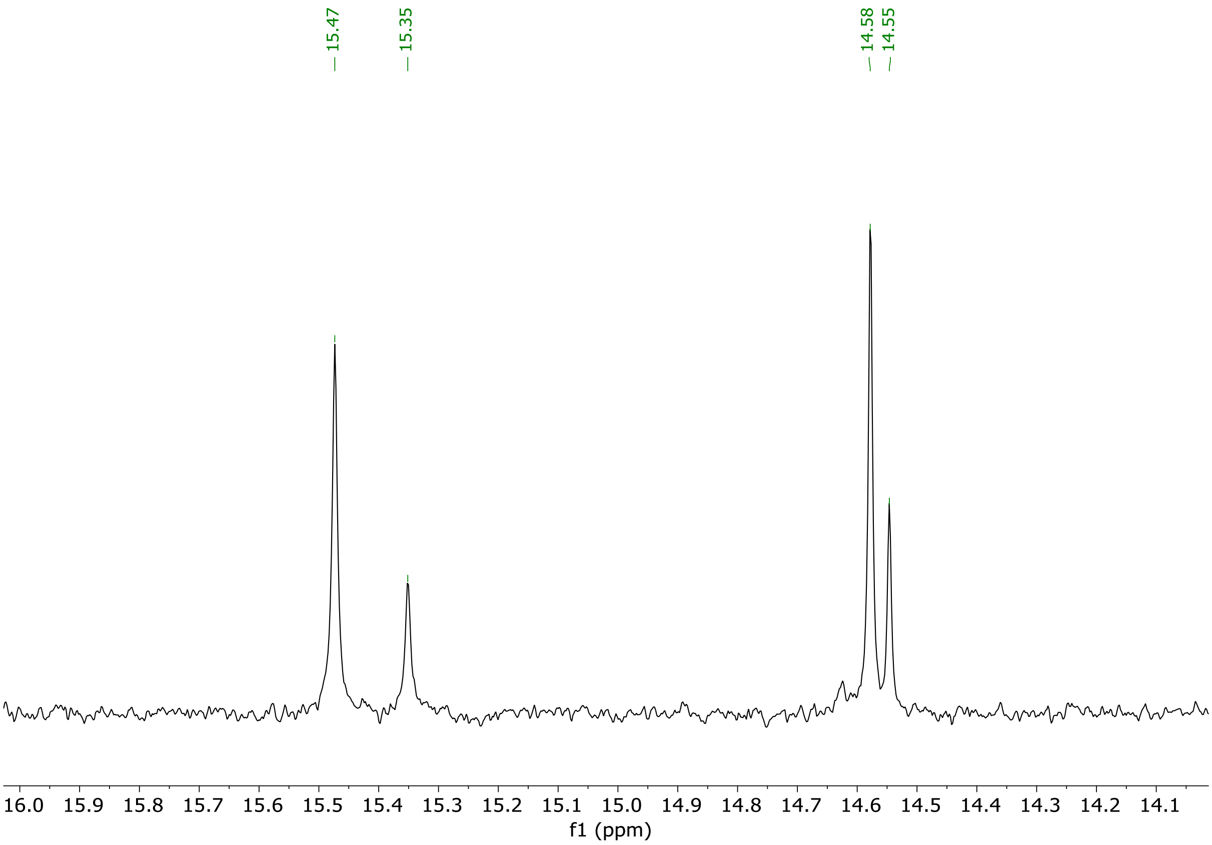

^{1}H NMR (600 MHz, $CDCl_3$) of compound **(3*R*, 36*S*)-CF_1-4**. *See procedure*.

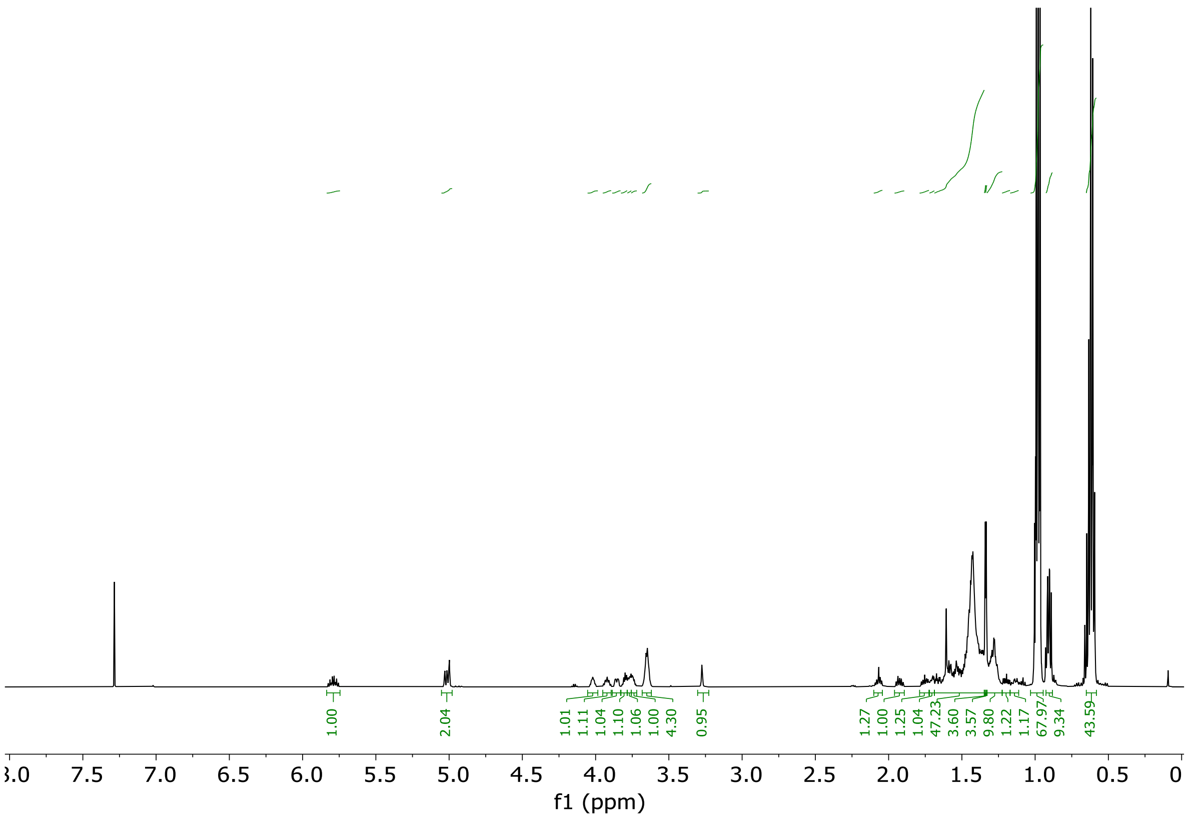

dr at C36 determined through integration of peaks below, >95:5

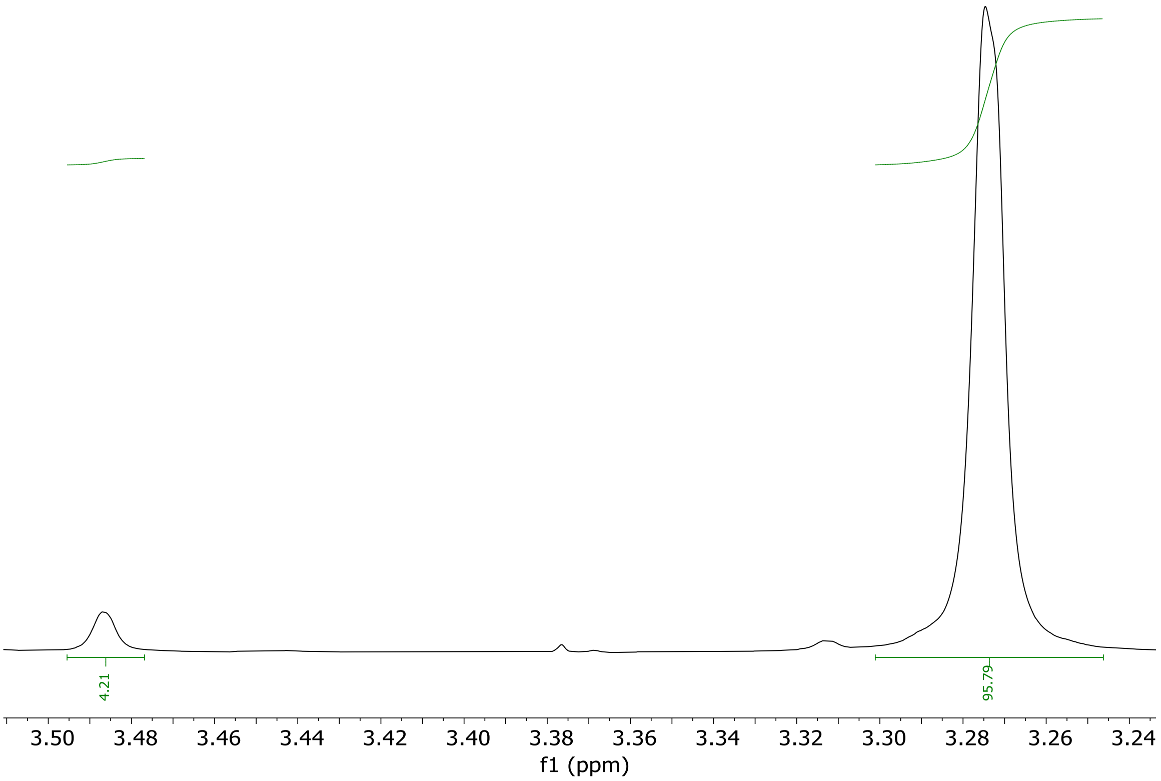

^{13}C NMR (151 MHz, $CDCl_3$) of compound **(3*R*, 36*S*)-CF_1-4**. *See procedure*.

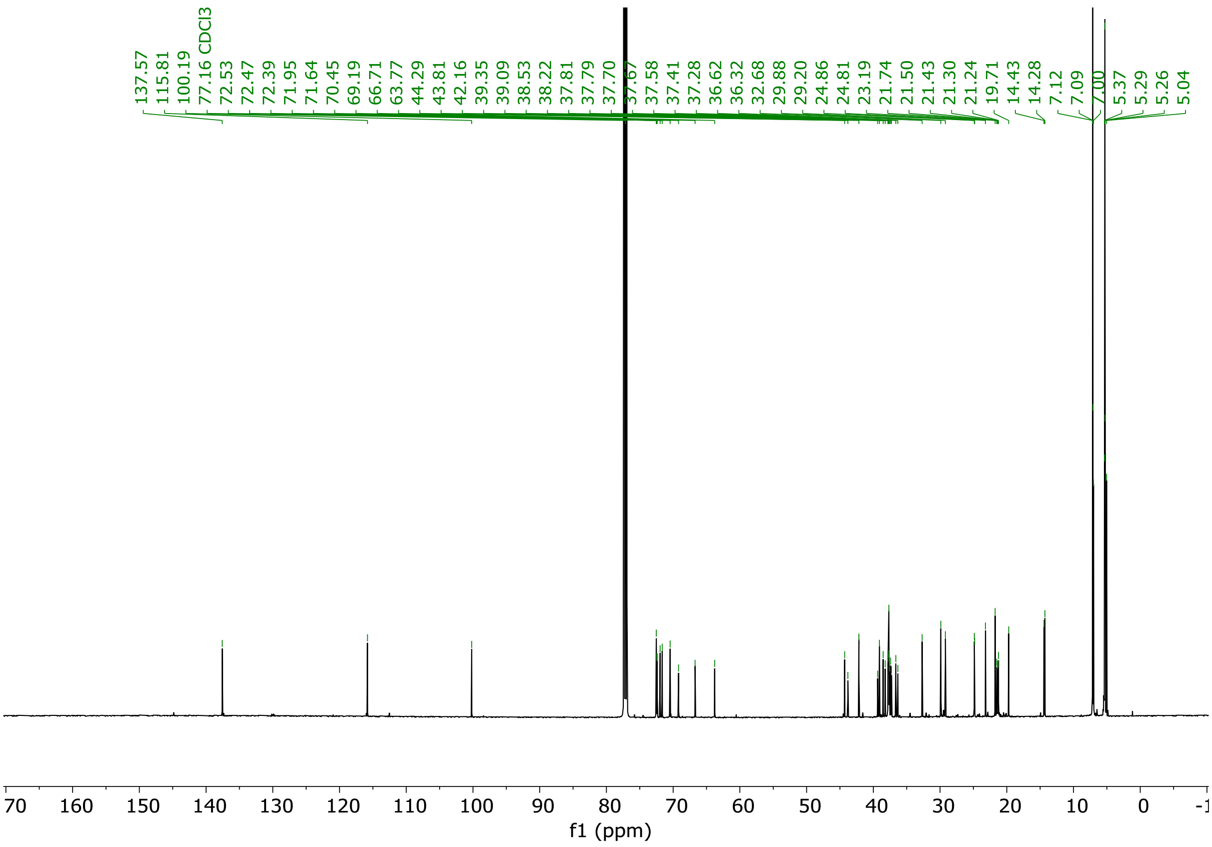

^{1}H NMR (600 MHz, $CDCl_3$) of compound **(3*S*, 36*S*)-18**. See procedure.

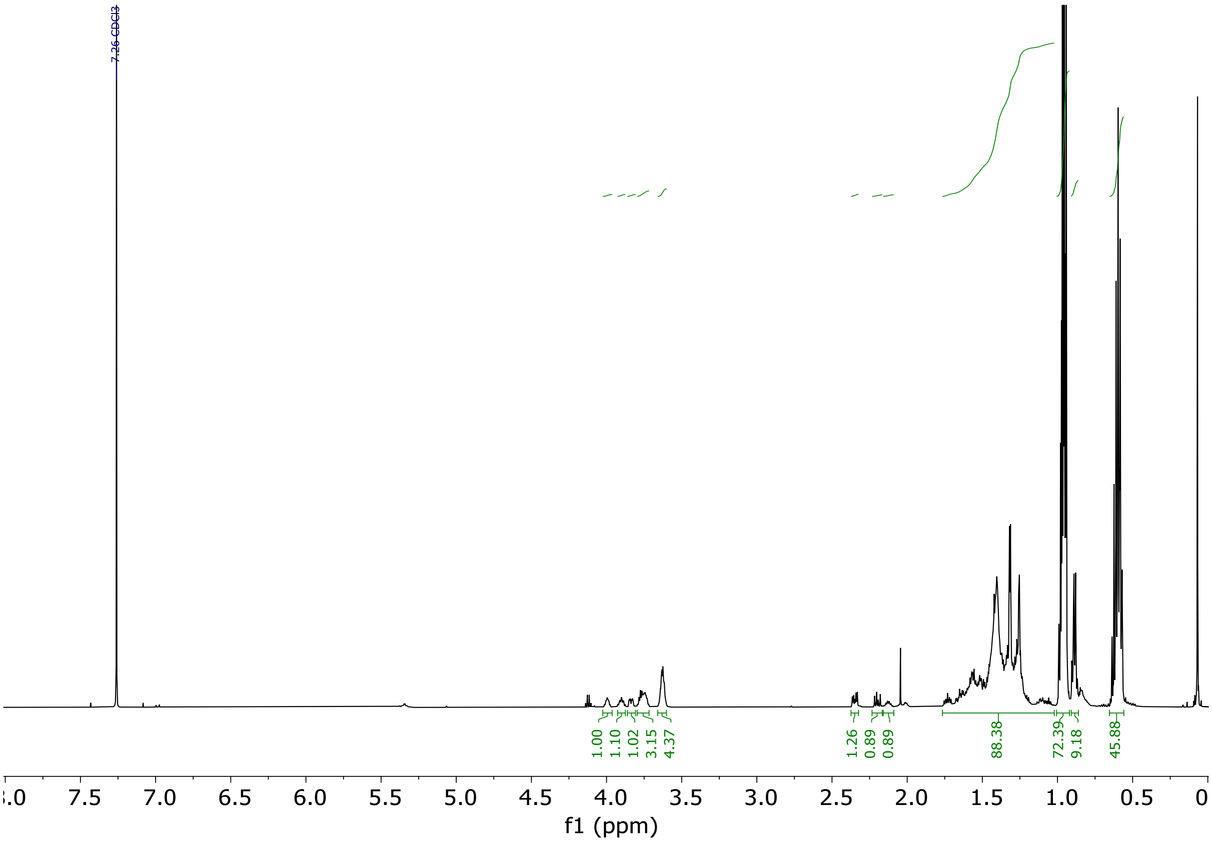

^{13}C NMR (151 MHz, $CDCl_3$) of compound **(3*S*, 36*S*)-18**. See procedure.

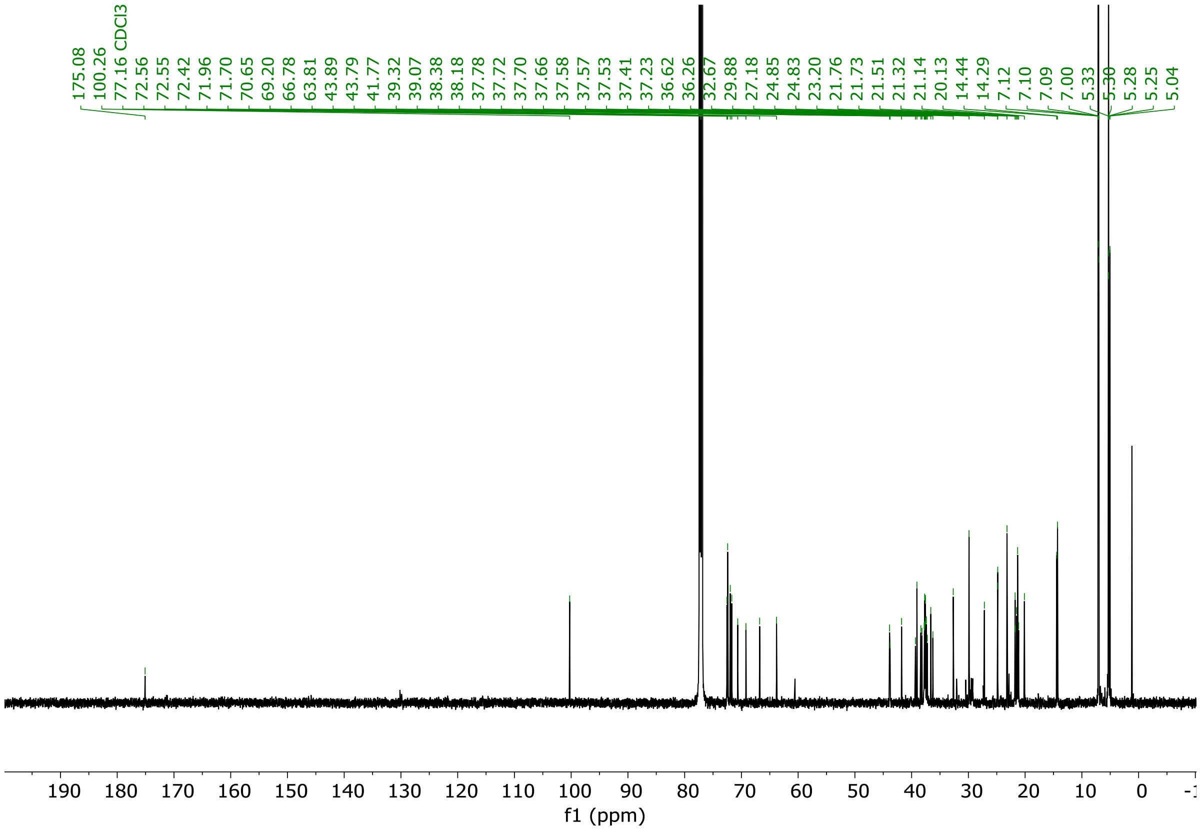

^{1}H NMR (600 MHz, $CDCl_3$) of compound **(3*S*, 36*S*)-18**. See procedure.

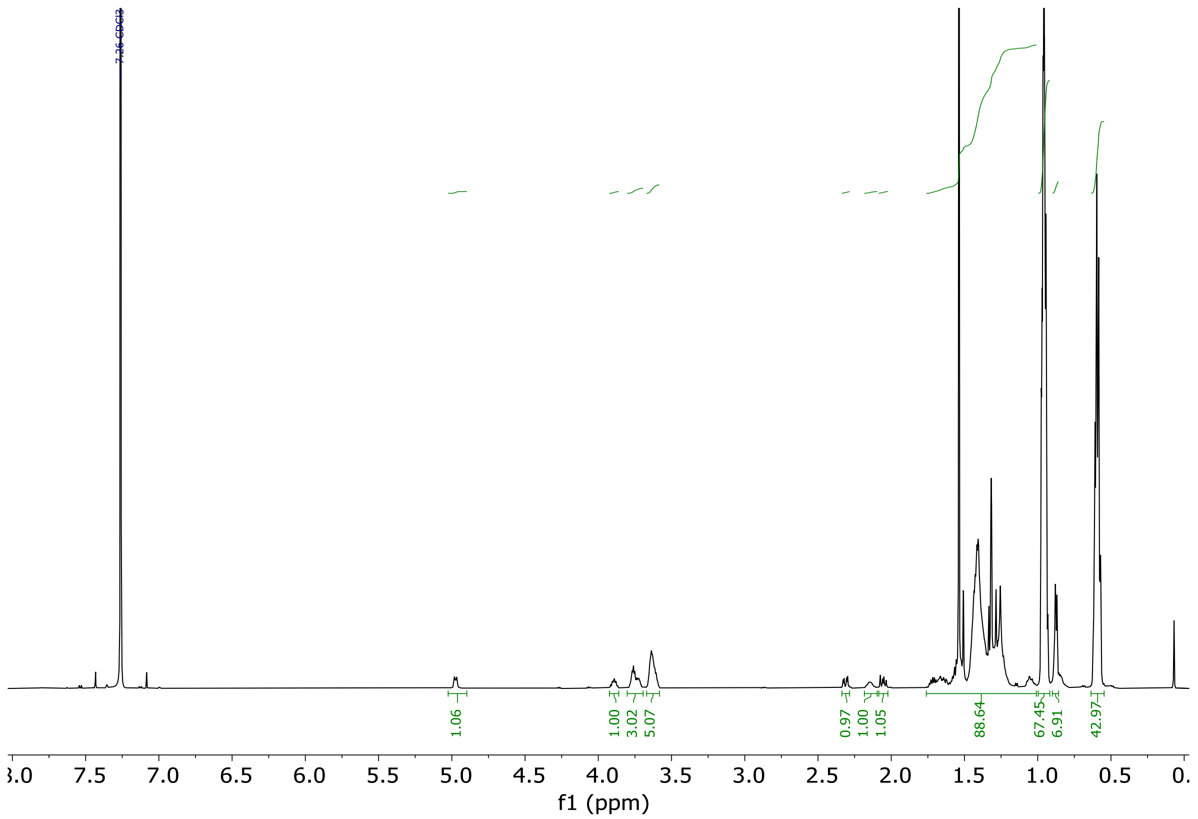

^{13}C NMR (151 MHz, $CDCl_3$) of compound **(3*S*, 36*S*)-18**. See procedure.

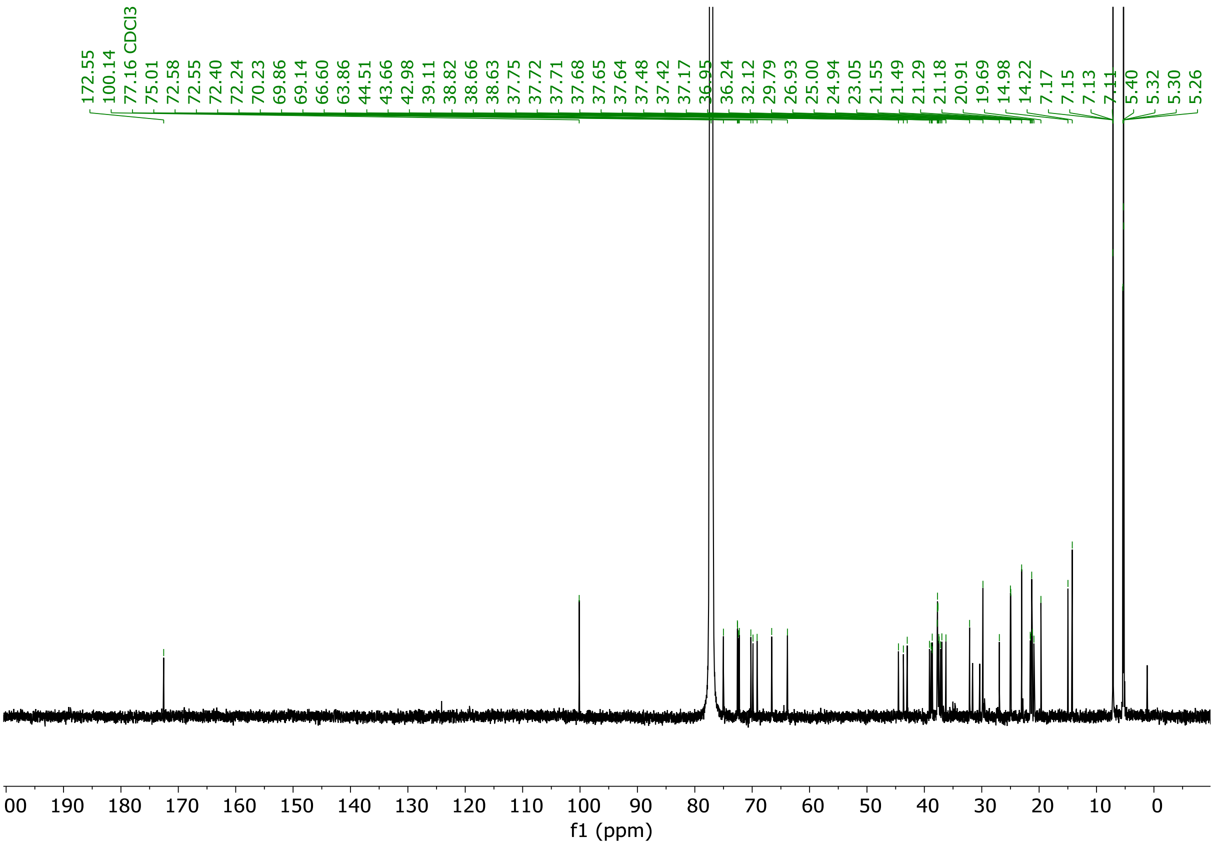

^{1}H NMR (700 MHz, pyridine-*d5*) of compound **(3*S*, 36*S*)-CA**. See procedure.

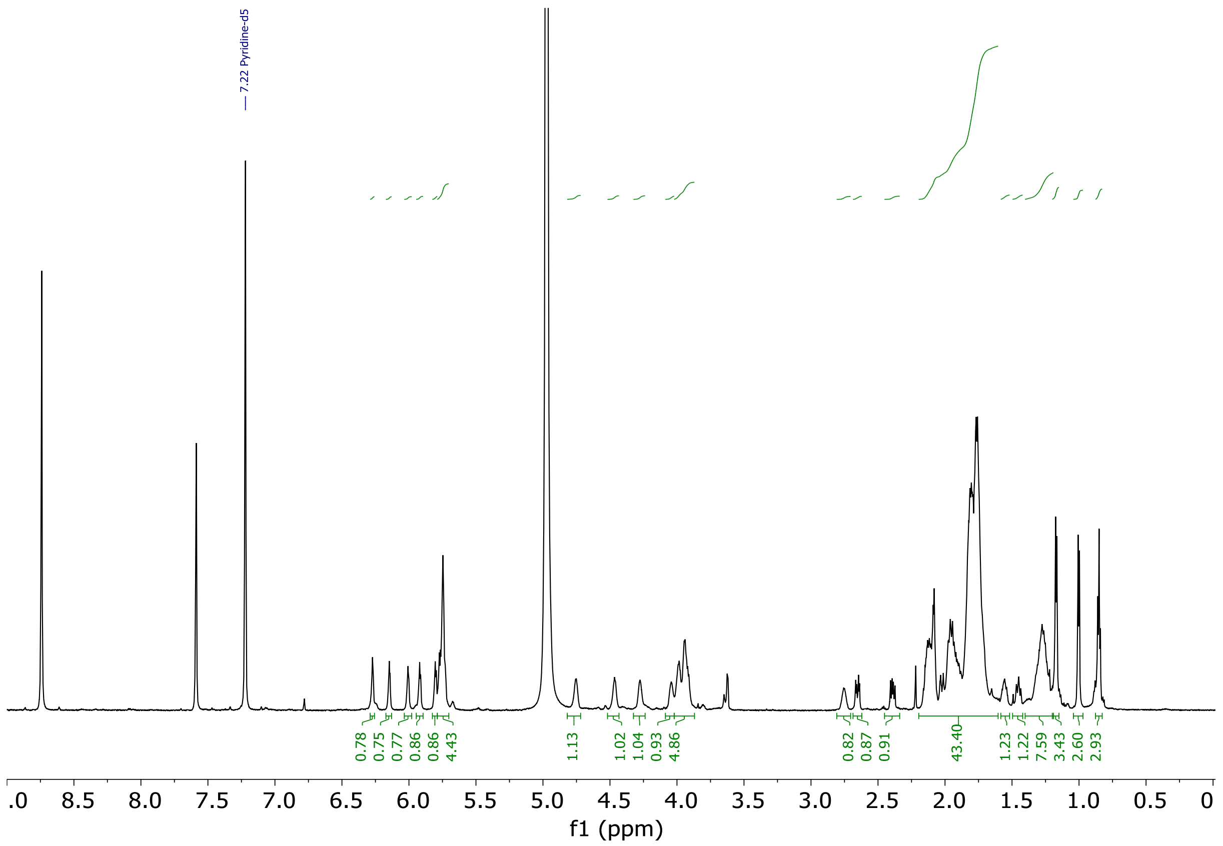

^{13}C NMR (151 MHz, pyridine-*d5*) of compound **(3*S*, 36*S*)-CA**. See procedure.

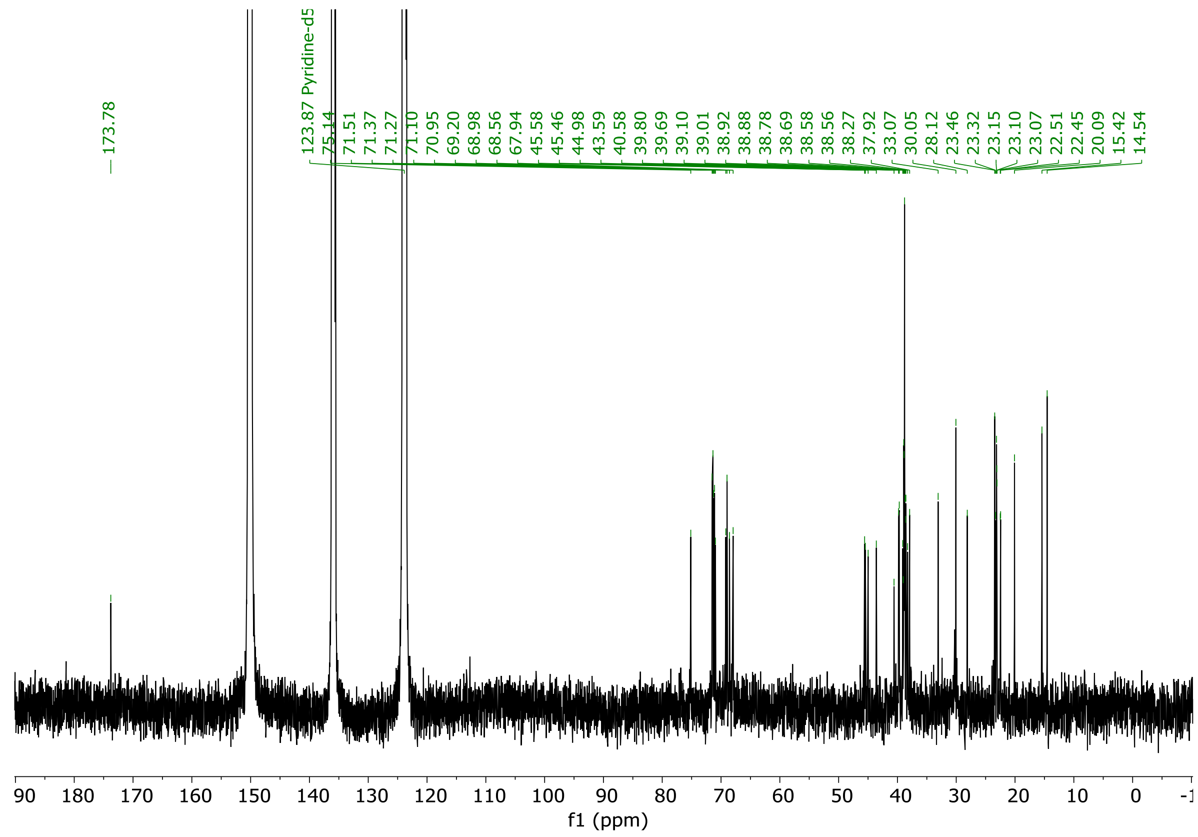

^{1}H NMR comparison of synthetic vs naturally occurring caylobolide A

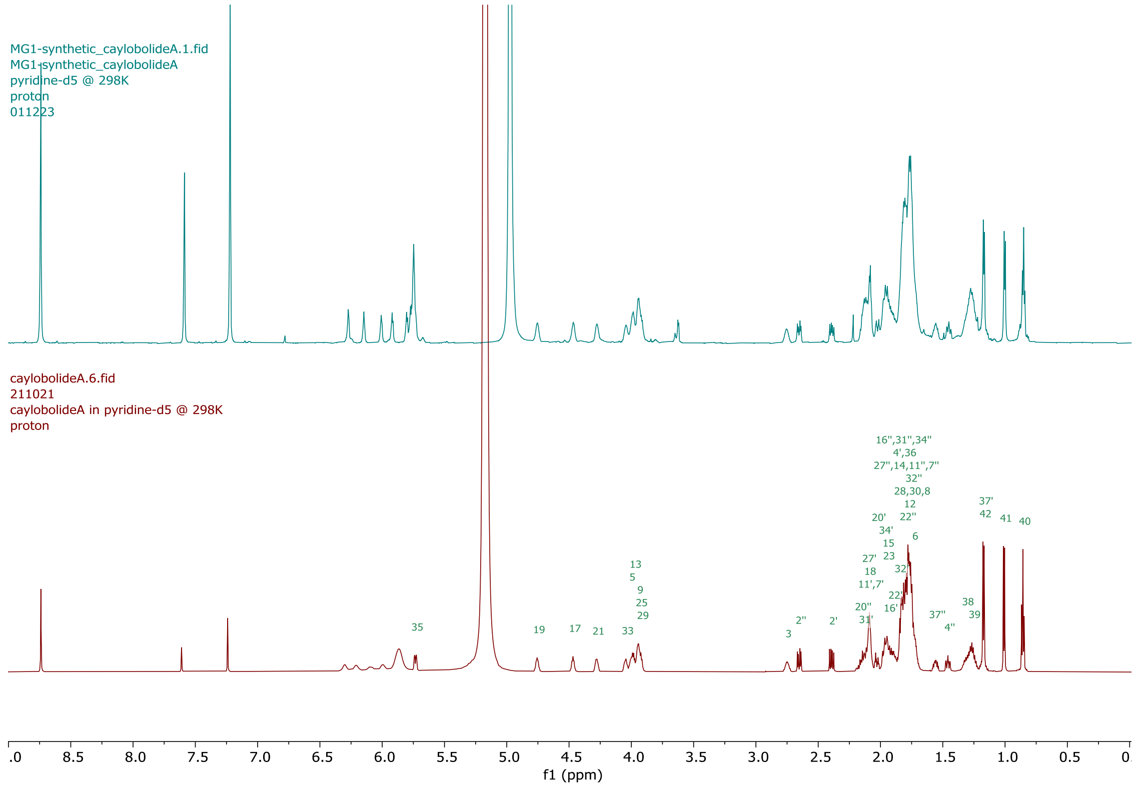

Figure S20: Full ^{1}H NMR spectral comparison of synthetic and naturally occurring caylobolide A in pyridine-*d*5 (700 MHz).

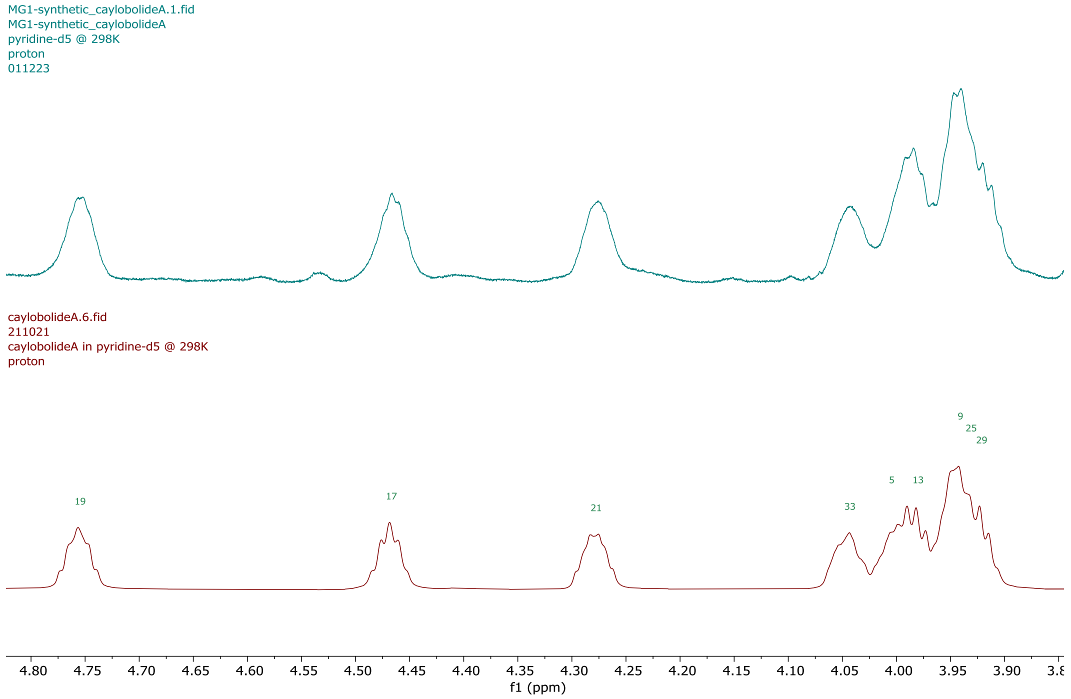

Figure S21: ^{1}H NMR spectral comparison of the C*H*OH region of synthetic and naturally occurring caylobolide A in pyridine-*d*5 (700 MHz).

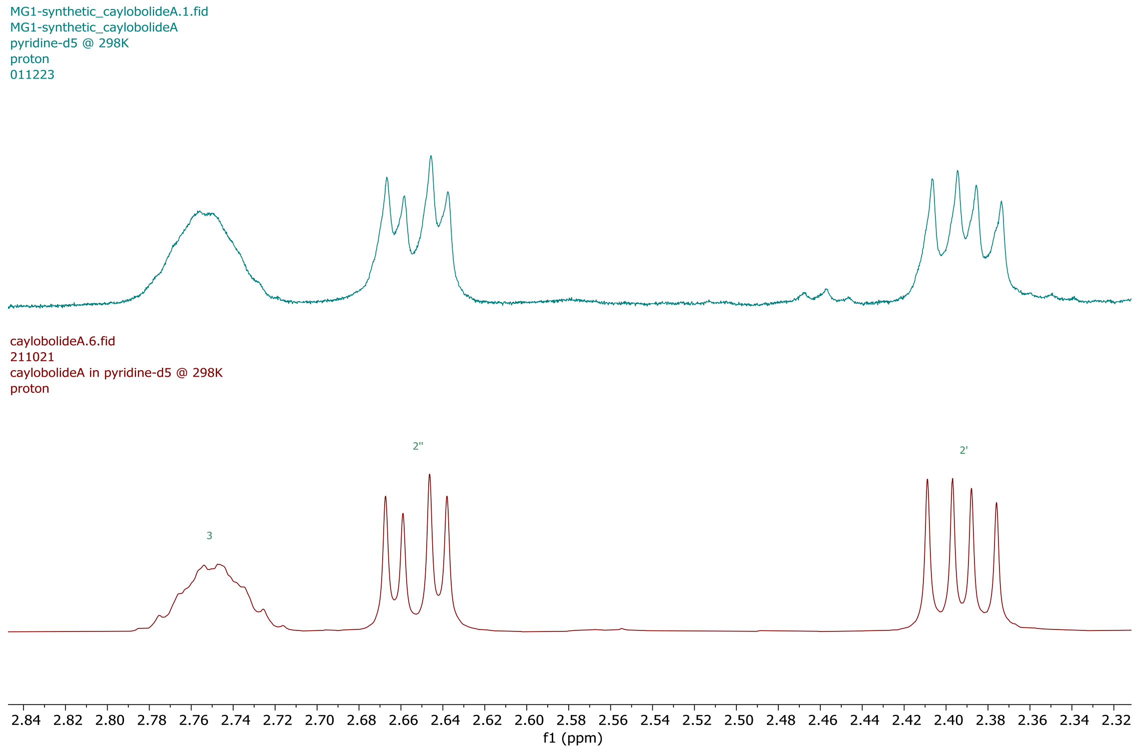

Figure S22: ^{1}H NMR spectral comparison of C*H*$_2$-2 and C*H*-3 of synthetic and naturally occurring caylobolide A in pyridine-*d*5 (700 MHz).

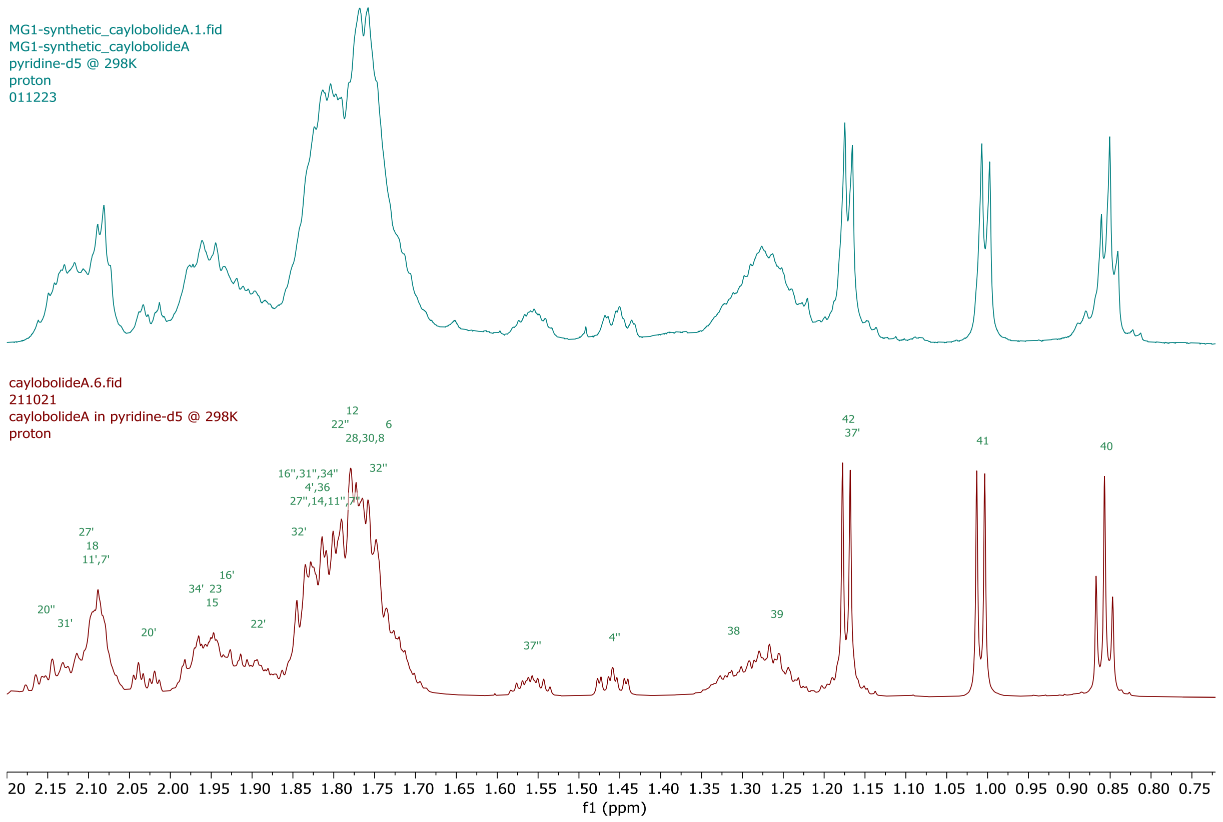

Figure S23: ^{1}H NMR spectral comparison of the alkyl region (0.7-2.2 ppm) of synthetic and naturally occurring caylobolide A in pyridine-*d*5 (700 MHz).

^{13}C NMR comparison of synthetic vs naturally occurring caylobolide A

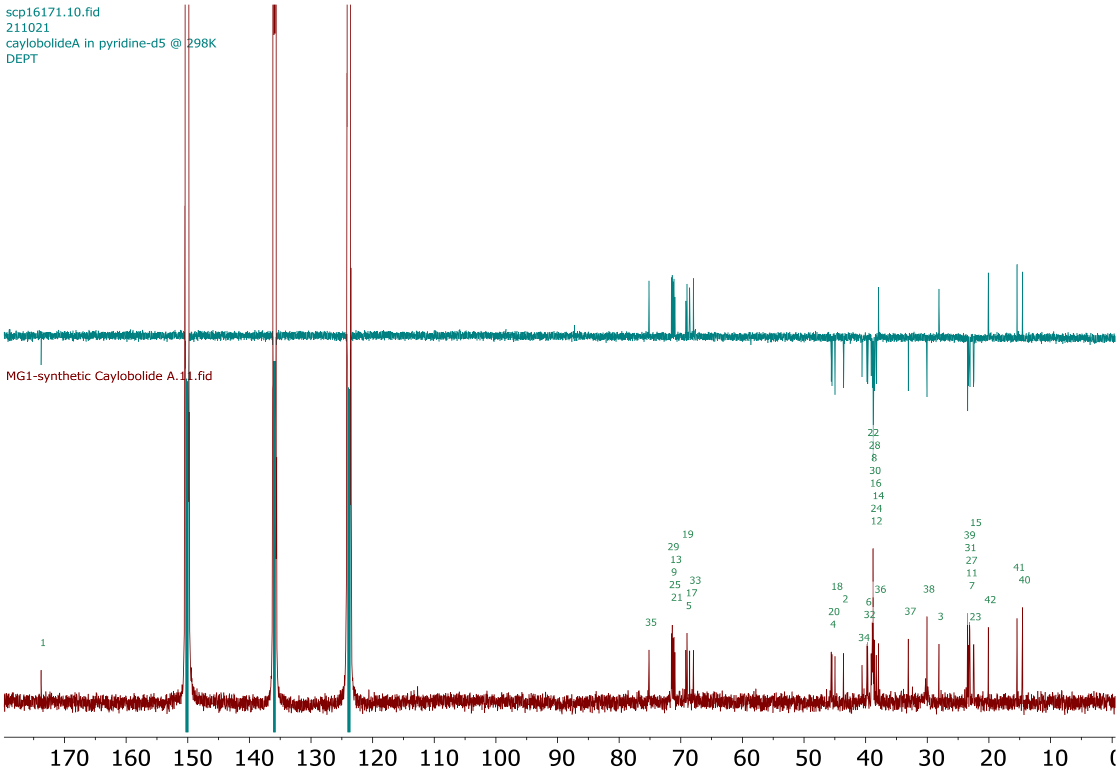

Figure S24: Full ^{13}C NMR spectral comparison of synthetic and naturally occurring caylobolide A in pyridine-*d*5 (151 Hz synthetic, 176 MHz natural).

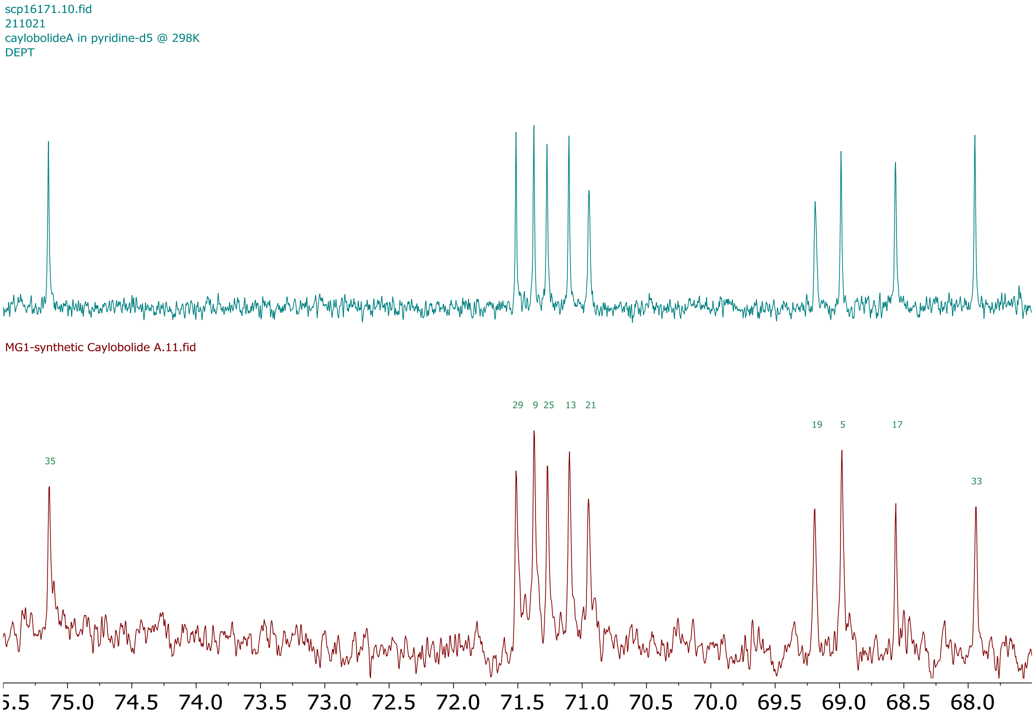

Figure S25: ^{13}C NMR spectral comparison of the *C*HO region (67-76 ppm) of synthetic and naturally occurring caylobolide A in pyridine-*d*5 (151 Hz synthetic, 176 MHz natural).

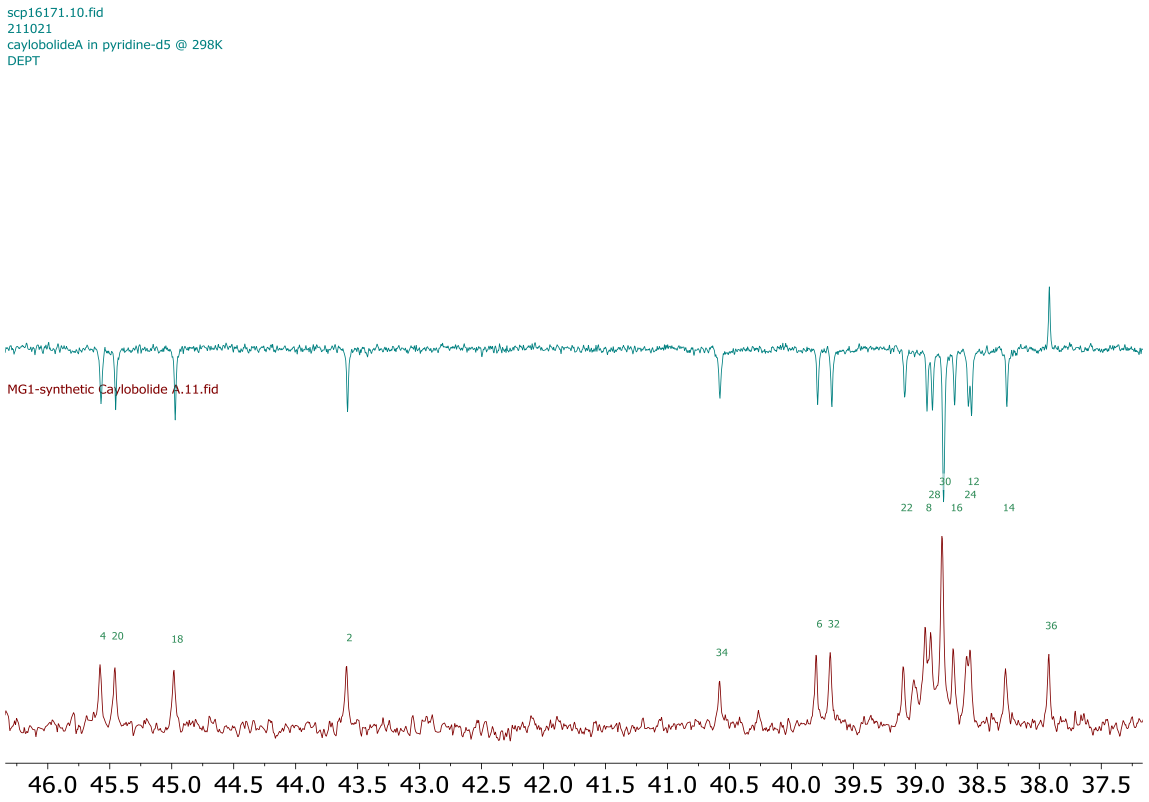

Figure S26: ^{13}C NMR spectral comparison of the α-CH_2 methylene region (36-46 ppm) of synthetic and naturally occurring caylobolide A in pyridine-*d*5 (151 Hz synthetic, 176 MHz natural).

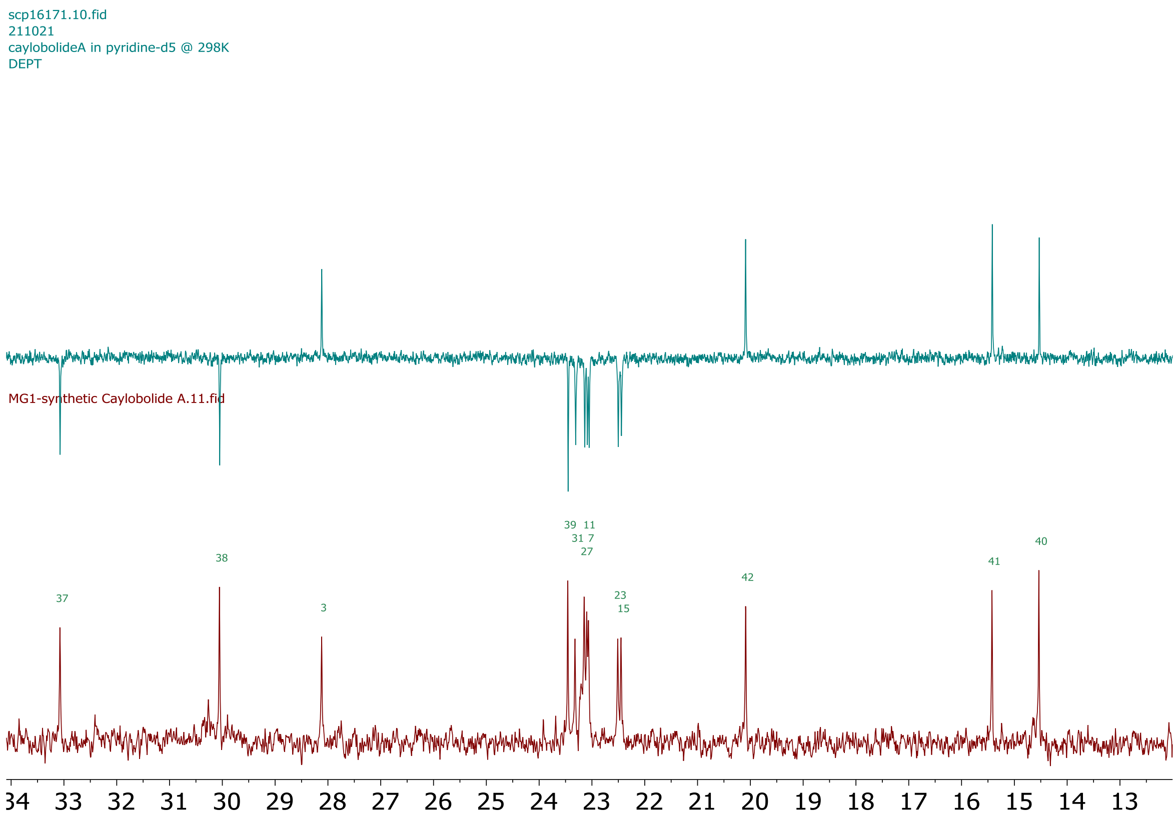

Figure S27: ^{13}C NMR spectral comparison of the β-CH_2 methylene, methyl and *C*-3 region (12-34 ppm) of synthetic and naturally occurring caylobolide A in pyridine-*d*5 (151 Hz synthetic, 176 MHz natural).
